# Comparative phenomics: a new approach to study heterochrony

**DOI:** 10.3389/fphys.2023.1237022

**Published:** 2023-11-06

**Authors:** Jamie C. S. McCoy, John I. Spicer, Simon D. Rundle, Oliver Tills

**Affiliations:** Marine Biology and Ecology Research Centre, School of Biological and Marine Sciences, University of Plymouth, Plymouth, United Kingdom

**Keywords:** phenomics, heterochrony, evolution, development, embryo, bio-imaging

## Abstract

Understanding the links between development and evolution is one of the major challenges of biology. ‘Heterochronies’, evolutionary alterations in the timings of development are posited as a key mechanism of evolutionary change, but their quantification requires gross simplification of organismal development. Consequently, how changes in event timings influence development more broadly is poorly understood. Here, we measure organismal development as spectra of energy in pixel values of video, creating high-dimensional landscapes integrating development of all visible form and function. This approach we termed ‘Energy proxy traits’ (EPTs) is applied alongside previously identified heterochronies in three freshwater pulmonate molluscs (*Lymnaea stagnalis*, *Radix balthica and Physella acuta*). EPTs were calculated from time-lapse video of embryonic development to construct a continuous functional time series. High-dimensional transitions in phenotype aligned with major sequence heterochronies between species. Furthermore, differences in event timings between conspecifics were associated with changes in high-dimensional phenotypic space. We reveal EPTs as a powerful approach to considering the evolutionary importance of alterations to developmental event timings. Reimagining the phenotype as energy spectra enabled continuous quantification of developmental changes in high-dimensional phenotypic space, rather than measurement of timings of discrete events. This approach has the possibility to transform how we study heterochrony and development more generally.

## 1 Introduction

Heterochronies, changes in the timings of developmental processes between ancestors and their descendants, are proposed as an important mechanism of evolutionary change, and are frequently regarded as the main process linking development to evolution (Gould, 1977; Gould, 1982; McKinney, 1988; Smith, 2001; Smith, 2003; Keyte and Smith, 2014). Heterochrony research typically involves comparing the timings of development between extant, closely related taxa, and subsequently inferring evolutionary change by mapping these changes to a phylogeny (Smith, 2003). A relatively recent advancement has been the use of relative timings of developmental events, and changes in their sequence relative to one another (Smith, 2001; Jeffery et al., 2002; Bininda-Emonds et al., 2002). Historically, such studies have focussed on the rates of growth of morphological structures, primarily because of the reliance on morphology by palaeontologists studying heterochrony, who are often forced to use size as a proxy for age (Gould, 1977; McKinney, 1988; Spicer and Gaston, 1999; Smith, 2001; Smith, 2003). More recently there have been calls for more integrated approaches to the investigation of developmental event timings, via the inclusion of both functional and behavioural developmental characters (Spicer, 2006; Spicer and Rundle, 2006). Incorporation of functional developmental event timings into heterochrony research has enabled changes in functional events to be examined within the evolutionary context of heterochrony (Spicer and Rundle, 2006).

Measuring the timings of discrete developmental events enables direct comparison of developmental itineraries between species, but this comes at the expense of reducing complex and dynamic developmental processes down to a single point (Burggren, 2021). Furthermore, the selection of developmental events from a vast number of candidates is; i) reliant on their occurrence in the study species of interest, and therefore must be identified *a priori* (Walls et al., 2019), and ii) potentially introduces a significant element of chance to selection of developmental events related to biological processes of interest (Houle et al., 2010; Forsman, 2015). Consequently, our understanding of the links between development and evolution via heterochrony must be limited by the current frameworks and methodologies used to investigate it. What is required is a new, more objective, way of describing and analysing heterochrony and development generally drawing on as much biological information as possible.

The capacity to use bioimaging to continuously measure phenotypic change at the organism wide scale in developing animals offers new opportunities for interrogating the lines between heterochrony as a pattern, and its role as a process in macro-evolutionary change (Burggren, 2021). Bioimaging enables researchers to apply new computer-vision approaches to measuring phenotypic change, using methods with no manual equivalents. Energy proxy traits (EPTs) are a measure of fluctuations in pixel intensities quantified as a spectrum of energies across different temporal frequencies (Welch, 1967) and are proving a valuable approach to measuring complex phenotypes in developing embryos (Tills et al., 2018; Tills et al., 2021). EPTs, rather than selecting specific aspects of an organism’s morphology, physiology or behaviour, are indiscriminate measures of the phenotype applicable to different species and experimental designs that can be followed continuously during the course of development. As a method of quantifying features of developing embryos they therefore overcome the limitations associated with measuring discrete points in development time. EPTs are effective at capturing developmental transitions in embryos of an aquatic invertebrate, where traditional phenotypic measures are largely ineffective or non-transferable between stages of development. There is also evidence to suggest that they are indicative of energy turnover at the biochemical level (Tills et al., 2021). However, so far EPTs have not been used to compare species with different developmental itineraries, to investigate high-dimensional phenotypic change associated with evolutionary differences in the timings of development.

Consequently, our aim here is to present an alternative way of describing and analysing heterochrony, by investigating the extent to which evolutionary differences in the timings of developmental events (heterochronies) are associated with high-dimensional phenotypic change, using EPTs. We hypothesise that the onset of developmental events are associated with changes in EPT spectra, and that evolutionary differences in the timings of developmental events are reflected in time series of EPT spectra. To do this, we measured interspecific differences in EPTs in encapsulated embryos of species within a well-resolved phylogeny. Species of freshwater pulmonate snail exhibit sequence heterochronies at the familial level (Smirthwaite et al., 2007), thereby providing a tractable model system. Smirthwaite et al. (2007) investigated event timings in 13 species across three families and detected significant sequence heterochronies in embryos of the Lymnaeidae and Physidae. Embryos of the physid *Physella acuta* exhibit sequence heterochronies in the timings of muscular crawling and cardiac function, relative to embryos of the lymnaeids *Lymnaea stagnalis* and *Radix balthica*, specifically an earlier onset of cardiac function relative to muscular crawling in the two lymnaeids. Therefore, we used EPTs to characterise high-dimensional changes in observable phenotype throughout embryonic development in these species, alongside measuring the timings of major functional developmental events: i) the onset of ciliary driven rotation; ii) the onset of cardiac function; iii) attachment to the wall of the egg capsule and the transition to muscular crawling; iv) the onset of radula function (referred to throughout as rotation, heart, crawling and radula respectively) (Smirthwaite et al., 2007).

## 2 Materials and methods

### 2.1 Animal collection and maintenance

Adult snails *L. stagnalis* and *P. acuta* were collected using a sweep net (1 mm mesh) from Exeter canal and nearby streams, Devon, United Kingdom (50°41′57.8″N 3°30′43.7″W). Adult *R. balthica* were collected using the same method from drainage canals, Middle Furlong Rhynie Bridgwater, UK (51°11′23.9″N 2°52′47.9″W). Snails were immediately transferred to the laboratory in plastic containers containing water and pondweed within 24 h of collection. Upon arrival snails were divided between a number of plastic containers (volume = 4 L) each filled with continuously aerated artificial pond water (CaSO_4_—120 mg L^−1^, MgSO_4_—245 mg L^−1^, NaHCO_3_—192 mg L^−1^, KCl—8 mg L^−1^), and maintained at T = 15°C. Snails were fed spinach and lettuce *ad libitium*. Snails were acclimated to laboratory conditions for a minimum of 1 week prior to experimentation under a 12 h light/12 h dark light regime, and with weekly water changes.

### 2.2 Embryo collection

Snails regularly deposited egg masses onto the walls and floor of rearing aquaria. These masses were carefully removed using a piece of thin laminate plastic. When viewed under low power magnification (10–40×) any eggs that had not developed past the 4-cell stage were removed. Embryos from a minimum of 3 egg masses were used for each species in order to account for any brood variation. Individual embryos were carefully removed from their egg masses selected haphazardly before being transferred into individual wells of microtitre plates containing artificial pond water (Nunc, Microwell, 96 wells, 350 µL per well).

### 2.3 Bio-imaging

Embryonic development from the 4-cell stage to hatching was recorded using an Open Video Microscope (OpenVIM), enabling long term repeated video imaging of aquatic embryos (Tills et al., 2018). Microtitre plates containing embryos were placed into incubation chambers (H101-K-Frame, Okolab™, Italy), and reared for the duration of their embryonic development at 20°C. Temperature was controlled by circulation of water through the chamber by a temperature bath (H101-CRYO-BL, Okolab™, Italy). Water in the incubation chambers was constantly but gently aerated using an air pump (OKO AP, Okolab™, Italy). To reduce evaporation within wells of microtitre plates, air was pre-humidified using a humidity module (Okolab™, Italy). Incubation chambers were mounted onto an aluminium frame, the position of which was controlled using a motorised XY stage (SCAN 130 × 85, Märzhäuser Wetzlar™, Germany). There were mortalities of 33.3%, 16.7% and 10.4% during various points in the development of embryos of *L. stagnalis*, *R. balthica* and *P. acuta* respectively. Mortalities were excluded from analyses given that these embryos did not undergo all developmental events used in this study.

Image sequences of individual embryos were acquired using an inverted lens at ×200 magnification (VH-720R, Keyence™, United Kingdom) attached to a Charged Couple Device digital camera (resolution: 2048 × 2048 pixels, Pike F421B, Allied Vision™, Germany). Dark field illumination was achieved using an LED ring light placed above the incubation chamber (LDR2-42-SW2, CCS, United Kingdom). Raw video data some of which was published in Tills et al., 2018; Tills et al., 2021), was used for *R. balthica*. Image sequences were acquired hourly for 30 s at between 30 frames s^-1^ (*R. balthica*) and 48 frames s^-1^ (*P. acuta* and *L. stagnalis*) for the duration of embryonic development, using the open source ImageJ plugin µManager (Edelstein et al., 2010). A resolution of 512 × 512 pixels was used for embryos of *P. acuta* and *L. stagnalis*, and 1,048 × 1,048 pixels for embryos of *R. balthica*.

### 2.4 Image analysis

Image sequences of developing embryos were analysed using an open source Python package Embryo Computer Vision (EmbryoCV) (16). Energy proxy traits (EPTs) were calculated for each 30 s video timepoint, for each embryo, using the method of Tills *et al.* (2021). In brief, mean-pixel values of the region containing the embryo in each frame were extracted as a time series. Signal decomposition using Welch’s method (1967) was then used to decompose mean pixel value signals into the temporal domain, thereby providing a spectra of energy values at different temporal frequencies.

**FIGURE 1:**
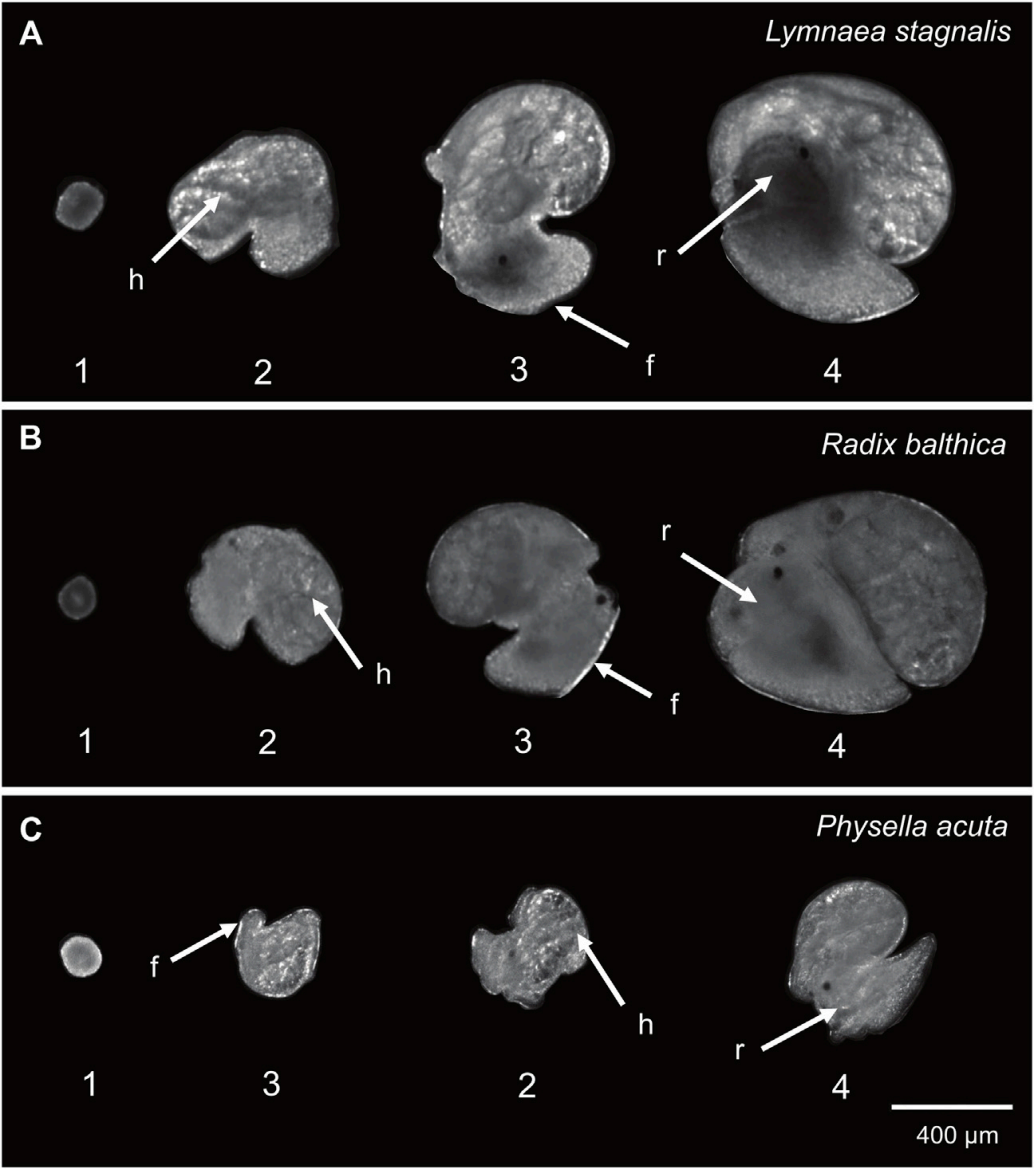
Developmental events used in this study (1 = onset of ciliary driven rotation, 2 = onset of cardiac function, 3 = foot attachment and onset of muscular crawling, 4 = onset of radula function). **(A)** *Lymnaea stagnalis* **(B)** *Radix balthica* **(C)** *Physella acuta*. Locations of developmental events recorded are indicated: h = cardiac function, f = foot attachment, r = radula function. In *Physella acuta,* attachment of the foot on the wall of the egg capsule occurs before the onset of cardiac function.

For each embryo, the timings of onset of a number of key developmental events were quantified from video *via* manual analysis for each species. These were i) the onset of ciliary driven rotation, ii) the onset of cardiac function (determined by the first visible heart beat), iii) attachment to the wall of the egg capsule and transition to muscular crawling (the point at which there is a clear attachment of the foot, rather than the embryo simply ‘resting’ on the wall of the egg capsule), and iv) the onset of radula function, the last developmental stage in all three species used (Smirthwaite et al., 2007) (Table 1; Figure 1). Heterochronies in the timing of muscular crawling and cardiac function exist between *P. acuta, L. stagnalis* and *R. balthica*. *Physella acuta* exhibits a significantly earlier onset of attachment and crawling on the wall of the egg capsule, relative to the onset of cardiac function, and relative to the timing of this event in *L. stagnalis* and *R. balthica* (Smirthwaite et al., 2007).

**TABLE 1:** Descriptions of developmental events used in this study [after Smirthwaite et al. (2007)]. Each developmental event was recorded when it was first observed during observations of hourly timepoints recorded for each embryo.

Developmental event	Description
Rotation	Onset of ciliary driven rotation of the embryo
Attachment and crawling	Attachment of the foot to the wall of the egg capsule, and onset of muscular crawling
Cardiac function	First observable heartbeat
Radula	Onset of radula function, located in the head

To visualise developmental differences in EPTs between species, the sum of energy levels across all frequencies of the EPT spectra (hereafter referred to as total energy) were calculated, normalised within each individual (0–1), and expressed as a time series. The duration of embryonic development (i.e., the time taken to hatch) varies between individuals and species, therefore to generate a standardised developmental rate that would allow for comparison between species and individuals, and be invariant to differences in overall rates of development, the absolute timings from the 4-cell stage to hatching were converted to relative time (0–1).

### 2.5 Dimensionality reduction

EPT spectra are high-dimensional representations of observable movement, and therefore to compare interspecific, and development stage specific differences in combinatorial signals across frequency spectra, dimensionality reduction in the form of principal component analysis (PCA) was used. Temporal frequency data were binned to 0.1 Hz increments (0.03—6.0 Hz, 60 frequency bins). Frequency data were restricted to 6.0 Hz as the upper limit at which biologically meaningful signals would be expected. Mean energy within each frequency bin for each normalised time point (0–1) was calculated and log transformed (McCoy et al., 2023). PCA (prcomp, package ‘stats’, v4.0.3) was applied to these data and the resulting eigenvectors were used to determine whether the onset of developmental events were associated with changes in high dimensional phenotypic space.

### 2.6 Statistical analysis

All data were analysed in R v4.0.3 (R Core Team, 2020). To determine whether time series of total energy data were different between species, a repeated measures analysis of variance (ANOVA, *p* < 0.05) was applied to total energy data. Additionally, Bayesian structural time series (BSTS) model using the R package CausalImpact (Brodersen et al., 2014) was applied to determine whether the onset of developmental events were associated with changes in total energy time series. To determine whether the onset of developmental events was associated with frequency specific differences in EPTs, a Kruskal–Wallis test (*p* < 0.05) was applied to mean energy values within each of the 60 frequency bands of 5 time point values before and after the onset of each developmental event. Multiple testing correction was applied using the Bonferroni method.

## 3 Results

### 3.1 Interspecific differences in developmental EPTs

**FIGURE 2:**
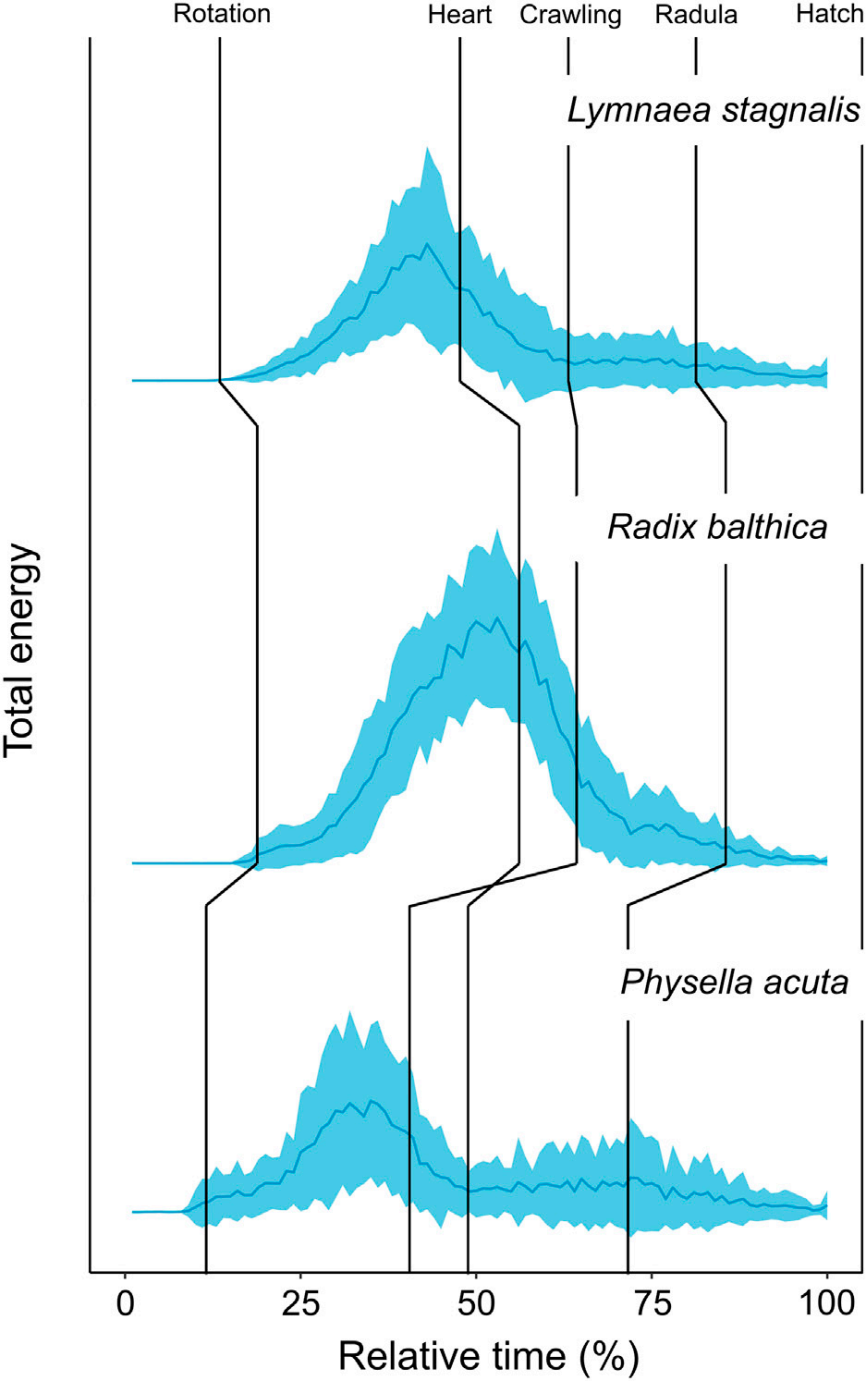
Developmental time series of normalised (0–1) total energy levels in embryos of *Lymnaea stagnalis* (N = 28), *Radix balthica* (N = 40) and *Physella acuta* (N = 43), across normalised developmental time (0%–100%) (mean ± sd). Median values of developmental event timings represented by vertical lines (Supplementary information 1). These include: i) rotation (the onset of ciliary driven rotation); ii) heart (the onset of cardiac function); iii) crawling (attachment to the wall of the egg capsule and the onset of ciliary driven rotation); iv) radula (the onset of radula function); v) hatch (emergence from the egg capsule).

Before we discuss the differences in EPT profiles between species, we will first describe the general trends in EPT profiles observed in these embryos. First, the timing of onset of rotation was associated with pronounced increases in total energy in the first quartile of development (Table 2) in each species, and reach a peak in total energy (Figure 2), followed by reductions in total energy prior to the onset of the heart and crawling approximately midway through development (Figure 2; Table 2). Total energy rapidly declines prior to the onset of crawling, before gradually reducing in the final quartile of development in all species (Figure 2).

**TABLE 2:** Effect of the onset of developmental events on time series of total energy through application of a Bayesian structural time series (BSTS) model using the R package CausalImpact (Brodersen et al., 2014).

Species	Event	Actual value	Predicted value	Absolute effect	Relative effect	Posterior tail-area probability (P)	Posterior probability of causal effect (%)
*Lymnaea stagnalis*	Rotation	26,631	107	26,523 (29,257, 26,527)	246.40 (246.08, 245.99)	**0.001**	99.90
	Heart	35,968	26,581	9,386 (24,814, −5,117)	−0.35 (0.93, −0.19)	0.120	88.00
	Crawling	11,201	37,678	−26477 (−14668, −38047)	−0.70 (−0.38, −1.01)	**0.001**	99.90
	Radula	6,480	11,538	−5,058 (−3,917, −6,262)	−0.43 (−0.34, −0.54)	**0.001**	99.90
*Radix balthica*	Rotation	30,577	94	30,483 (30,587, 30,378)	324.36 (325.37, 323.14)	**0.001**	99.90
	Heart	41,150	30,476	10,673 (37,004, −6,126)	0.35 (0.87, −0.20)	0.110	89.00
	Crawling	9,957	42,632	−32674 (−24544, −41431)	−0.77 (−0.58, −0.97)	**0.001**	99.89
	Radula	1704	10,389	−8,684 (−5,566, −11619)	−0.84 (−0.54, −1.12)	**0.001**	99.89
*Physella acuta*	Rotation	21,995	729	21,266 (21,489, 21,020)	29.15 (29.45, 28.81)	**0.001**	99.90
	Crawling	17,465	21,645	−4,179 (5,091, −14801)	−0.19 (0.27, −0.68)	0.200	80.00
	Heart	10,011	17,011	−7,000 (−2,763, −11586)	−0.41 (−0.16, −0.68)	**0.002**	99.79
	Radula	5,910	10,086	−7,000 (−3,442, −4,974)	−0.41 (−0.34, −0.49)	**0.001**	99.90

Bold values indicate statistically significant result.

Clear differences occurred in EPT profiles between species (repeated measures ANOVA, F_2, 199_ = 54.52, *p* < 0.0001, Figure 2). Additionally to this, the onset of functional developmental events were also associated with pronounced differences in EPTs within each species (Table 2). The reduction in total energy levels prior to the onset of crawling appeared to occur earlier in *Physella acuta* relative to *Lymnaea stagnalis* and *Radix balthica*. Additionally, this period of total energy decline preceding the onset of crawling was compressed in *P. acuta* relative to *L. stagnalis* and *R. balthica*. In *L. stagnalis* and *R. balthica*, embryos remain free swimming during the development of the heart and other functions. Before crawling, embryos will intermittently rest with increasing frequency on the wall of the egg capsule, which is evident *via* the gradual reduction in total energy levels before crawling. However, in *P. acuta* this free swimming stage is absent which likely explains the comparatively compressed period of total energy decline prior to crawling. Finally, the generally later onset of each developmental event in *R. balthica* was associated with a shift of the entire time series later into relative developmental time (Figure 2).

### 3.2 Dimensionality reduction for EPT spectra differentiation

To determine whether the onset of developmental events was associated with changes in EPTs, PCA was applied to multivariate EPT time series. Data collected from individuals for each species were first averaged (mean) by normalised time point (0–1). Following this, levels of energy within each frequency band were normalised (0–1) across relative developmental time.

**FIGURE 3:**
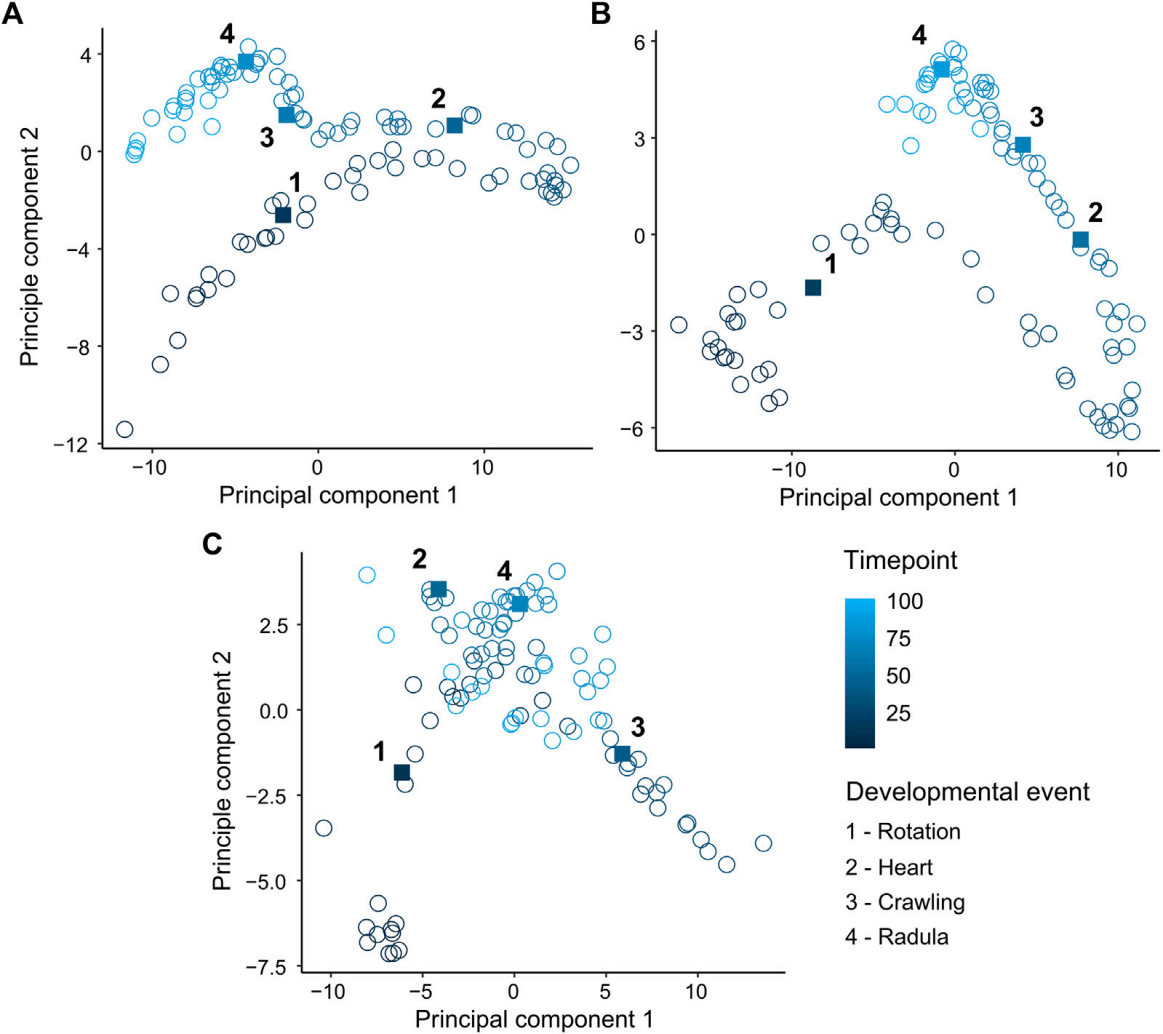
Principal component analysis (PCA) of mean levels of energy within 60 temporal frequency bins across normalised time points (0–1). **(A)** *Lymnaea stagnalis* **(B)** *Radix balthica* **(C)** *Physella acuta*. Square filled points and adjacent numbers indicate mean time point of onset of developmental events: 1 = Rotation (onset of ciliary driven rotation); 2 = Heart (onset cardiac function); 3 = Crawling (attachment to the egg capsule and onset of muscular crawling); 4 = Radula (onset of radula function).

Principle components 1 and 2 incorporated 97.2%, 95.2% and 93.5% of the variance in the EPT spectrum for *L. stagnalis* (PC1: 85.5%, PC2: 11.7%) (Figure 3A), *R. balthica* (PC1: 75.2%, PC2: 20%) (Figure 3B), and *P. acuta* (PC1: 69.5%, PC2: 24%) (Figure 3C) respectively. The onset of some developmental events were associated with distinct separation of points, notably the onset of muscular crawling and radula function in *L. stagnalis* and *R. balthica*. In *L. stagnalis* we observed clear separation of points along the axis of PC1 between the onset of cardiac function and muscular crawling. Variation along the axis of PC1 was predominantly driven by frequencies ranging from 1.7 to 3.7 Hz (Figure 3A). Additionally, frequencies up to 0.9 Hz were predominantly driving variation along the axis of PC2, suggesting that differences in embryos between the onset of crawling and radula function were driven predominantly by frequencies within this range (Figure 3A) (Supplementary Information S2).

### 3.3 High-dimensional phenotypic change associated with the onset of developmental events

**FIGURE 4:**
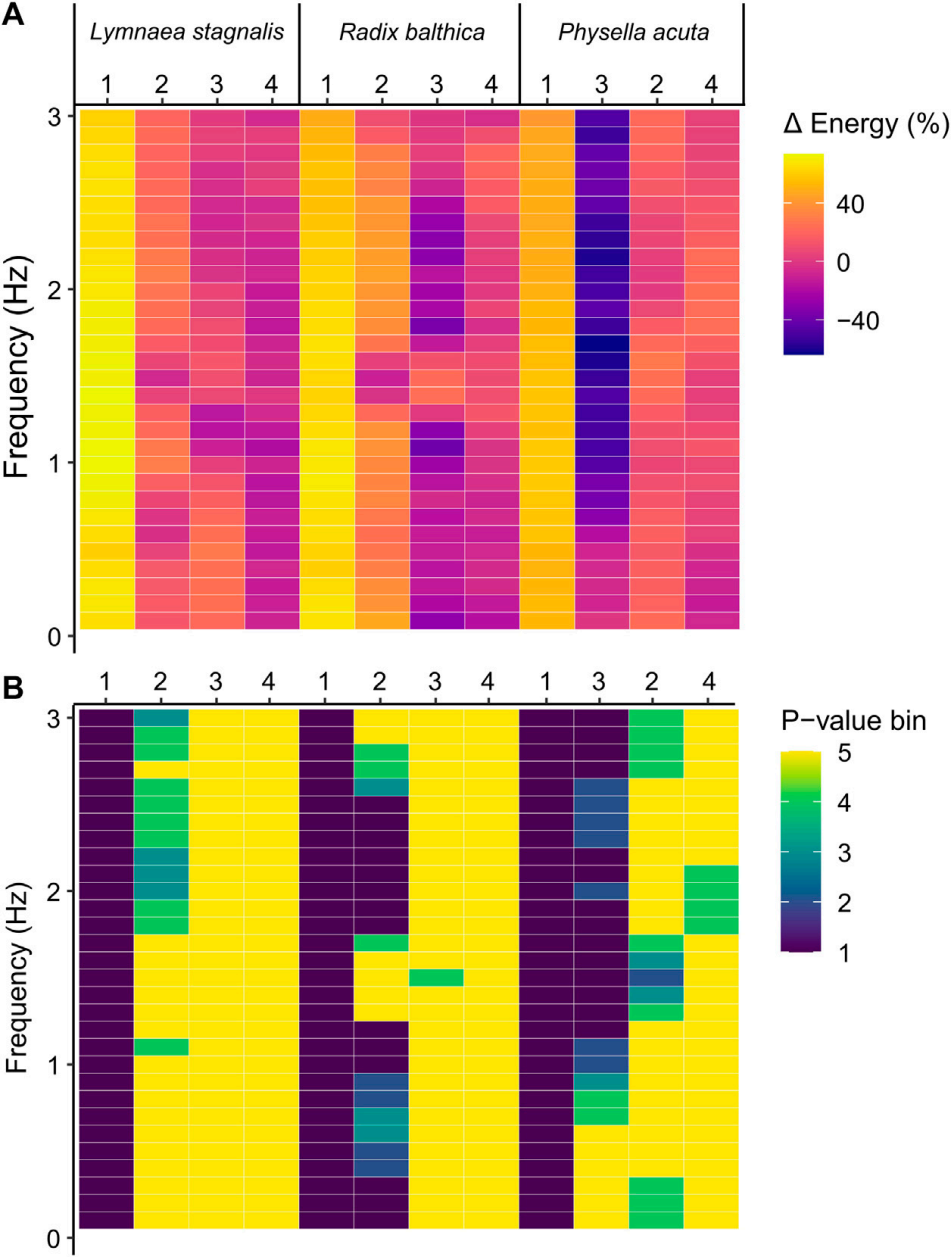
**(A)** Percentage change in energy values between each developmental event across frequencies in *Lymnaea stagnalis*, *Radix balthica* and *Physella acuta*. 1 = onset of ciliary driven rotation, 2 = onset of cardiac function, 3 = attachment to the wall of the egg capsule and onset of muscular crawling, 4 = onset of radula function. Colour indicates magnitude of change. **(B)** Results of Kruskal–Wallis test (Bonferroni adjusted, *p* = 0.00167) and statistical significance indicated by colour. *p*-value bins: 1 = 0–0.000001, 2 = 0.000001–0.00001, 3 = 0.00001–0.0001, 4 = 0.0001–0.00167, 5 = 0.00167–1 (not significant).

EPT are a spectra of energy values across different temporal frequencies. Therefore, to determine whether the onset of the developmental events used in this study were associated with composition differences of EPT spectra across temporal frequency bands, pairwise comparisons of energy before and after the onset of each developmental event were carried out for binned frequencies. In all species, the onset of ciliary driven rotation was associated with a significant increase in the levels of energy in all temporal frequency bands (Kruskal–Wallis, *p* < 0.0001, Figure 4; Supplementary Information S3). The onset of cardiac function was associated with a significant increase in energy up to 0.7 Hz and 1.6–1.8 Hz in *L. stagnalis*. In *R. balthica* and *P. acuta* the onset of cardiac function coincided with a significant increase in energy levels within the range of 1.2–1.6 Hz and 1.6–4.0 Hz respectively. (Kruskal–Wallis, *p* < 0.0001, Figure 4). The onset of muscular crawling on the wall of the egg capsule was associated with a significant reduction in energy within all temporal frequency bands for *P. acuta* (Kruskal–Wallis, *p* < 0.0001, Figure 4). Finally, following the onset of radula function, there were significant reductions in the levels of energy up to 0.5 Hz in *P. acuta* (Kruskal–Wallis, *p* < 0.0001, Figure 4).

## 4 Discussion

Our aim was to present an alternative way of describing and analysing heterochrony, by determining the extent to which evolutionary differences in the timings of developmental events (heterochronies) were associated with high-dimensional phenotypic change, using energy proxy traits (EPTs). We hypothesised that the onset of developmental events used in this study are associated with changes in EPT spectra, and that evolutionary differences in the timings of developmental events would be associated with changes in time series of EPT spectra between species. Causal impact analysis and PCA revealed that the onset of developmental events were associated with changes in EPT spectra. Furthermore, from our results we conclude that evolutionary differences in the timings of developmental events are associated with pronounced changes in high-dimensional phenotypic space. EPTs measure complex phenotypes as a spectrum of energy and application of these to the complete embryonic development of three closely related snail species *Lymnaea stagnalis*, *Radix balthica* and *Physella acuta* revealed interspecific differences in these spectra, tightly associated with previously described sequence heterochronies. Furthermore, investigation of EPT time series revealed functional changes before and after the onset of developmental events, but also striking differences between species for the same developmental events. Combinatorial analysis of EPTs using dimensionality reduction revealed distinct separation of points between developmental events, highlighting transitions in the functional phenotype of embryos in high-dimensional space. This suggests that developmental events, while a useful approach to comparative studies of development, may bely complex differences in functional phenotypes that could perhaps themselves be the object of natural selection.

### 4.1 Interspecific differences in EPTs coincide with pre-established sequence heterochronies

Time series of EPTs reflected interspecific differences in developmental event timings. Firstly, differences in the relative timing of onset of rotation was evident from time series of total energy between species. Ciliary driven rotation occurred earliest in *P. acuta* and this is evident as an earlier increase in total energy in relative developmental time. Following the onset of ciliary driven rotation, levels of total energy gradually increased in all species. Following this, embryos of all species showed clear reductions in total energy leading up to the transition to muscular crawling on the wall of the egg capsule, the timing of which reflected developmental differences between these species. In *P. acuta,* the onset of muscular crawling is brought forward significantly relative to the timing of cardiac function, and the onset of this event in *L. stagnalis* and *R. balthica* (Smirthwaite et al., 2007; Supplementary Information S1). From time series of EPT data, we can see a marked reduction in total energy occurring at approximately 37% of relative developmental time in *P. acuta* whereas this pronounced reduction in energy occurs at approximately 41% and 52% of relative developmental time in *L. stagnalis* and *R. balthica* respectively (Figure 2).

The observed changes in energy both before and after the timings of discrete developmental events used in this study showcase potential shortcomings in current approaches used to quantify the timings of development. Burggren (2021) observed that current research often focusses on development as a series of discrete events, when development should be seen as a continuum. Experimental protocols should gather data at multiple points of the developmental continuum, thereby allowing phenotypic measurements to be put into the context of the entirety of development. Here, we demonstrated considerable phenotypic change both before and after the onset of discrete developmental events, including the onset of ciliary driven rotation, and attachment to the wall of the egg capsule. The gradual increases and decreases in total energy likely reflect the continuous nature with which major multi-faceted developmental transitions take place in these embryos.

Firstly, the gradual increases in total energy with time co-occurs with increases in rates of rotation as development progresses (Figure 2). Without generalising to the other two species tested, previous research showed that embryos of *L. stagnalis* exhibited greater rates of rotation at the hippo stage (equivalent to approx. 40% developmental time) relative to the veliger stage (approx. 30% developmental time), which may contribute to explaining the gradual increases in total energy following the onset of rotation. Byrne *et al.* (2009) found that in embryos of *L. stagnalis* rates of rotation at the hippo stage were approximately 1.3–1.6 times greater than that of the veliger stage, and suggested that such differences in rotation rate between these two stages may be due to increases in anatomical complexity at the hippo stage.

Secondly, we also observed gradual decreases in total energy prior to attachment to the wall of the egg capsule and the onset of muscular crawling. Before this developmental event, embryos of *R. balthica* and *L. stagnalis* remain free swimming in the egg capsule (Meshcheryakov, 1990; Smirthwaite et al., 2007). During this period, embryos will increase the frequency at which they intermittently ‘rest’ their head on the wall of the egg capsule, before firmly attaching with the foot and commencing muscular crawling, which likely explains the gradual reductions in energy (and therefore to some extent overall rates of movement) proceeding the onset of this developmental event. When viewed as a discrete developmental event, the considerable phenotypic changes the developing embryo undergoes prior to its onset becomes masked, and arguable the event itself becomes increasingly arbitrary.

Additionally to this, there were features of both total energy time series and time series PCA that cannot be explained by the onset of, or changes in the timings of developmental events used in this study. For example, in each of the total energy time series, there is a distinct peak in total energy occurring at approximately 35%, 50% and 33% relative developmental time for *L. stagnalis, R. balthica* and *P. acuta* respectively (Figure 2). This peak occurred prior to the onset of cardiac function in *L. stagnalis* and *R. balthica* and muscular crawling in *P. acuta*, yet did not coincide with the onset of any of the developmental events used in this study. Embryos of *L. stagnalis* and *R. balthica* exhibit an intermittent resting behaviour prior to crawling. This peak represents the onset of this intermittent resting behaviour, prior to which rates of rotation during free swimming are greatest (Byrne et al., 2009). This indicates that the transitions to muscular crawling may better be regarded as a gradual reduction in rates of movement prior to attachment of the foot, rather than as a discrete event in developmental time, and highlights a potential limitation of using the timings of discrete developmental events for assessing evolutionary changes in early development.

Furthermore, measurement of this change in energy as a continuum also reveals evolutionary differences in developmental phenotype prior to the onset of attachment and muscular crawling, rather than just a difference in the timing as a discrete event in developmental time. The period of total energy decline in *P. acuta* prior to the onset of muscular crawling is compressed relative to *L. stagnalis* and *R. balthica* (Figure 2). In *P. acuta*, there is an absence of a free swimming stage, with embryos attaching to the egg capsule and commencing muscular crawling following the trochophore stage (Smirthwaite, 2007), thereby producing the relatively condensed period of total energy decline observed in the total energy time series (Figure 2). Consequently, by viewing development as a continuum of phenotypic change as we have done here, we were able to identify evolutionary differences in phenotype at the whole organism scale before, during and after the onset of discrete developmental events.

### 4.2 A high-dimensional phenotyping approach to physiological heterochrony

Development is an inherently complex phenomenon characterised by massive functional and spatial variation, variation that may act as a source of evolutionary change and innovation (West-Eberhard, 2003). There has been a historical emphasis on heterochrony as the main mechanism linking development to evolution (Gould, 1982; Smith, 2001; Smith, 2003). Whilst measurement of the timings of developmental events or stages in investigations of heterochrony facilitates direct interspecific comparison (Walls et al., 2019) quantification of the timings of developmental events which are in themselves discrete points in time, fails to capture any notion of development as a continuous and dynamic process. Furthermore, quantifying phenotypic change at the scale of the whole organism alongside the timings of discrete developmental events may provide a means of investigating the evolutionary significance of variation in developmental event timings, by linking the phenotype, which is the ultimate object of selection, to the timings of developmental events. Most biologists currently confine phenotyping efforts to a small number of observable traits, given the often overwhelming complexity of organismal biology and the challenge of quantifying it in a discrete and reproducible way. However, selection typically does not act on single traits, rather on multiple traits simultaneously (Lande and Arnold, 1983; Phillips and Arnold, 1999). When presented with high-dimensional datasets, dimensionality reduction allows for the visualisation of structure and cumulative drivers in high-dimensional phenotypic space. In the current study, we showed considerable separation of points associated with different points in relative developmental time from principal component analysis (PCA) (Figure 3). Additionally, PCA of time series data revealed distinct clustering of points following the onset of various developmental events, but also revealed considerable variation between these developmental events (Figure 3). Results from PCA of time series analysis further demonstrates how developmental events are only a snapshot in developmental time, and that focussing on these discrete events occludes phenotypic change during the periods of development surrounding them. Given what we know about how selection operates on multiple traits simultaneously, expanding the scale at which we capture phenotypic information during periods when phenotypic complexity is at its greatest may provide greater insight into the developmental mechanisms driving evolutionary change (Blows, 2007).

Despite this, application of high-dimensional phenotyping approaches are rarely extended to species and systems that may provide effective models for investigating the evolutionary significance of changes in the timings of development. In areas of research where high-dimensional phenotyping approaches are most developed, e.g., in plant and medical phenomics, there are already well-established and standardised phenotyping approaches applicable to the model species of interest (Houle et al., 2010; Furbank and Tester, 2011; Alexandrov et al., 2016; Tardeiu et al., 2017). A potential shortcoming in the field of comparative phenomics, and across evolutionary developmental biology more generally is an absence of phenotyping approaches that are readily transferable to non-model species of interest (Tills et al., 2018). One of the cornerstones of comparative developmental physiology is the selection of species that are best suited to answer a particular biological question (Krogh, 1929), yet approaches to high-dimensional organismal phenotyping remain constrained to model animals of interest, particularly the zebrafish *Danio rerio* (Pelkowski et al., 2011; Kalueff et al., 2016; Peravali et al., 2018)*,* nematode worm *Caenorhabditis elegans* (Yemini et al., 2013; Cornaglia et al., 2015; Olmedo et al., 2015), and the fruit fly *Drosophila melanogaster* (Chung et al., 2010; Levario et al., 2016). Here, EPTs were effective at characterising high-dimensional functional change in embryos of three species of freshwater gastropod, despite significant differences in their patterns of development. We suggest EPTs provide an effective and transferable phenotyping approach to quantifying phenotypic change in early life stages of a range of non-model species.

We suggest that evolutionary differences in the timings of developmental events are associated with differences in high-dimensional phenotypic space. As well as detecting interspecific differences in the timings of development between embryos of each species, we also found differences in the timings of EPTs between conspecifics. Previous research has shown that considerable standing variation exists in developmental event timings between conspecifics, which may provide the raw variation on which heterochronies are formed (Reilly et al., 1997; Mabee et al., 2000; Schmidt et al., 2004; de Jong et al., 2009; Kawajiri et al., 2009; Rundle et al., 2011; Tills et al., 2011; Rager et al., 2014). In addition to the observed interspecific differences in EPTs, individual level EPT data indicate considerable variation in EPTs between conspecifics. Comparison of developmental event timings alongside EPT data indicates that this variation corresponds with intraspecific differences in the timings of developmental events (Supplementary Informations S4-S6). The consequences of such variation are not currently understood but provide interesting lines of enquiry with which to investigate the performance and fitness implications of variation in the timings of developmental event between conspecifics. From previous research, EPTs appear to be related to some components of organismal fitness. Tills *et al.* (2021) showed that higher levels of total energy (reported in Figure 2) were associated with a faster growth rate in *R. balthica*, and posited that EPTs may in themselves be indicative of biochemical energy turnover, given that rates of biochemical energy turnover (metabolism) have been positively correlated with growth rates in a number of studies (Metcalfe et al., 1998; Nylin et al., 1998; Yamamoto et al., 1998) (although this is not always the case as such relationships are often context dependent, particularly between wild and laboratory reared specimens: Álvarez et al., 2005; Burton et al., 2011). Consequently, differences in the timings of developmental events may ultimately manifest as differences in biochemical energy turnover in developing embryos. Rates of energy expenditure are a significant object of selection (Bartheld et al., 2015), however, our understanding of the extent to which EPTs are linked with biochemical energetic turnover is currently poorly understood, and so is our understanding of how variation in EPTs ultimately influence components of organismal performance and fitness post-hatch. Further research is now needed to understand: i) the extent to which EPTs are related to biochemical energy turnover in developing embryos; and ii) the consequences of ontogenetic variation in EPTs for aspects of organismal performance and fitness.

## 5 Summary and conclusion

Evolutionary biologists have long sought to establish mechanistic links between development and evolution. Current research frames heterochrony, alterations in the timings of development as the main mechanism by which development leads to evolutionary change. However the current focus of heterochrony as the timings of discrete points in development may hinder quantification of phenotypic change associated with differences in the timings of these events. Here, through the application of a novel spectral phenotyping approach (EPTs), we captured a continuous functional time series of the embryonic development of three freshwater snails. Analysis of these time series provided evidence that evolutionary differences in the timings of development are associated with high-dimensional phenotypic change. Additionally to this, we provide preliminary evidence that intraspecific differences in EPTs coincide with differences in developmental event timings between conspecifics. We suggest that EPTs may provide an alternative approach to investigating the evolutionary significance of variation in the timings of development by allowing for the continuous quantification of phenotypic change at the scale of whole organism, associated with intraspecific and evolutionary differences in the timings of development. This study has the possibility of transforming the way we study heterochrony and development more generally.

## Data Availability

The original contributions presented in the study are included in the article/Supplementary Material, further inquiries can be directed to the corresponding author.

## Supplementary material

Supplementary Data sheet S1 Results of multivariate Kruskal-Wallis testing on the magnitude of change in energy across discrete frequency bands before and after the onset of key physiological events, in embryos of Lymnaea stagnalis, Radix balthica and Physella acuta.

Supplementary Data sheet S2 Relative and absolute timings of key physiological developmental events of embryos of Lymnaea stagnalis, Radix balthica and Physella acuta used in experimentation.

Supplementary Data sheet S3 Loadings data of PCA applied to frequency specific levels of energy across the development of embryos of Lymnaea stagnalis, Radix balthica and Physella acuta

Supplementary Data sheet S4 Total energy data across relative developmental time in embryos of Lymnaea stagnalis, Radix balthica and Physella acuta.

Supplementary Data sheet S5 Frequency specific levels of energy across relative developmental time in embryos of Lymnaea stagnalis, Radix balthica and Physella acuta.

Supplementary Figure S1 Individual plots of total energy across relative developmental time with overlaid physiological developmental events in embryos of Lymnaea stagnalis.

Supplementary Figure S2 Individual plots of total energy across relative developmental time with overlaid physiological developmental events in embryos of Radix balthica.

Supplementary Figure S3 Individual plots of total energy across relative developmental time with overlaid physiological developmental events in embryos of Physella acuta.
