# Supplementary figures and images for: Comparative phenomics: a new approach to study heterochrony
Source: Front Physiol. 2023 Nov 6;14:1237022. doi: 10.3389/fphys.2023.1237022 (PMC10658192; doi:10.3389/fphys.2023.1237022)

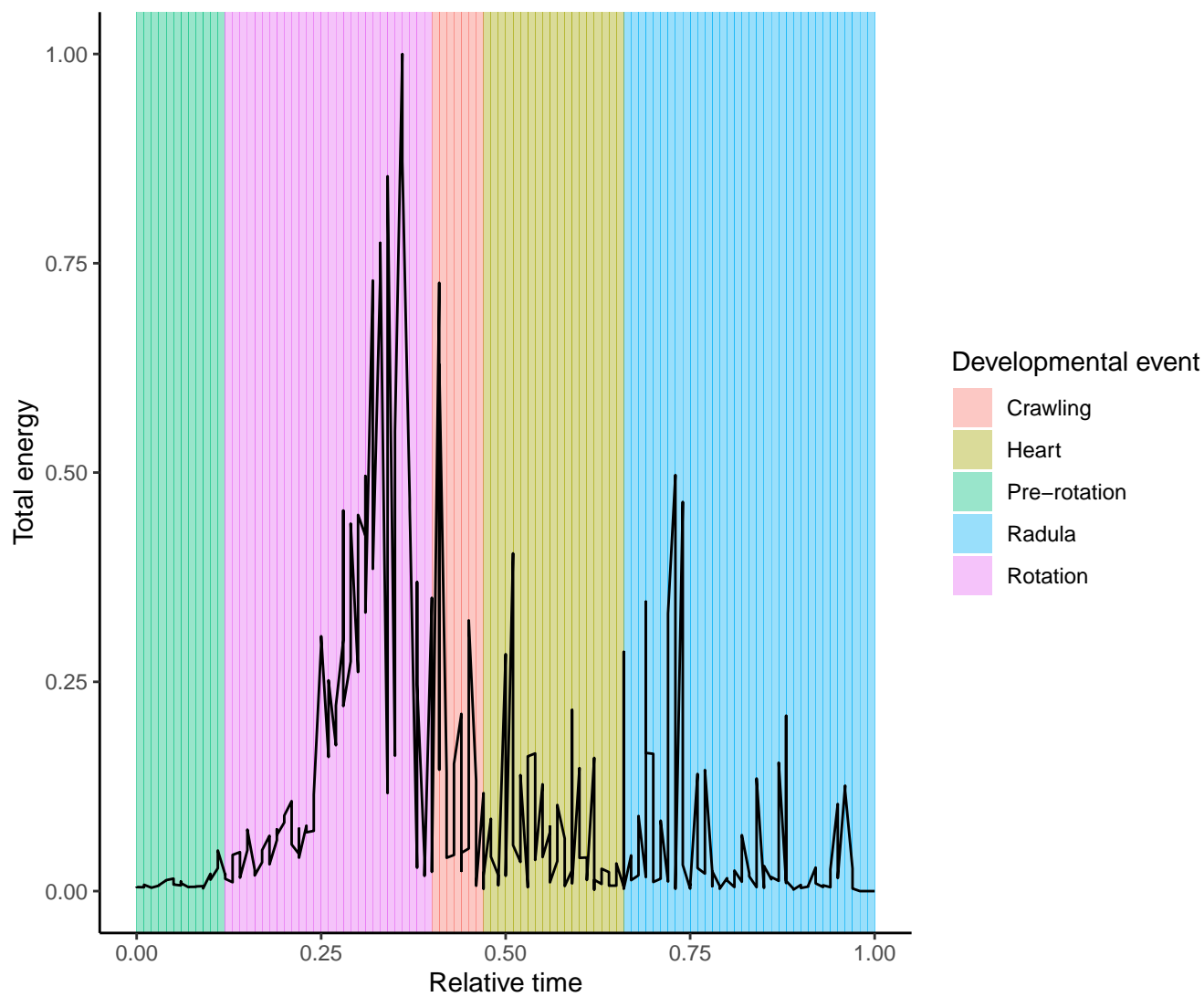

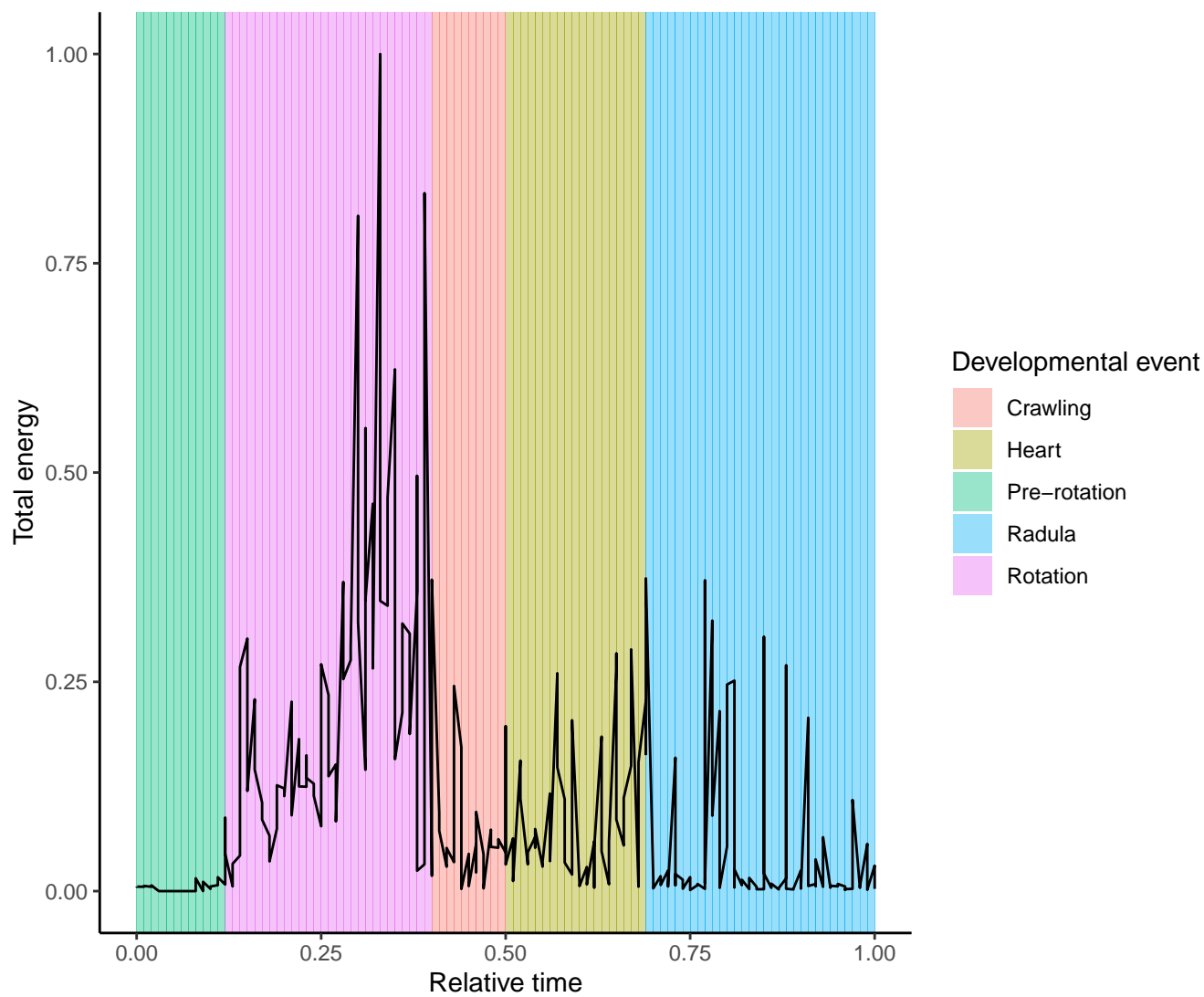

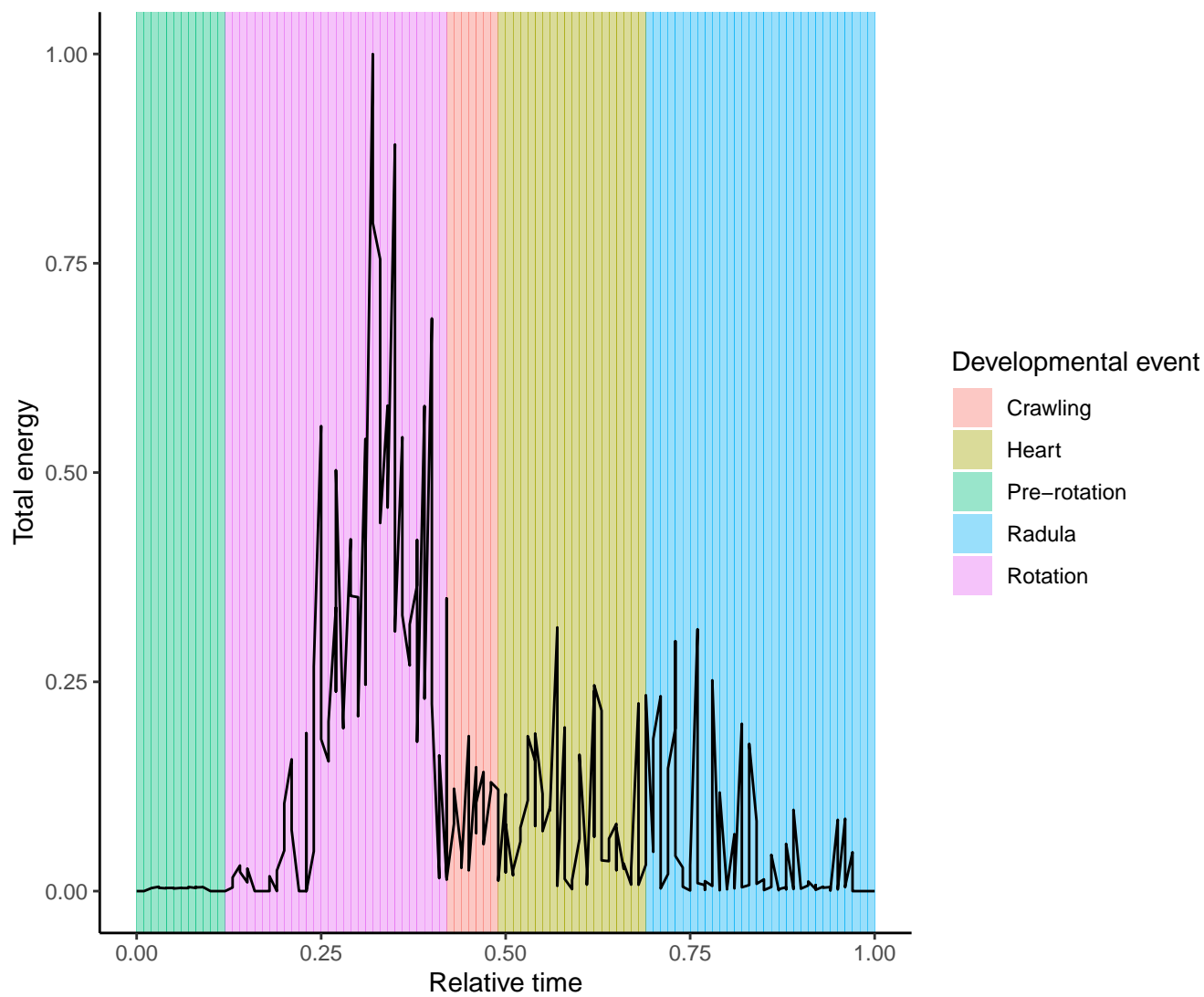

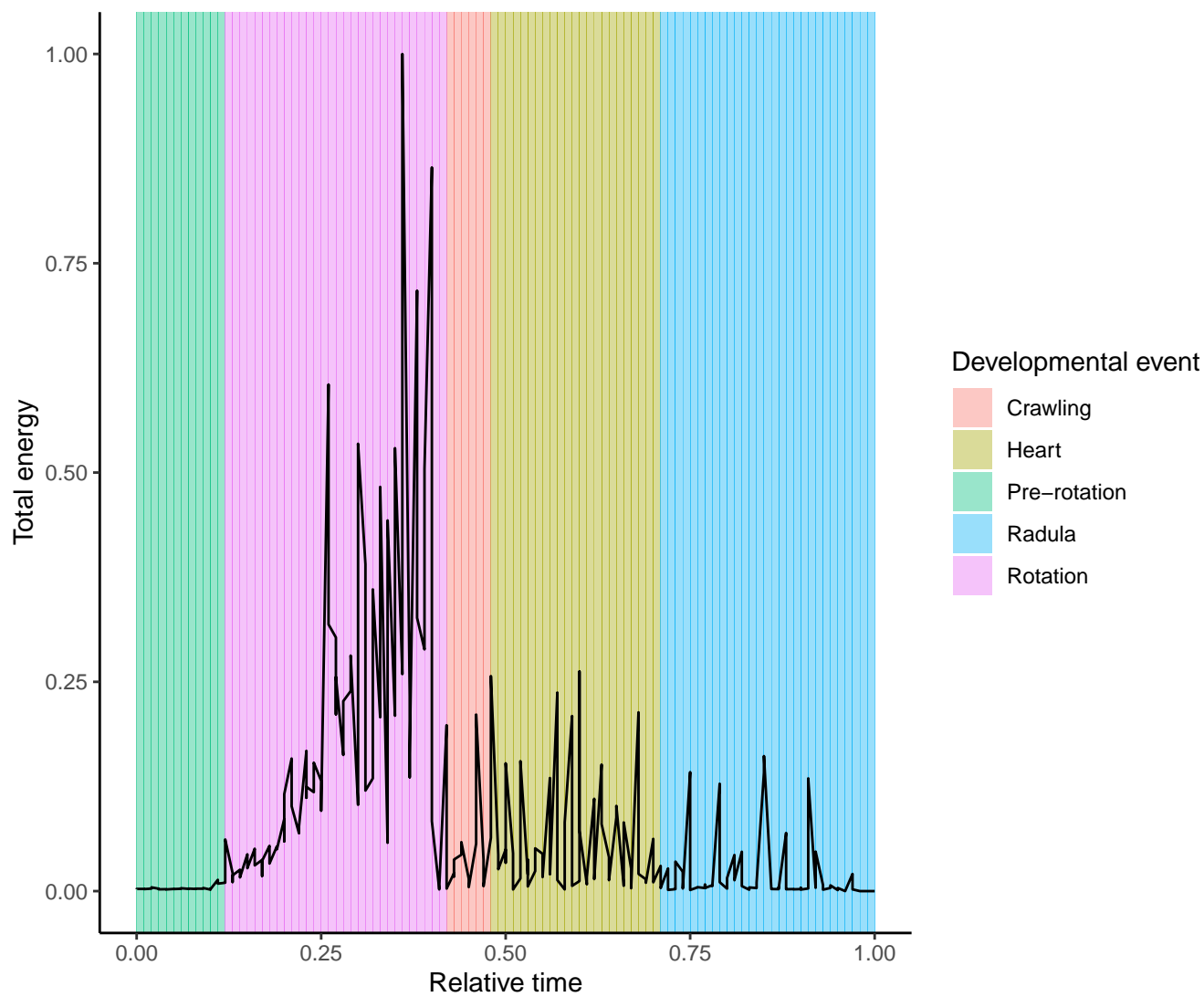

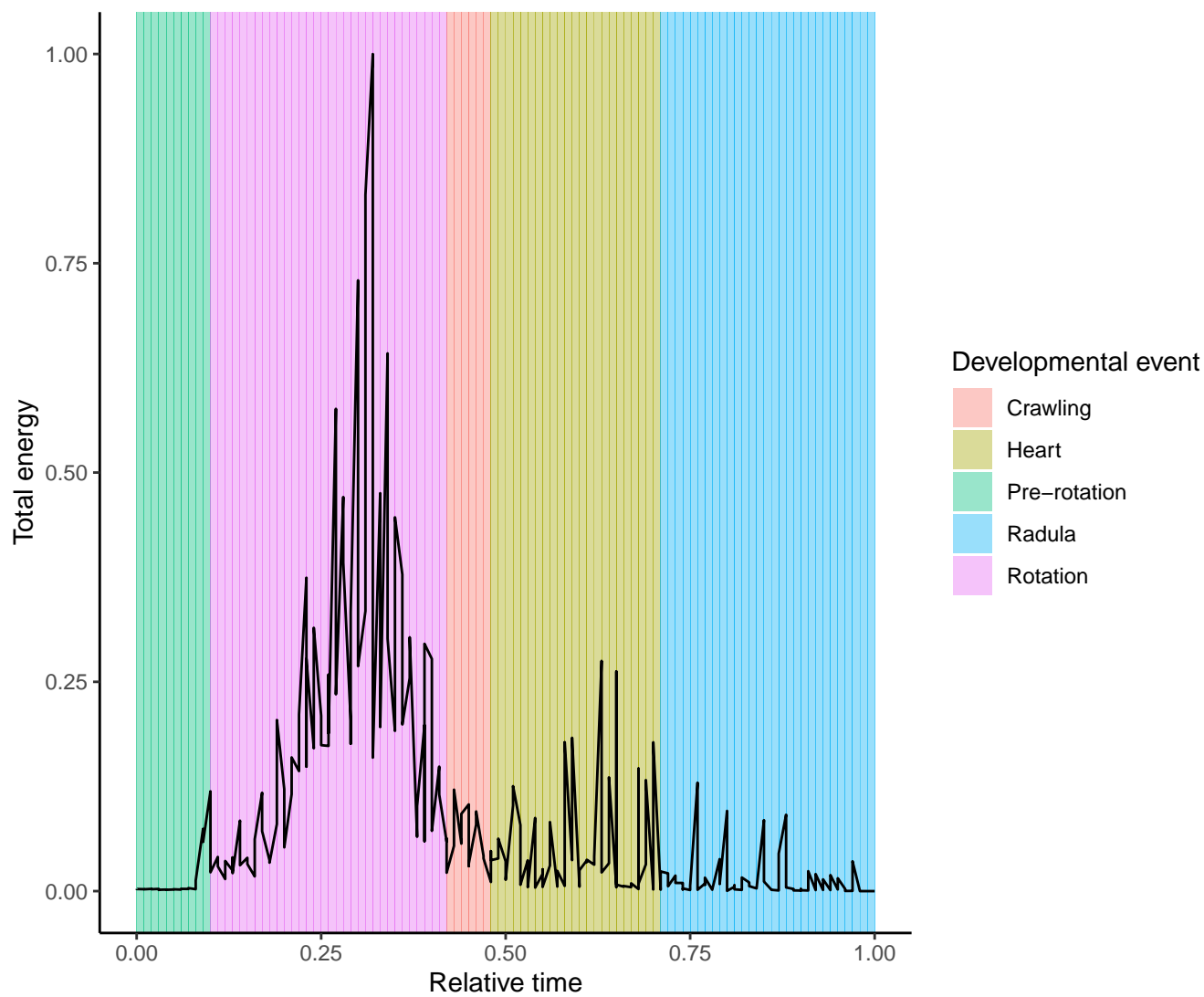

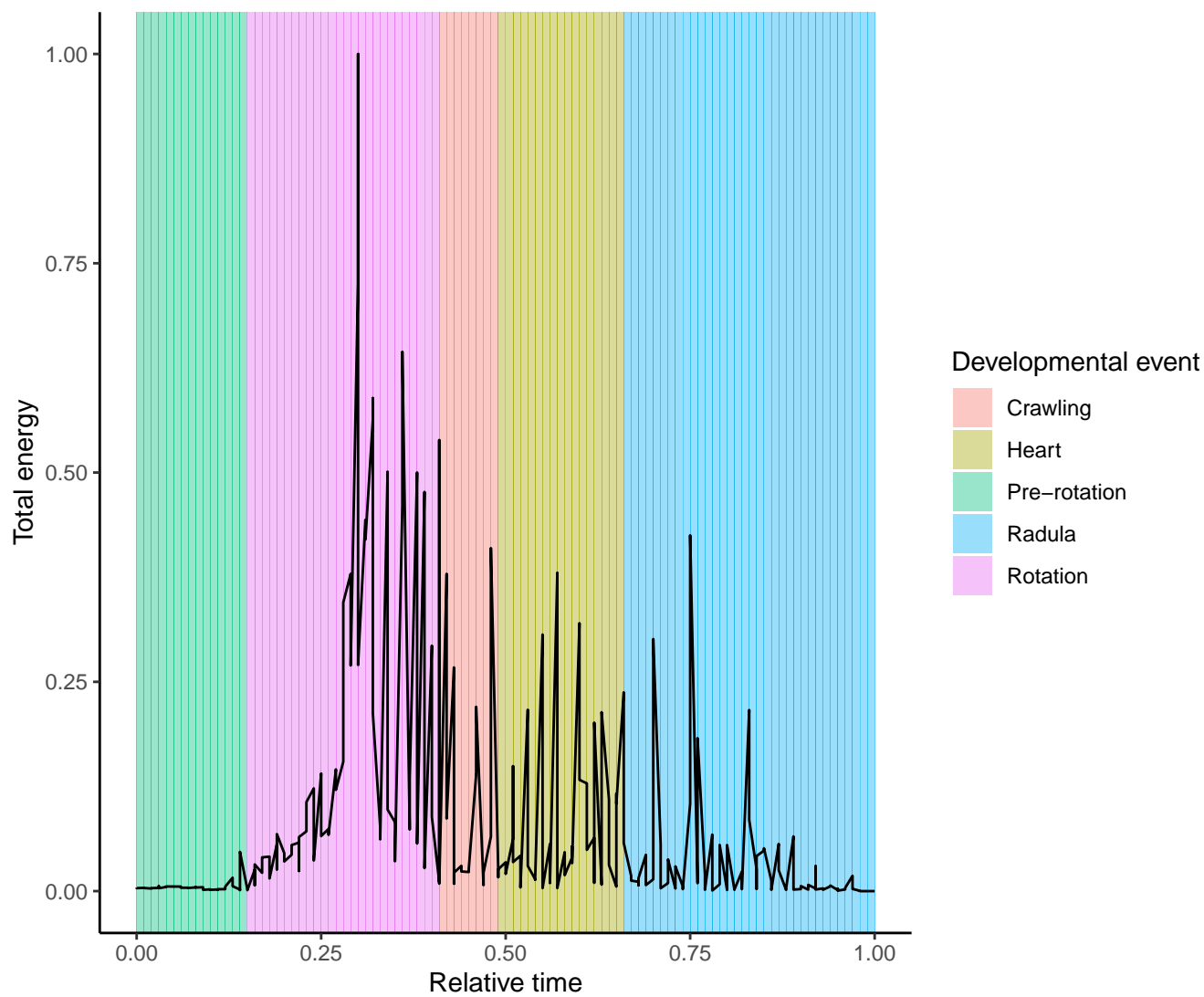

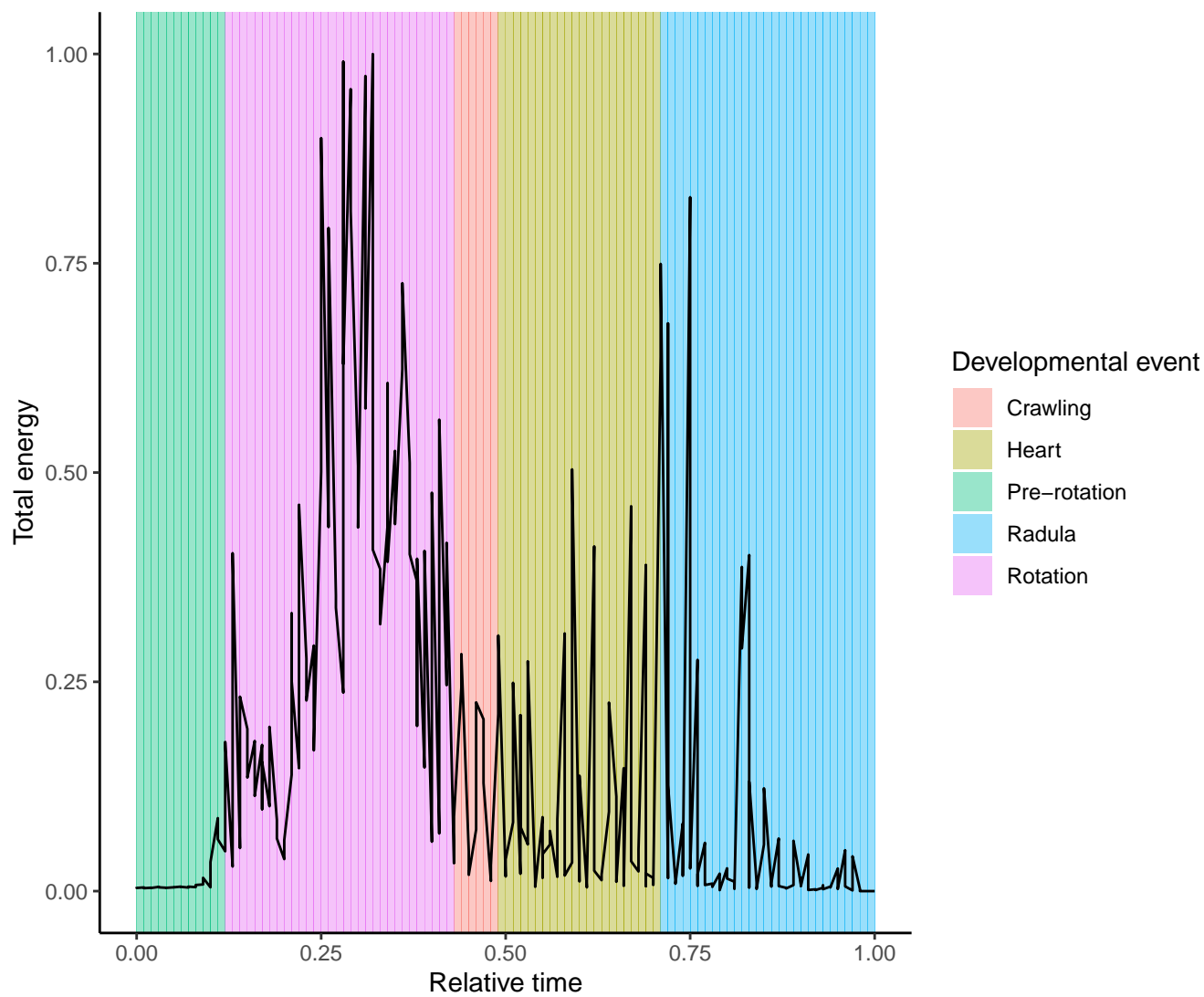

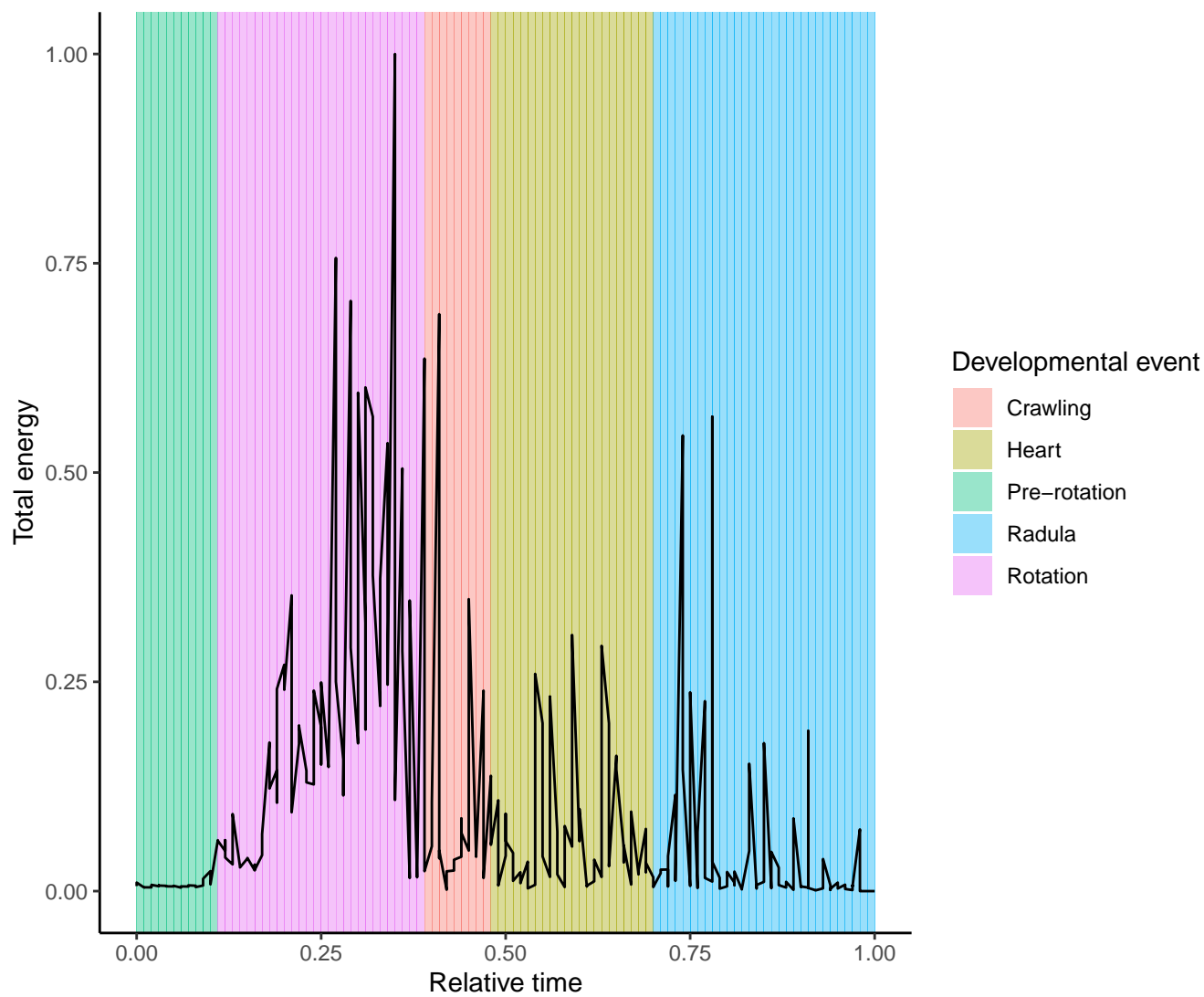

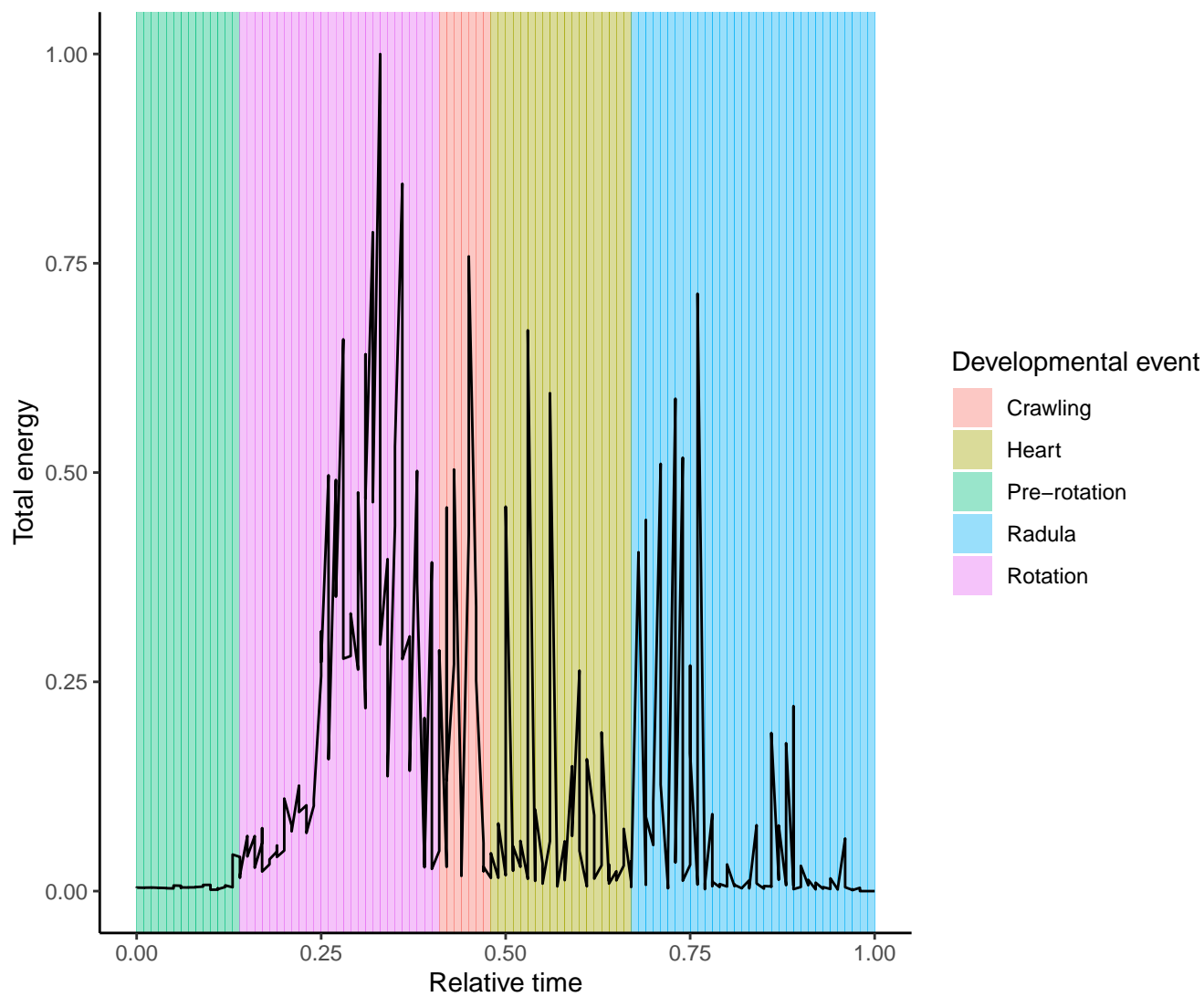

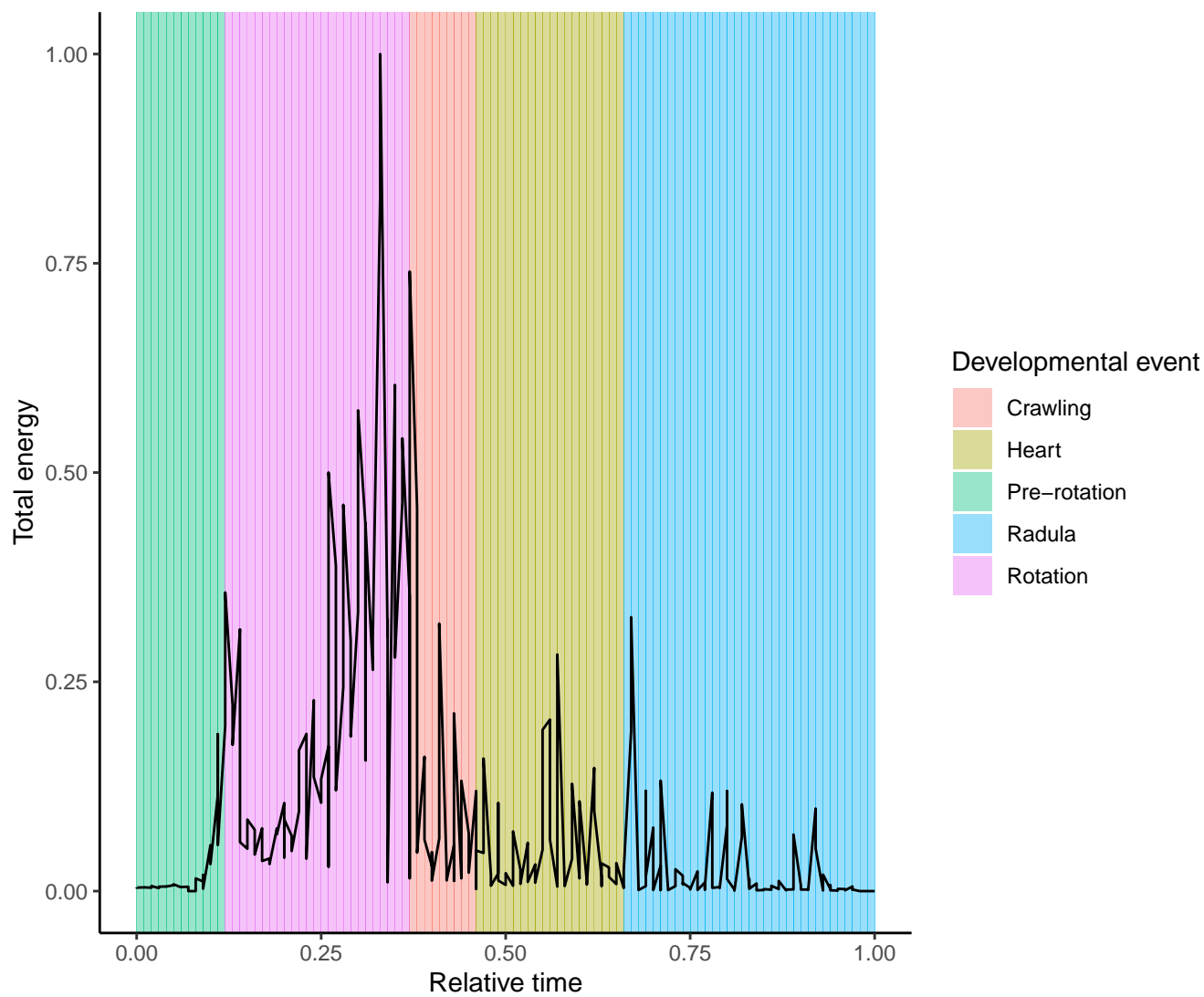

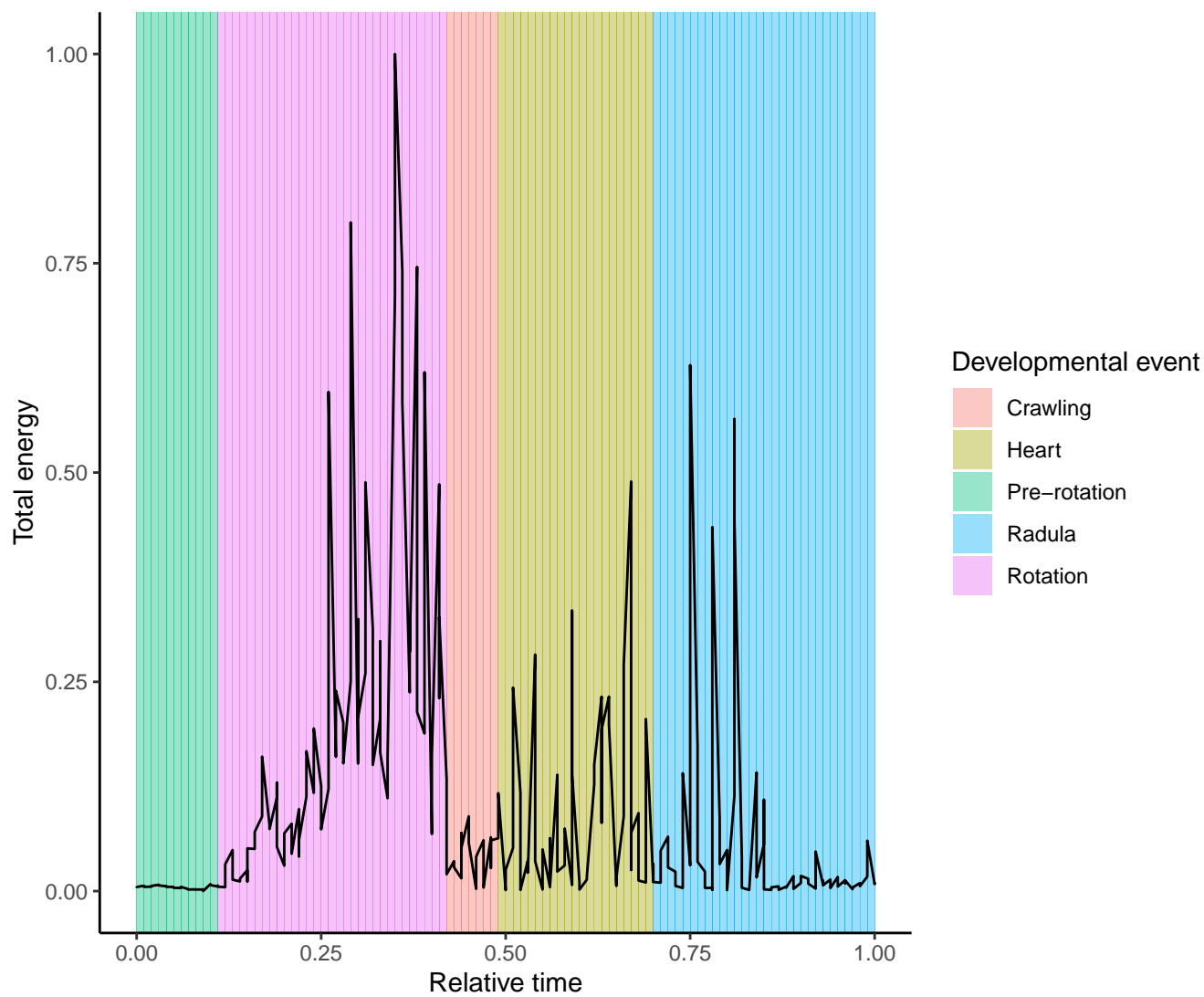

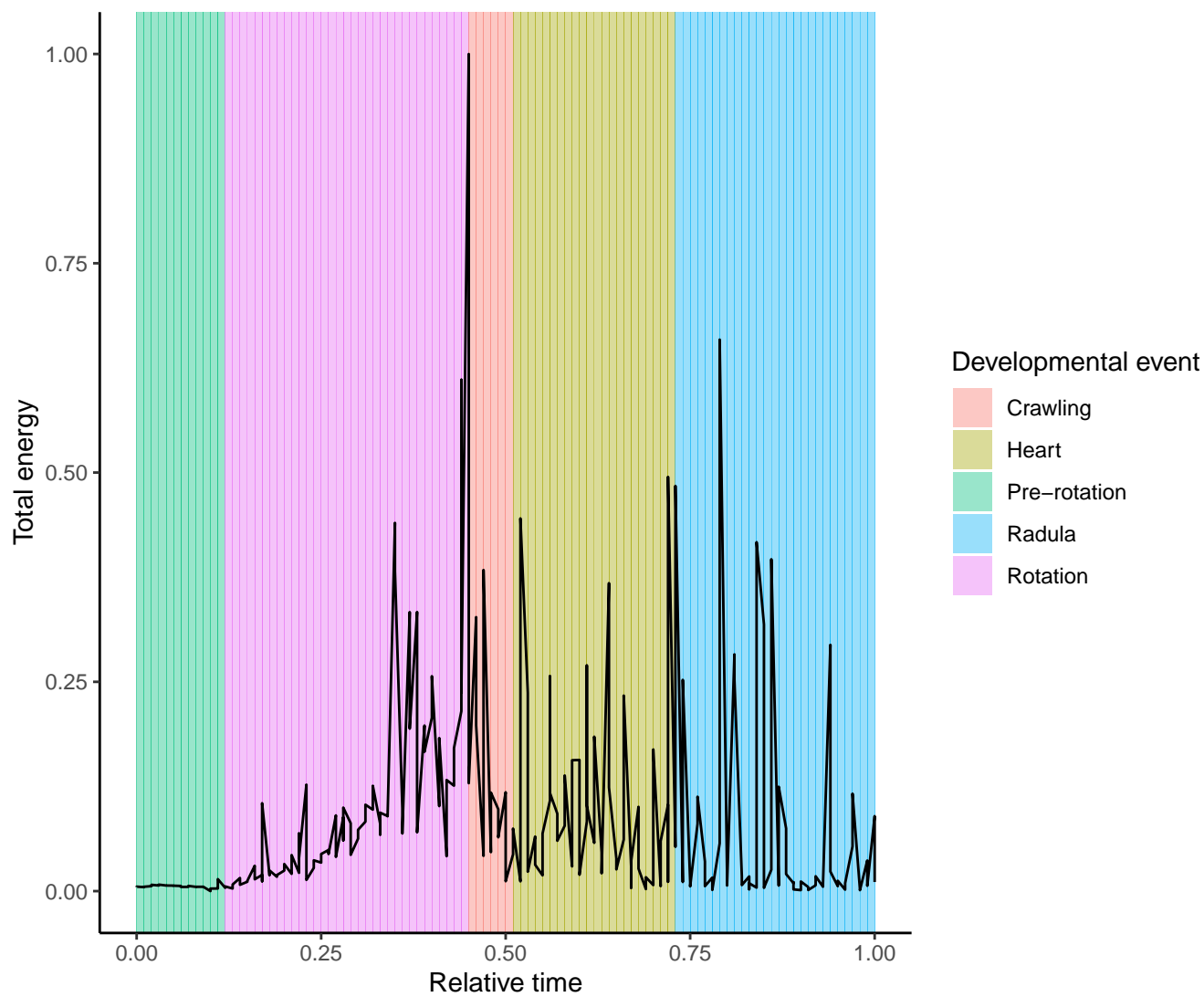

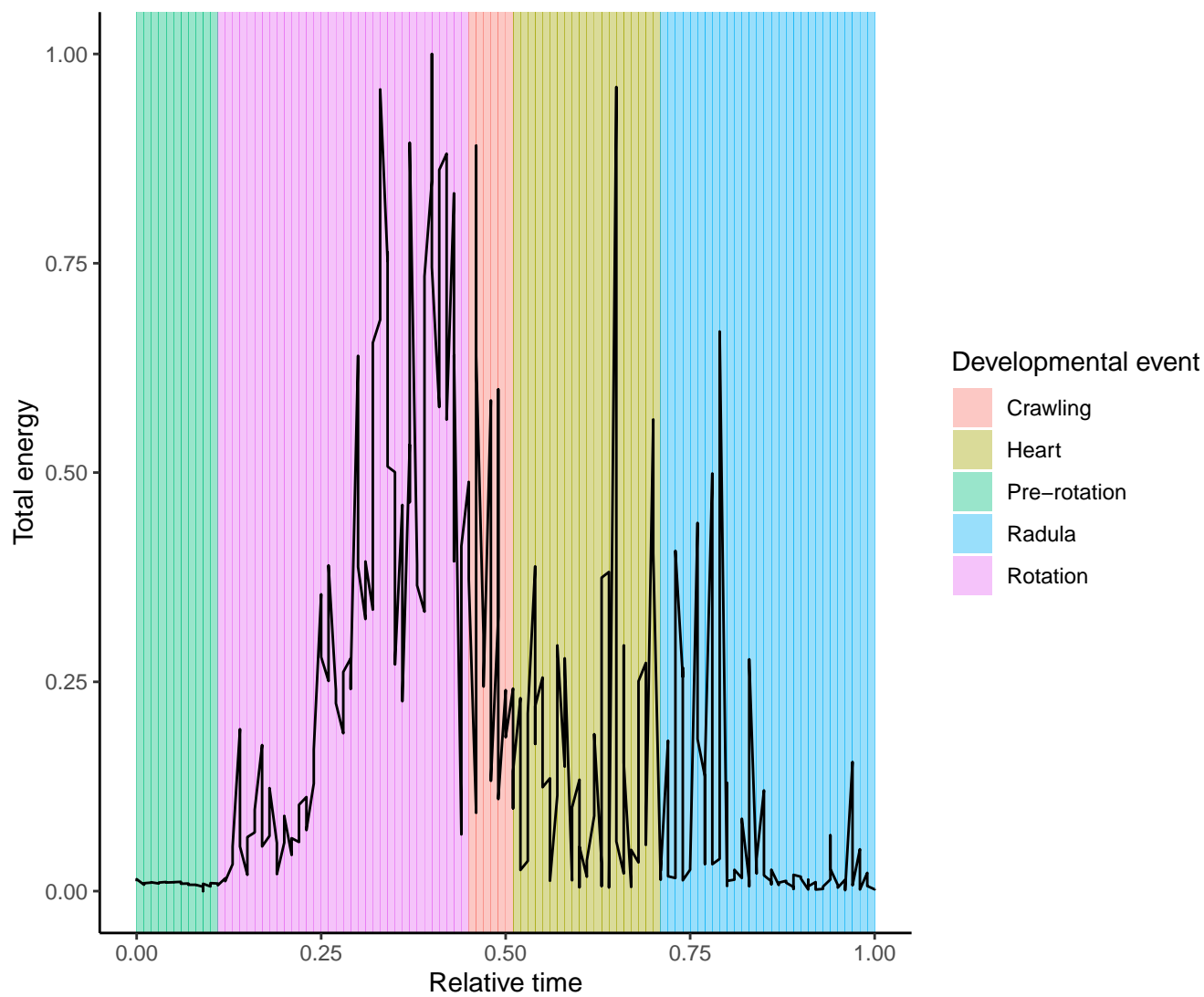

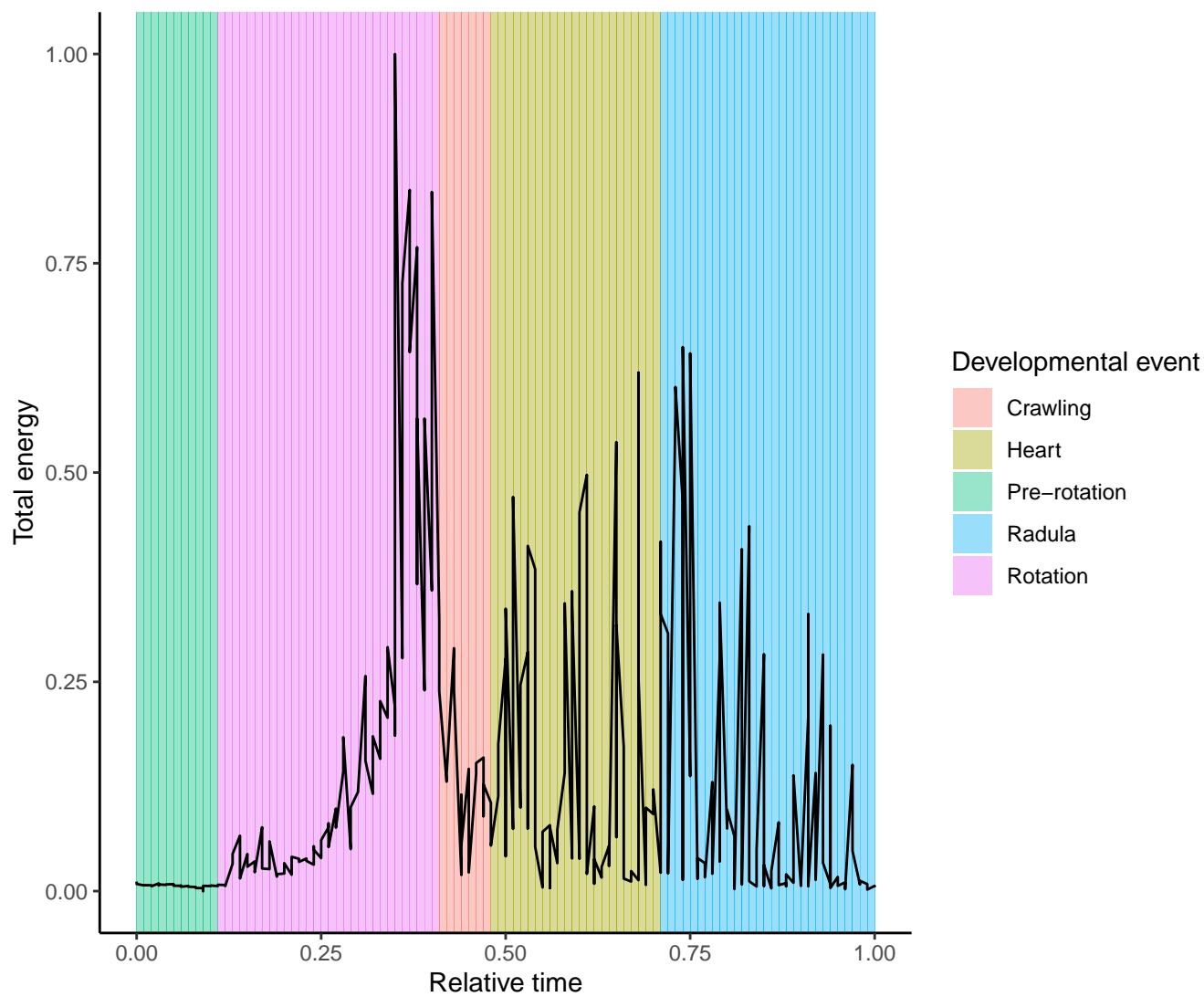

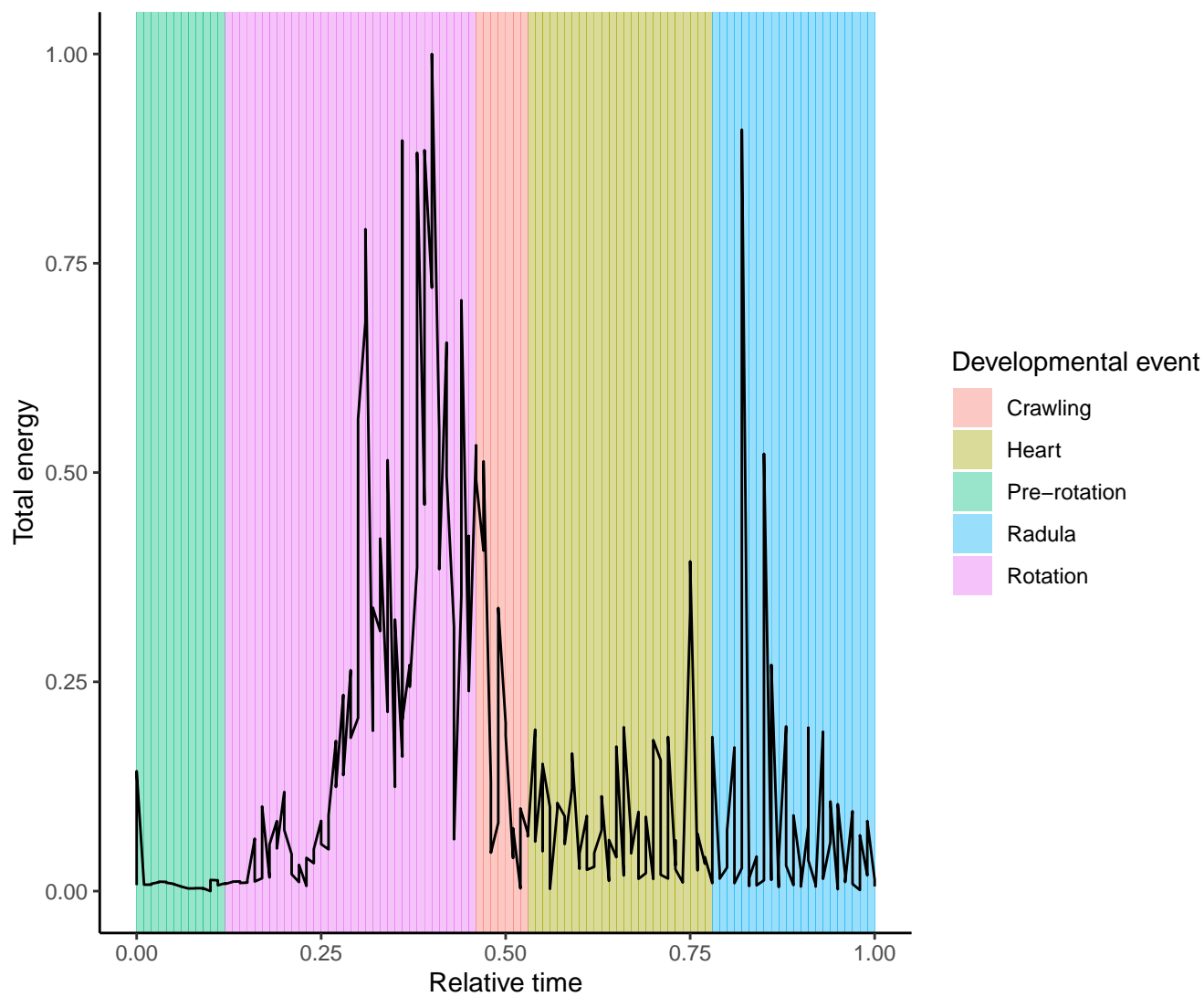

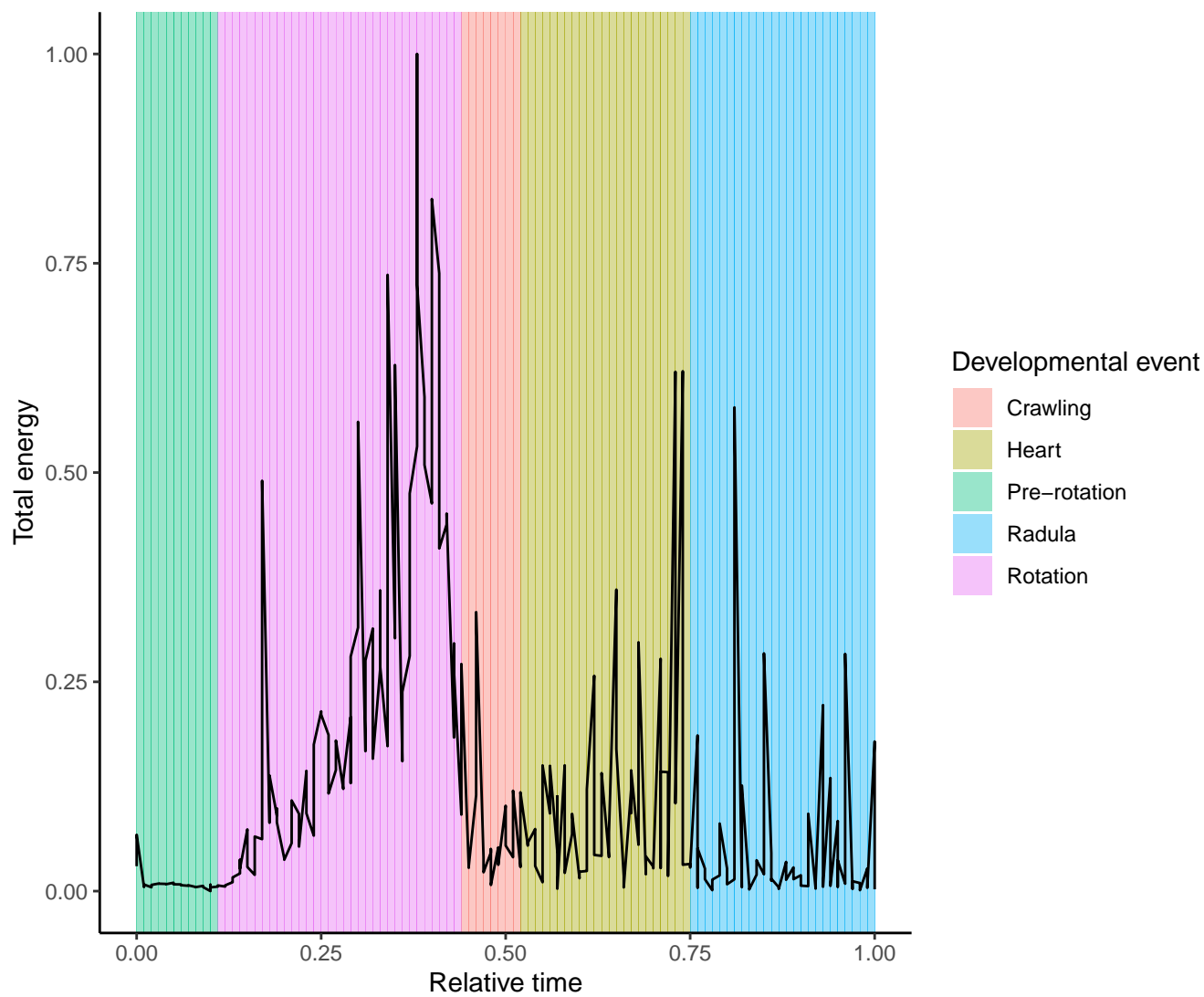

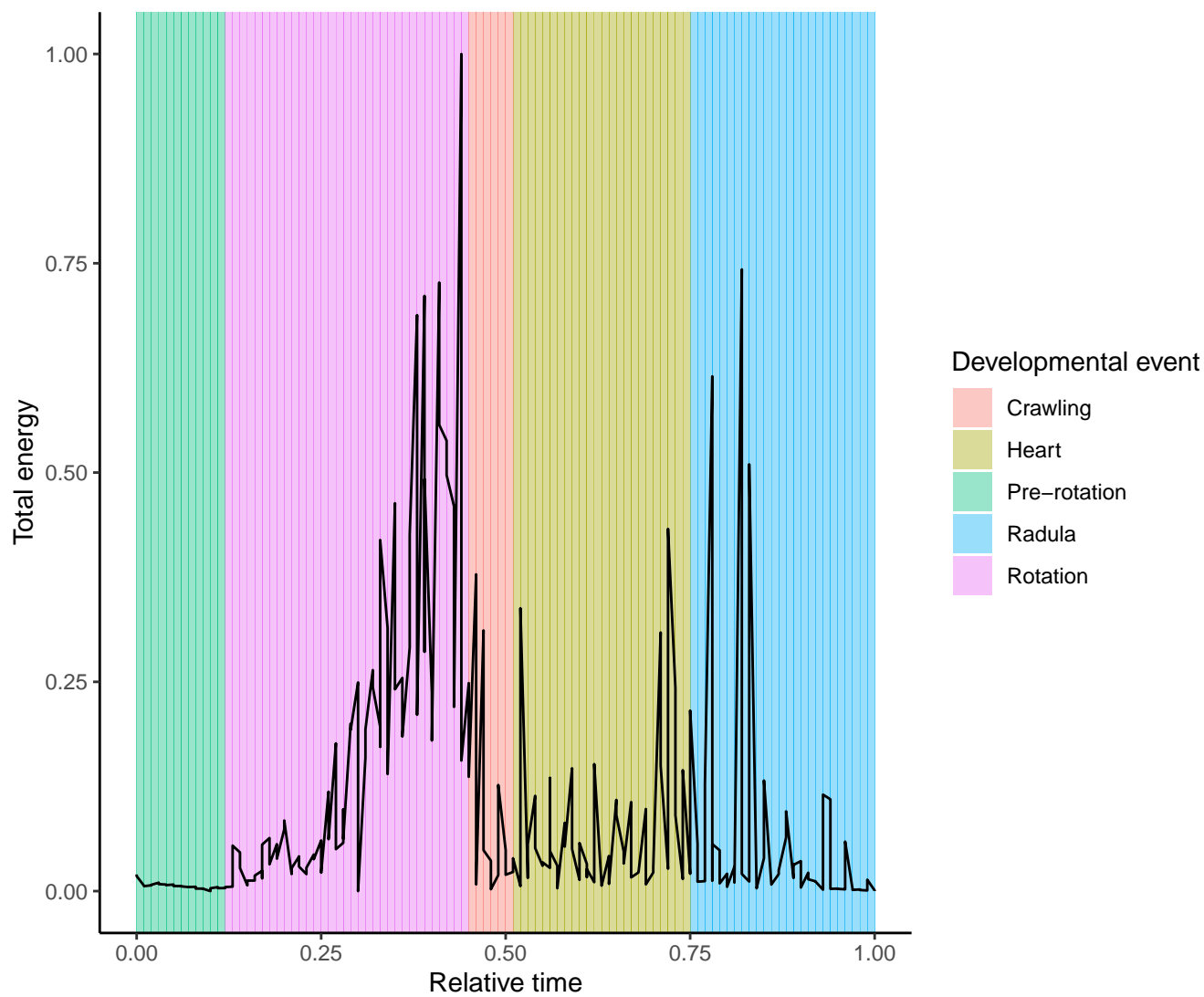

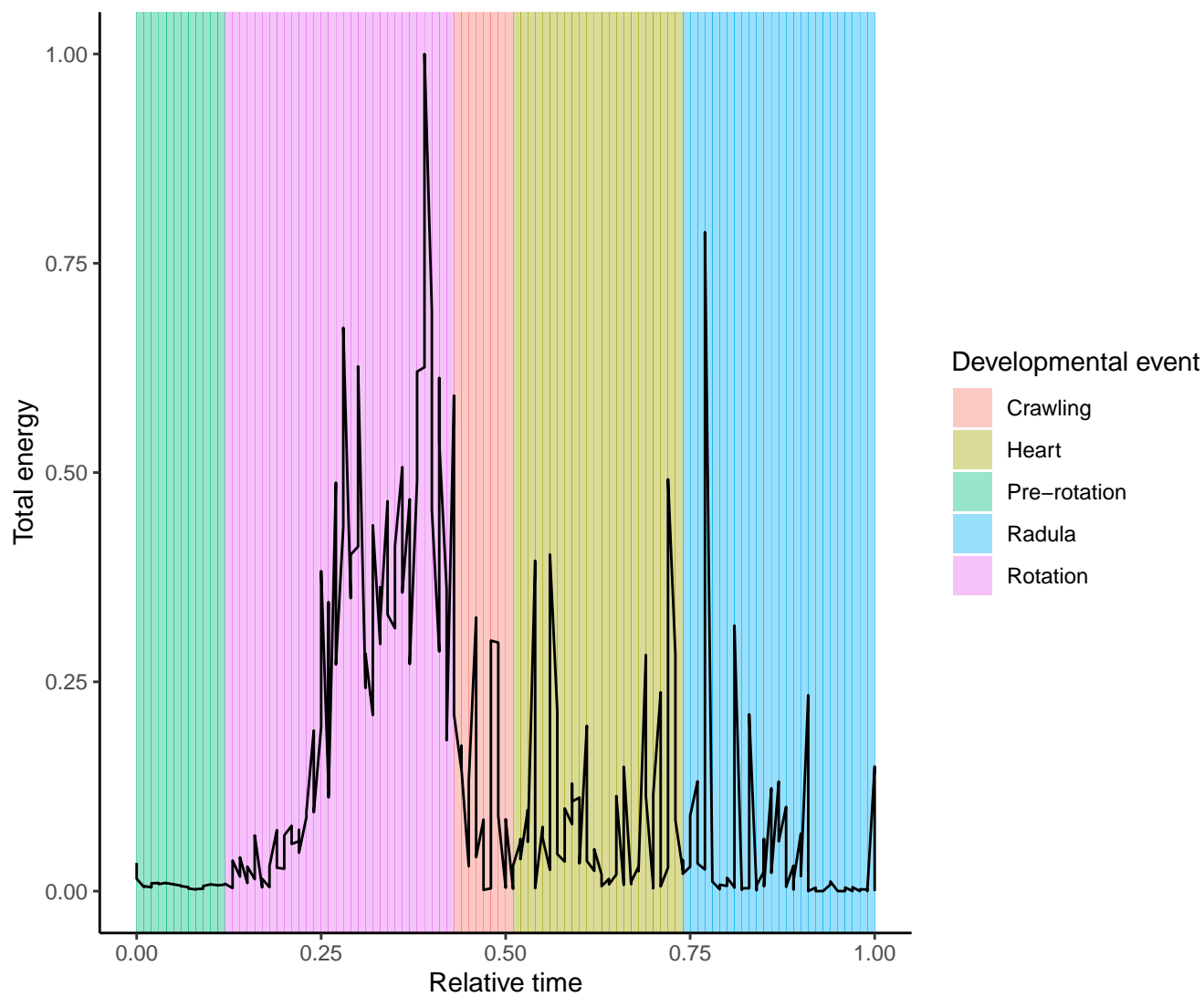

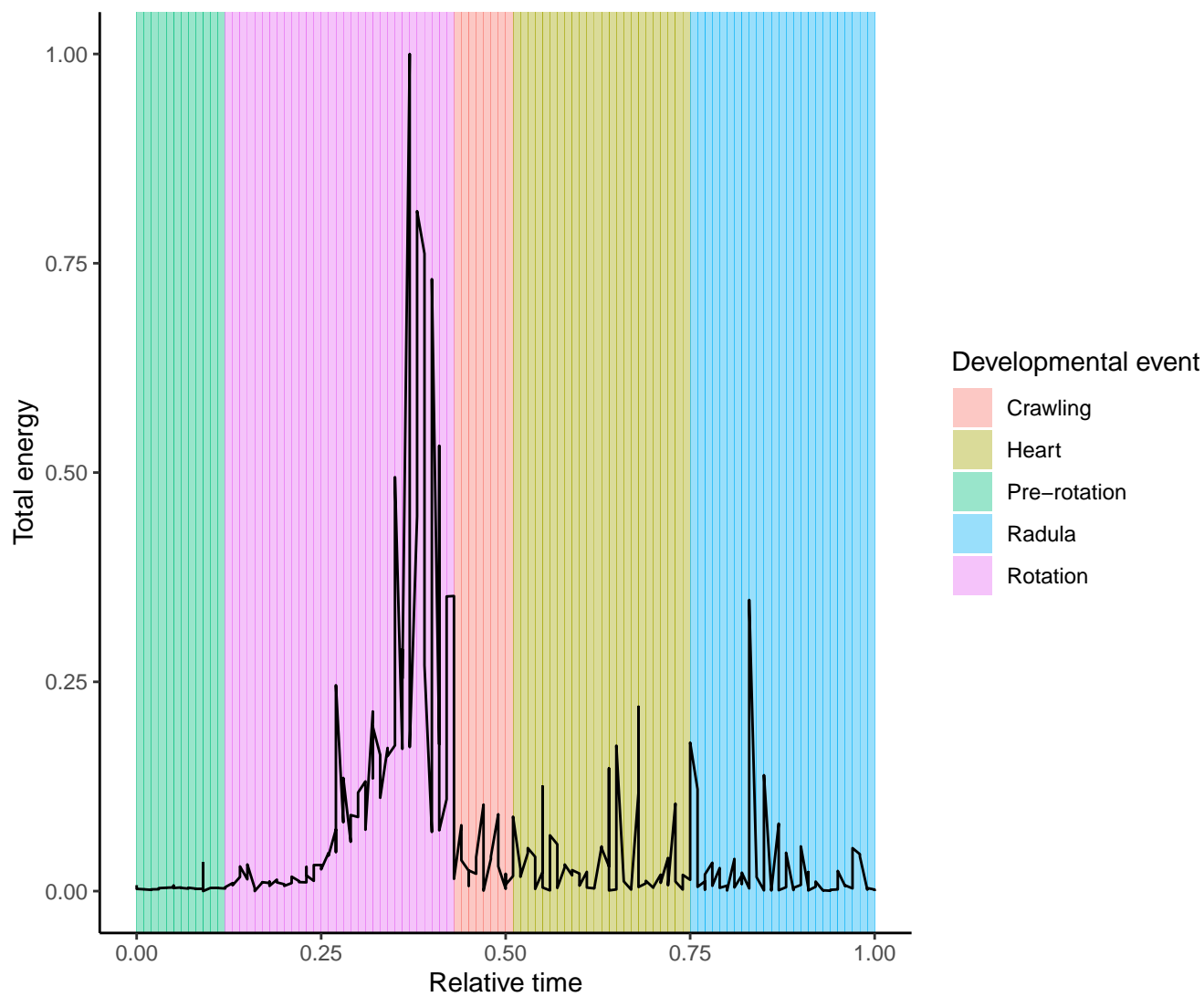

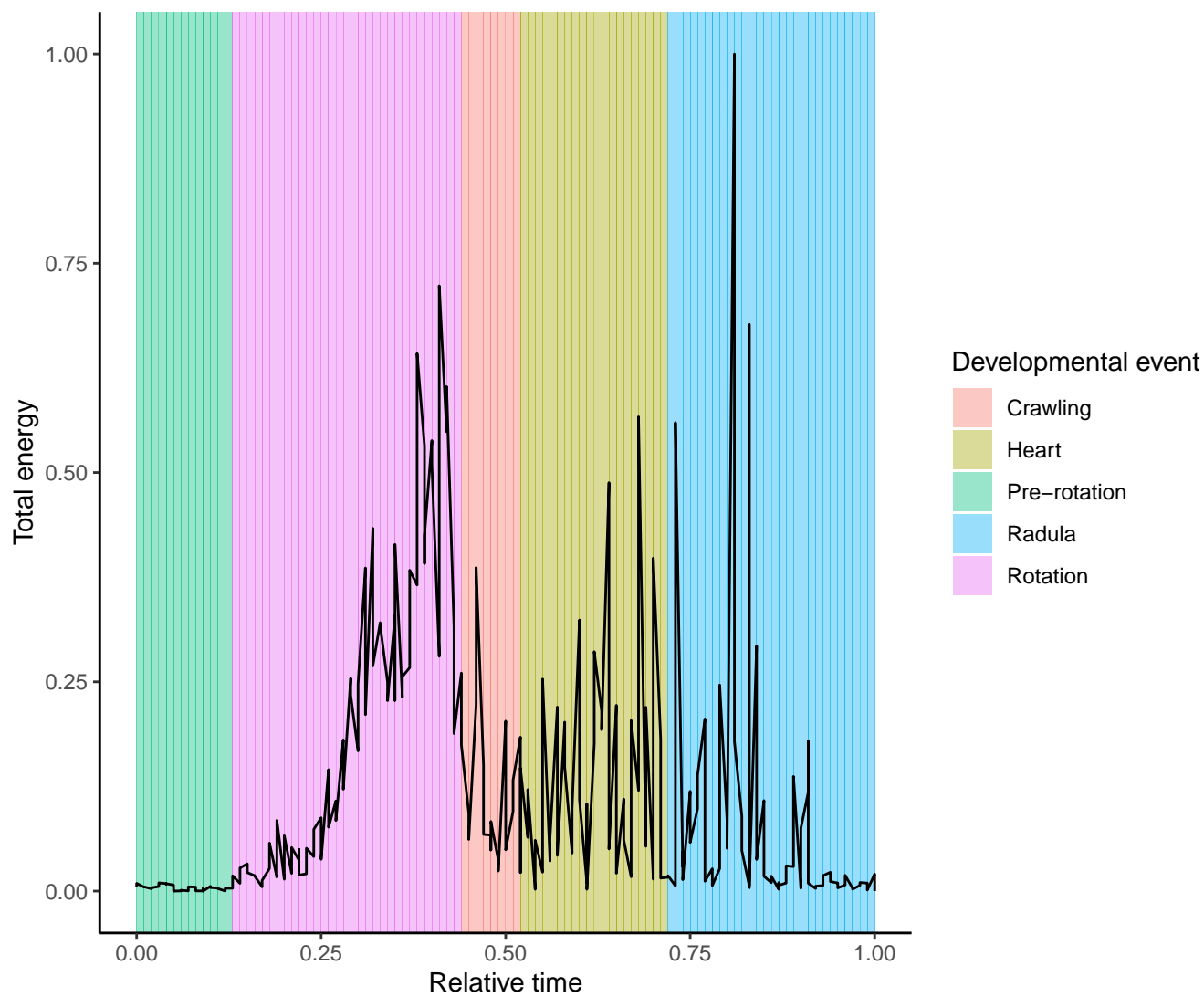

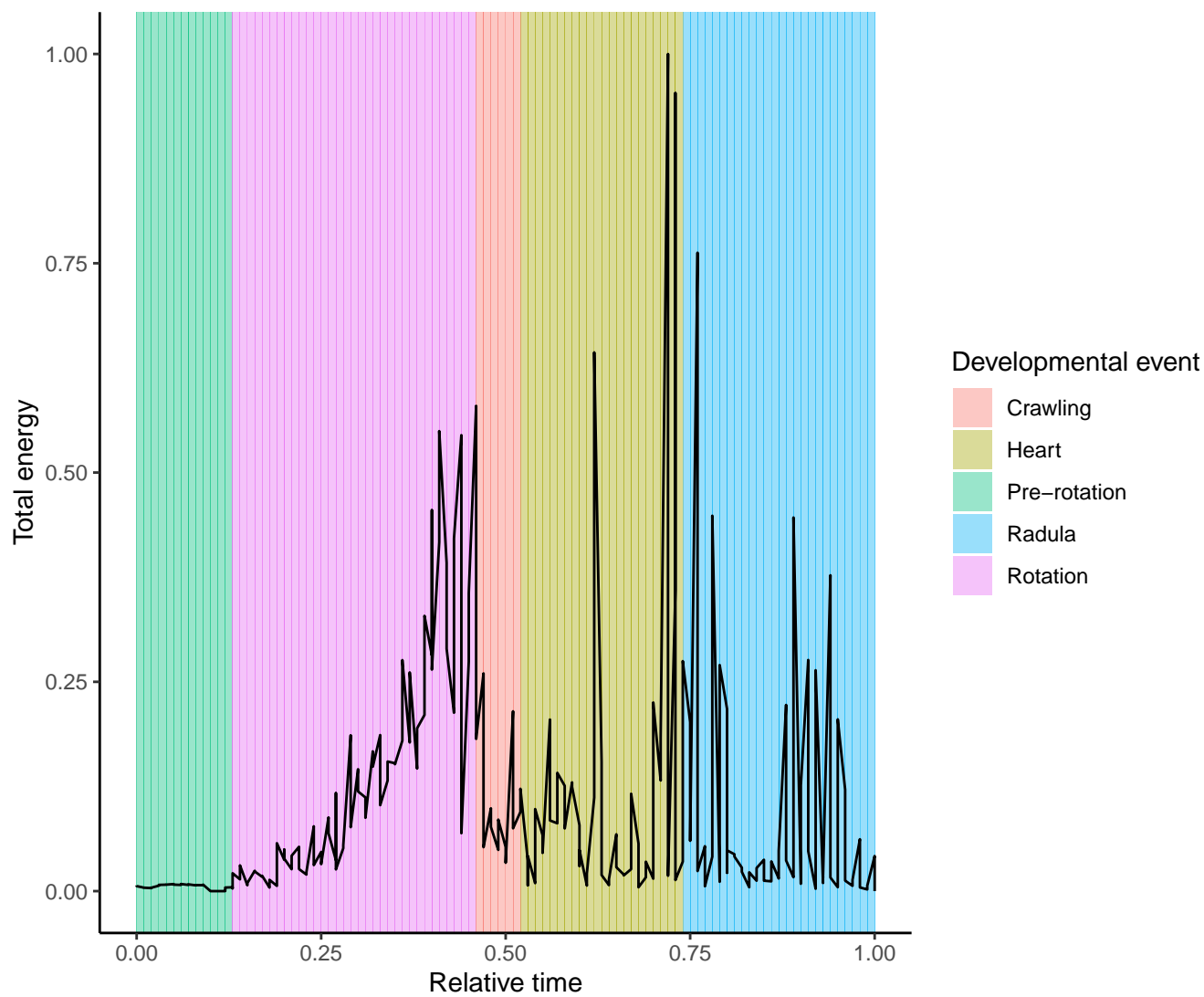

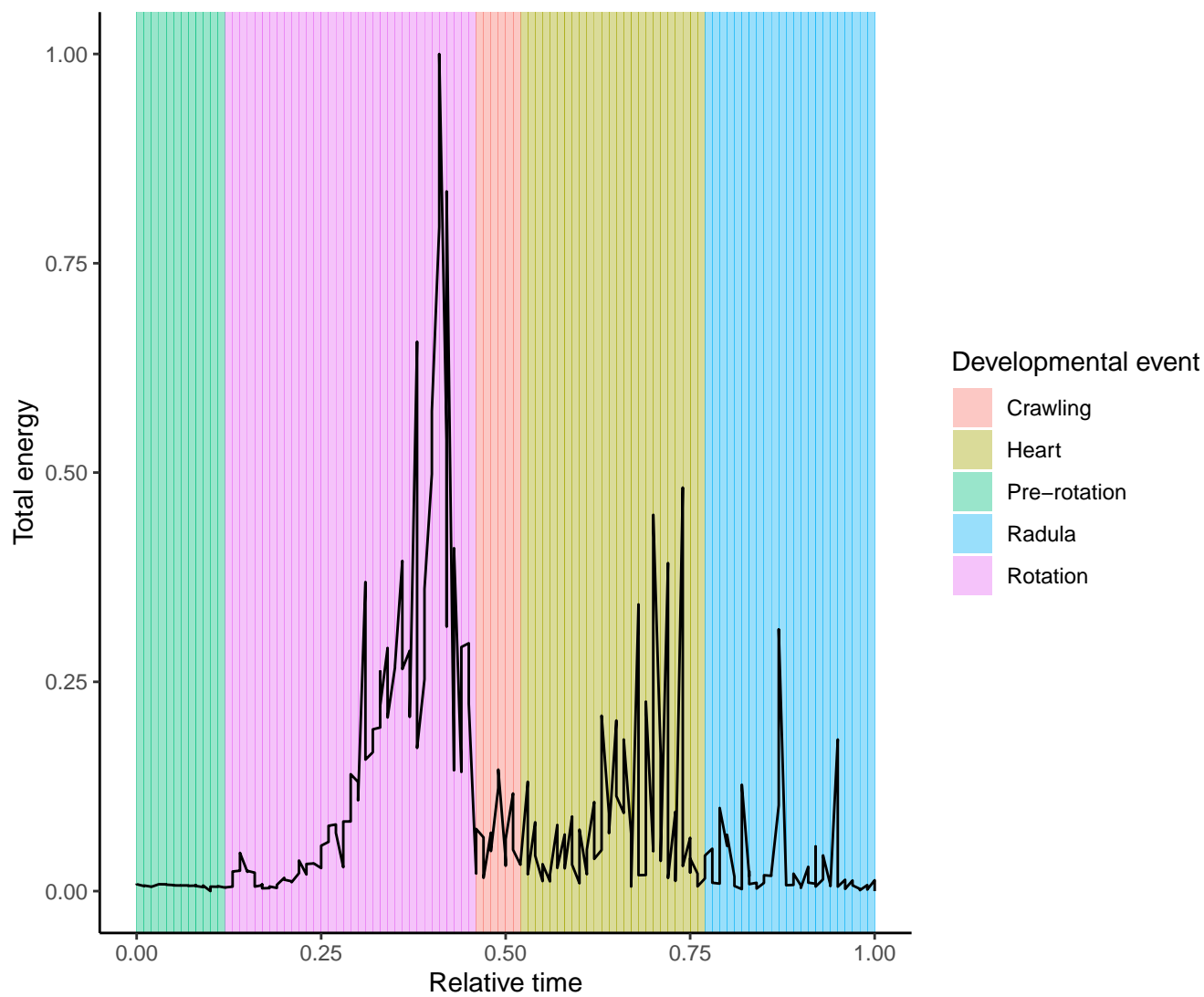

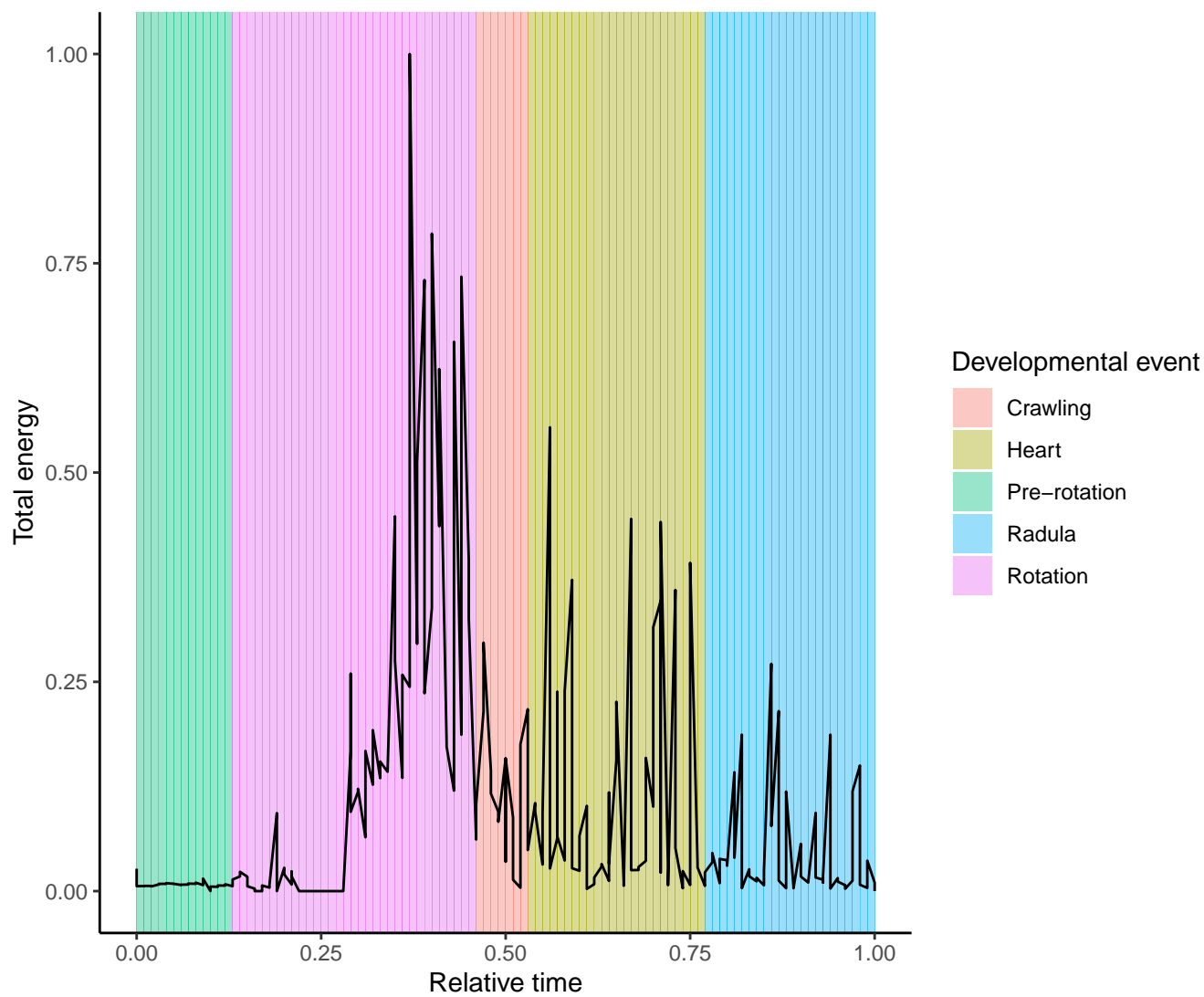

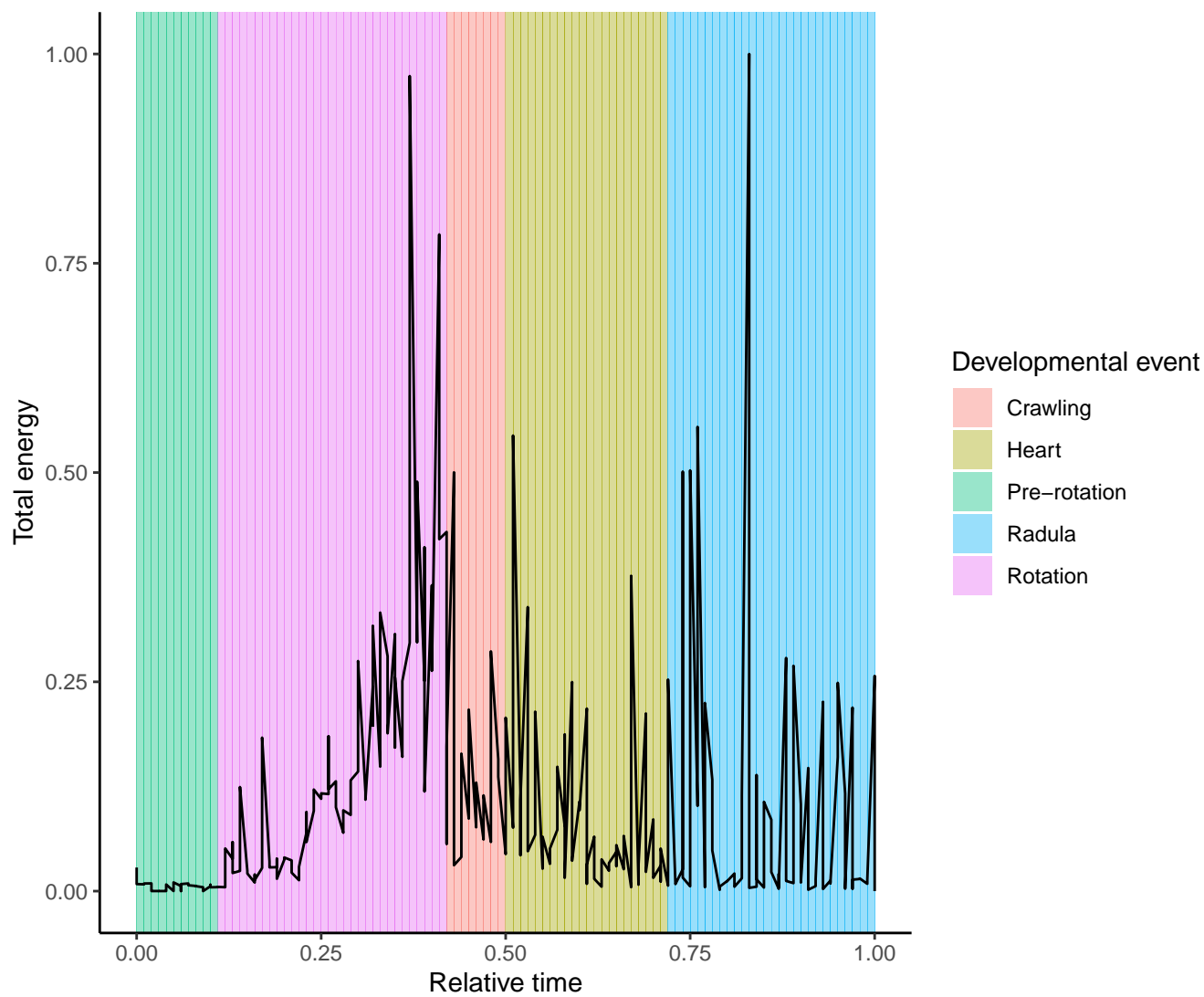

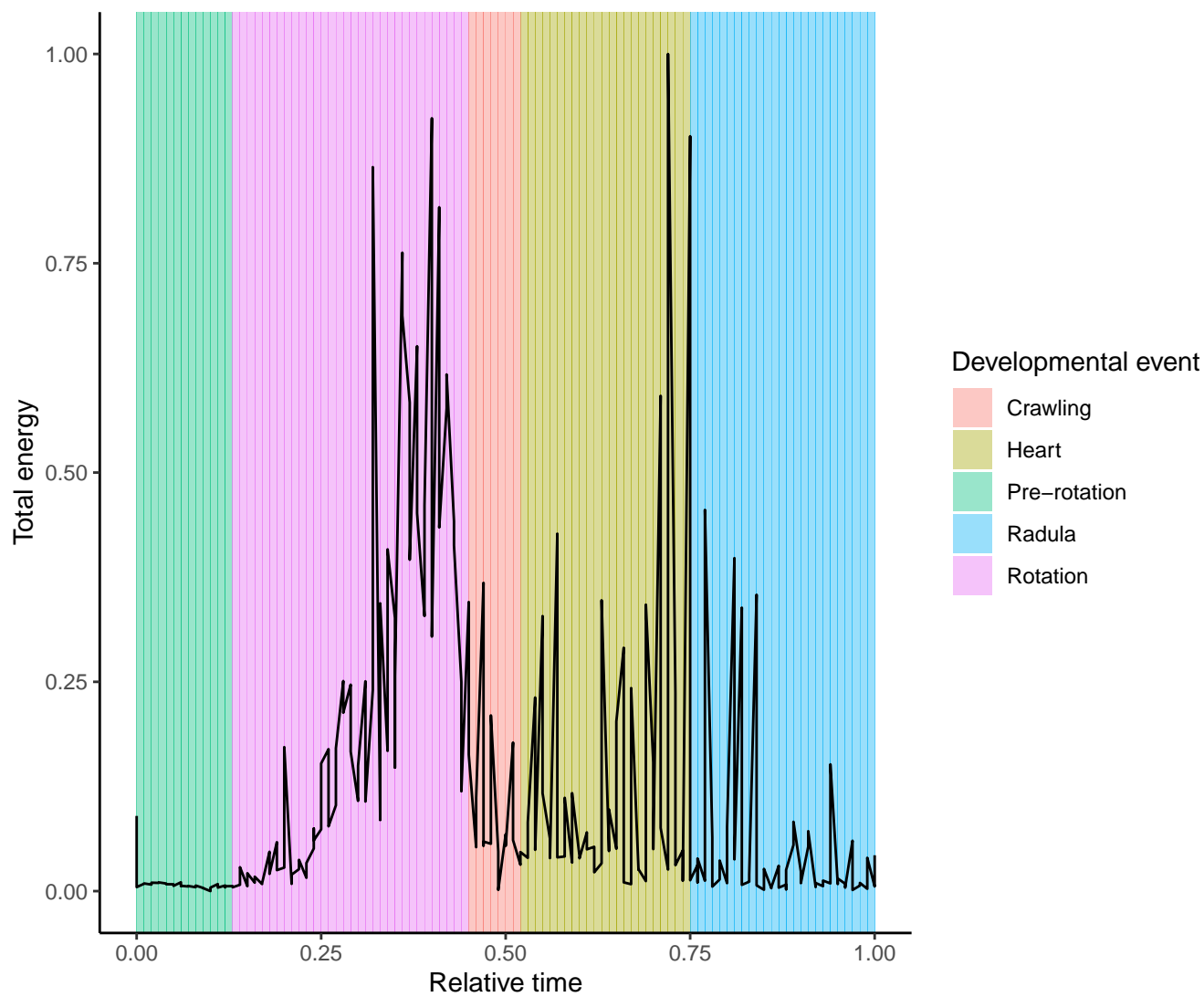

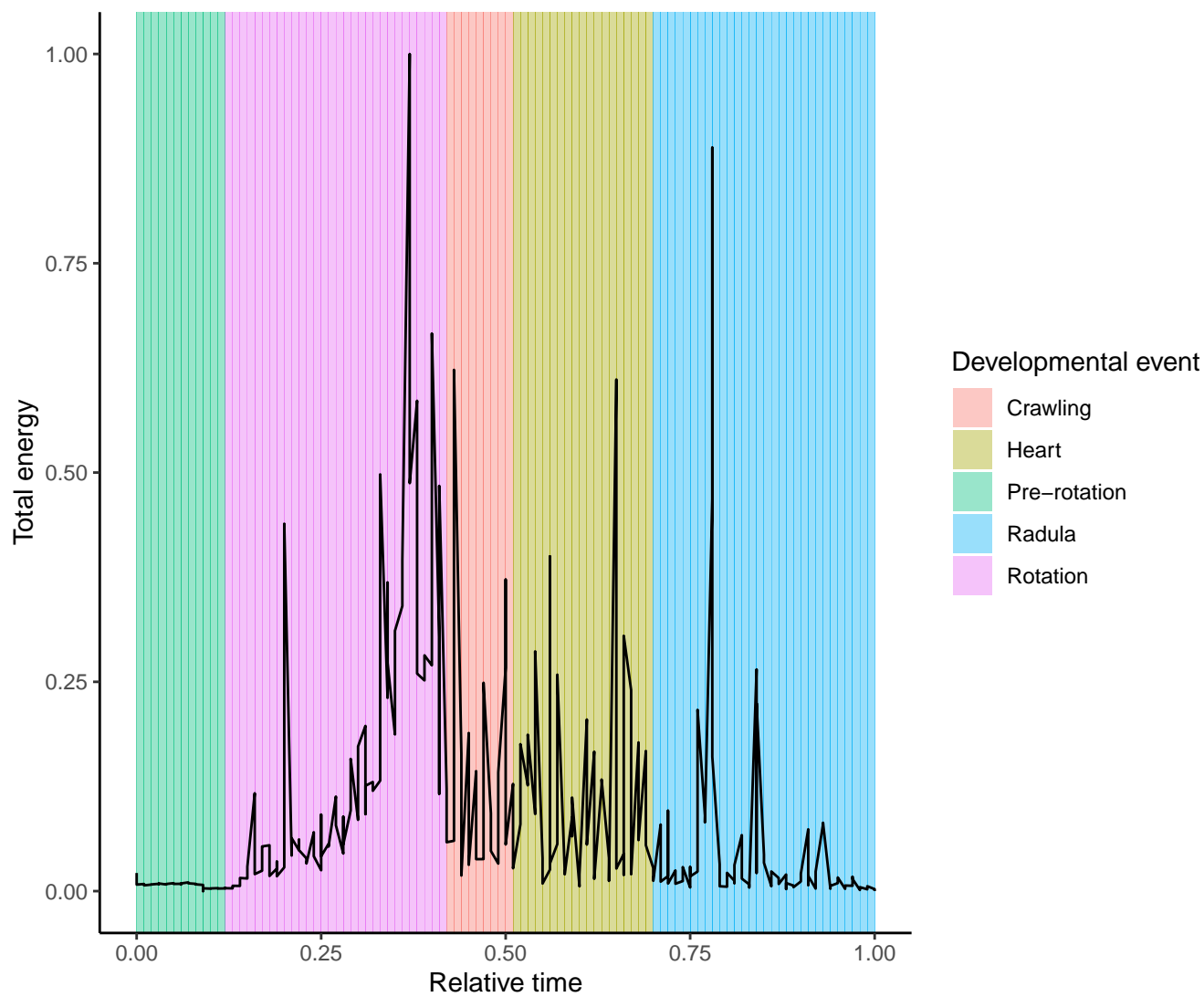

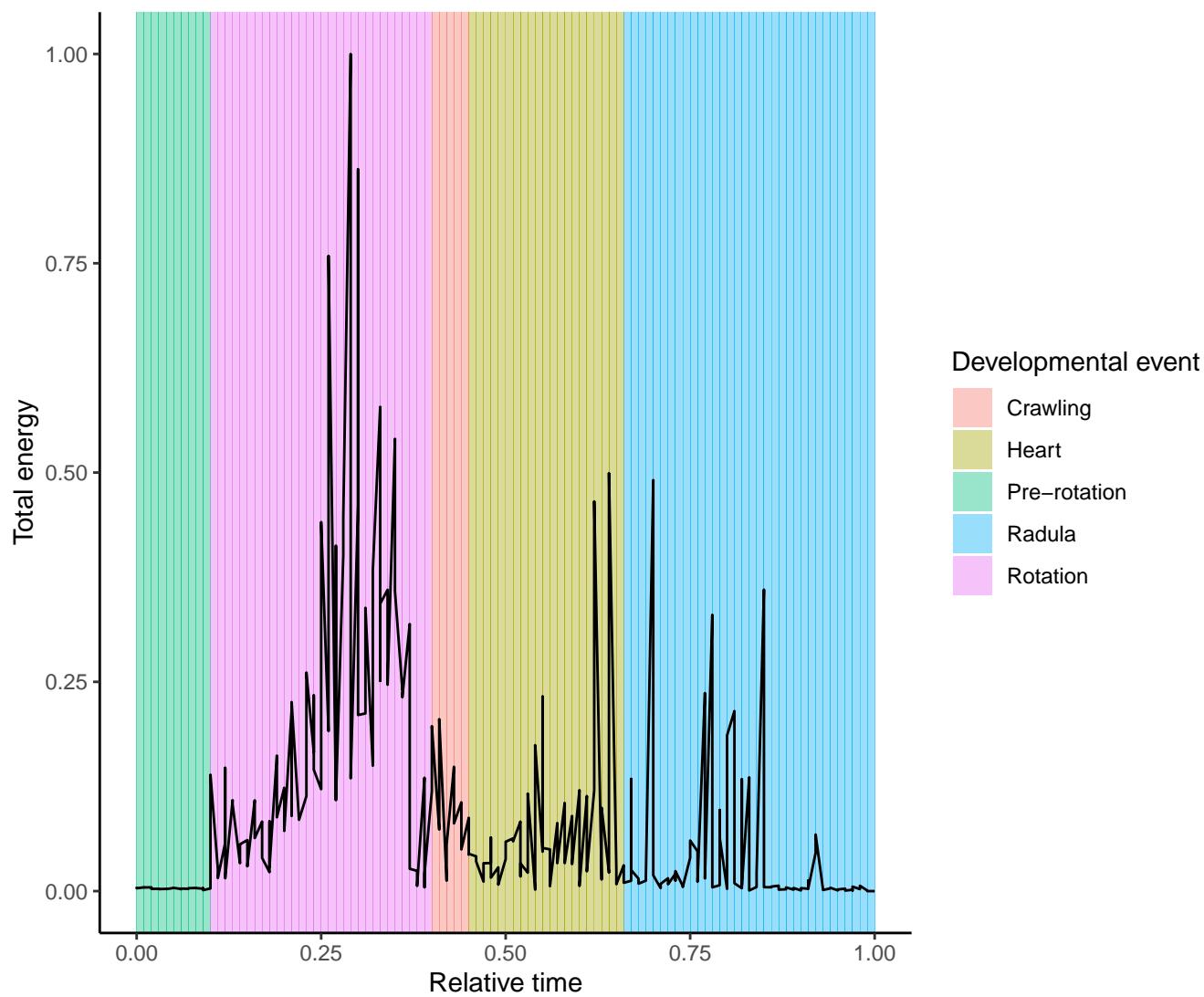

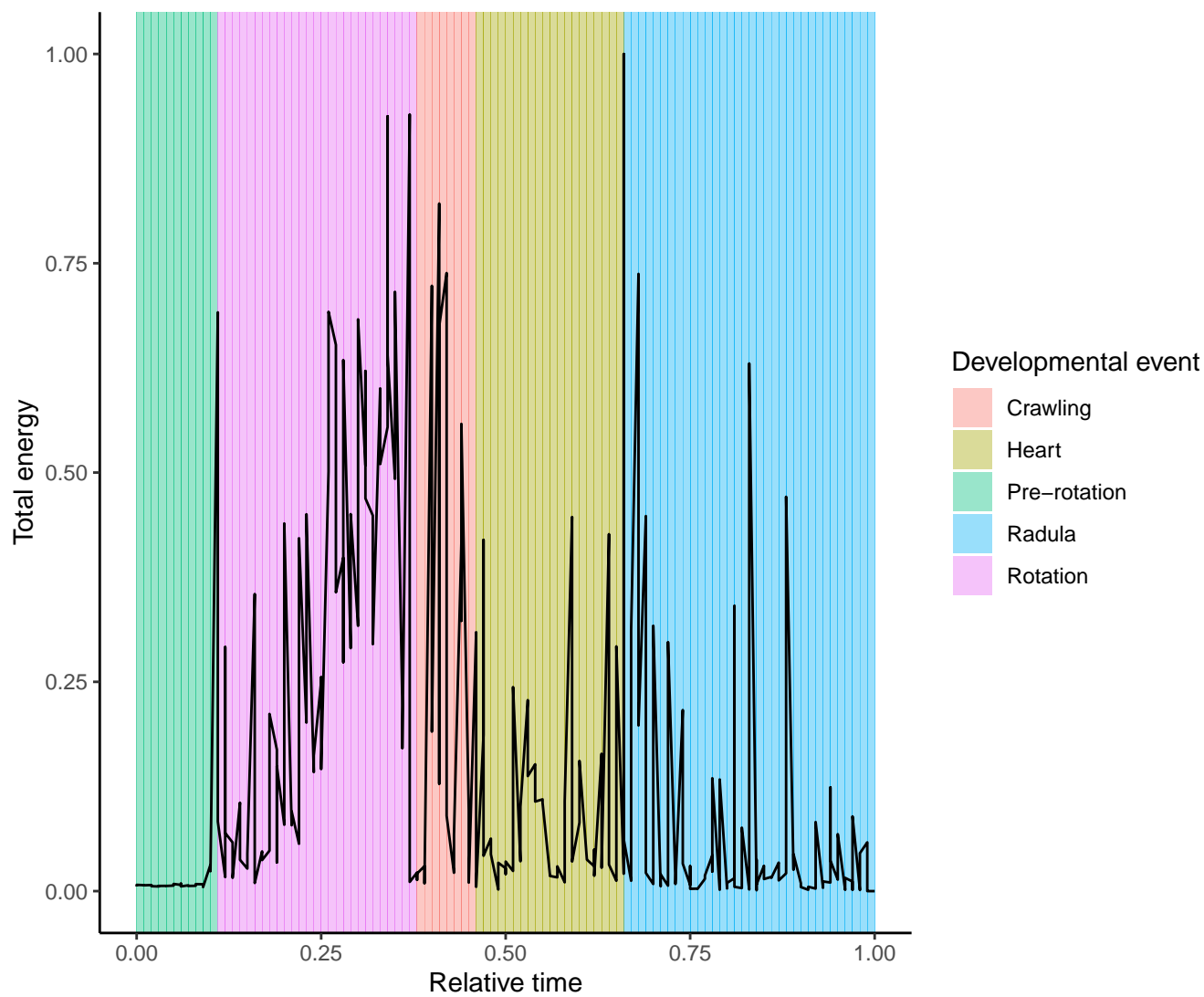

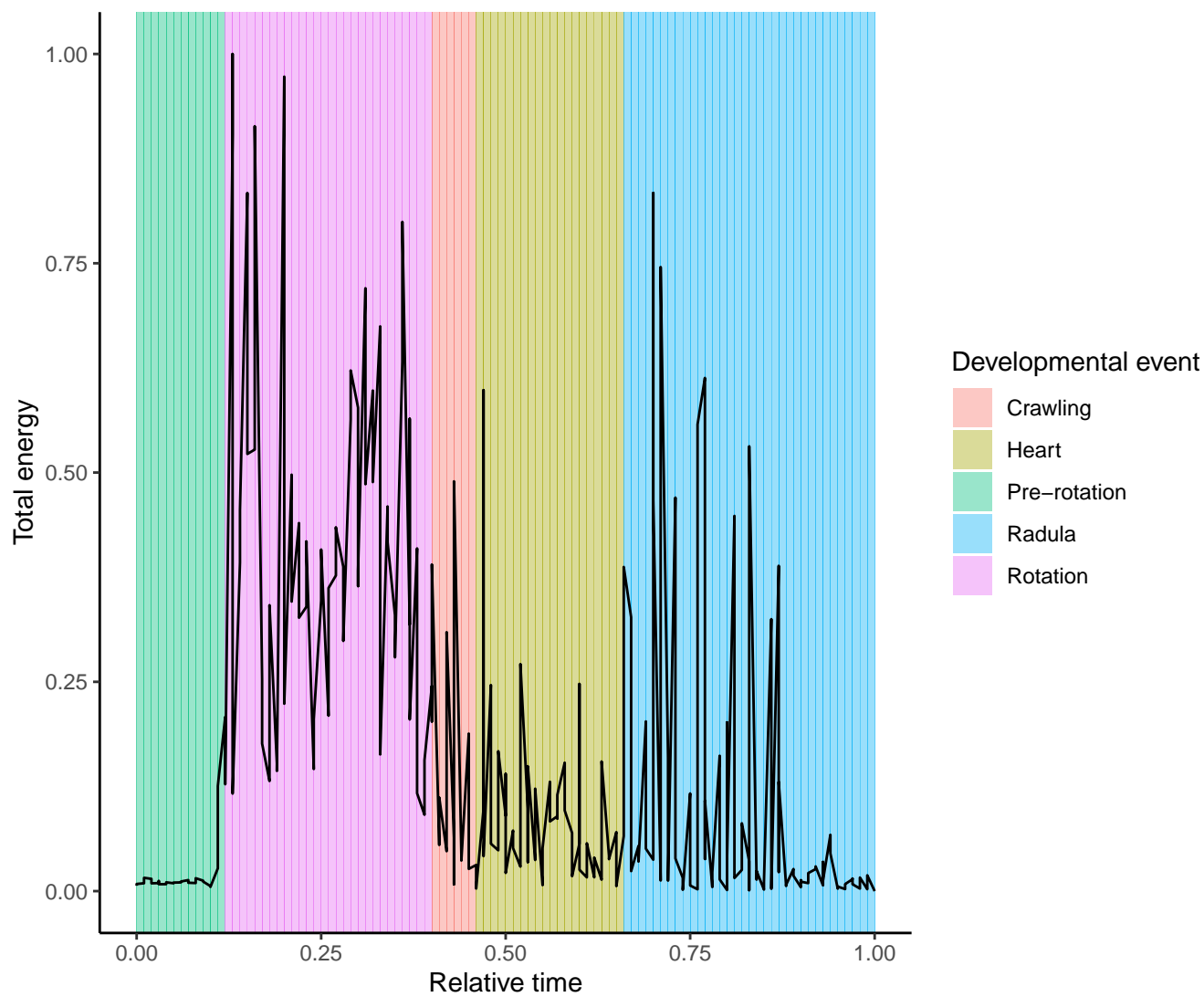

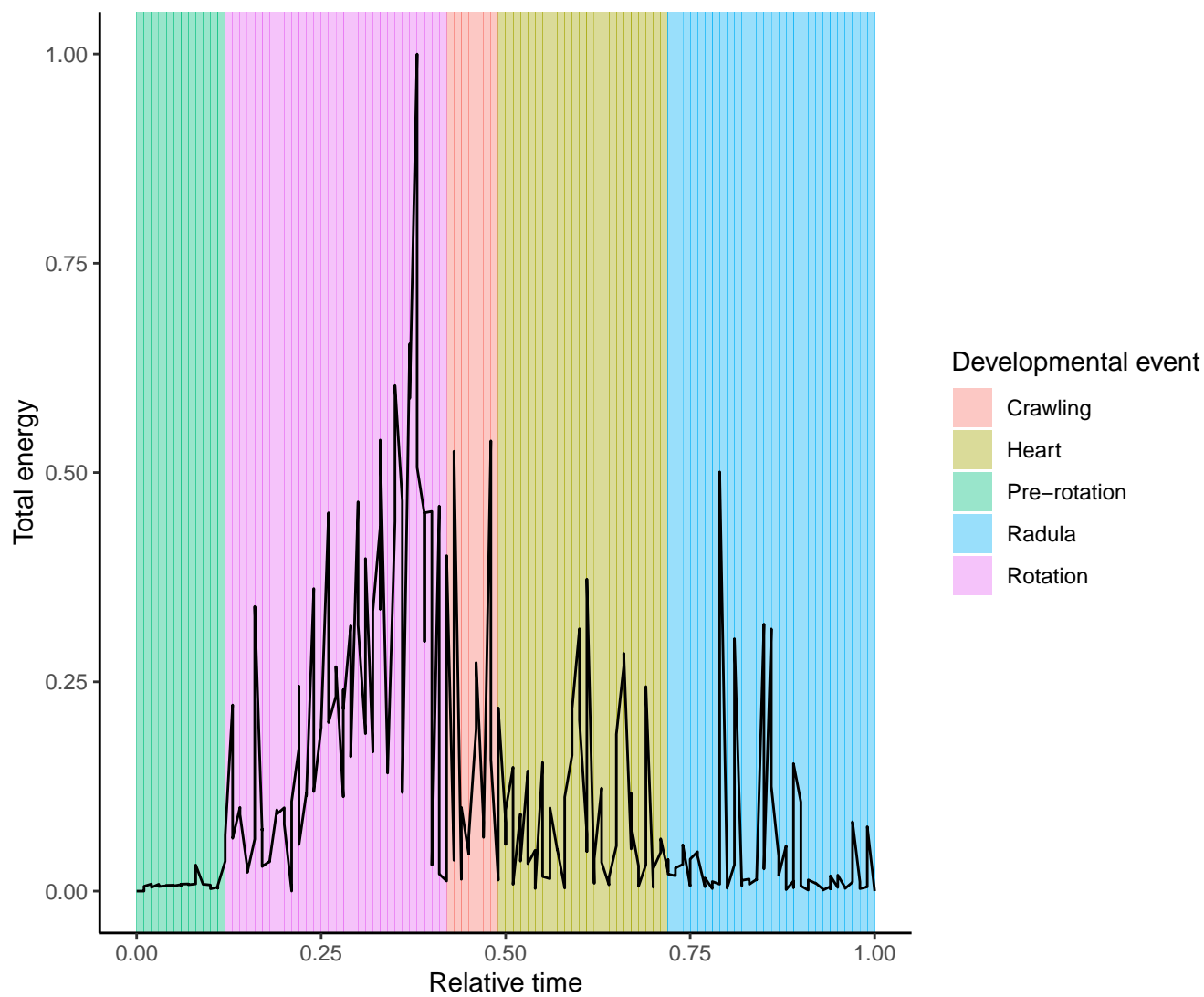

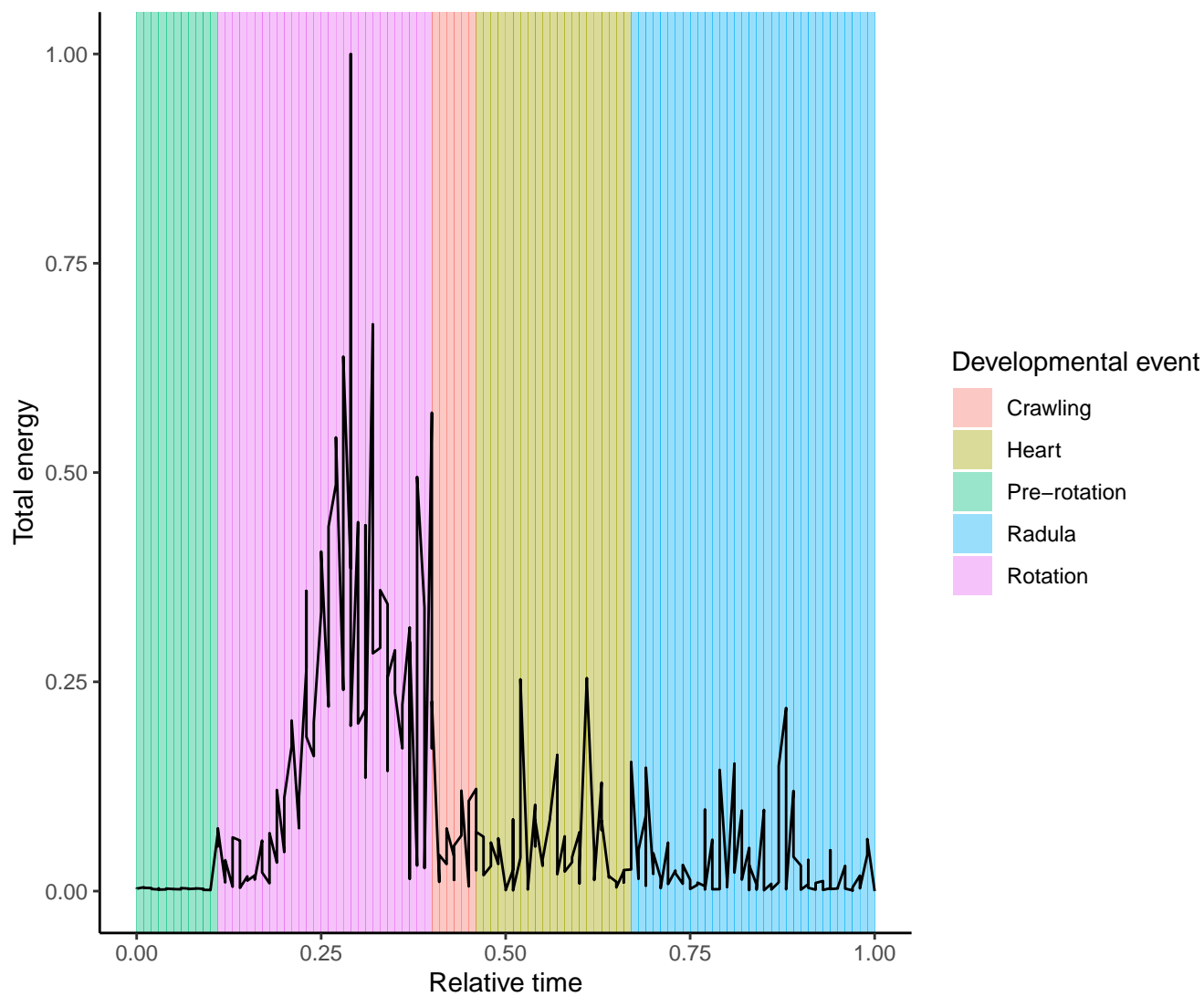

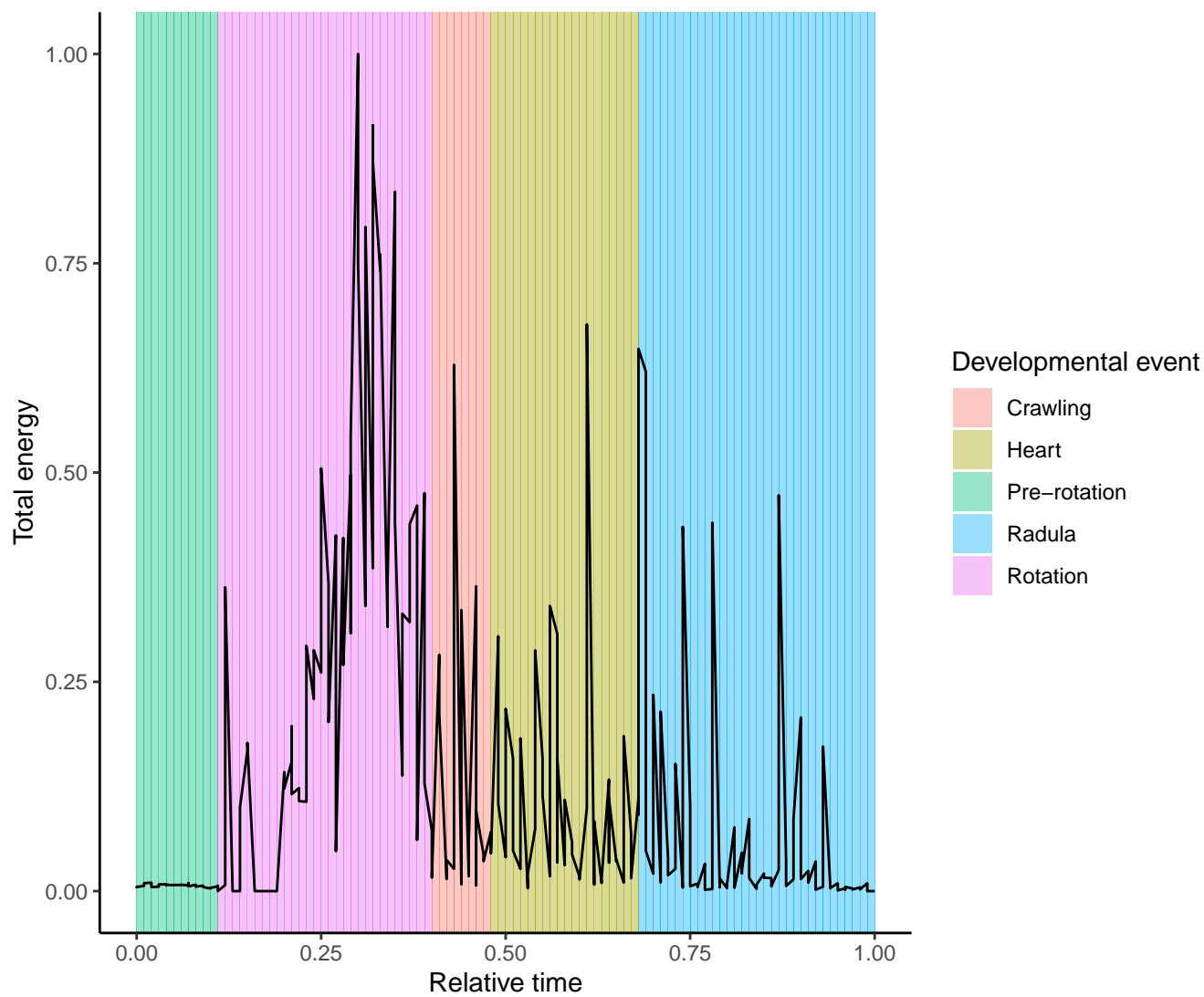

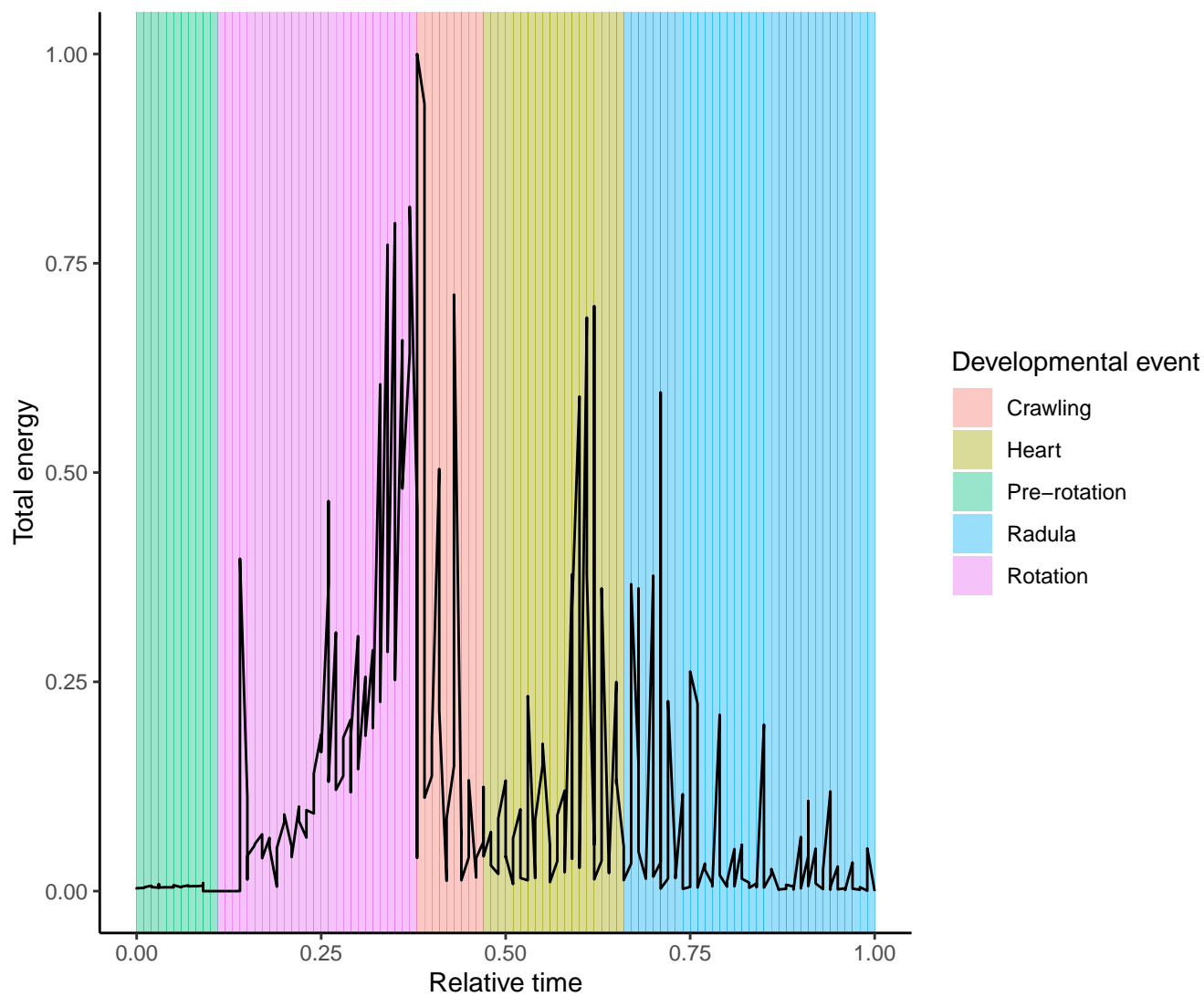

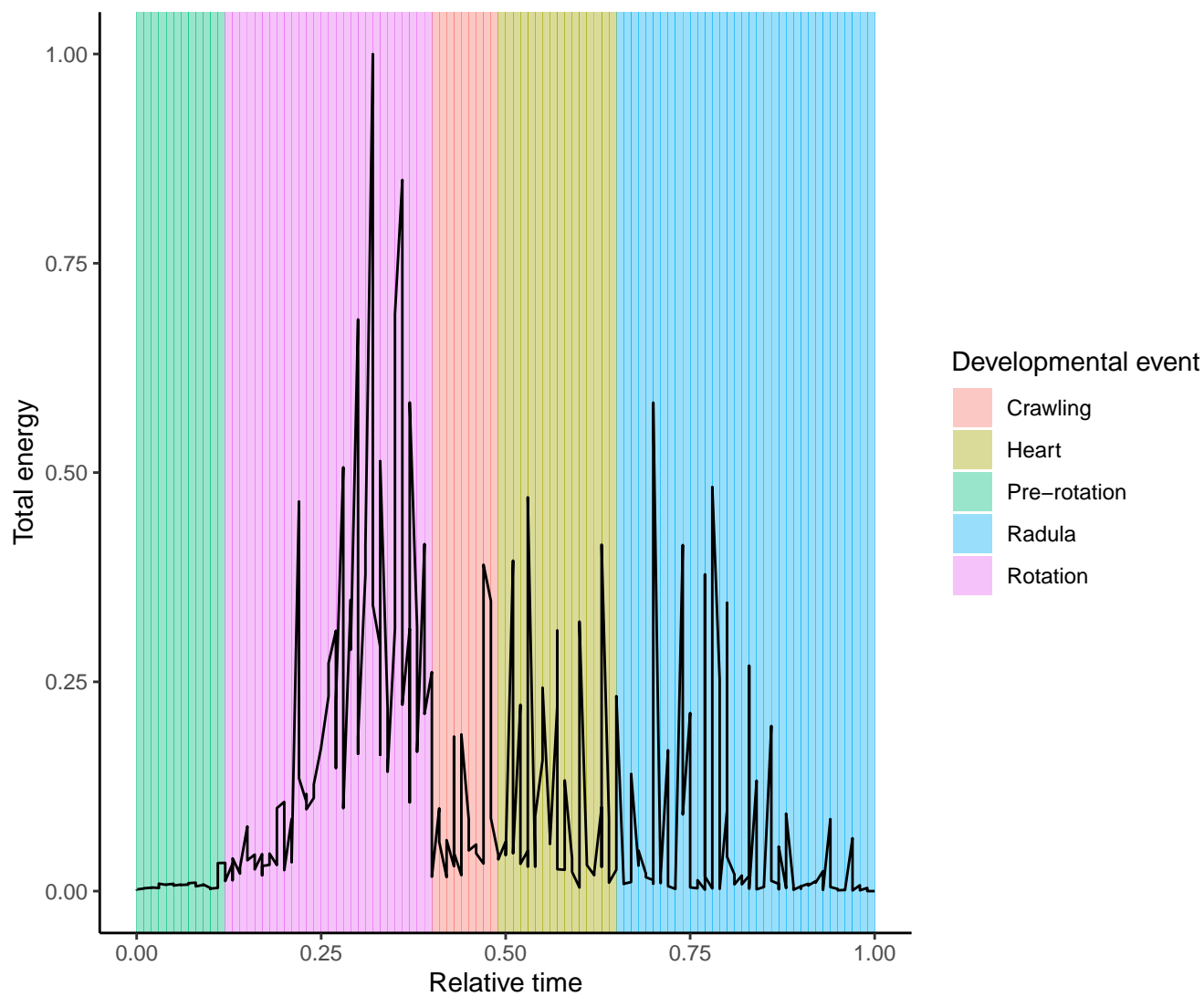

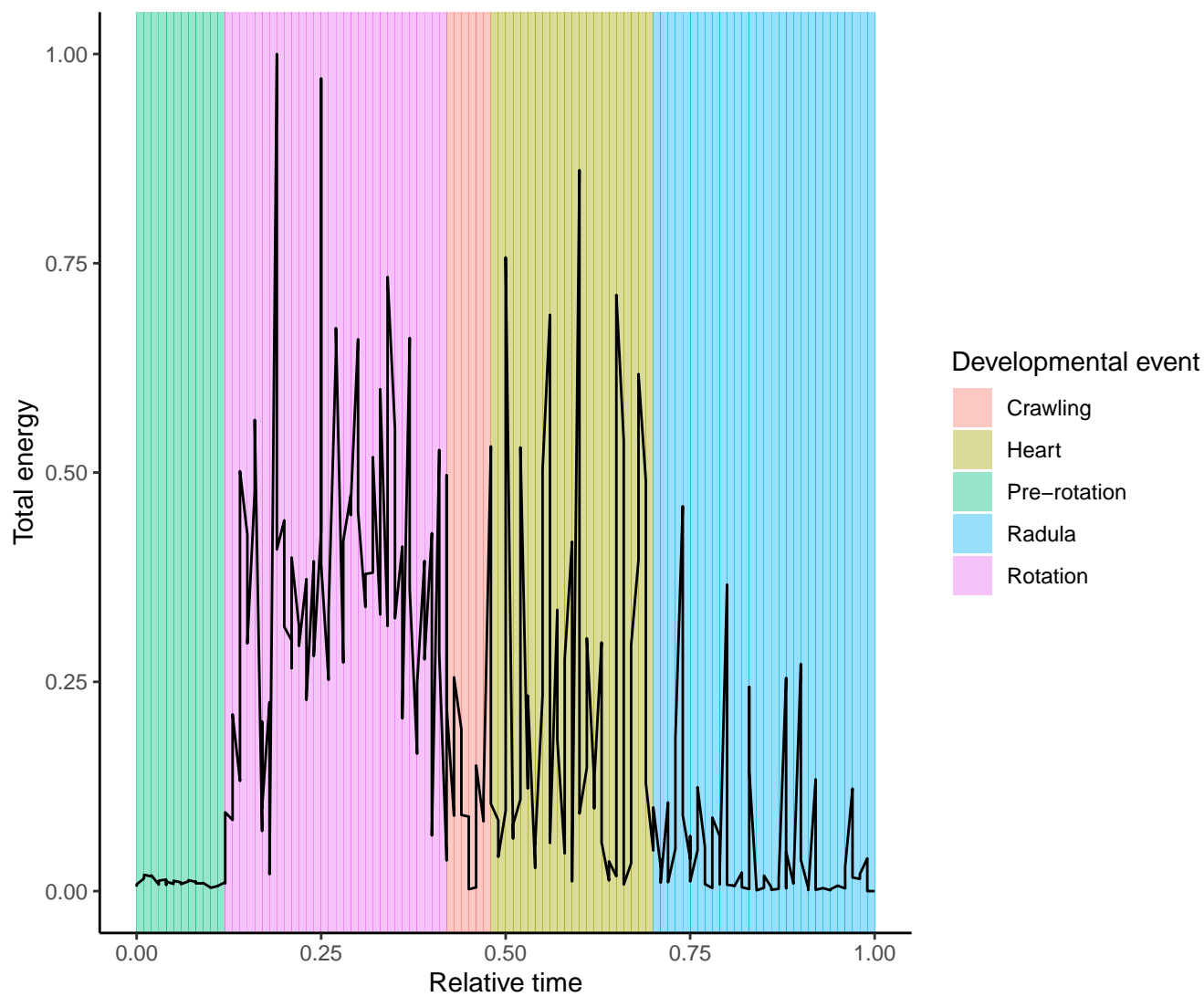

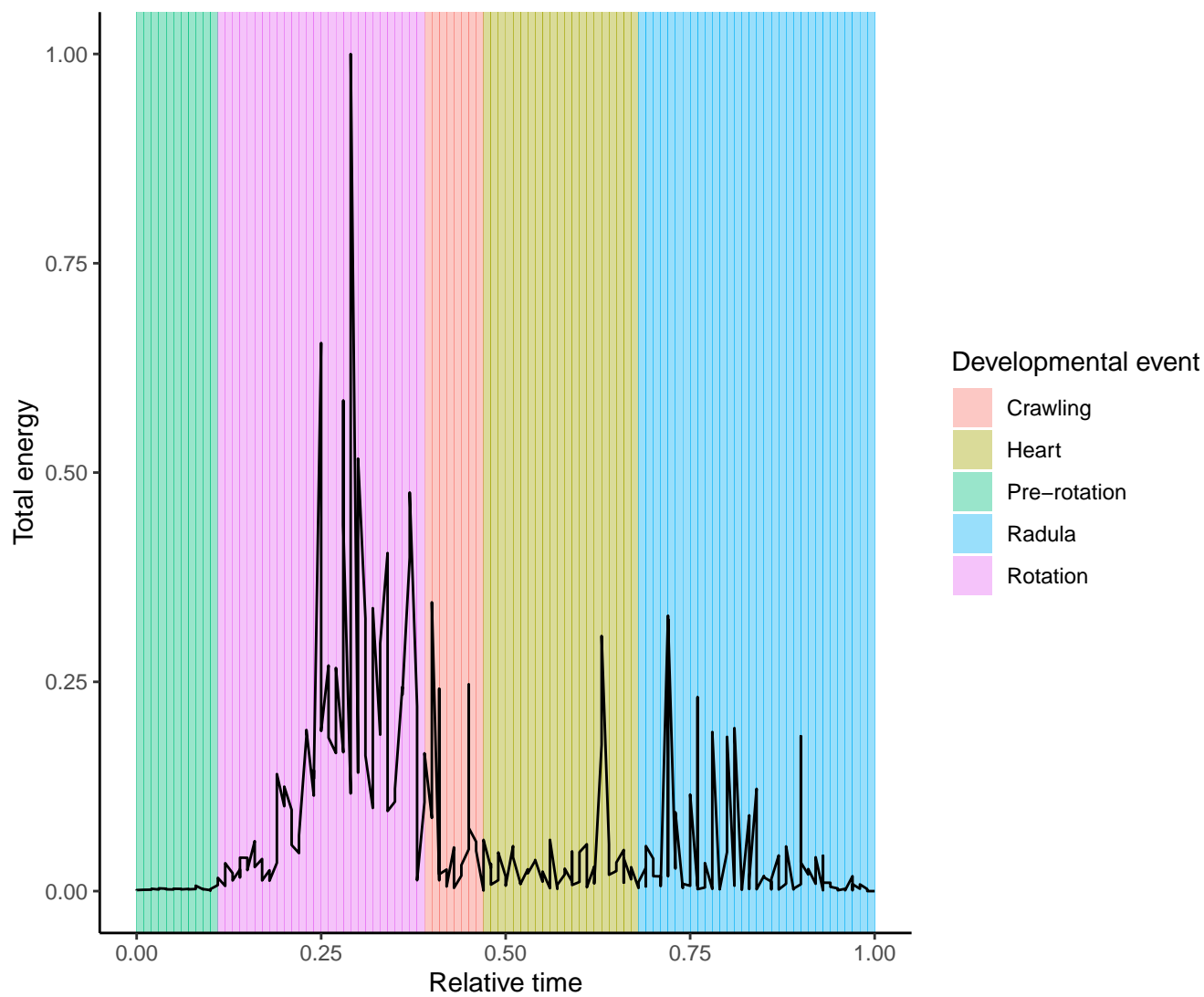

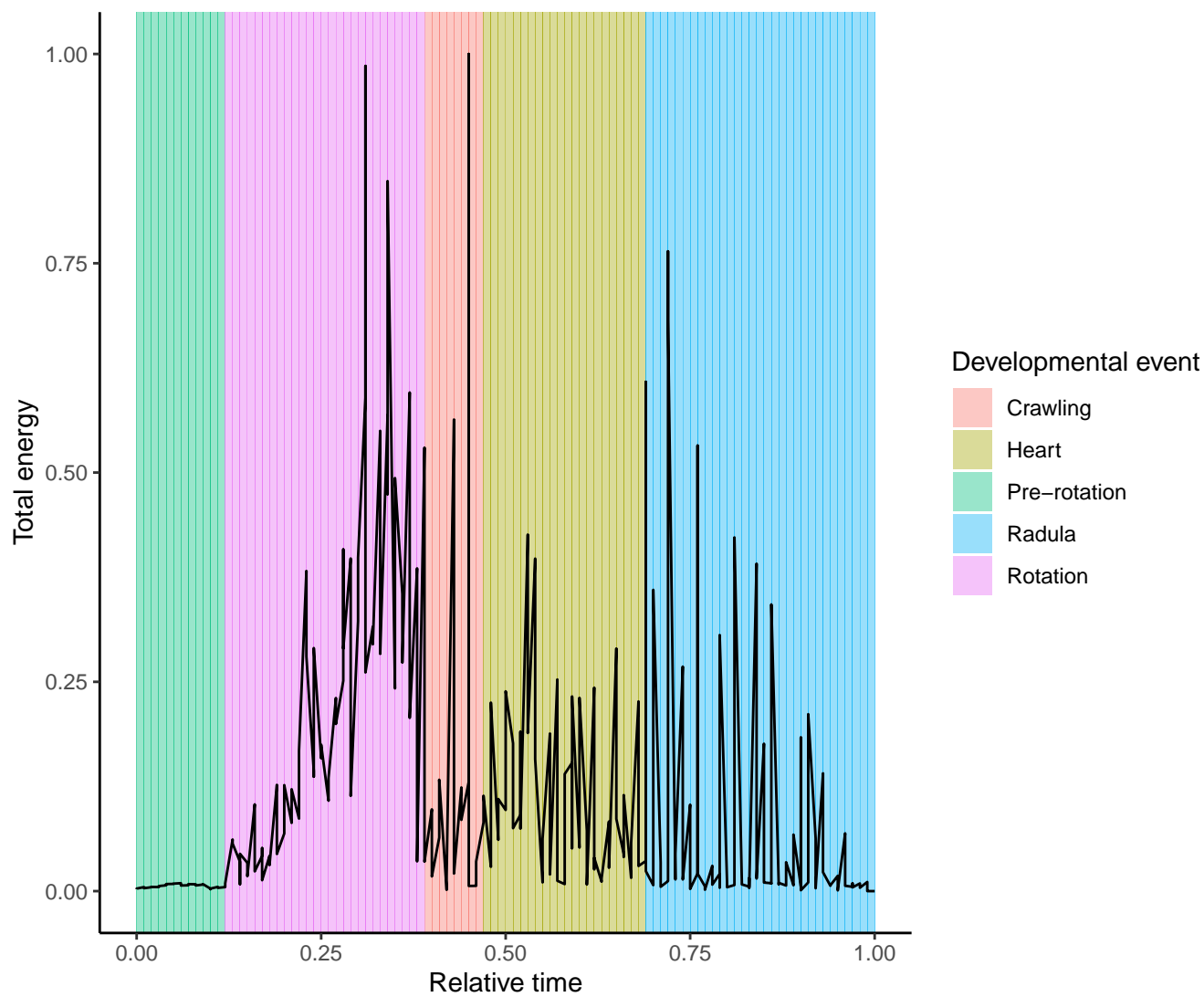

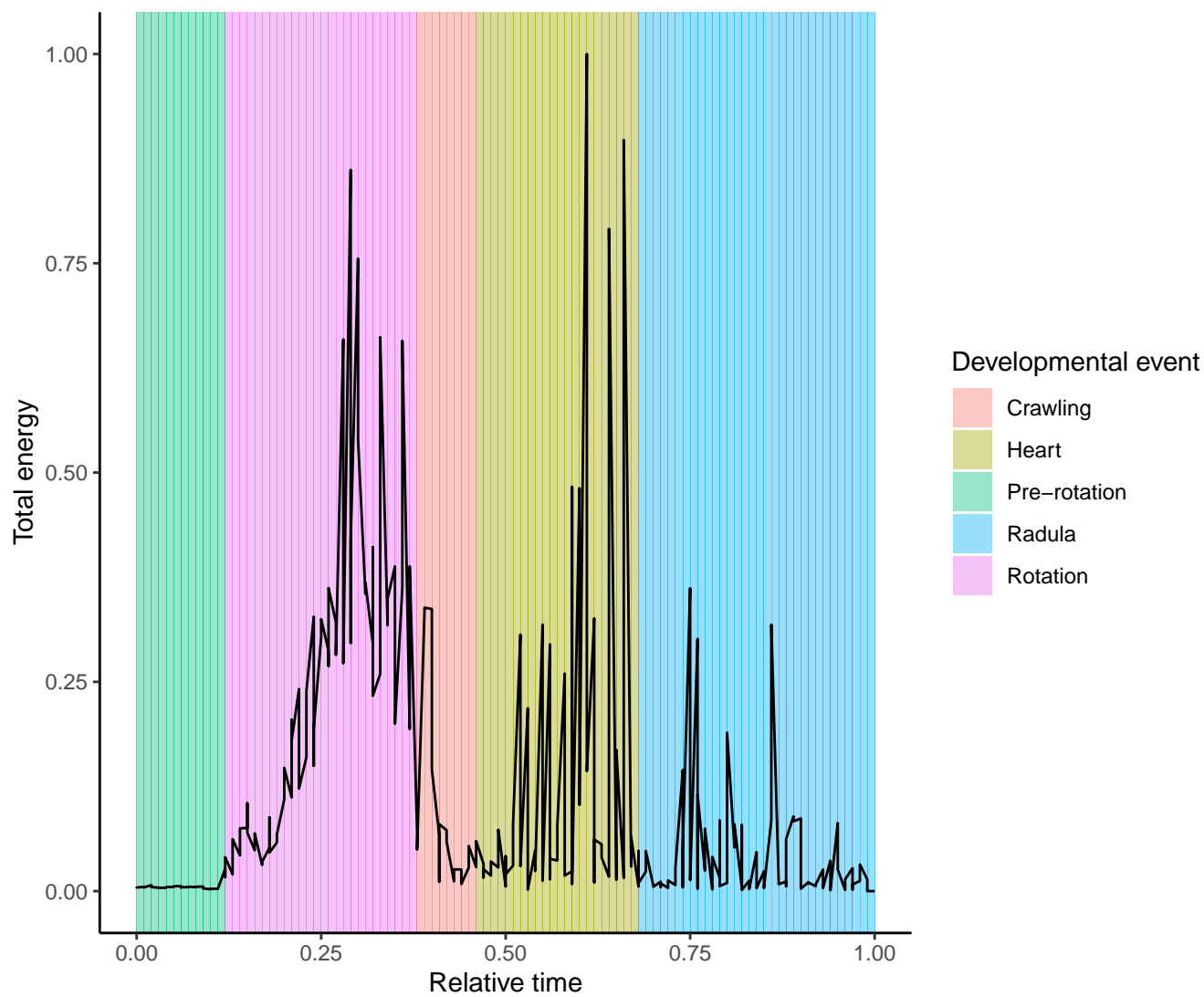

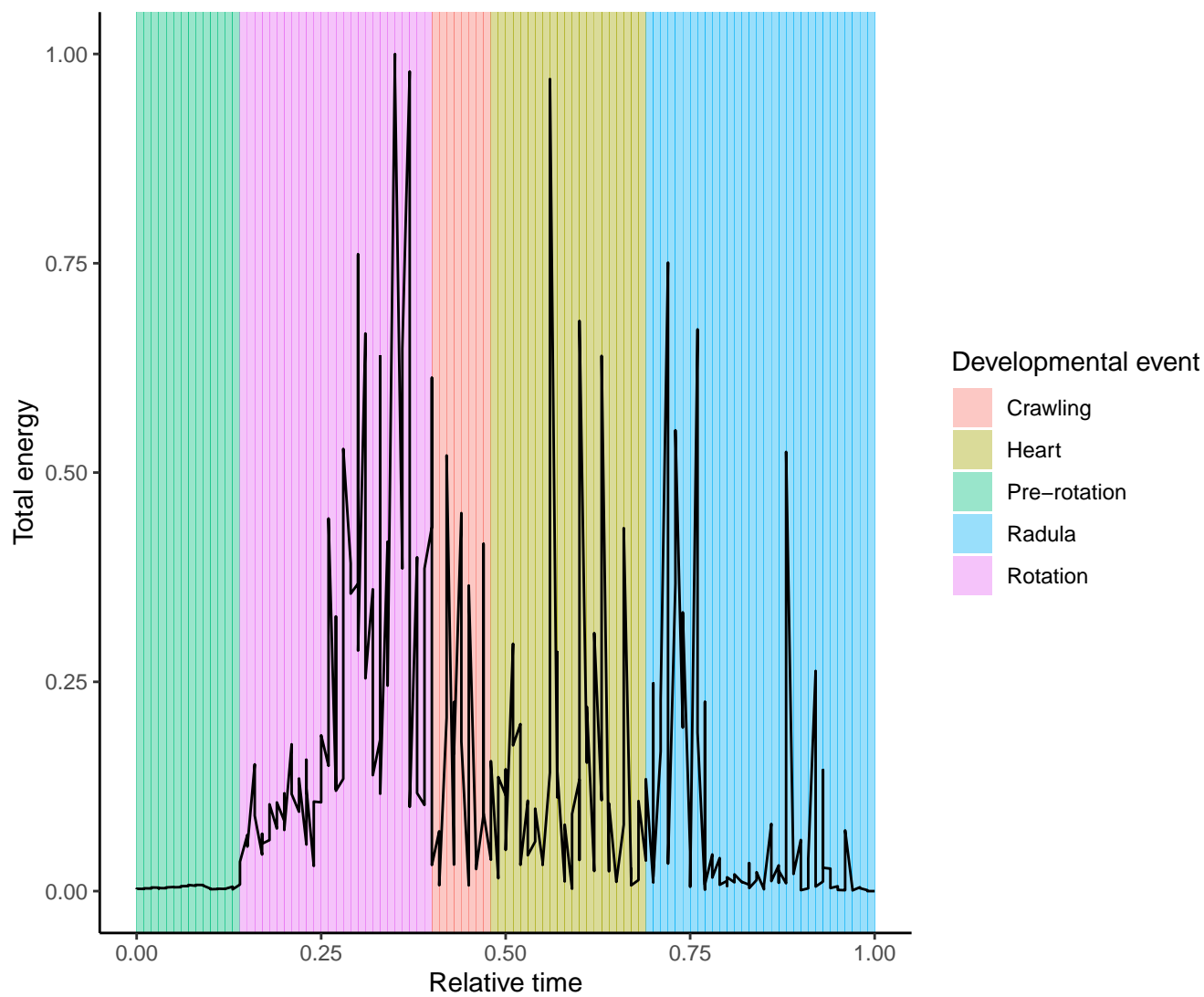

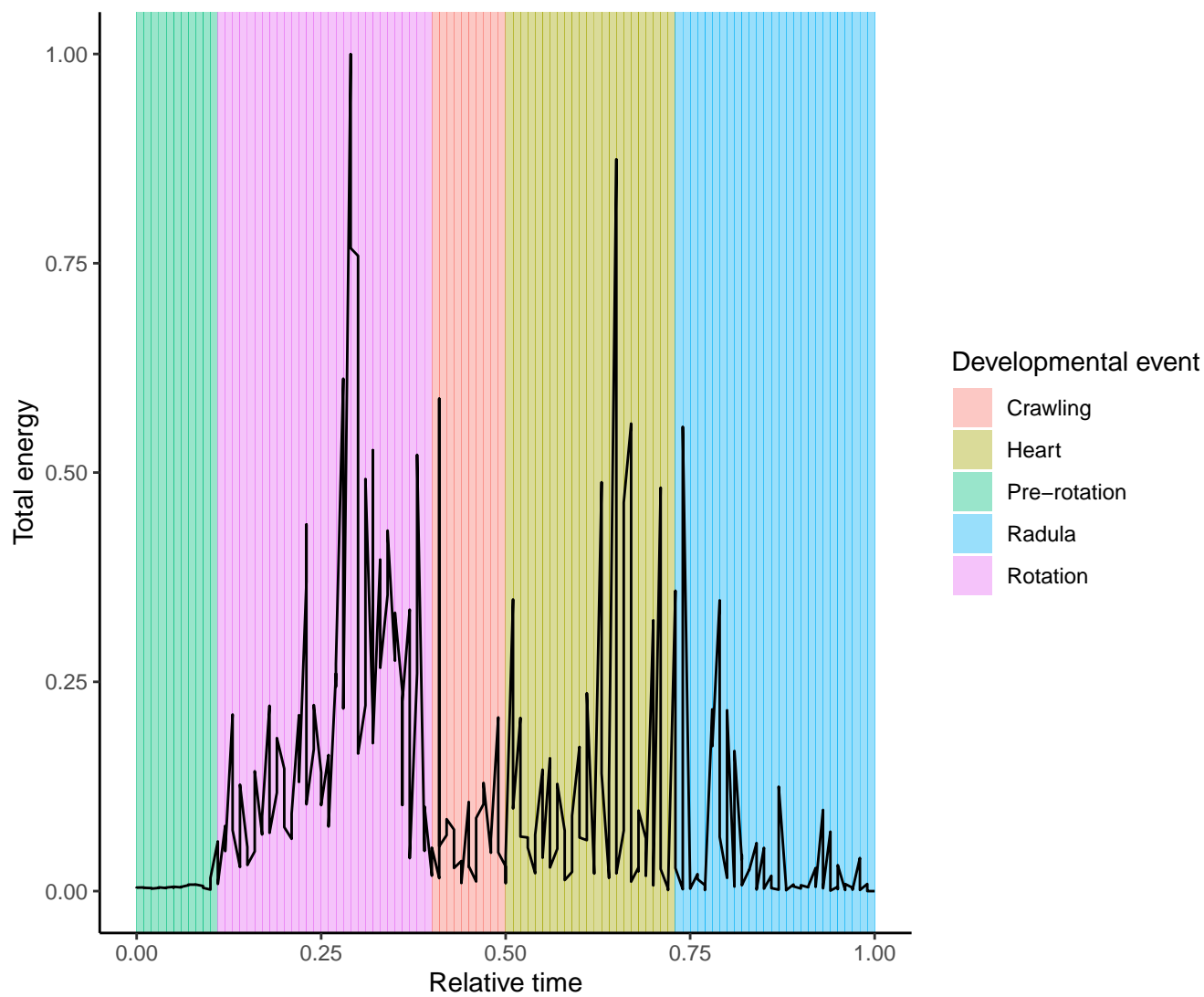

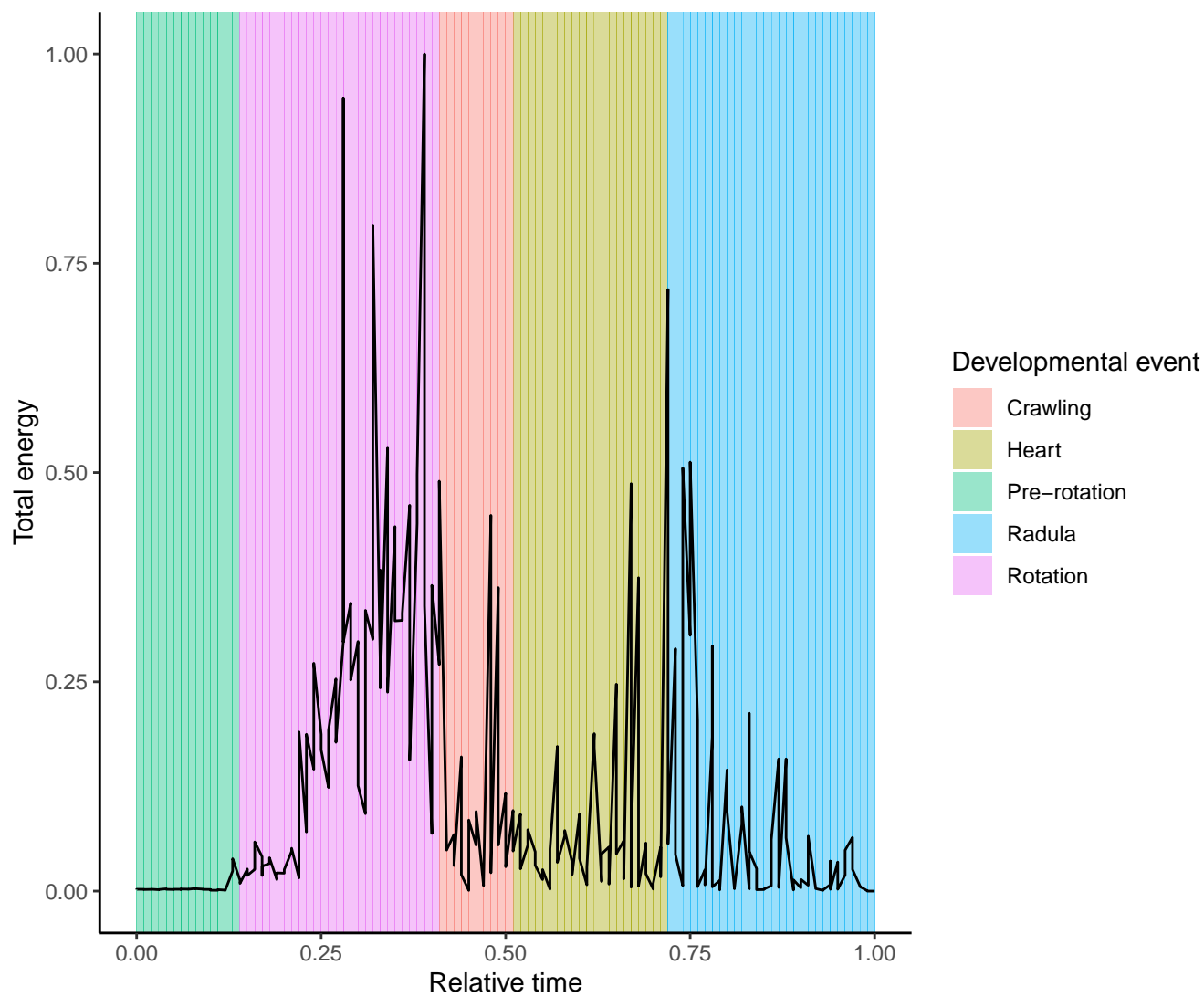

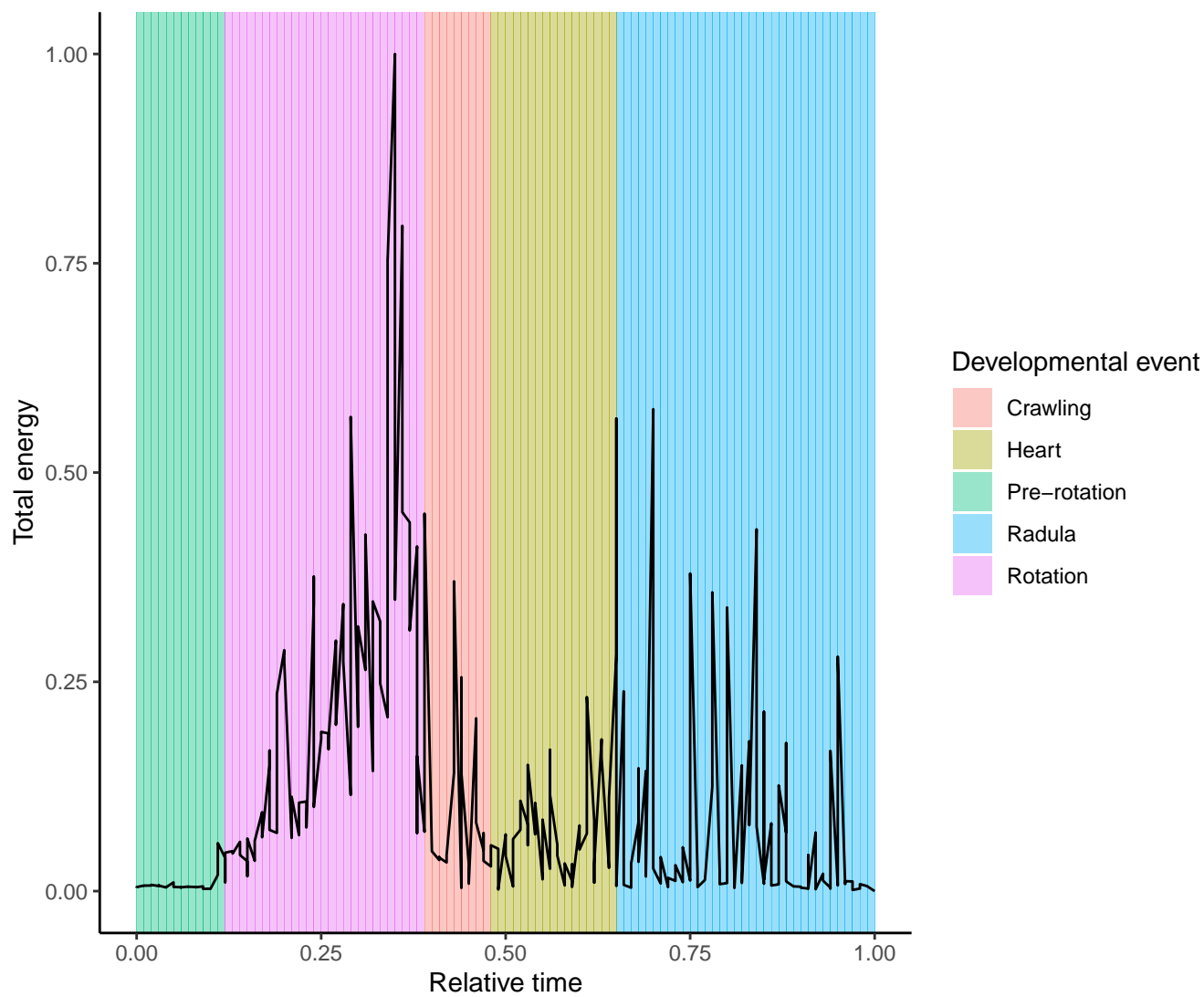

Supplement: Supplementary file 3 [file Image2.PDF]

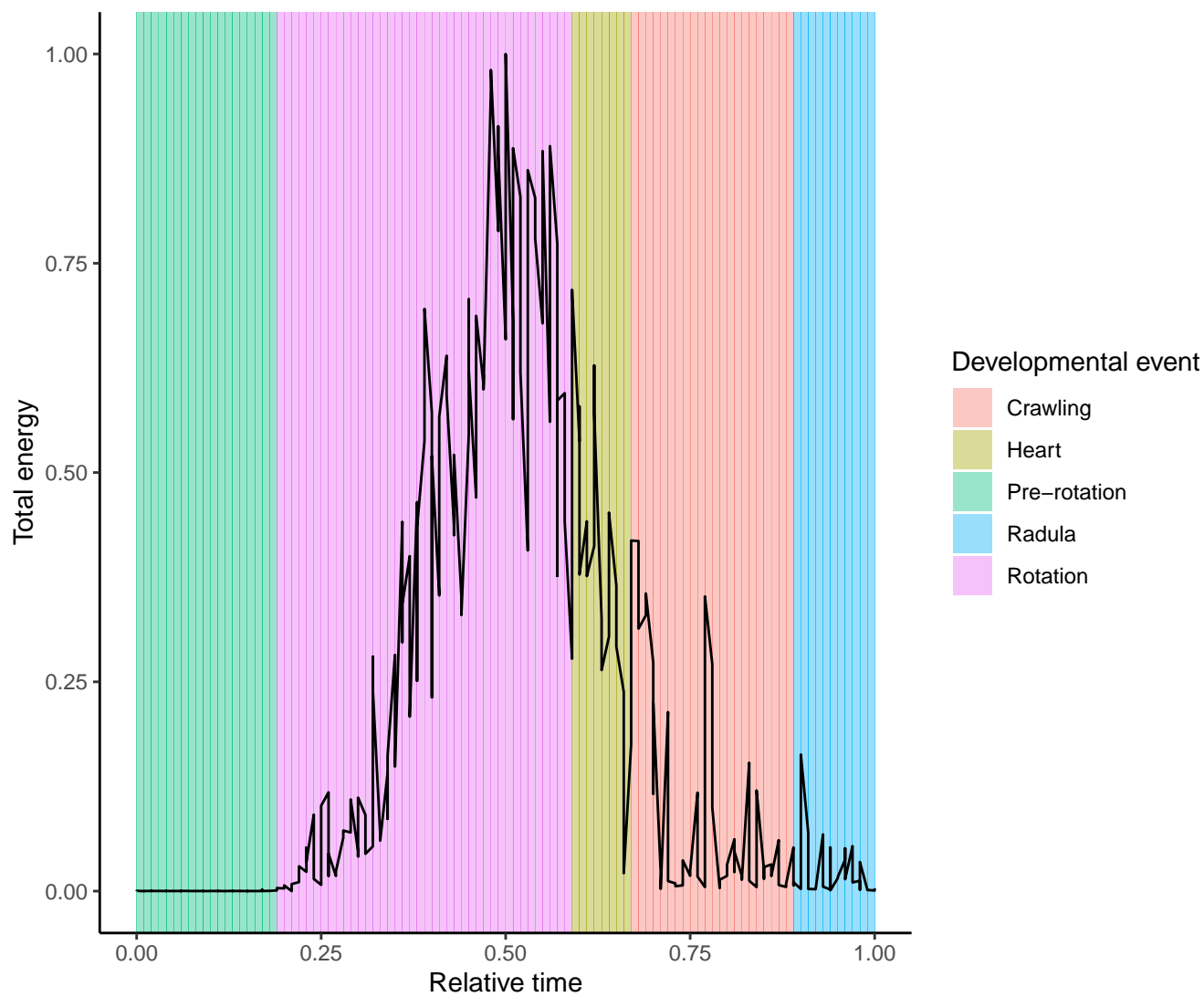

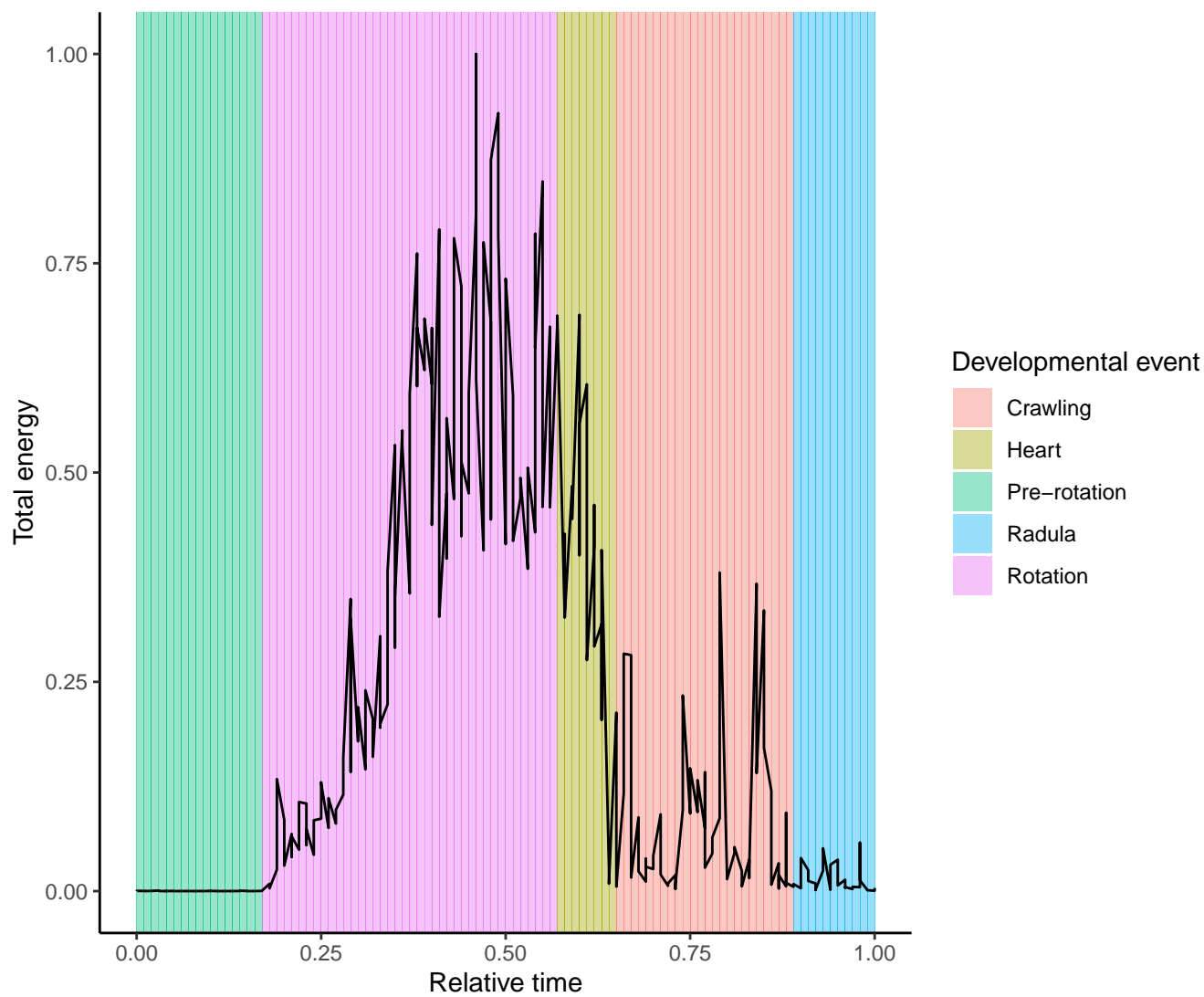

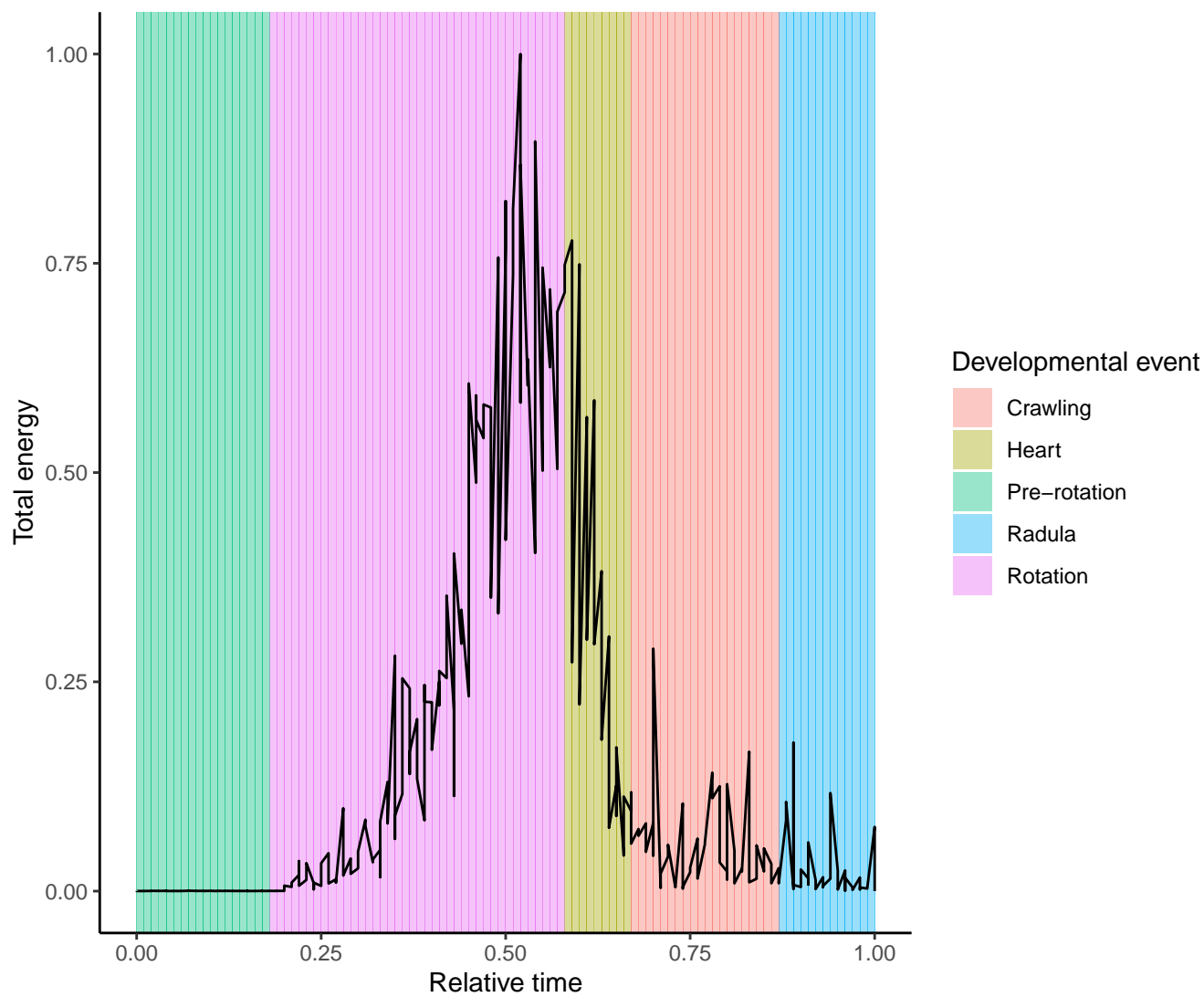

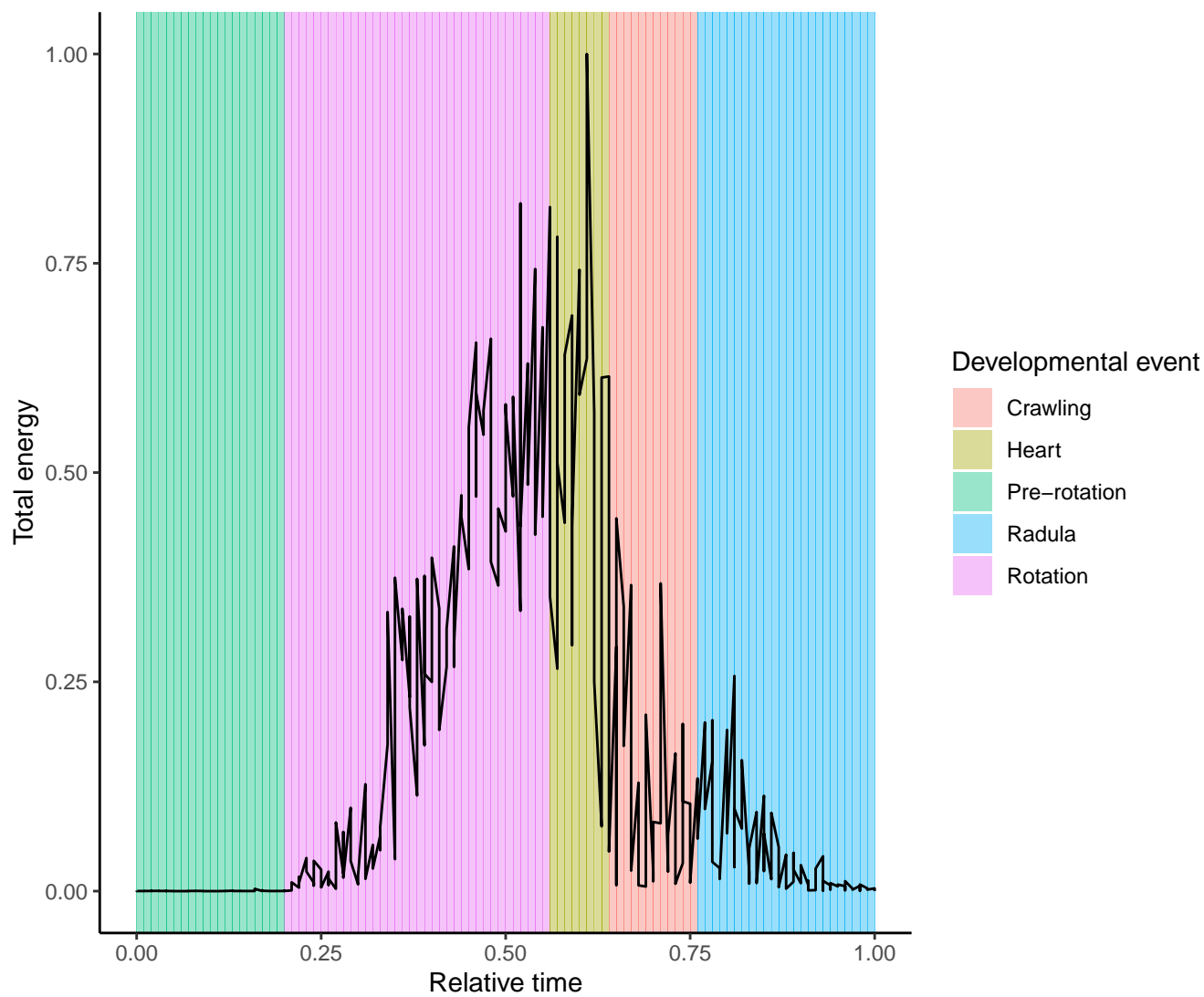

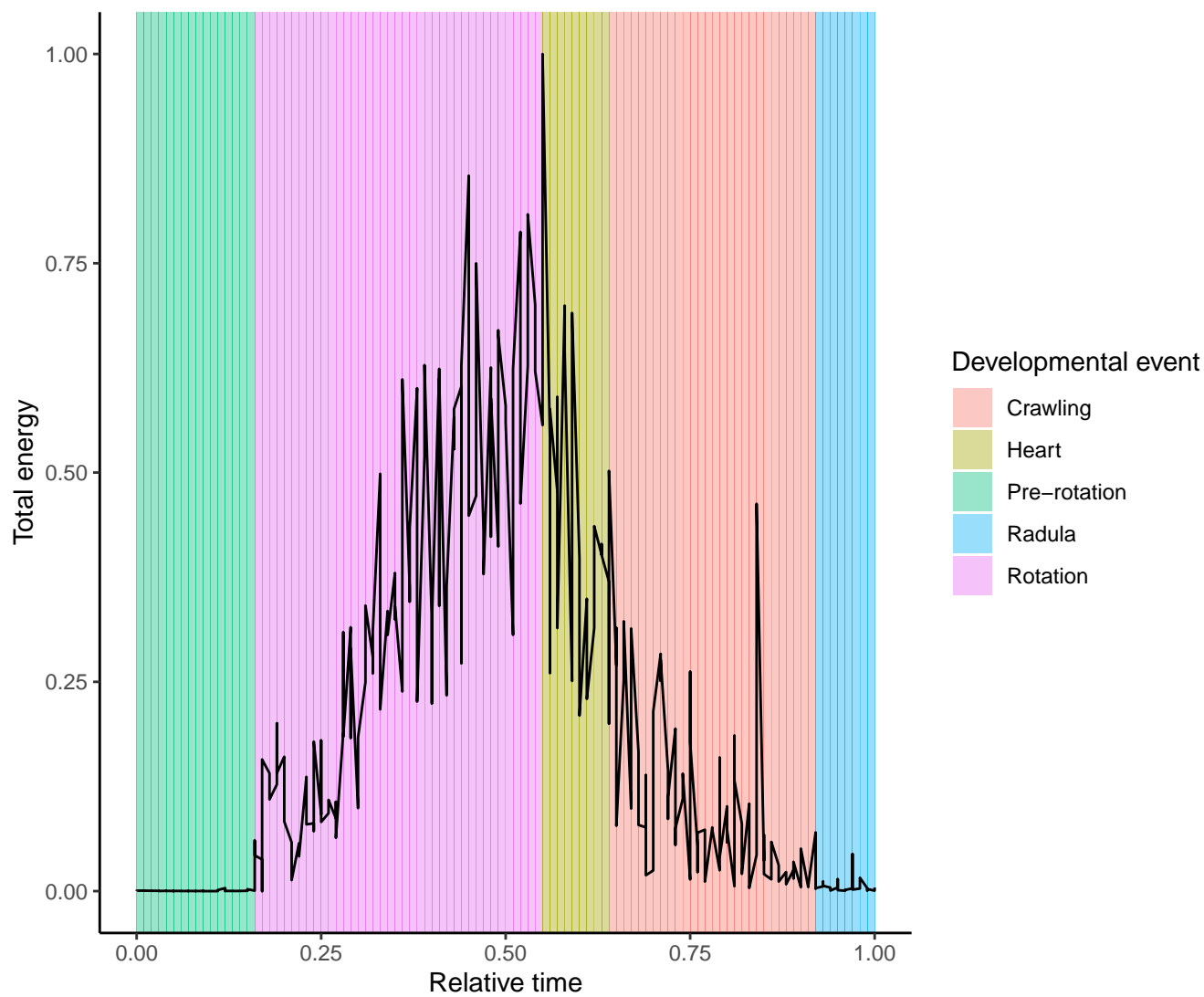

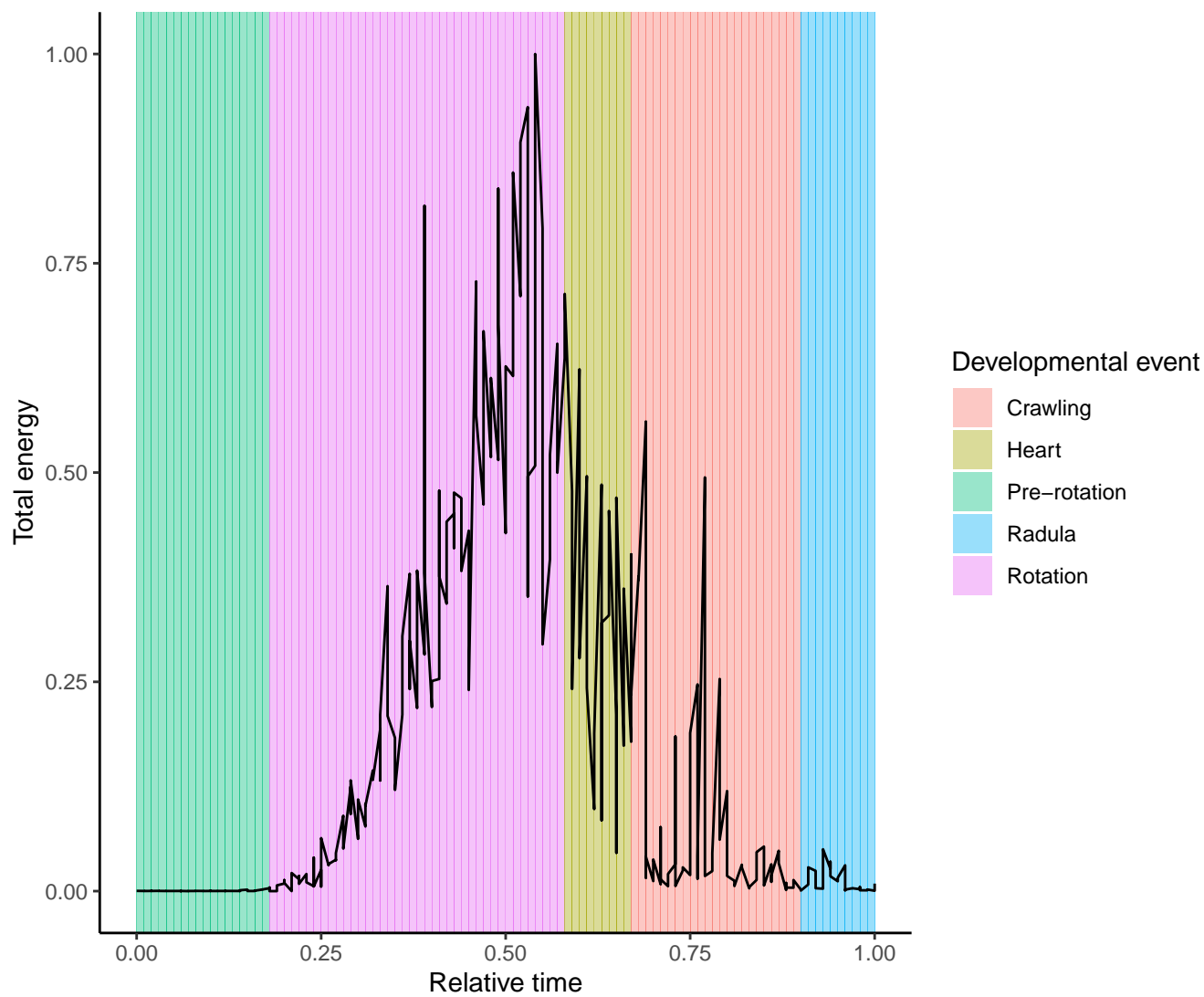

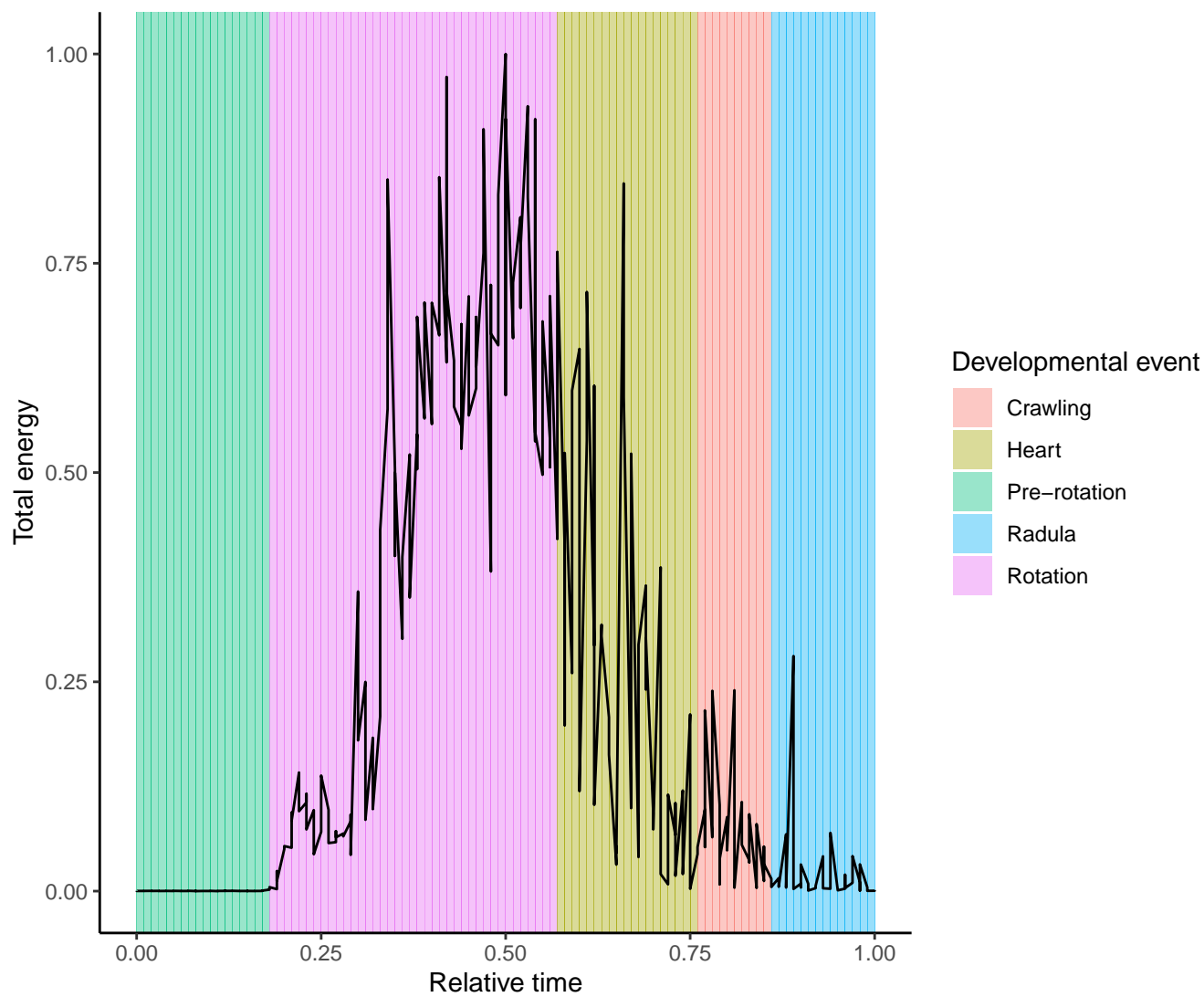

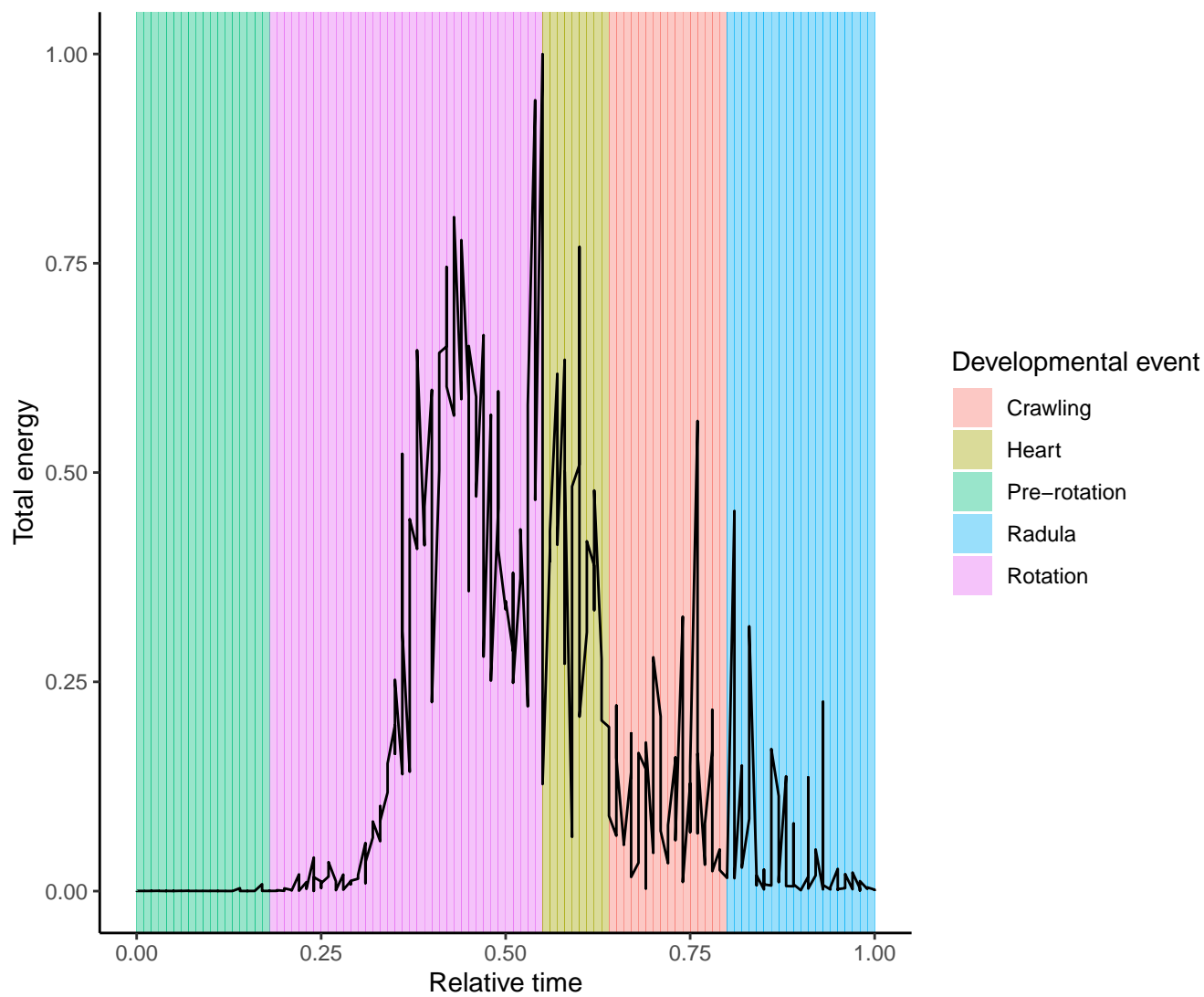

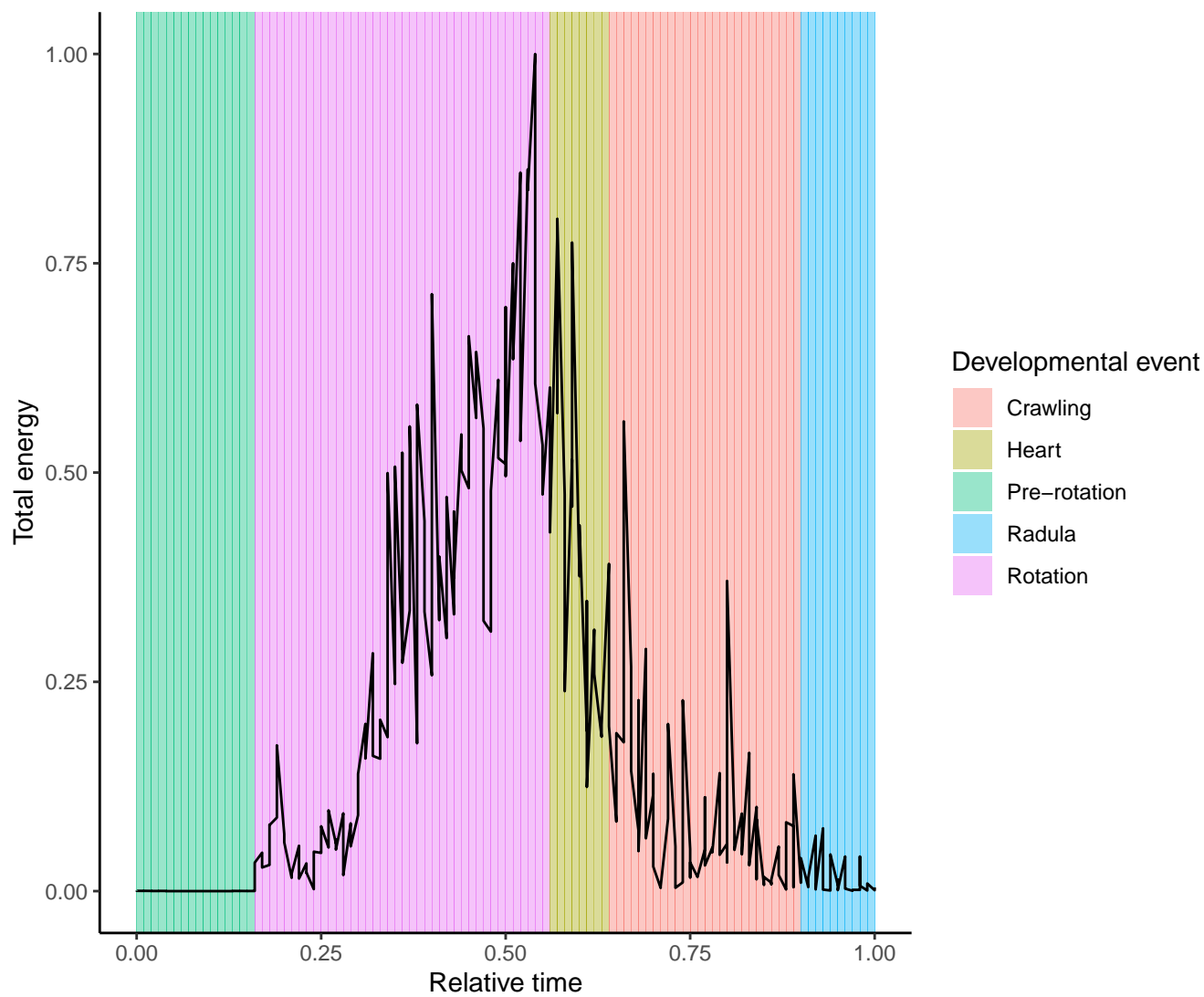

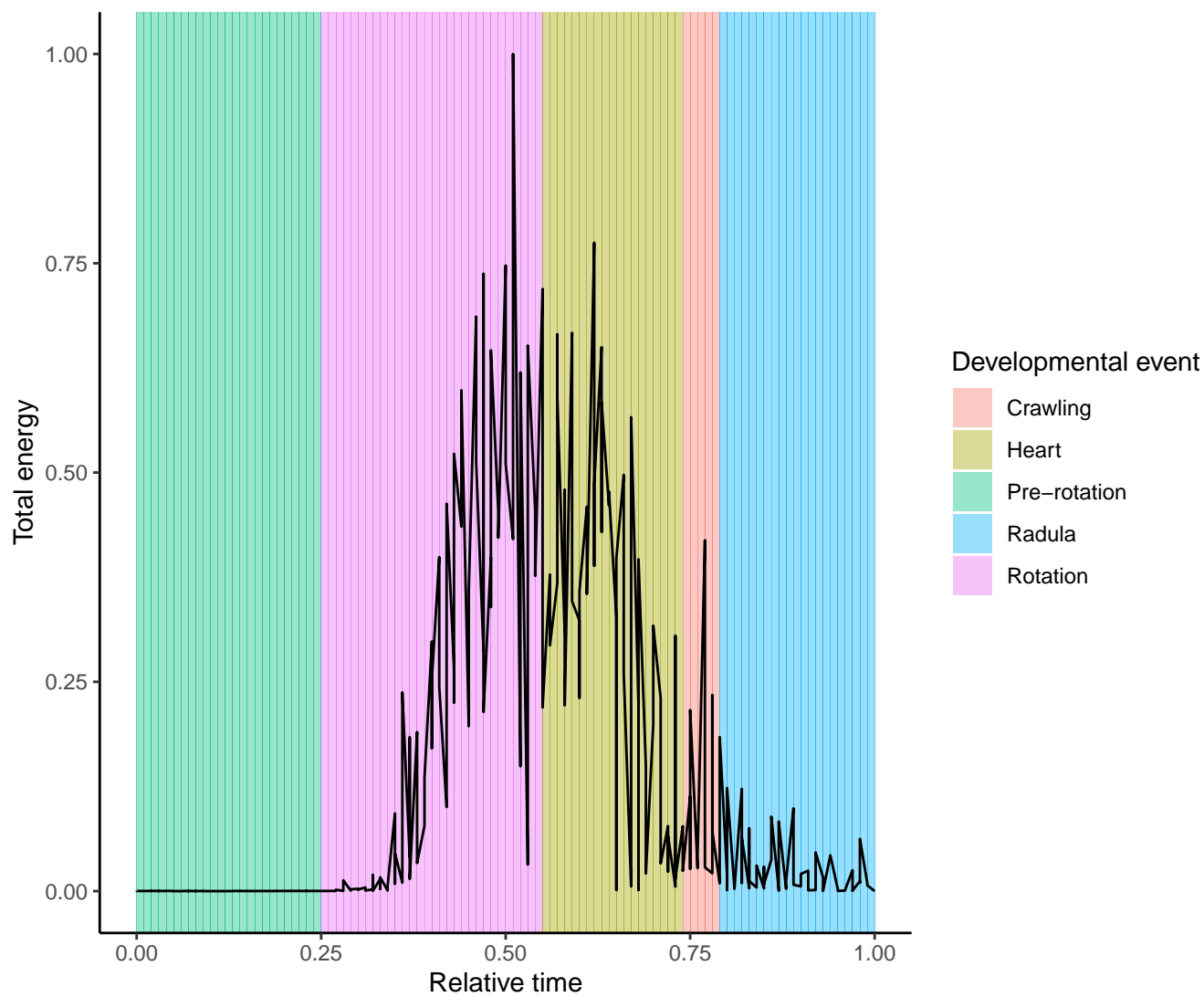

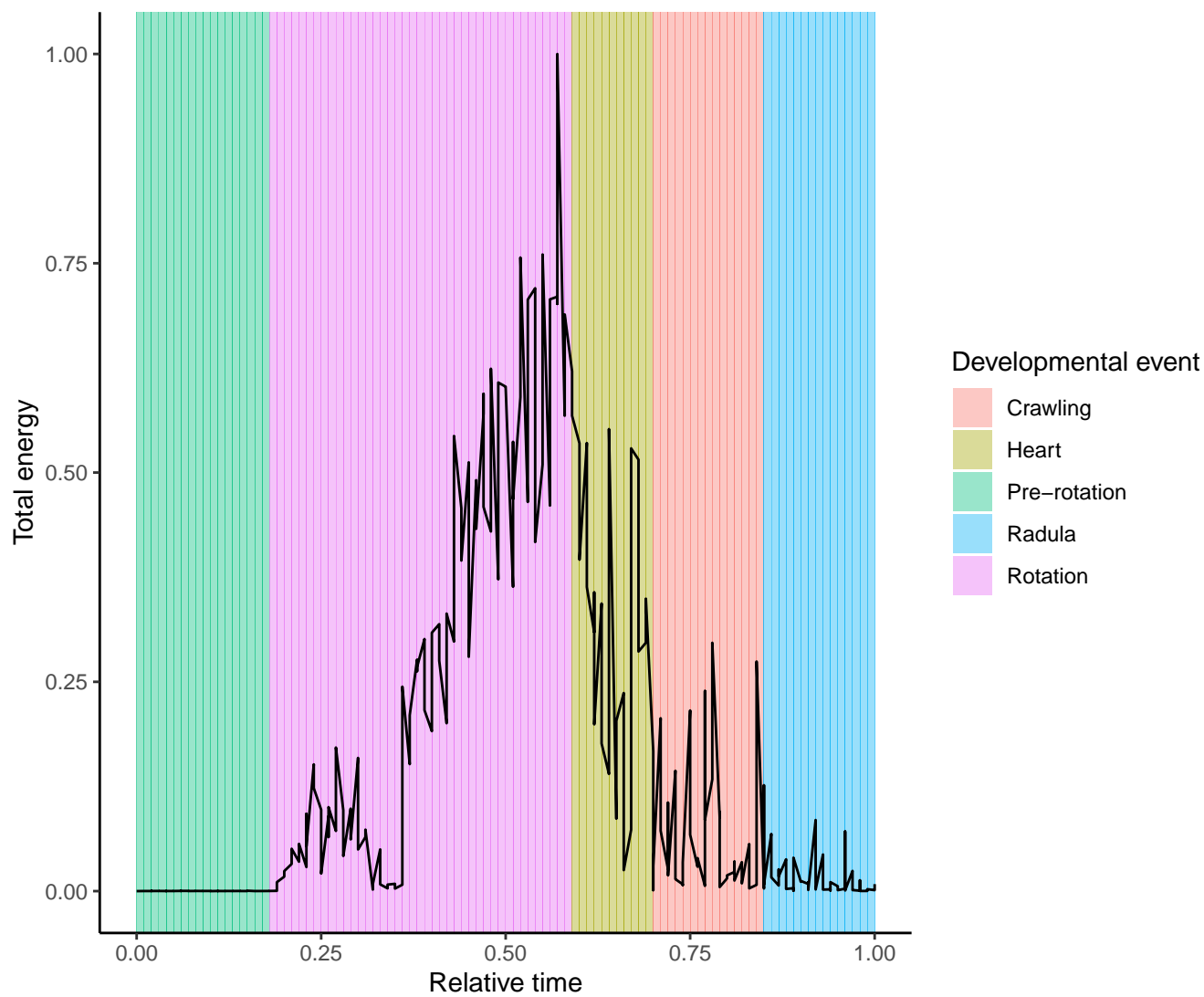

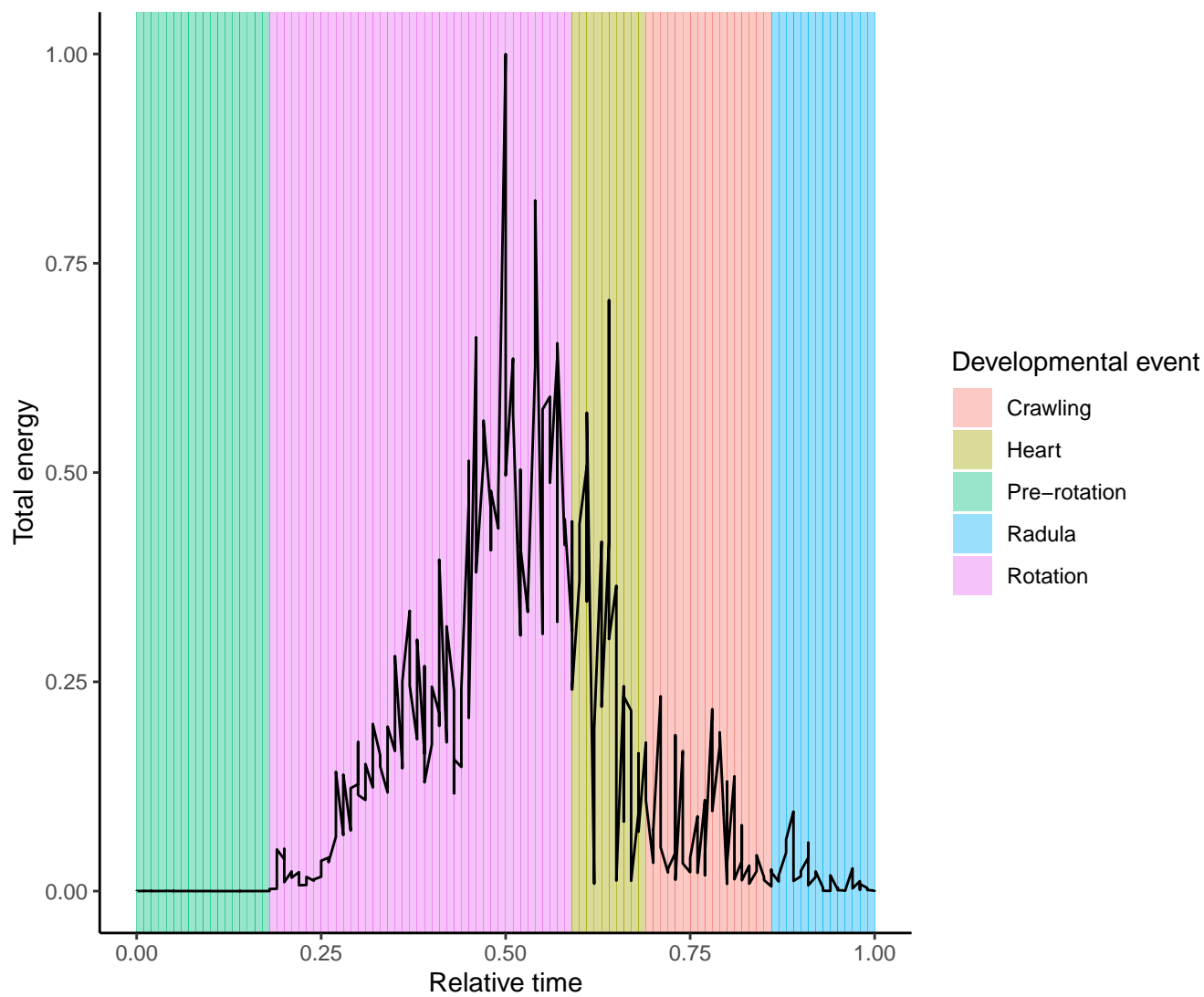

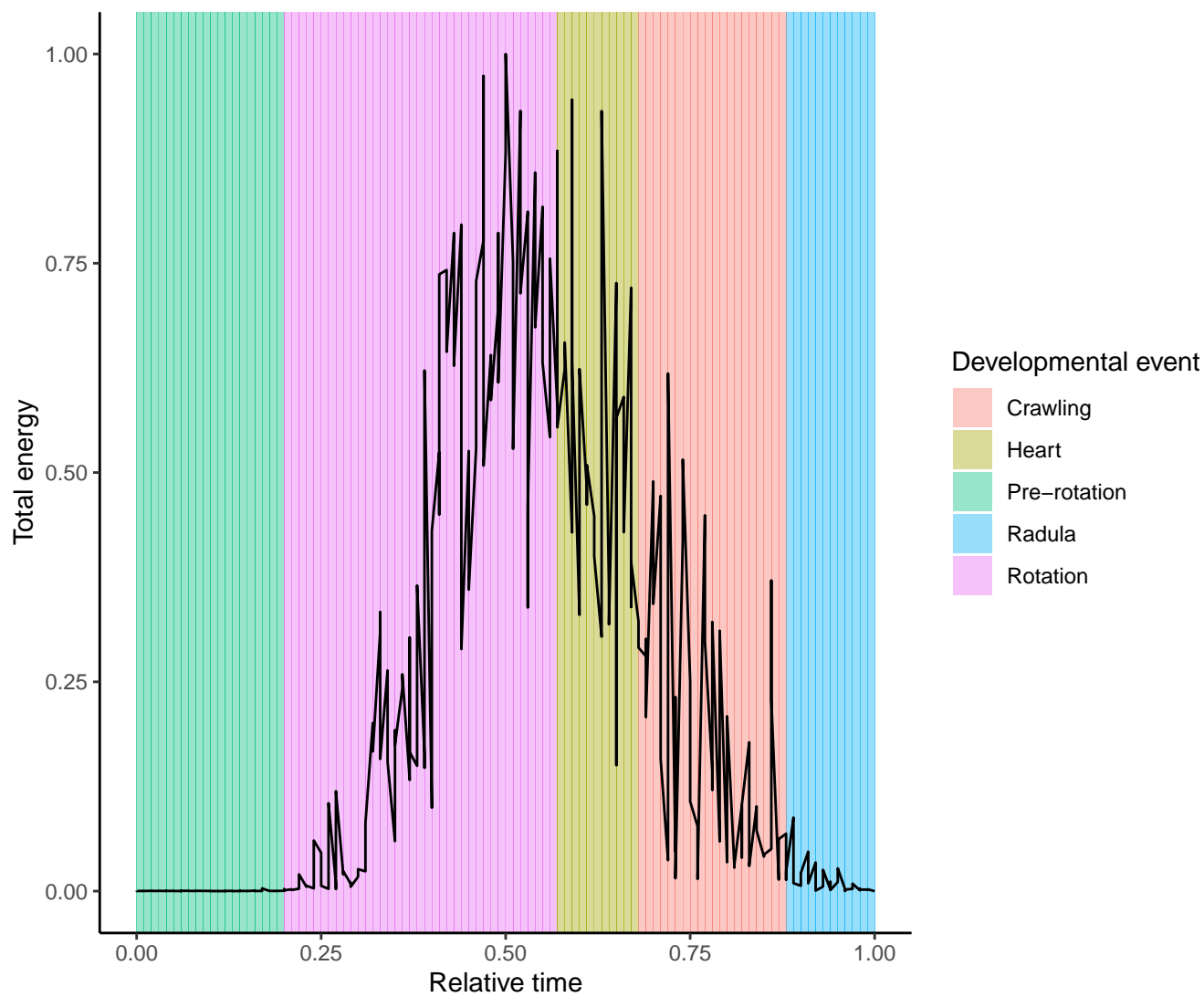

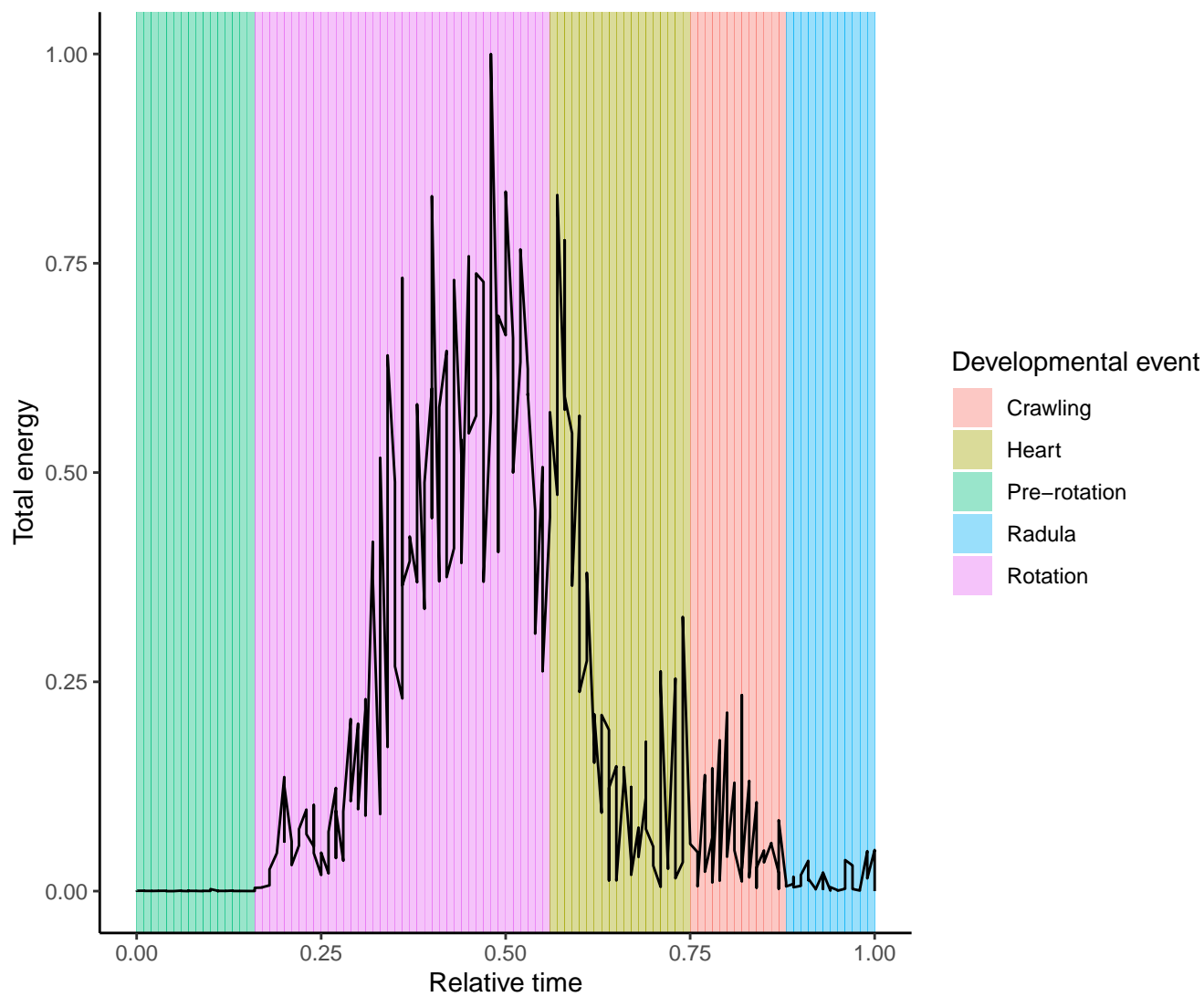

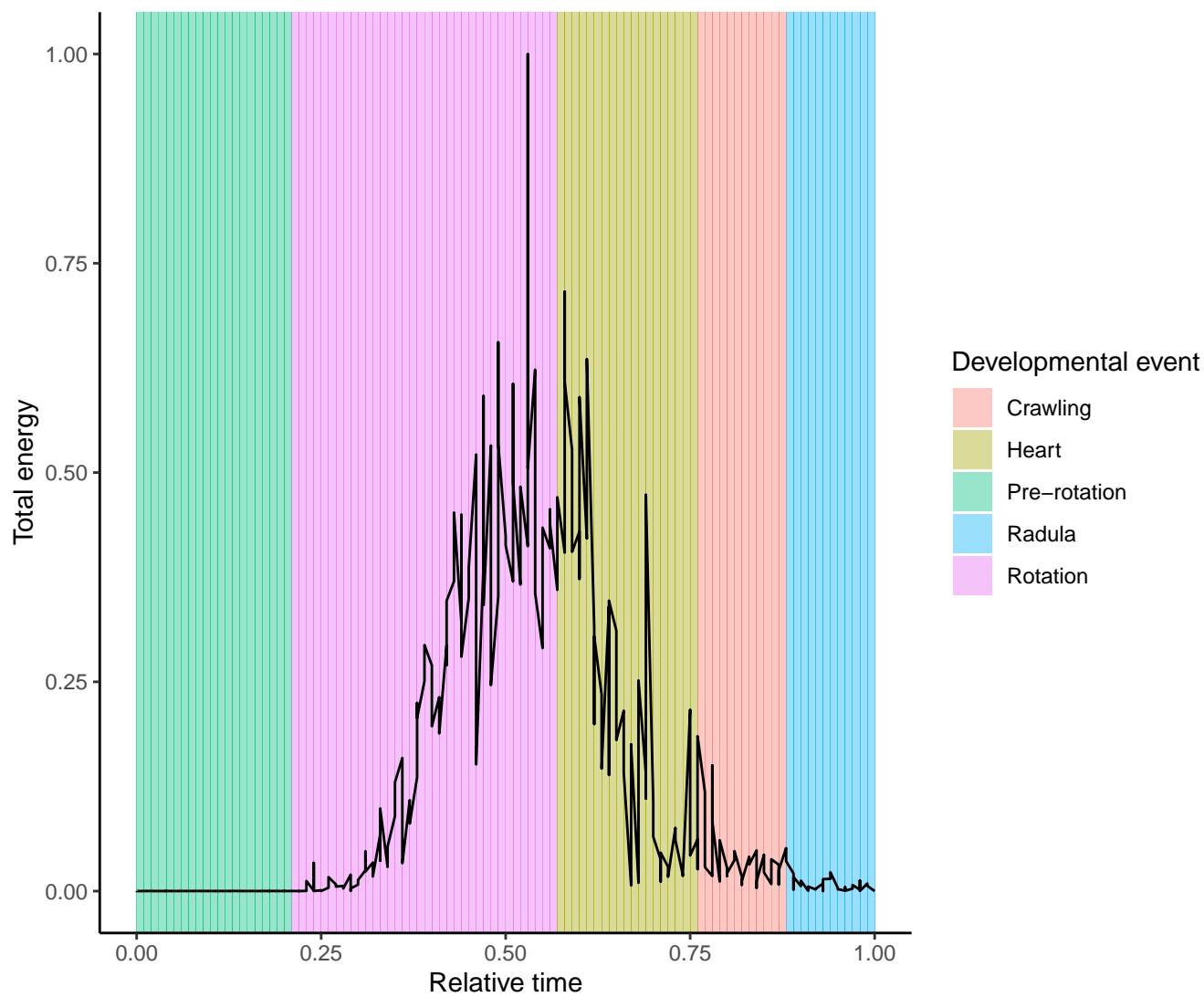

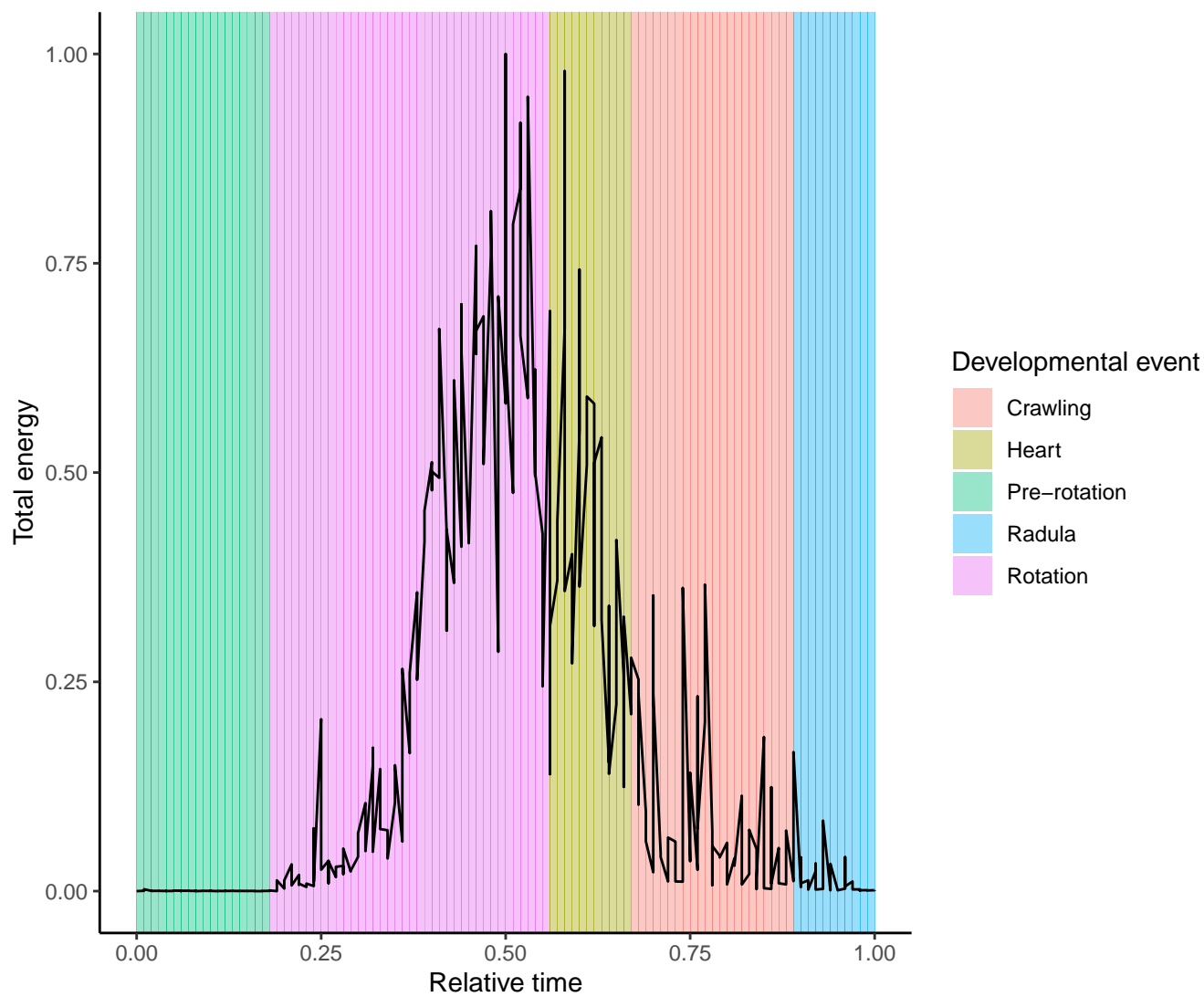

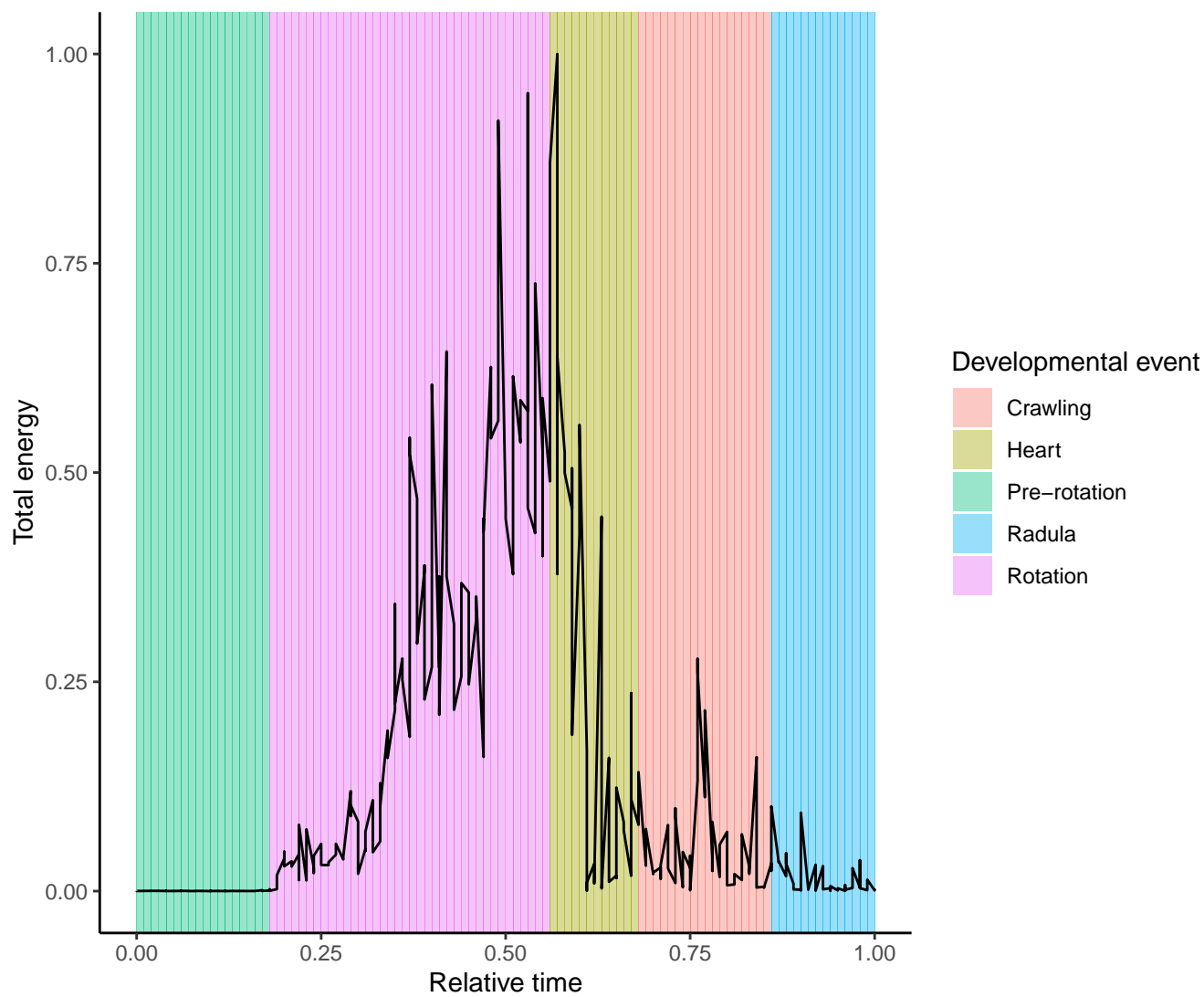

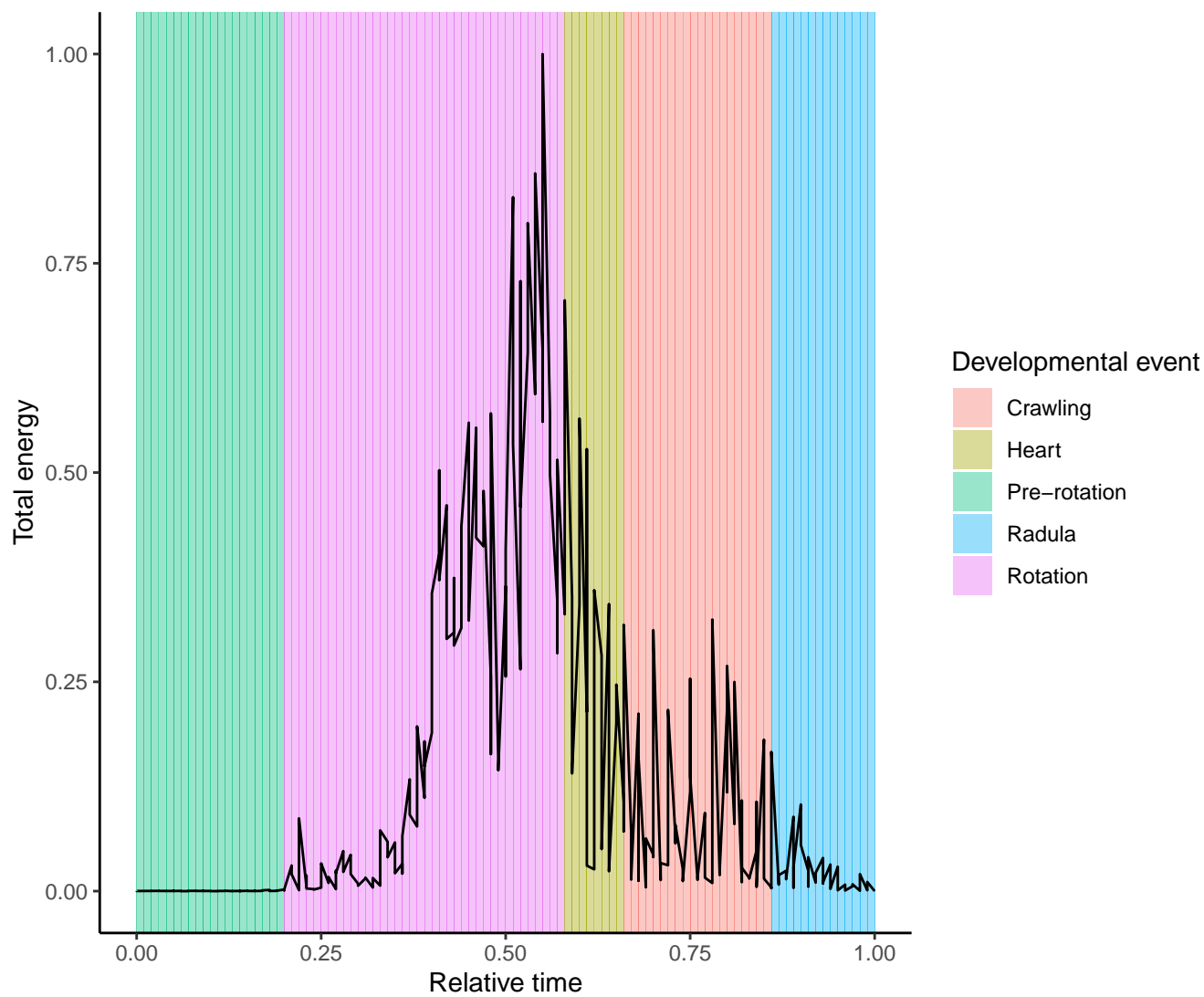

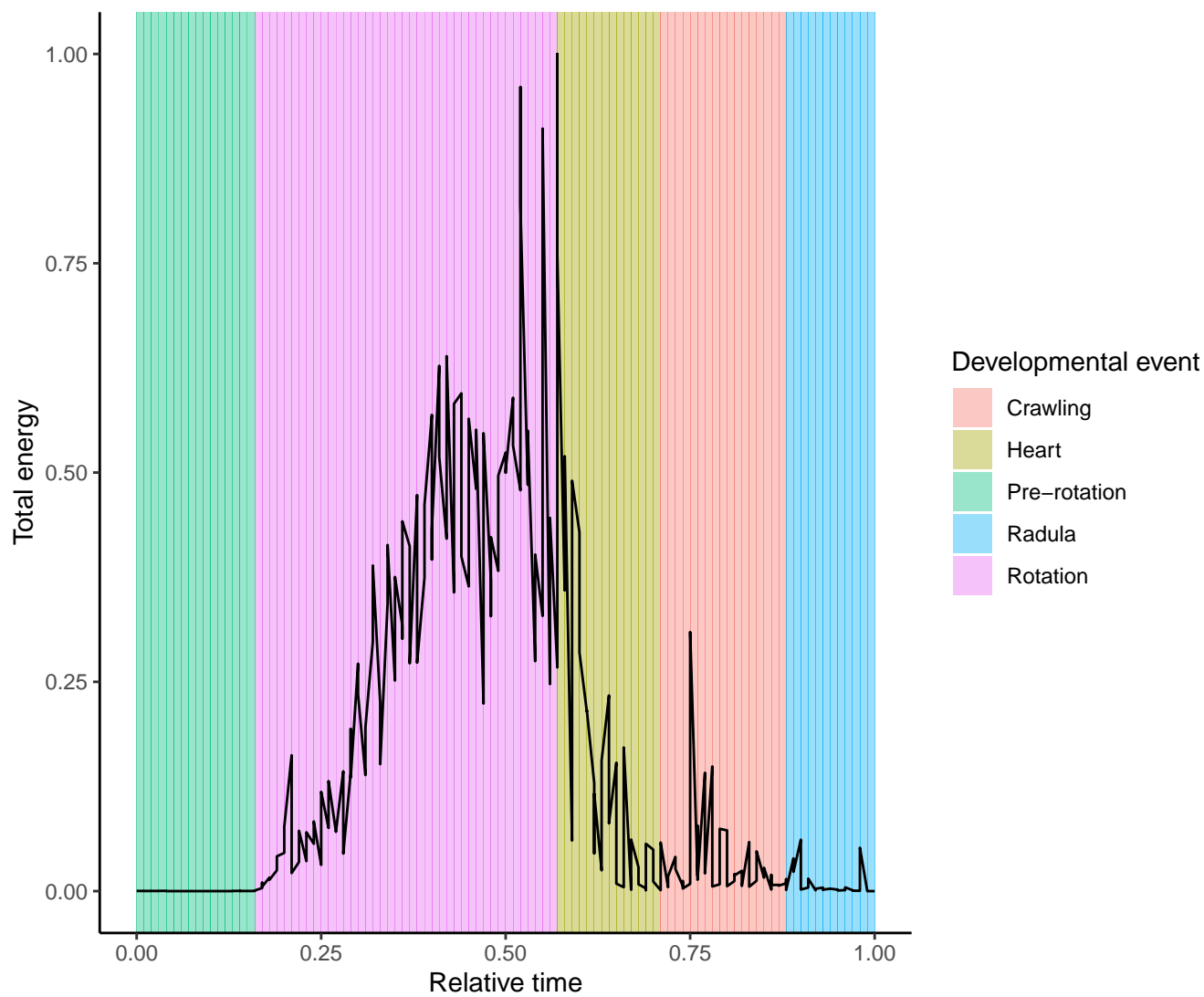

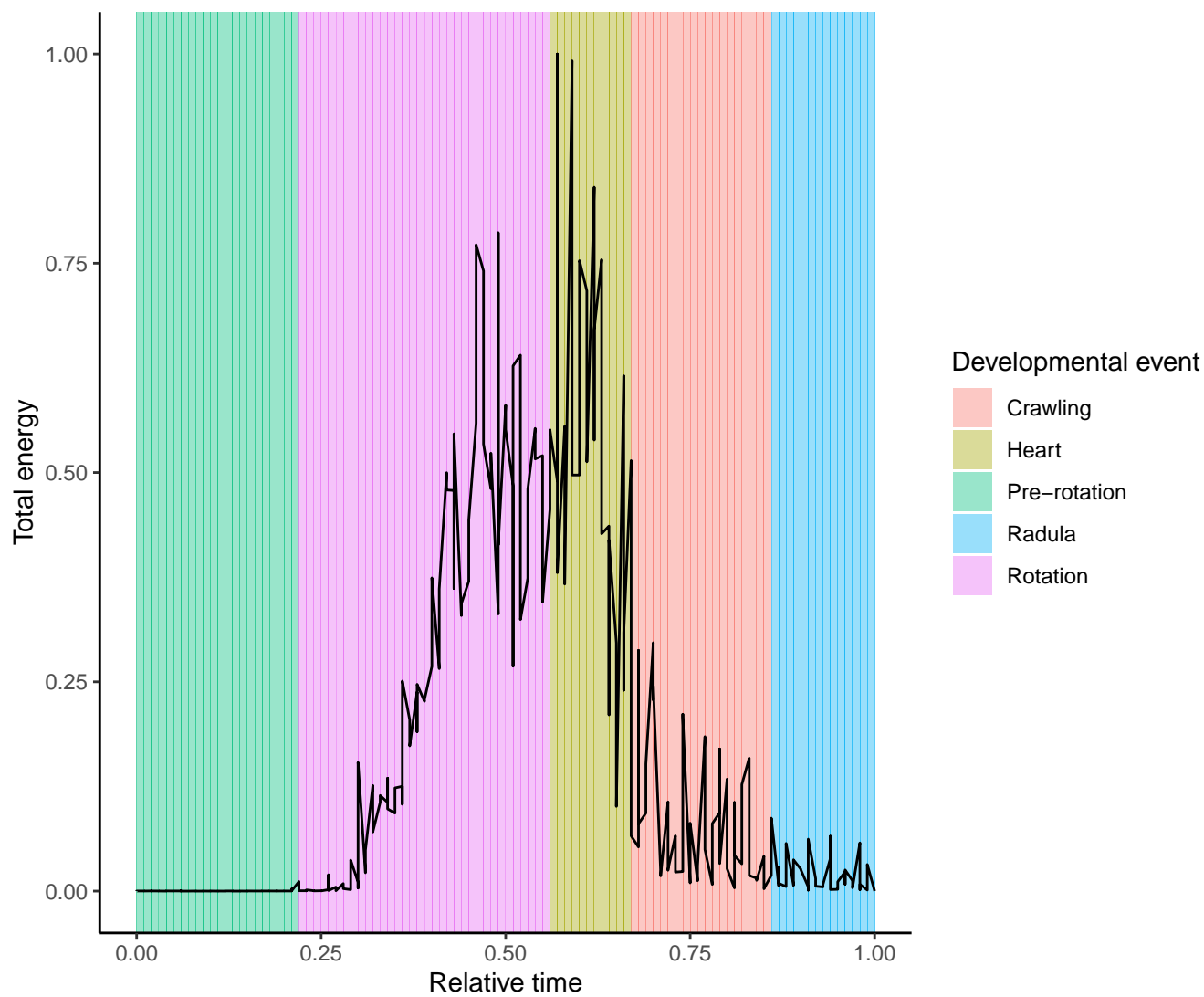

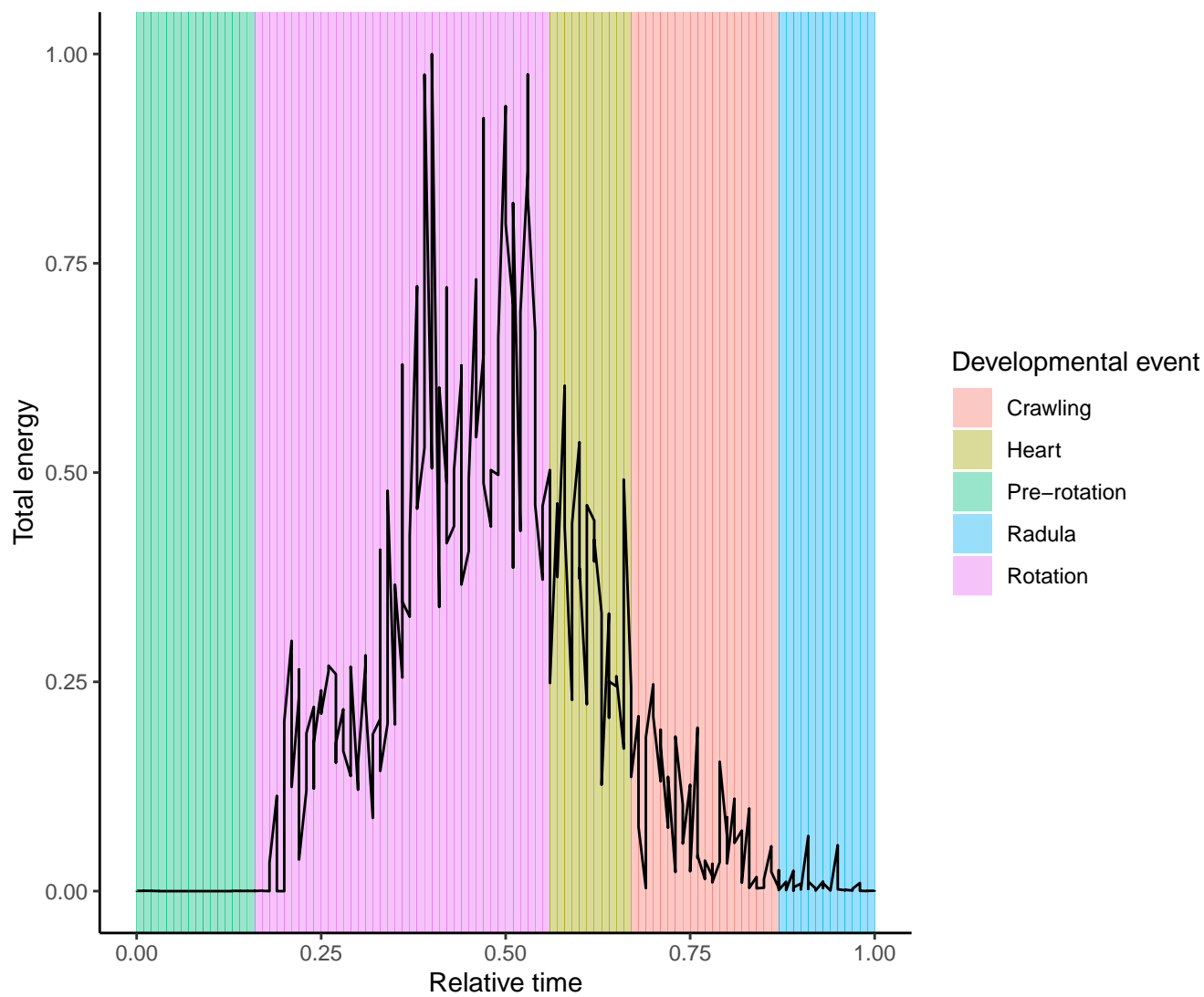

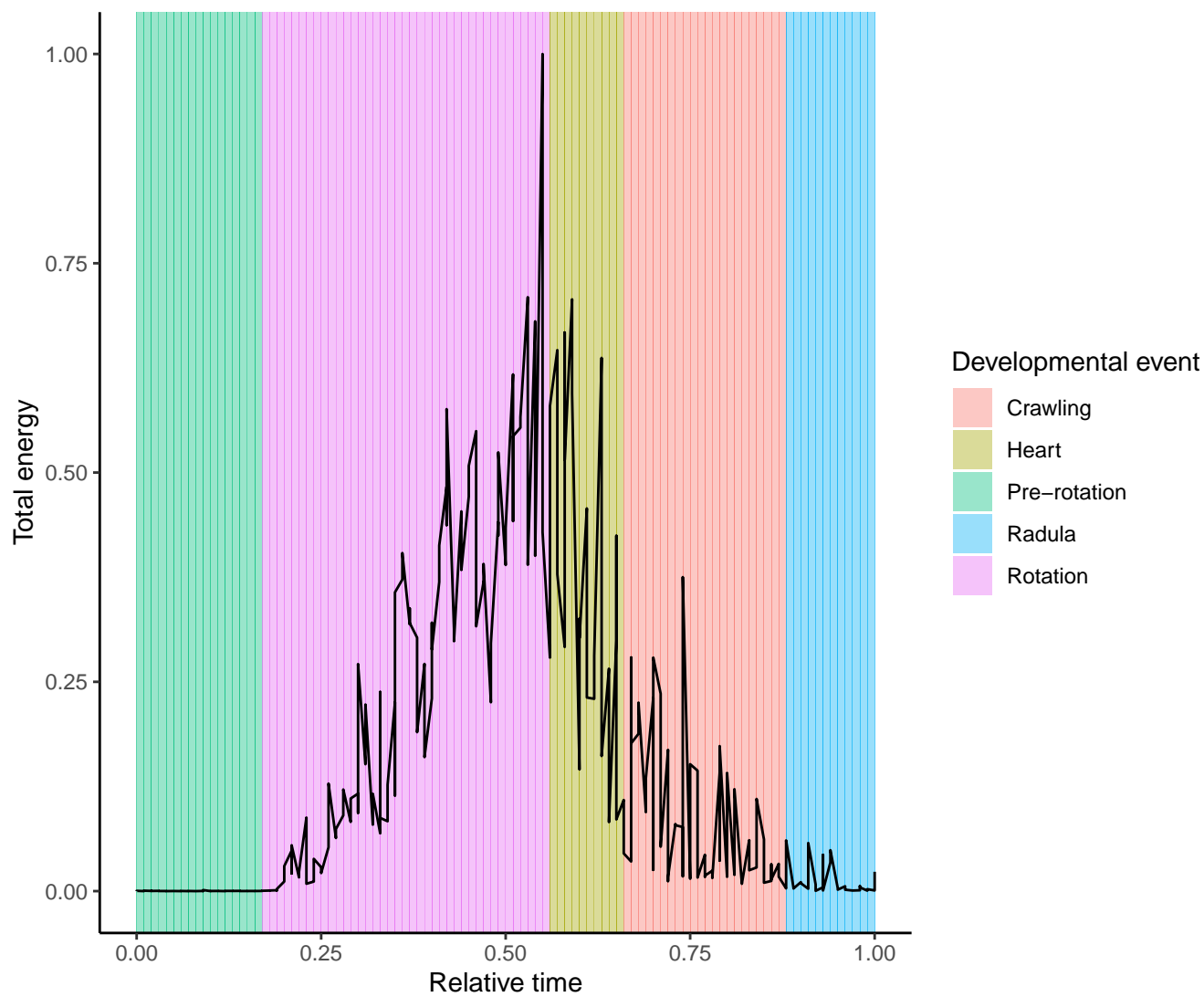

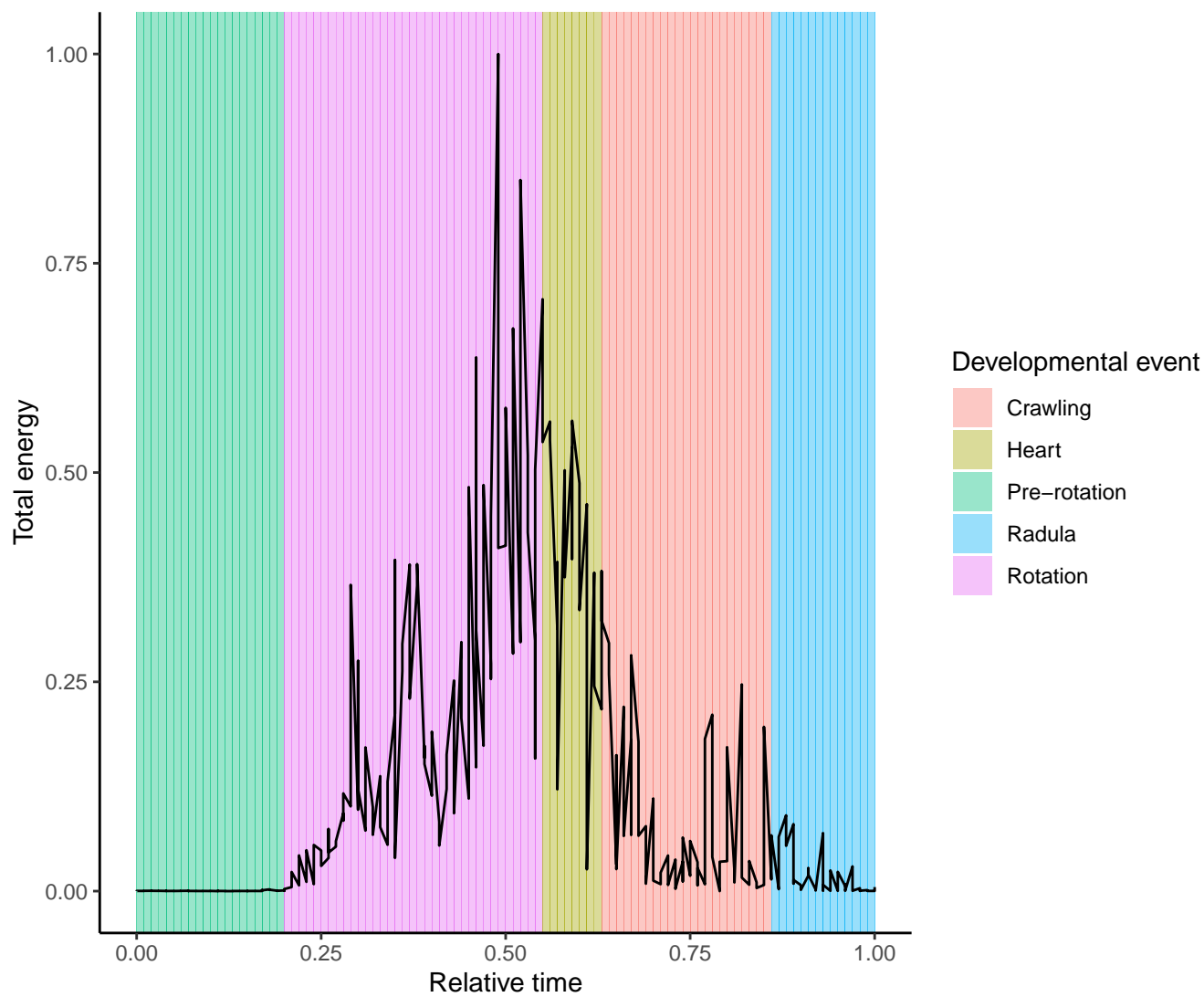

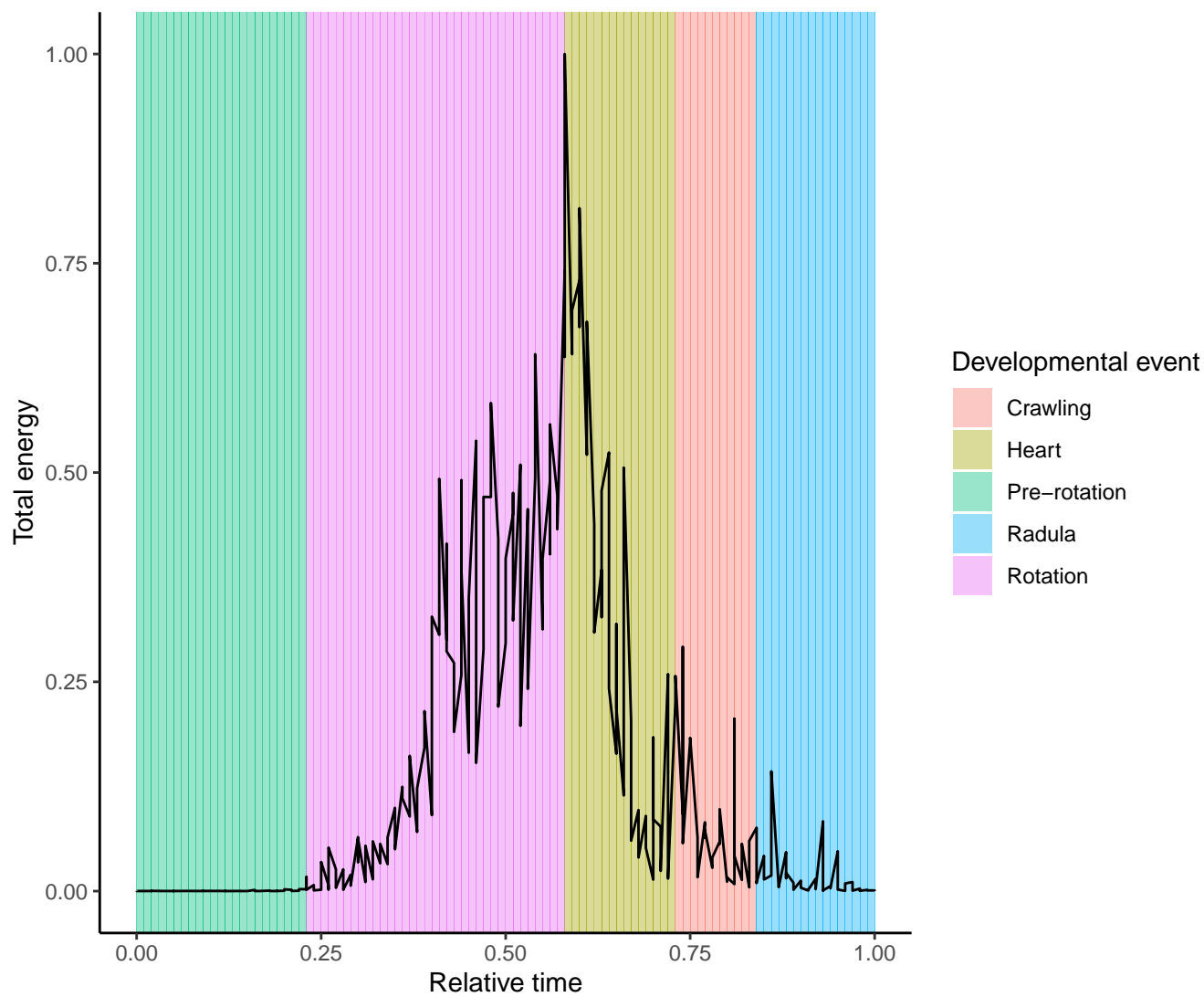

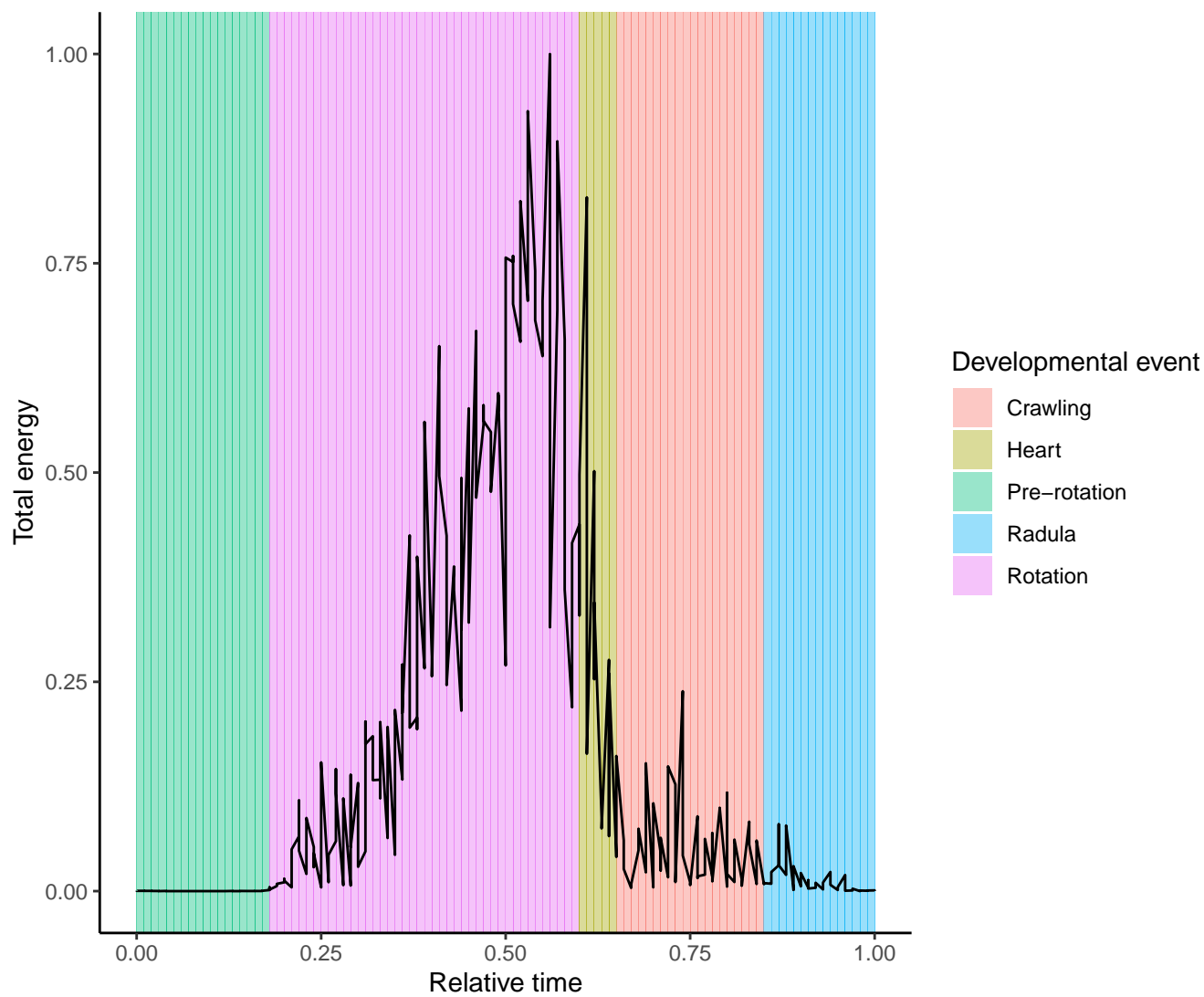

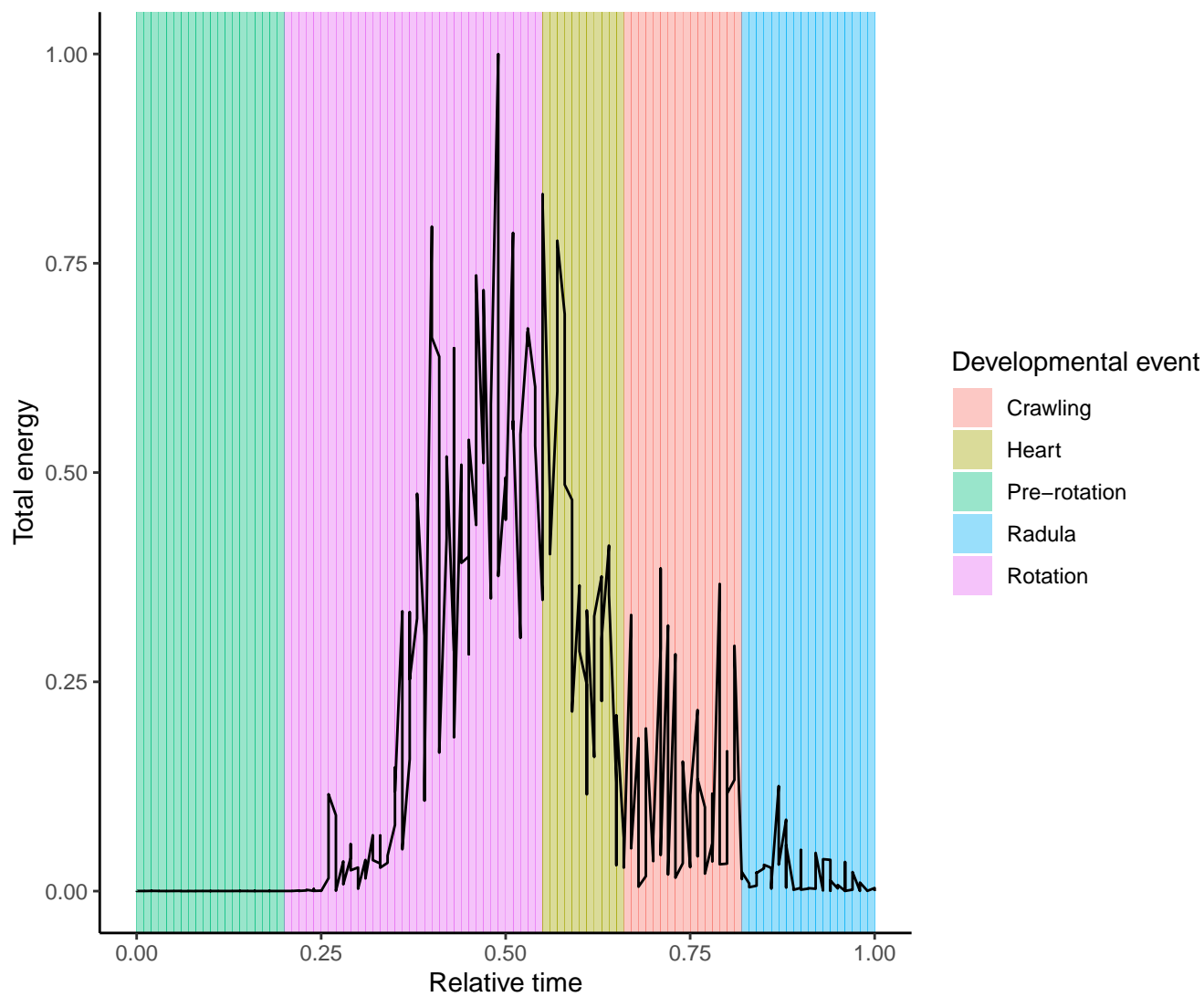

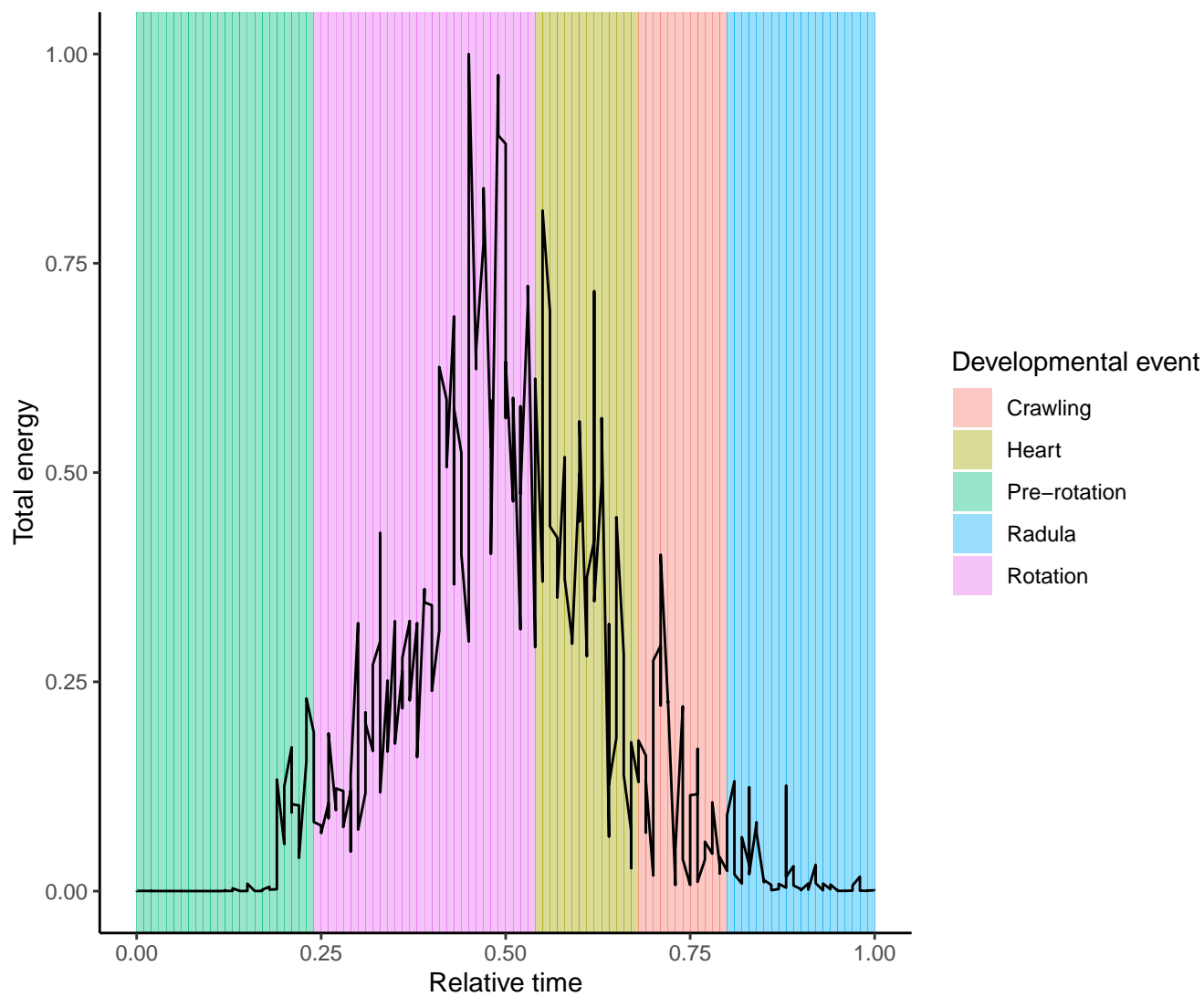

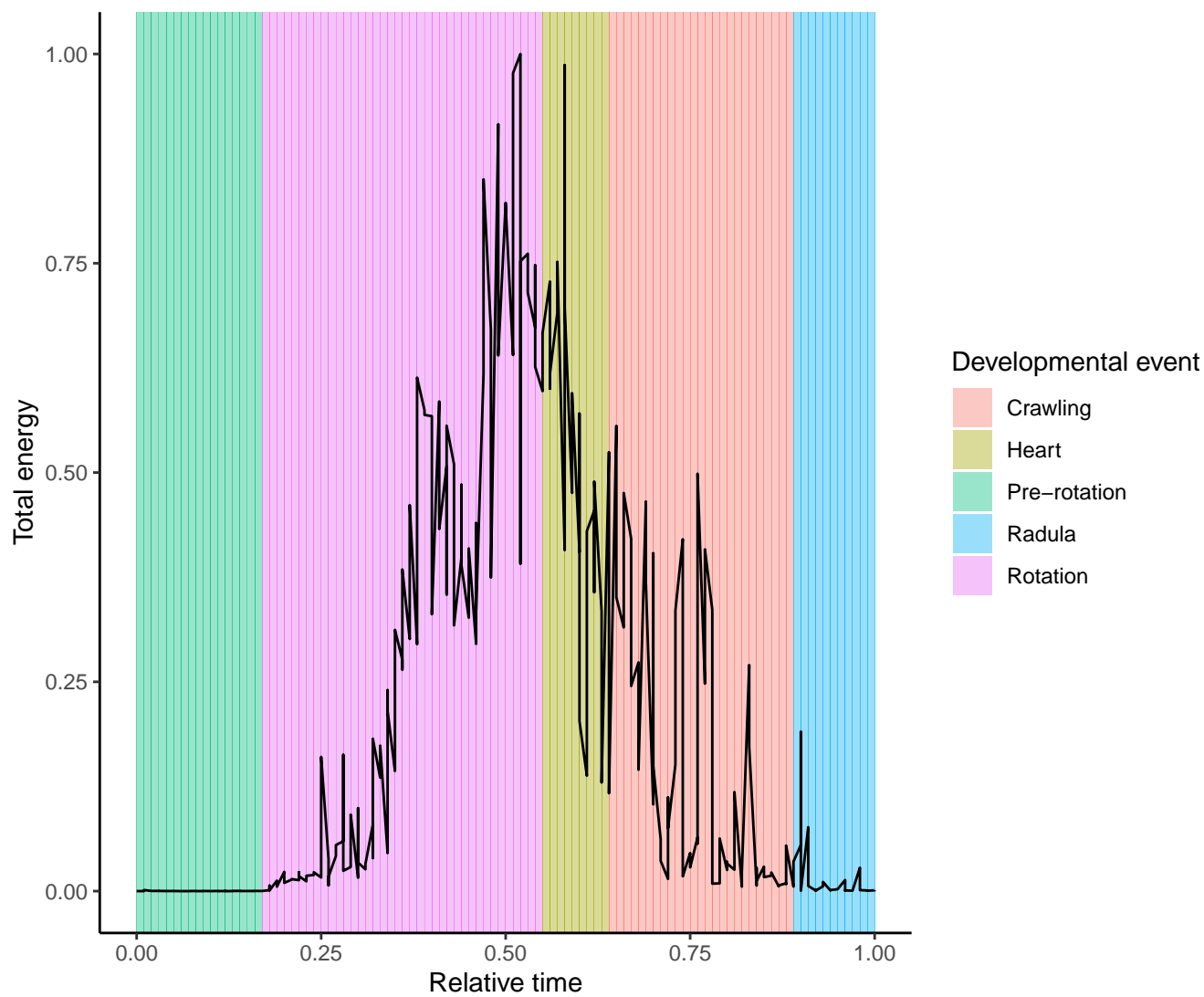

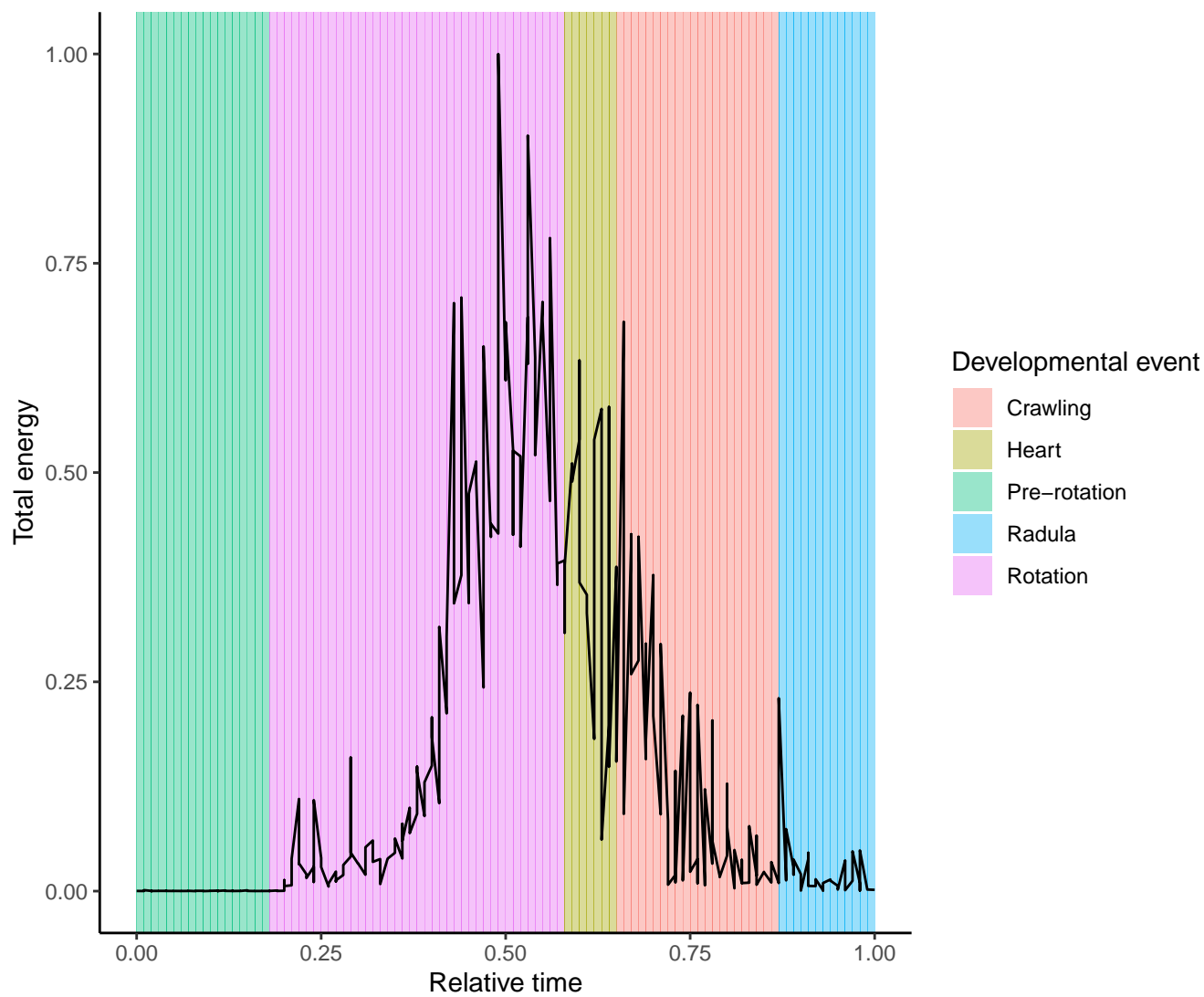

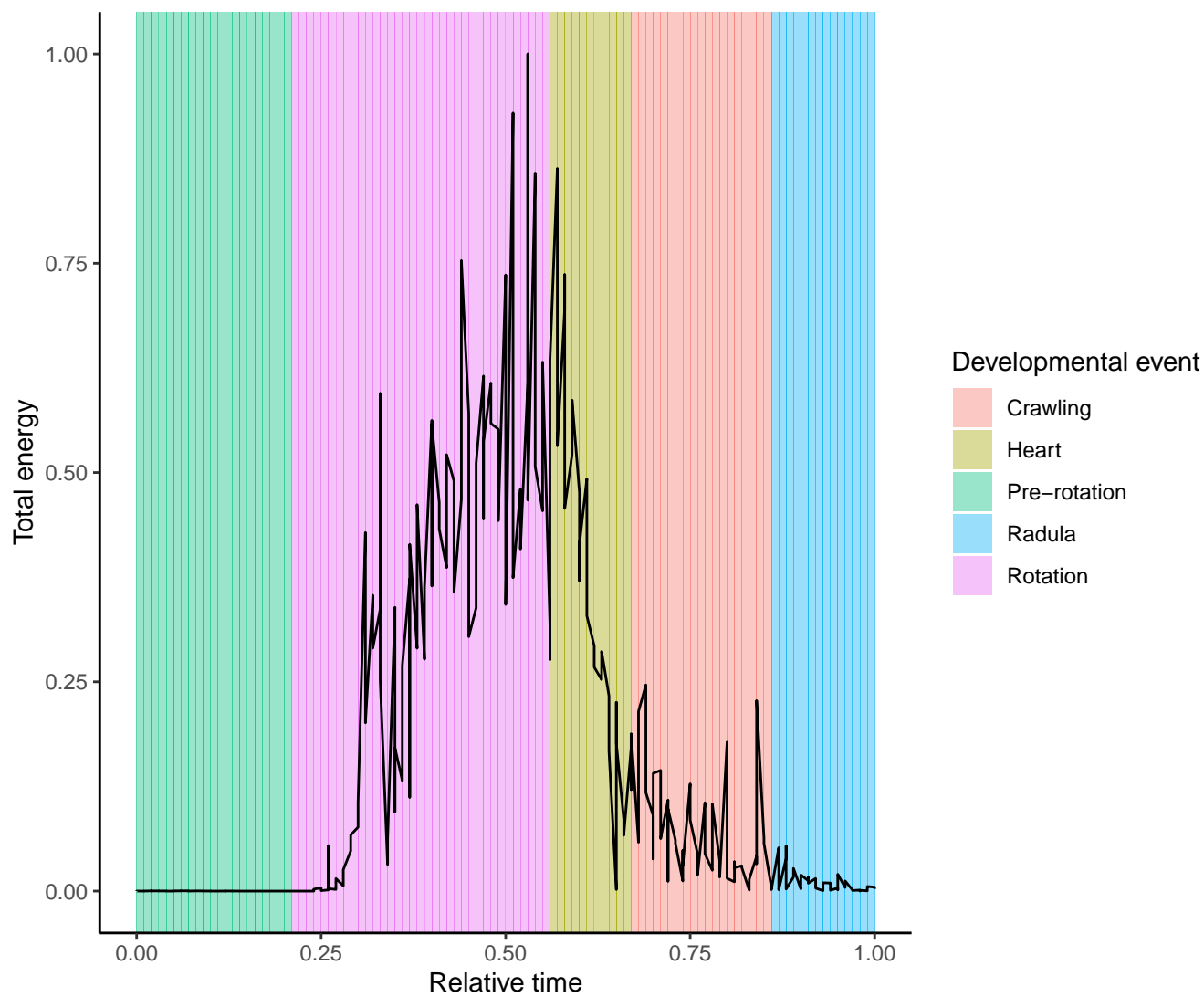

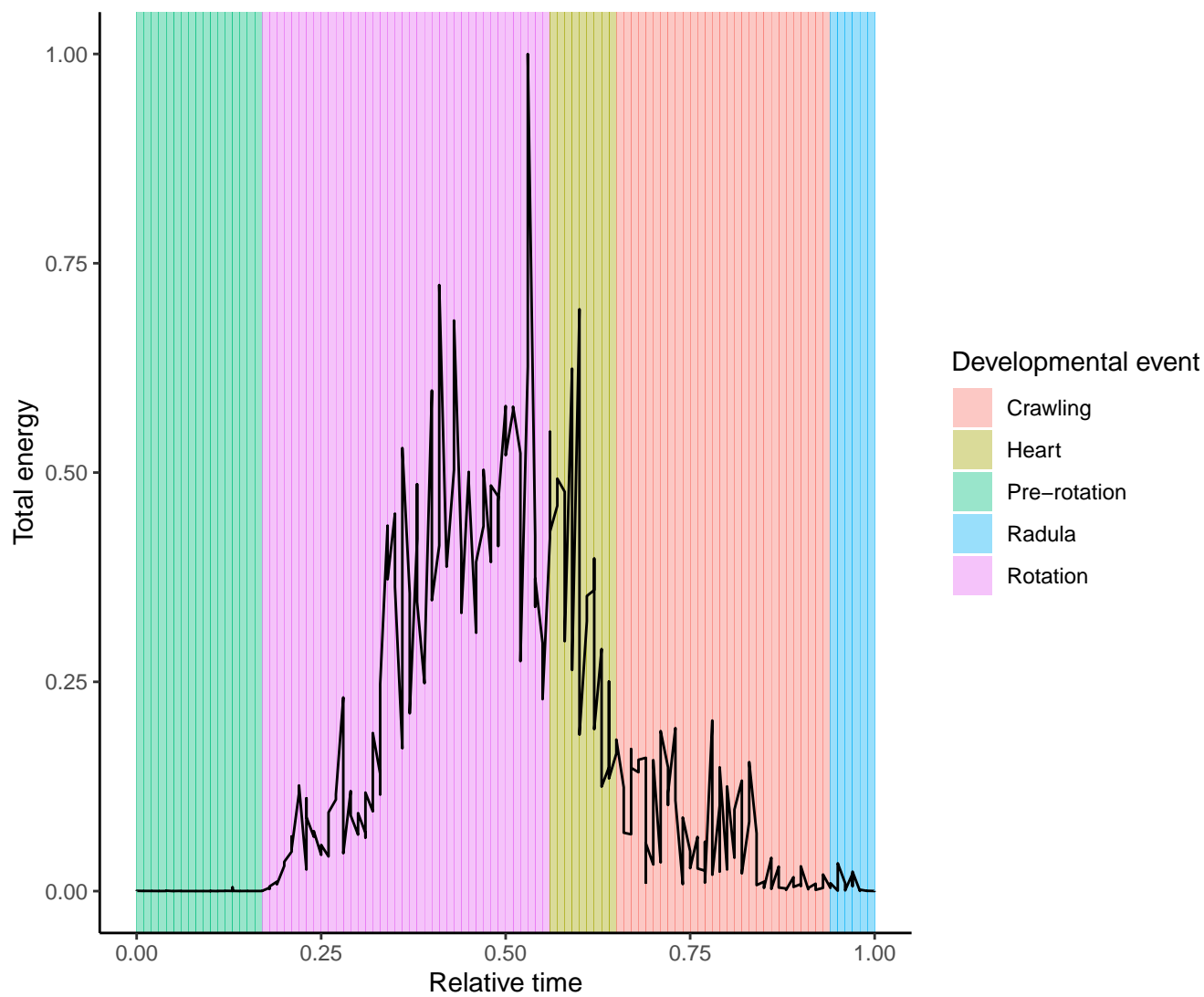

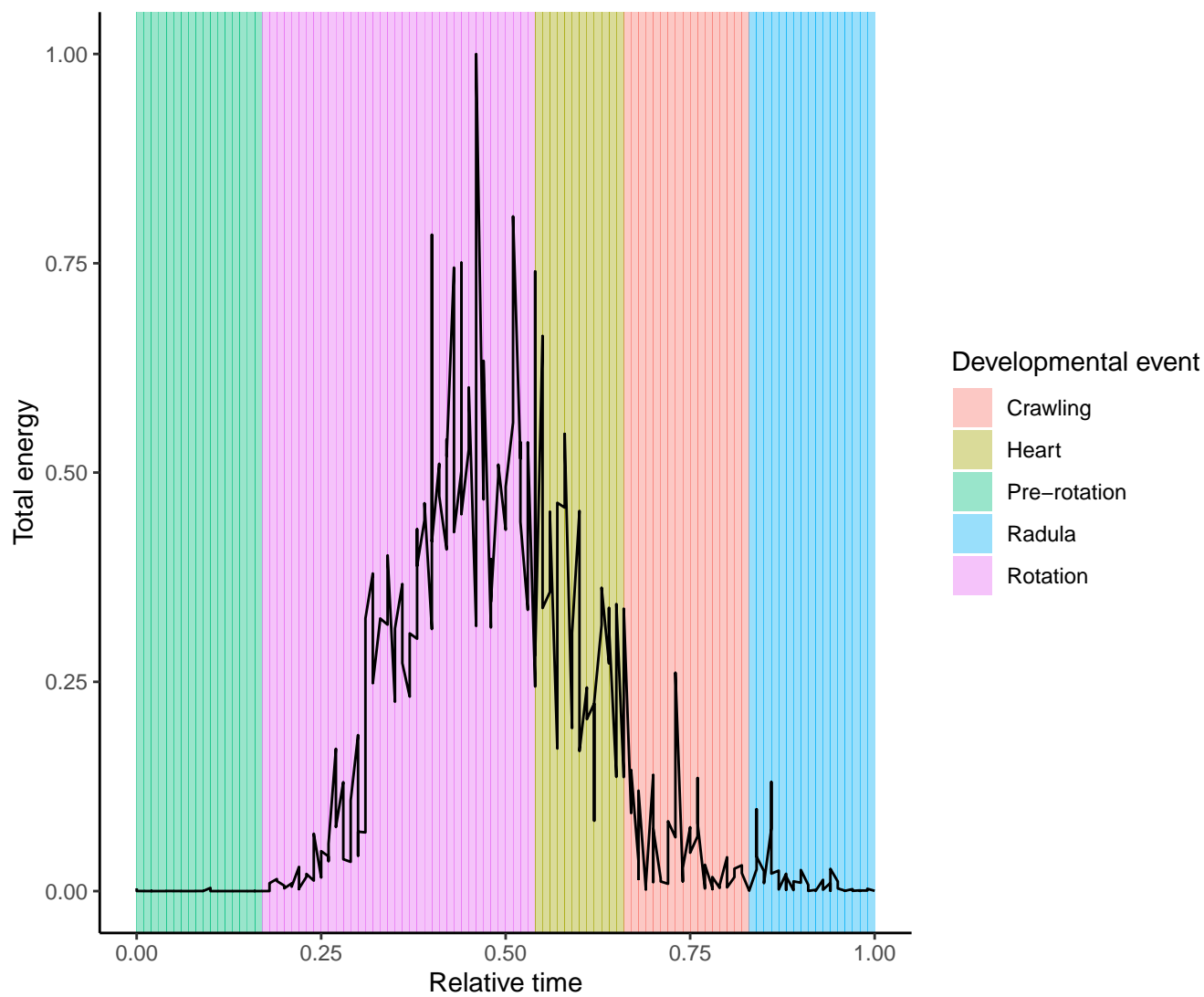

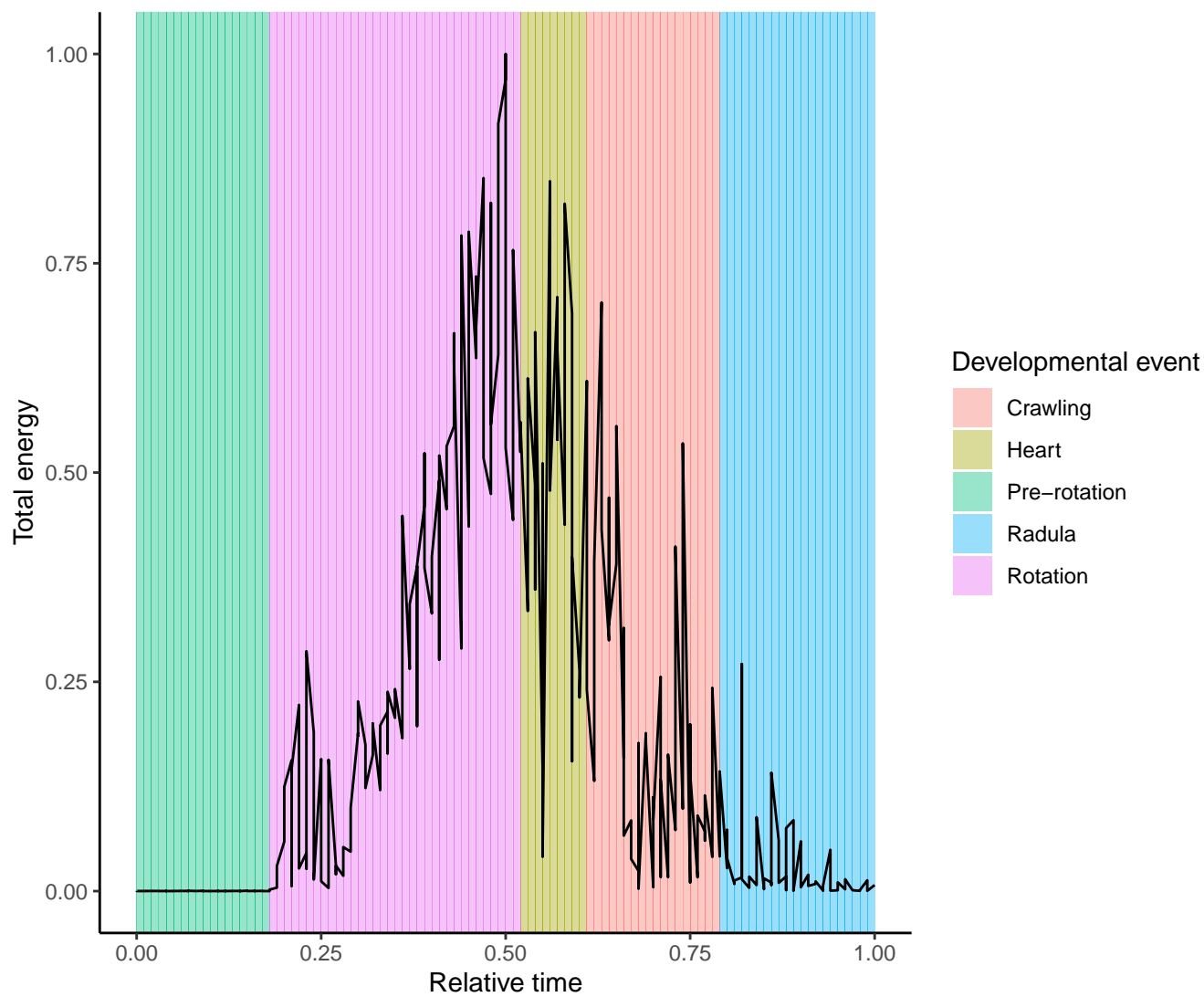

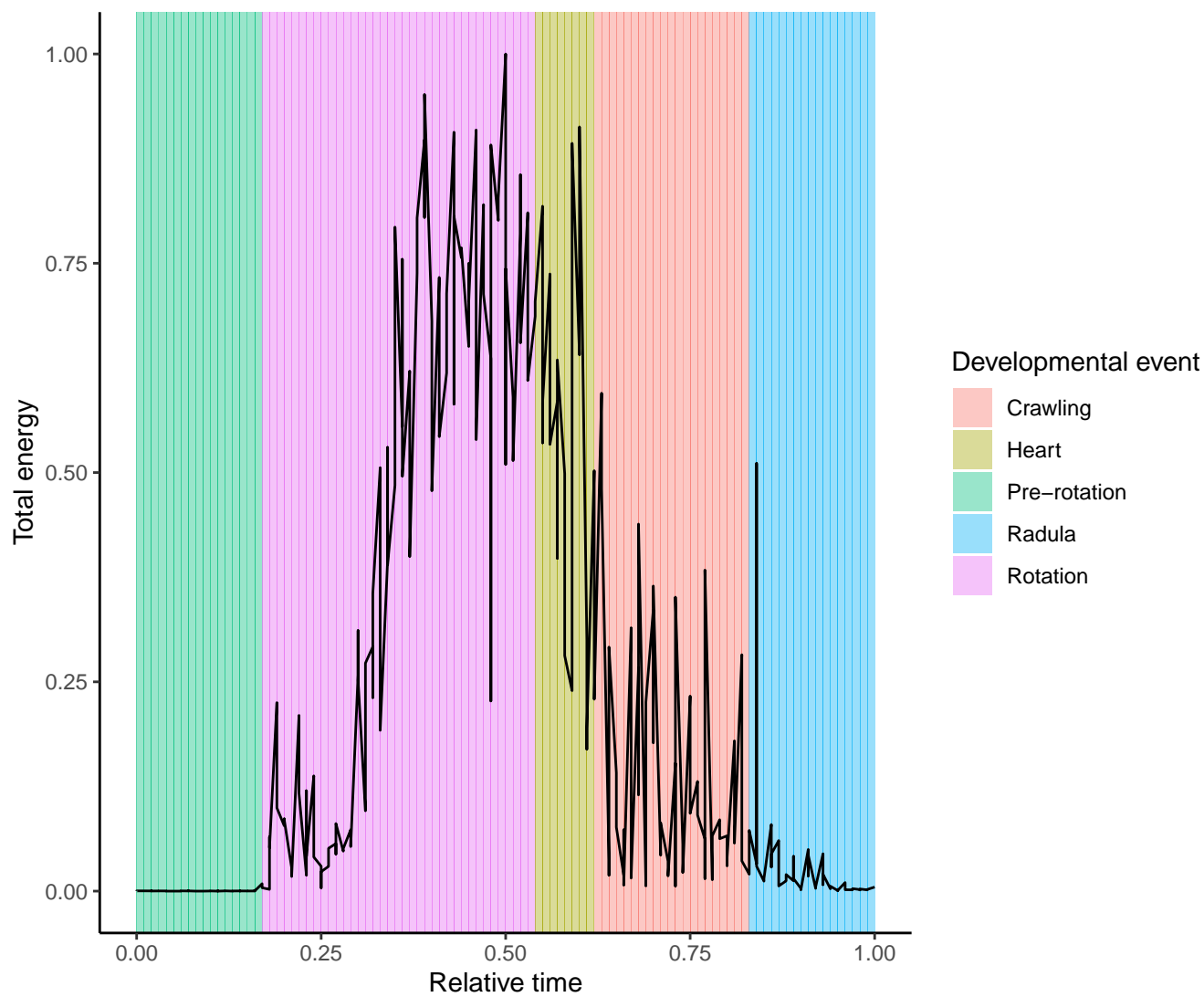

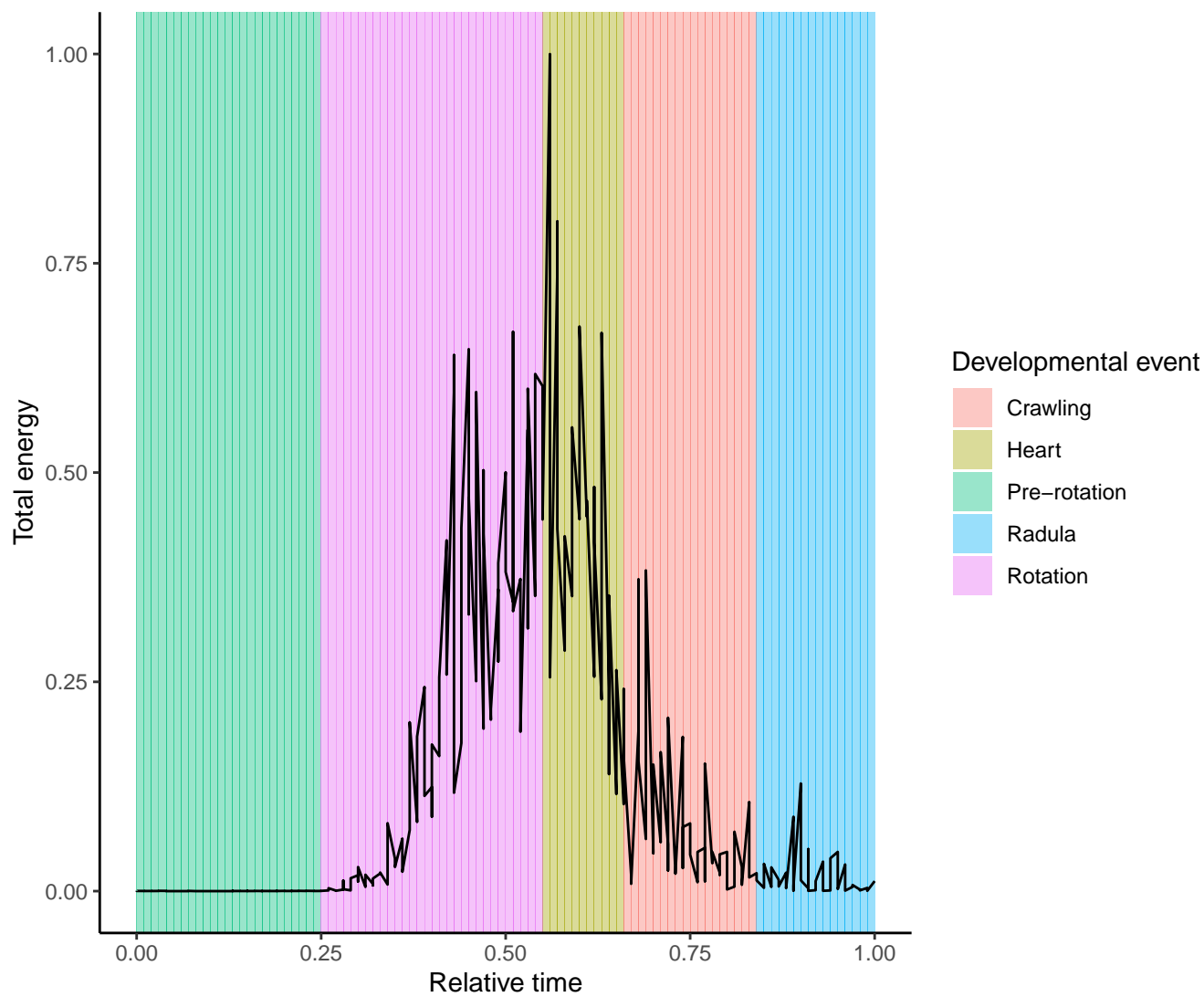

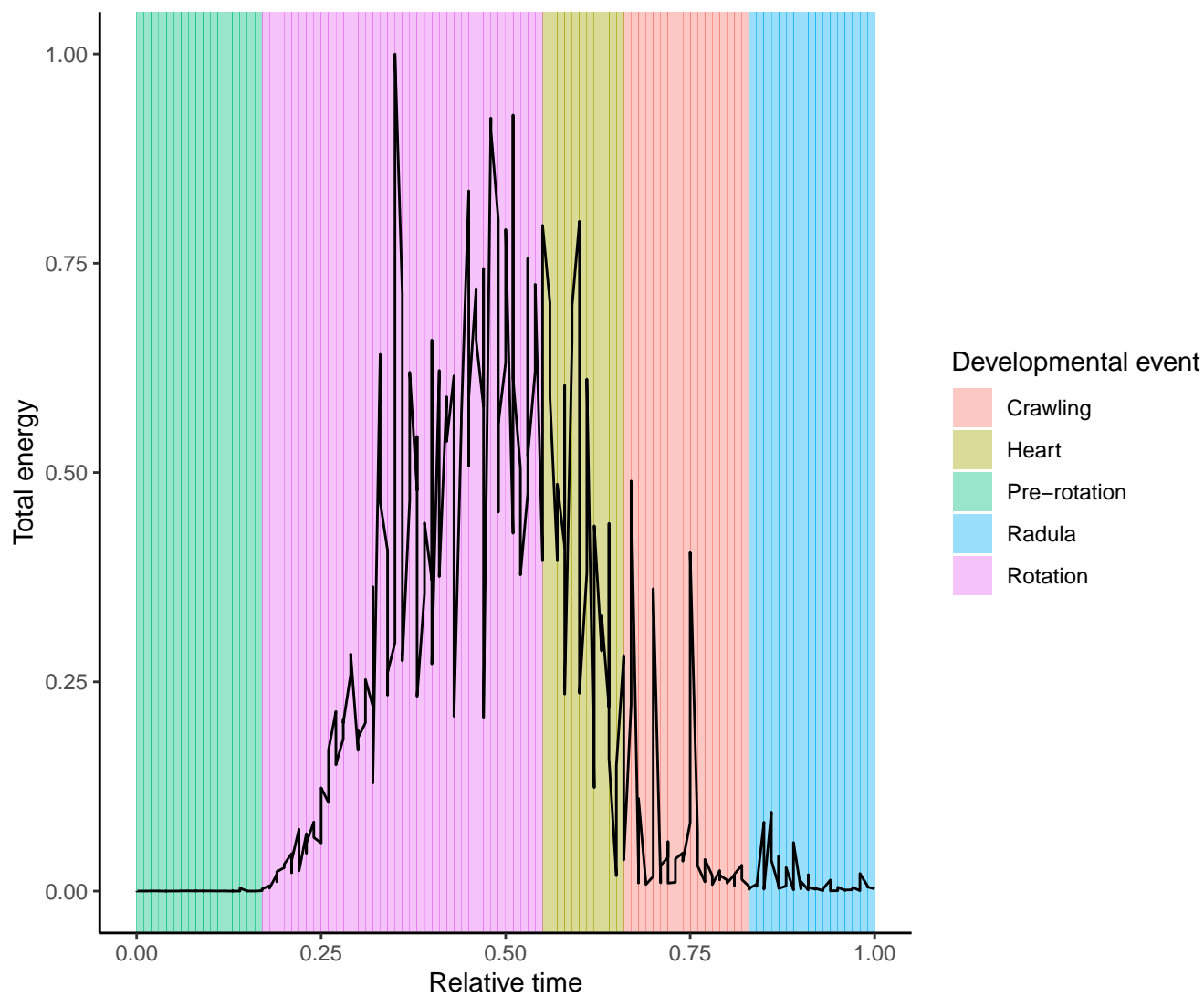

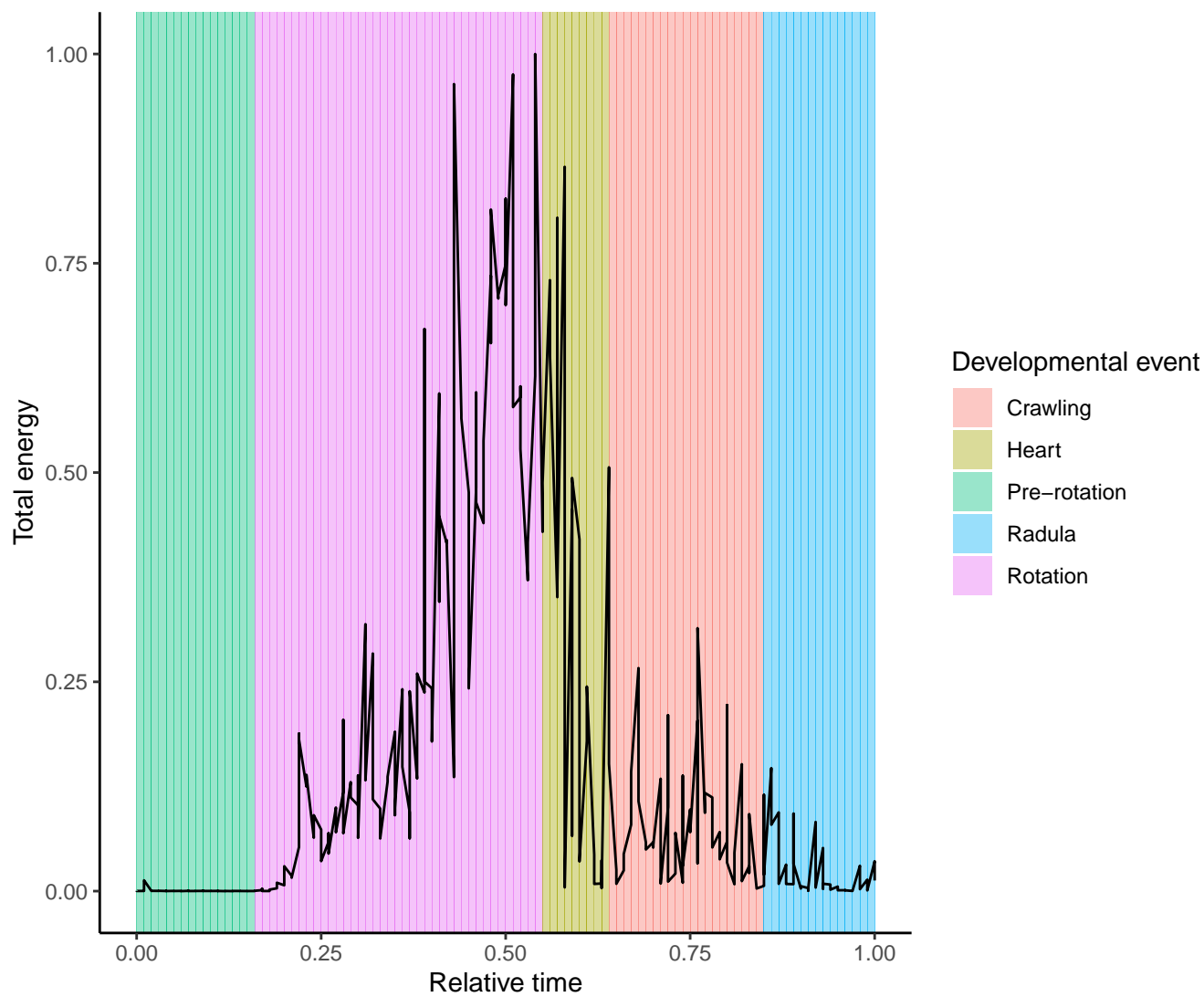

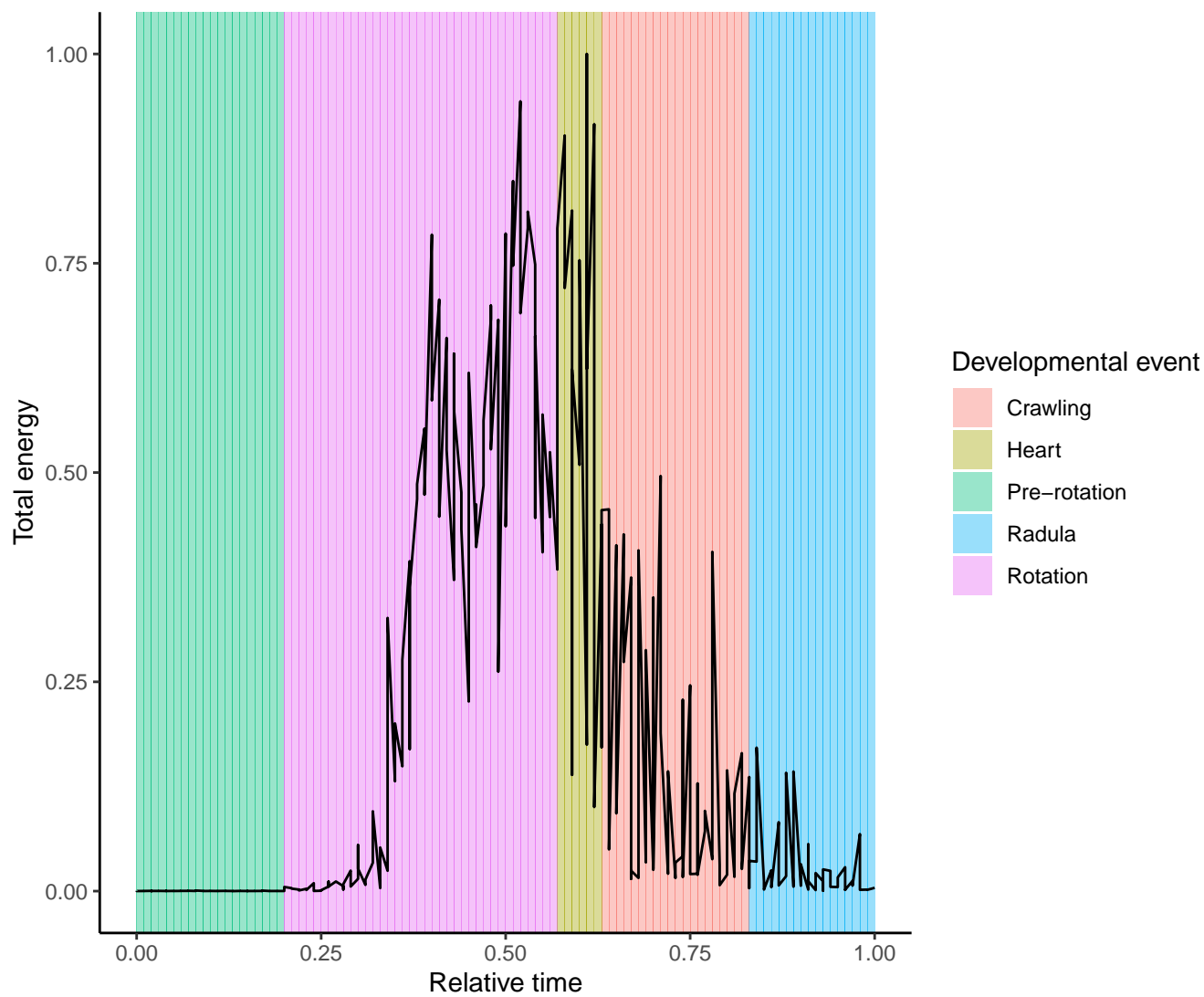

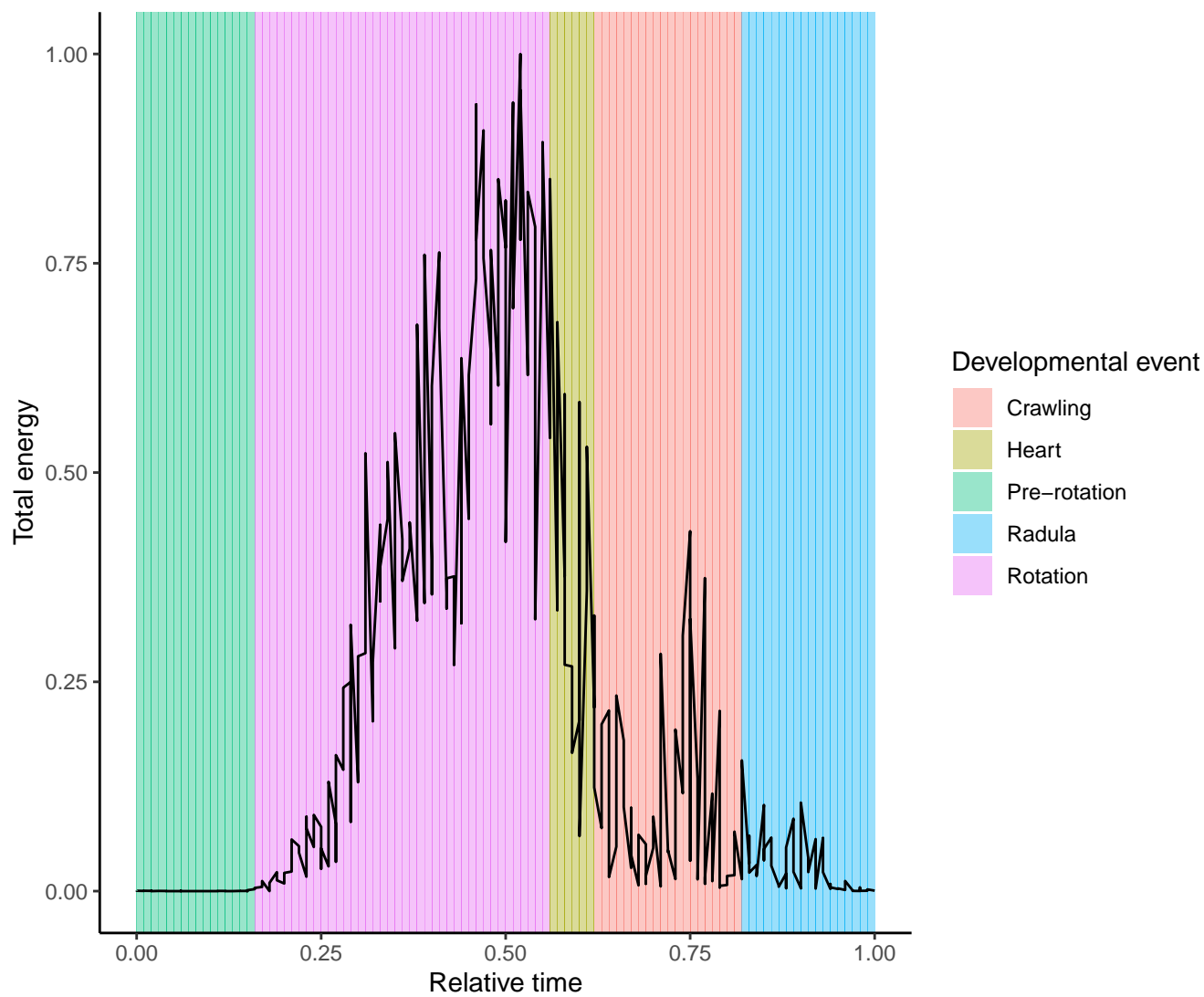

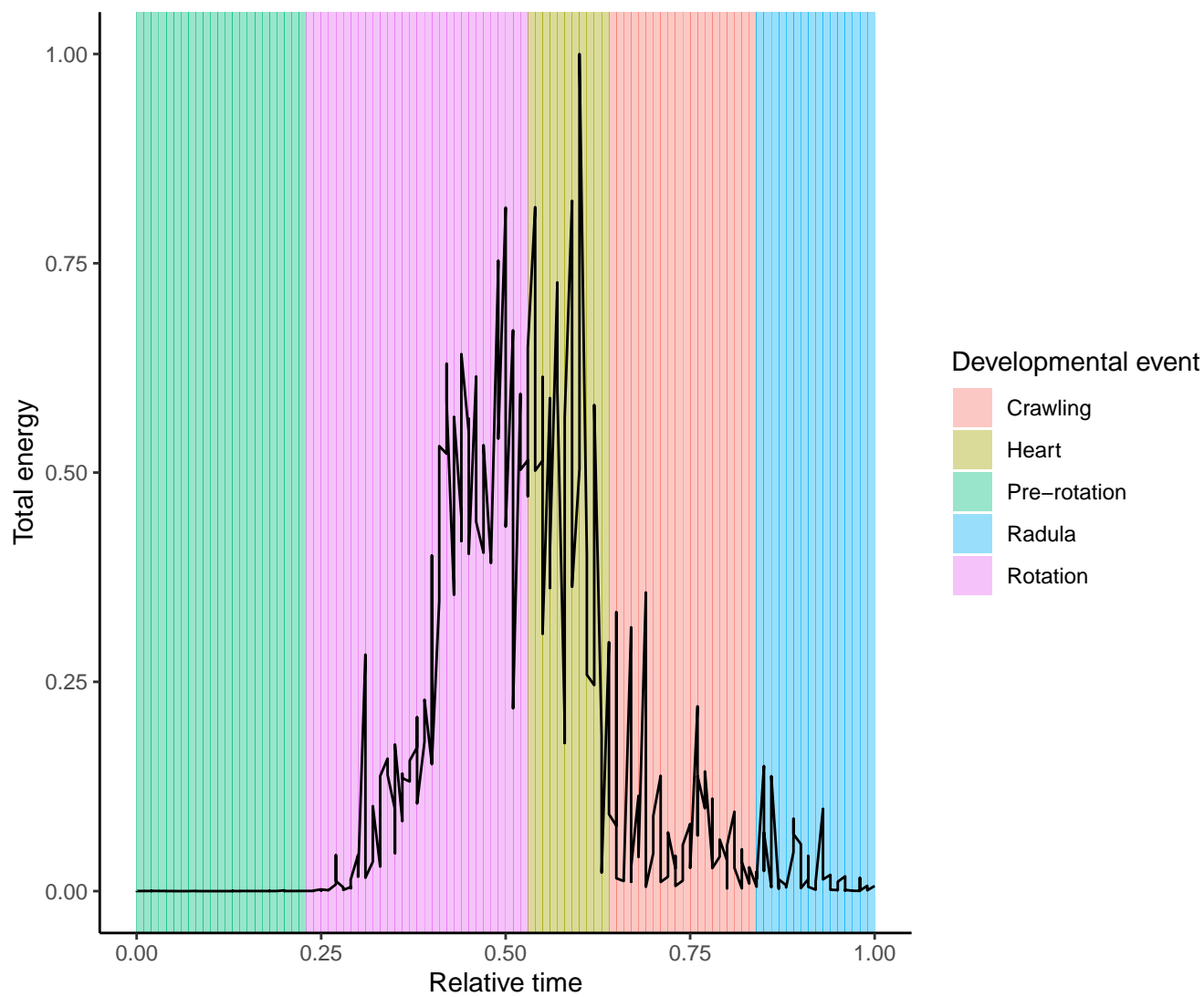

Supplement: Supplementary file 4 [file Image3.PDF]

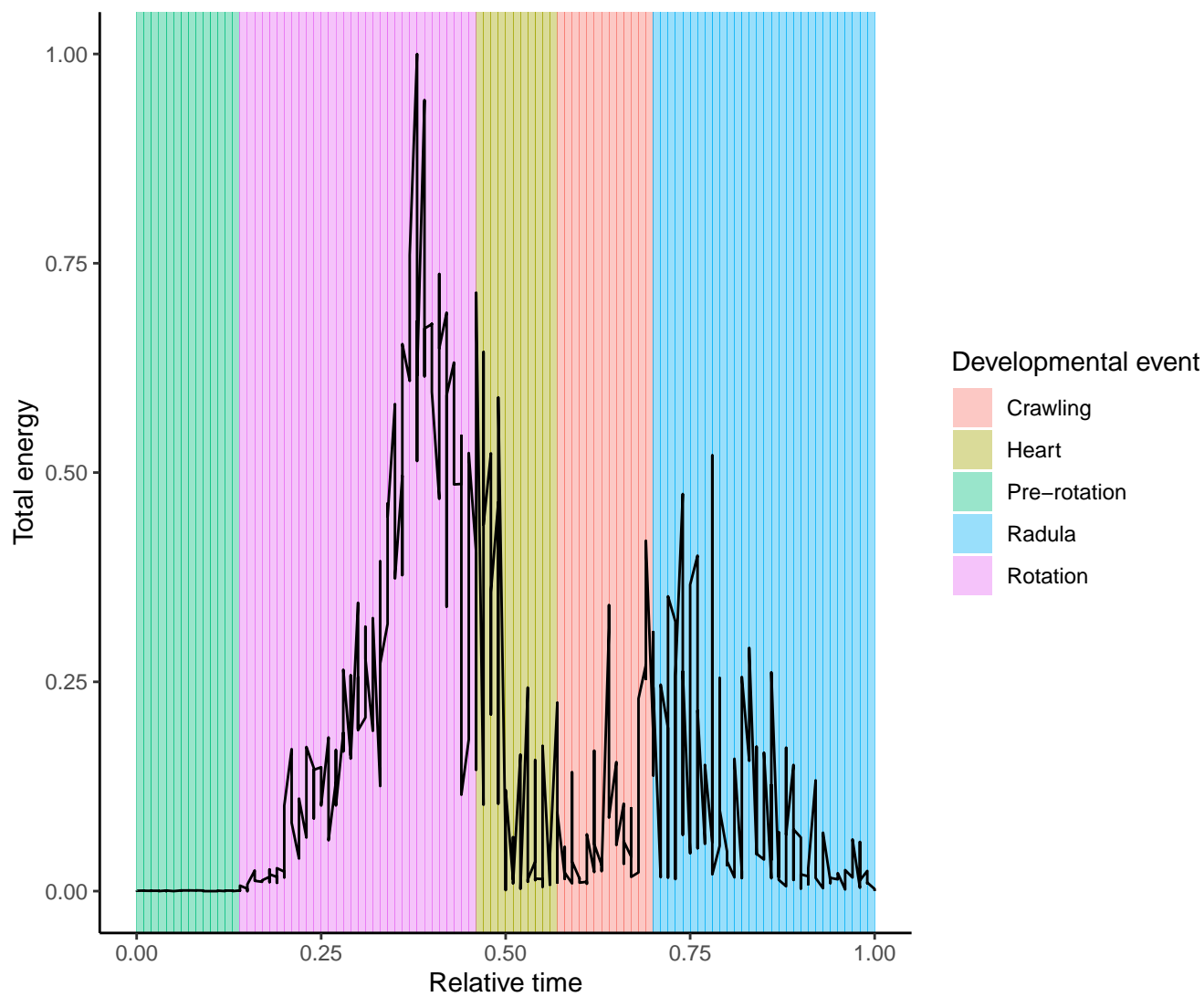

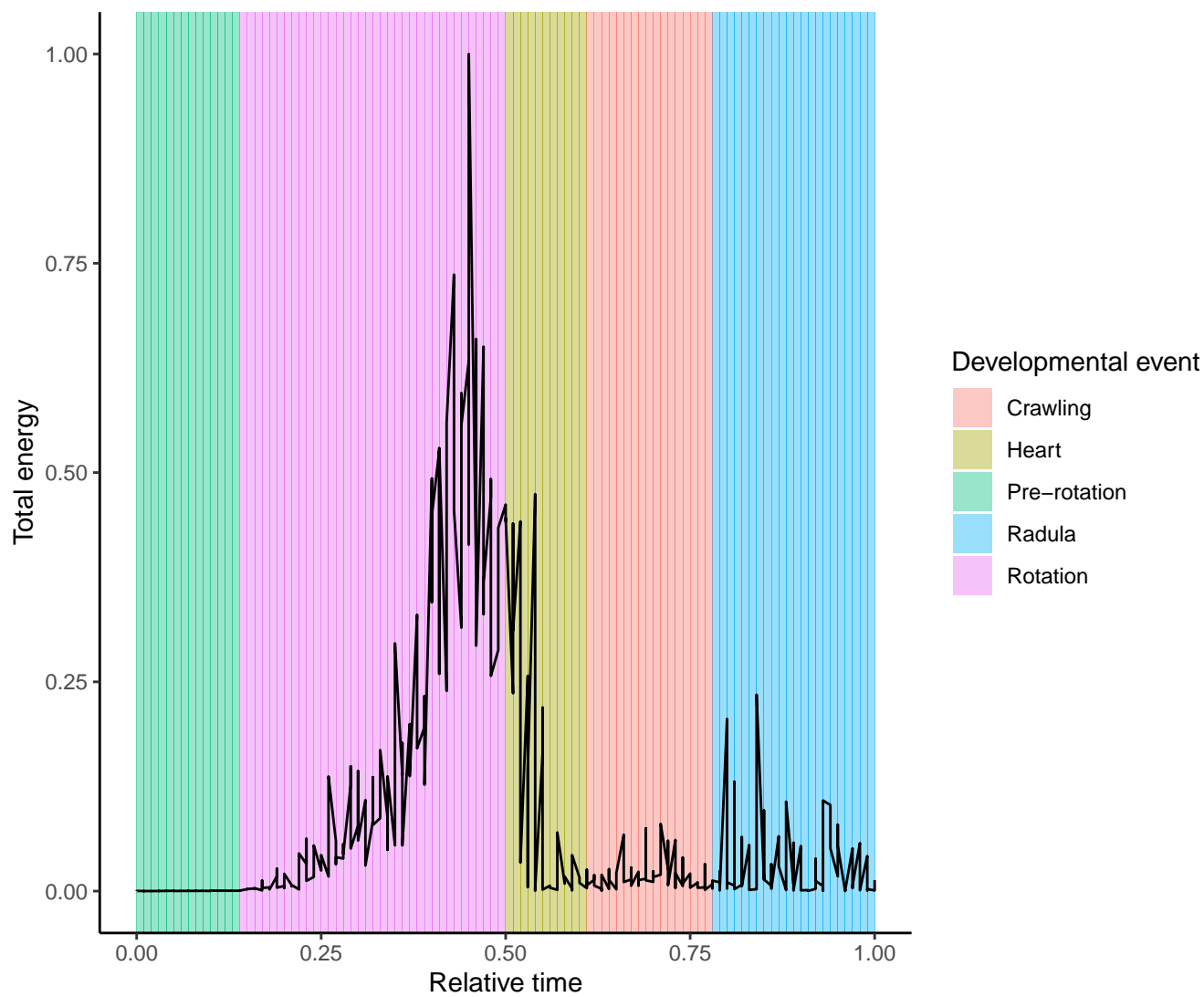

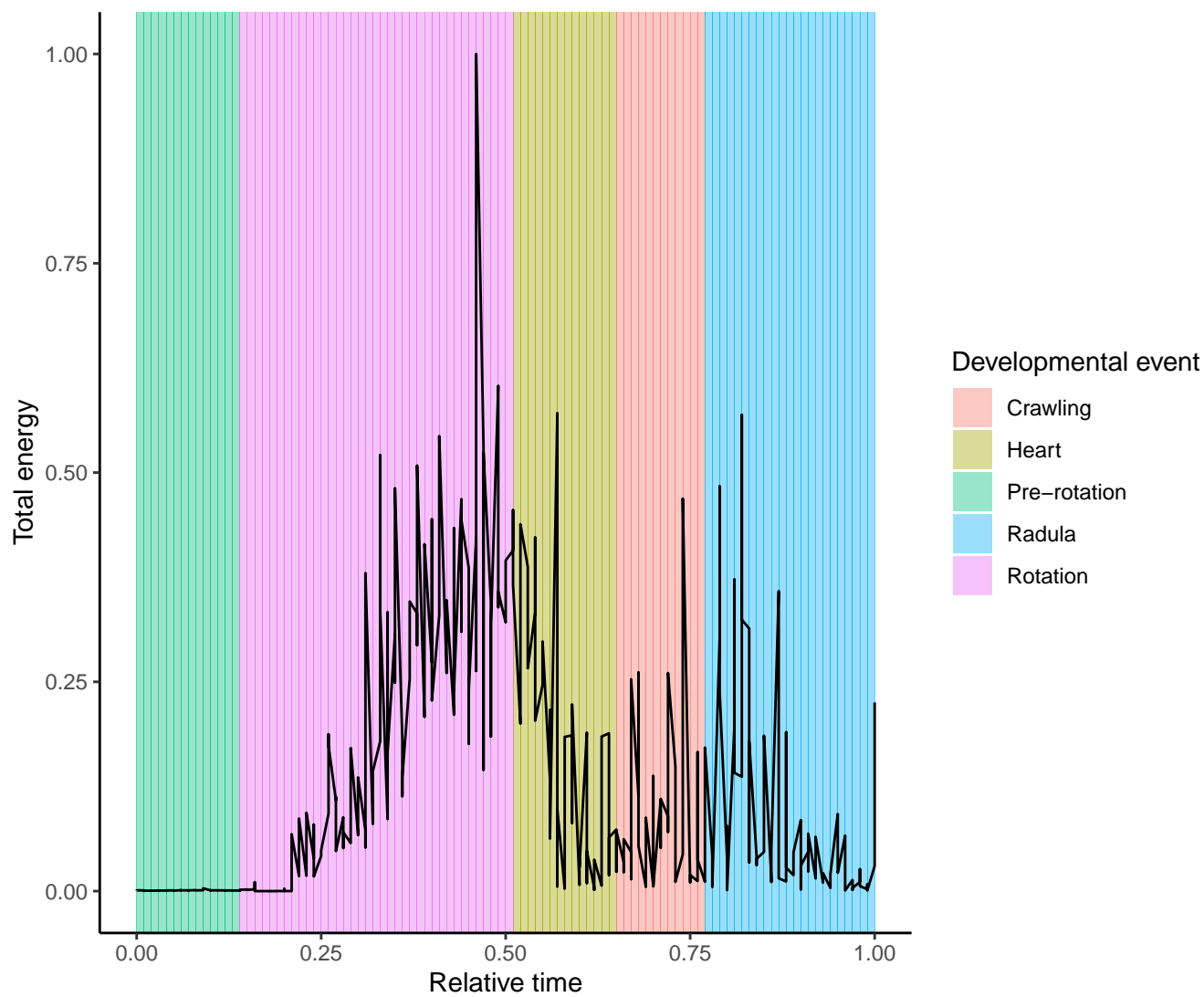

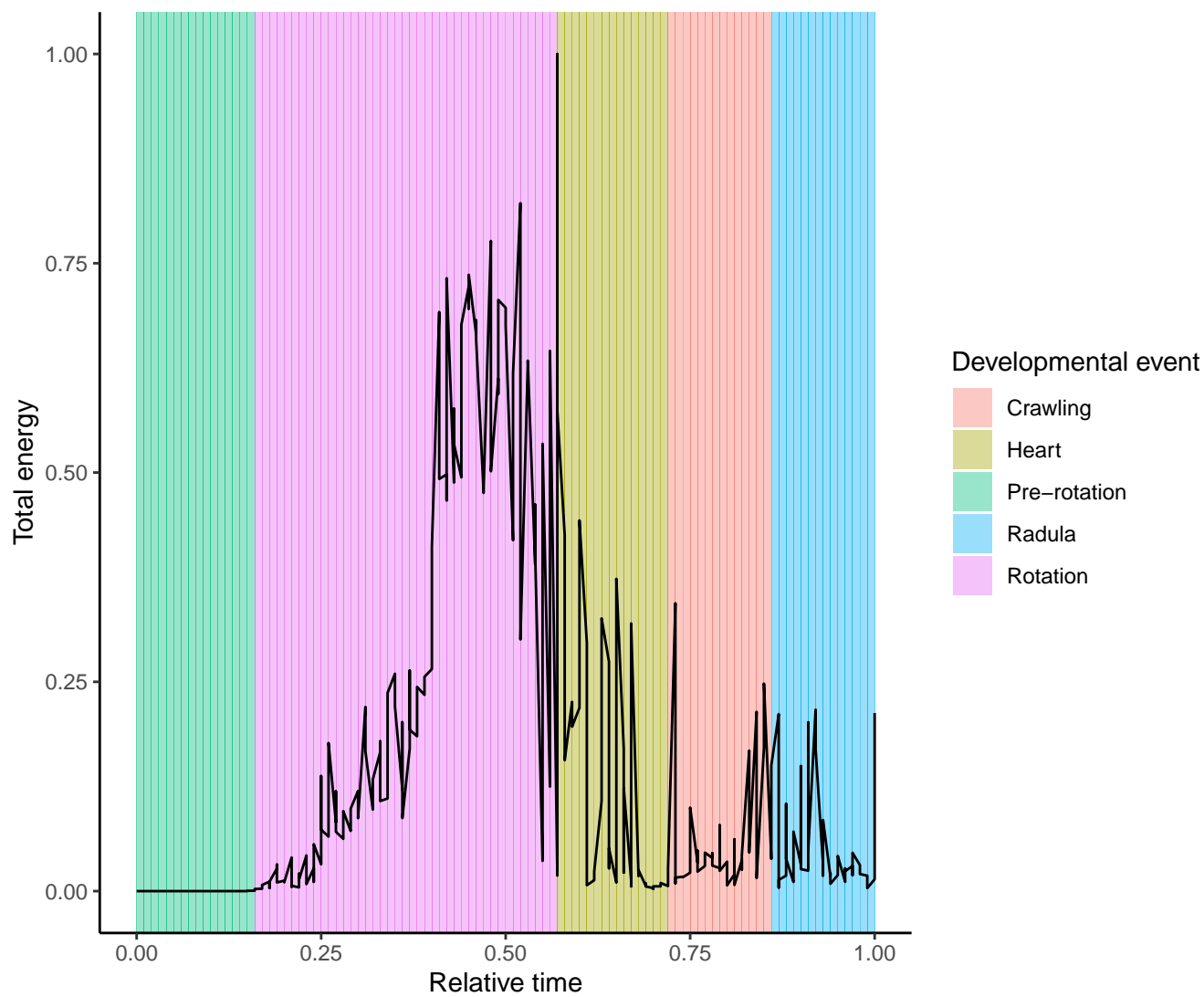

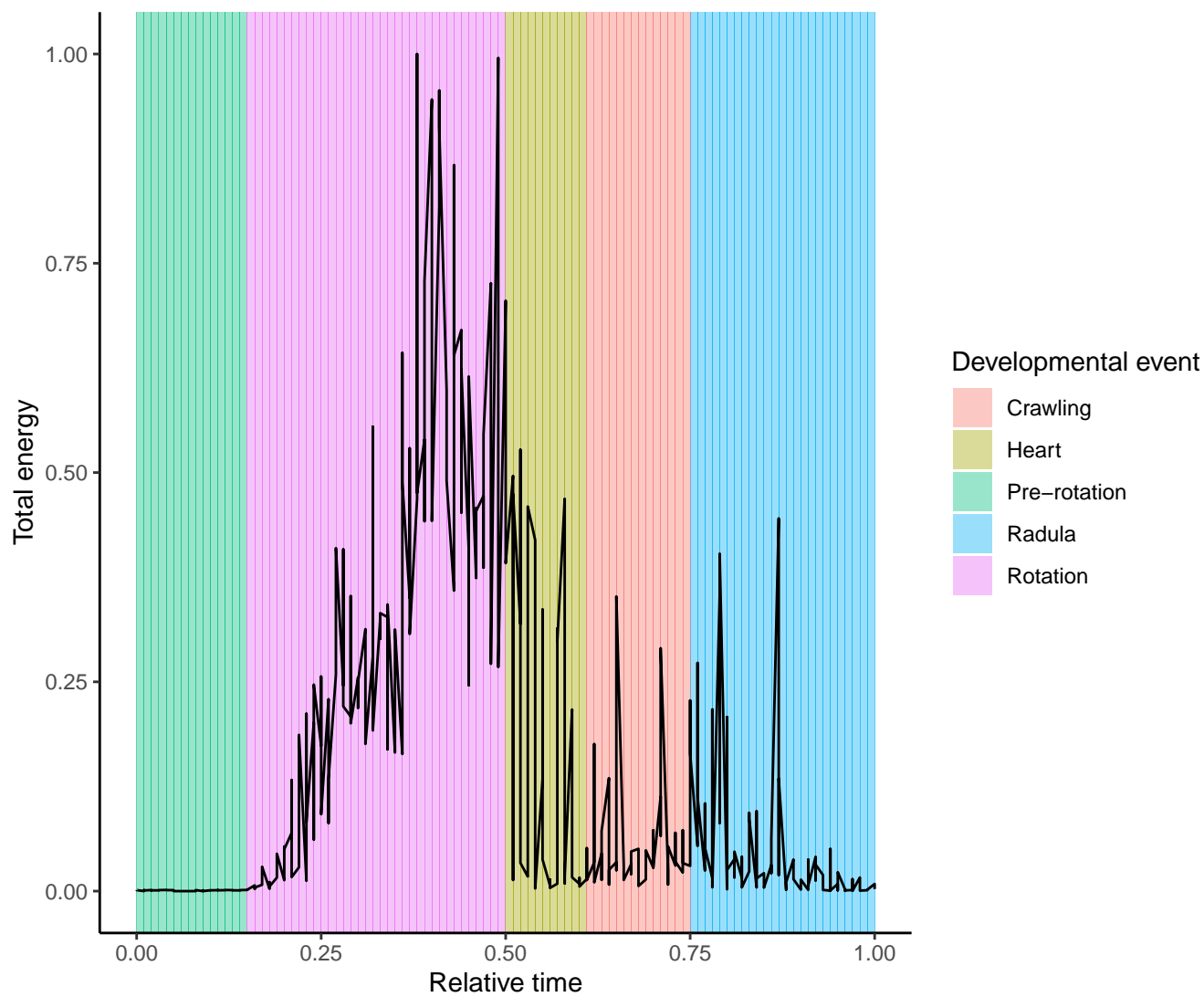

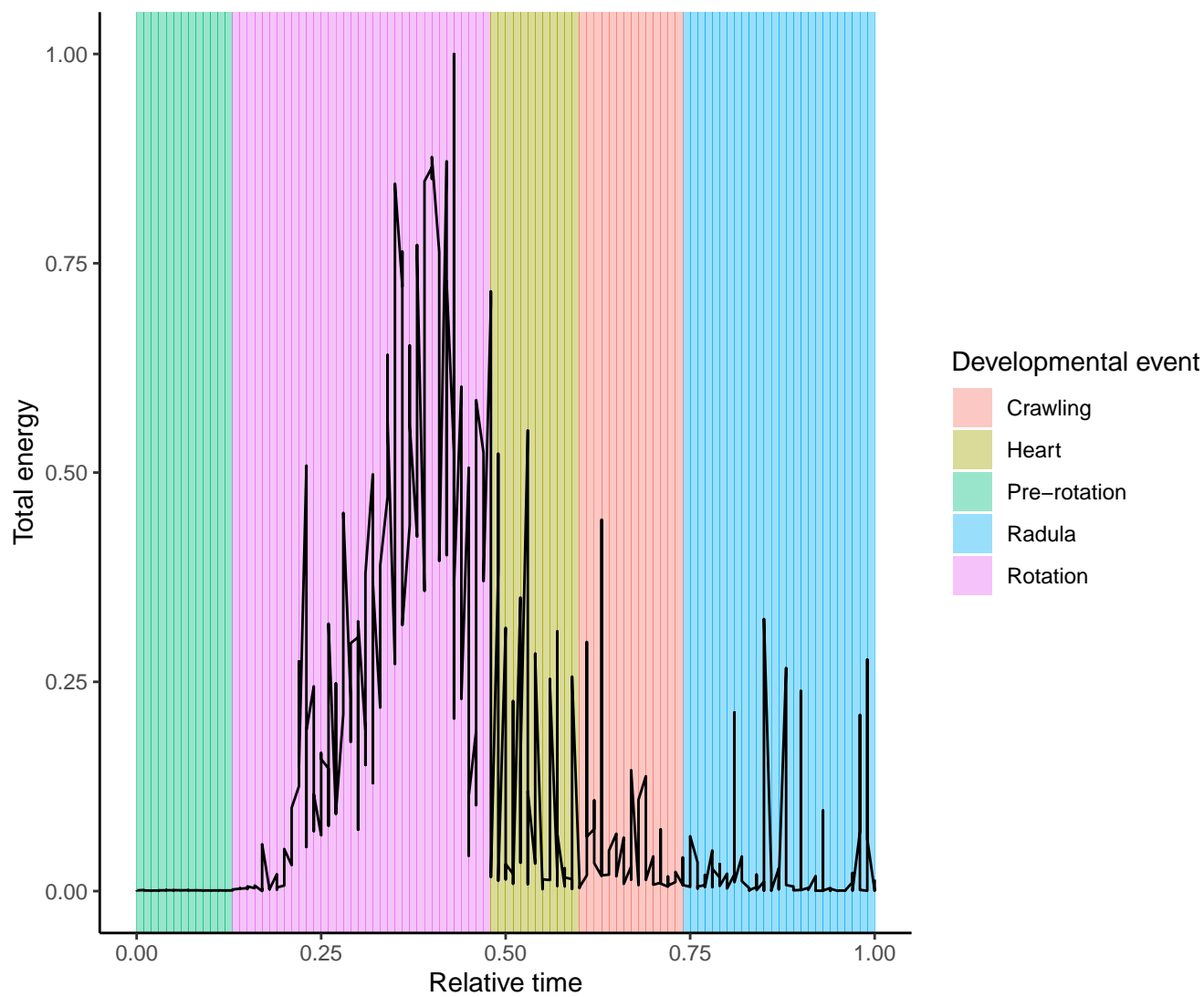

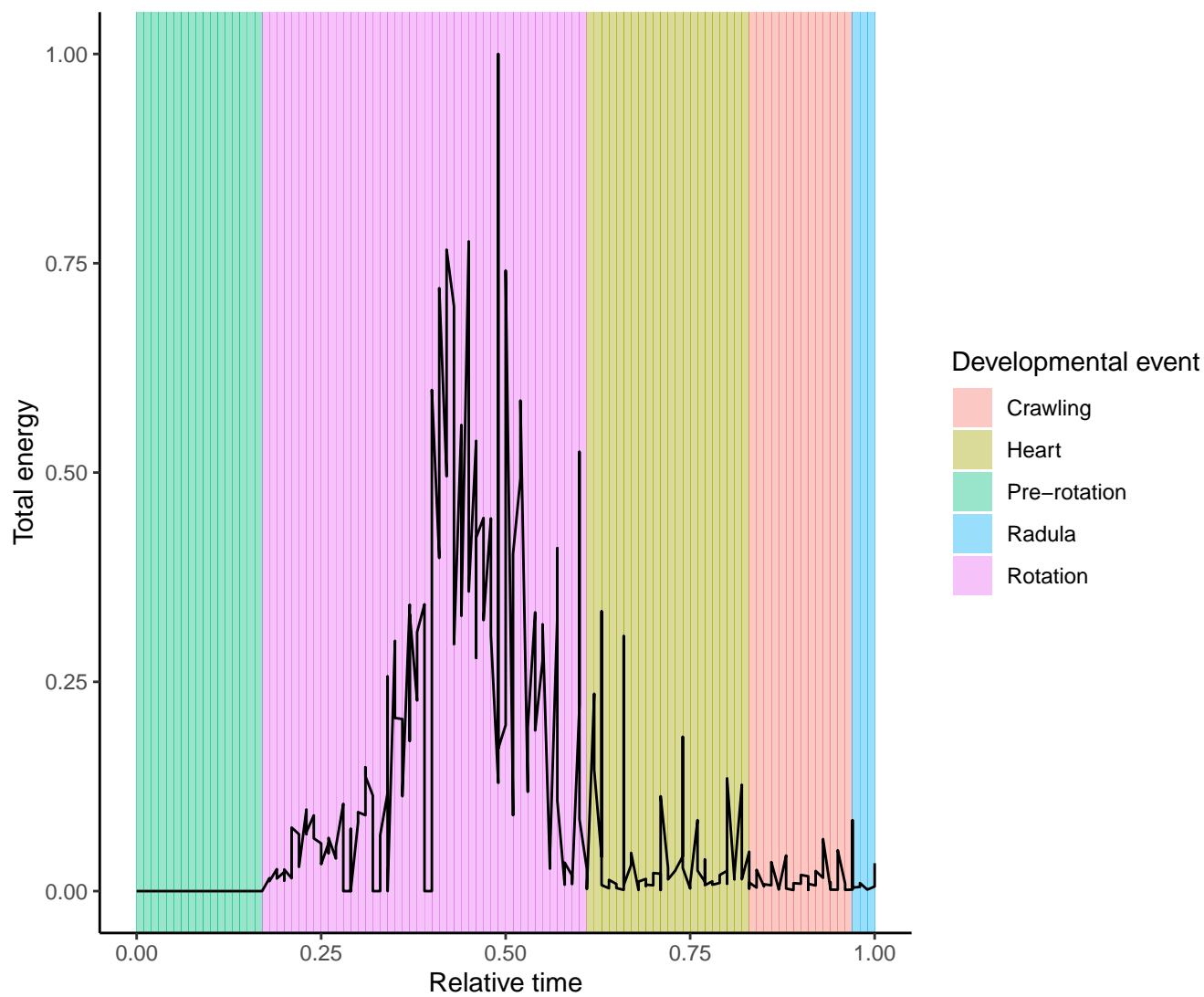

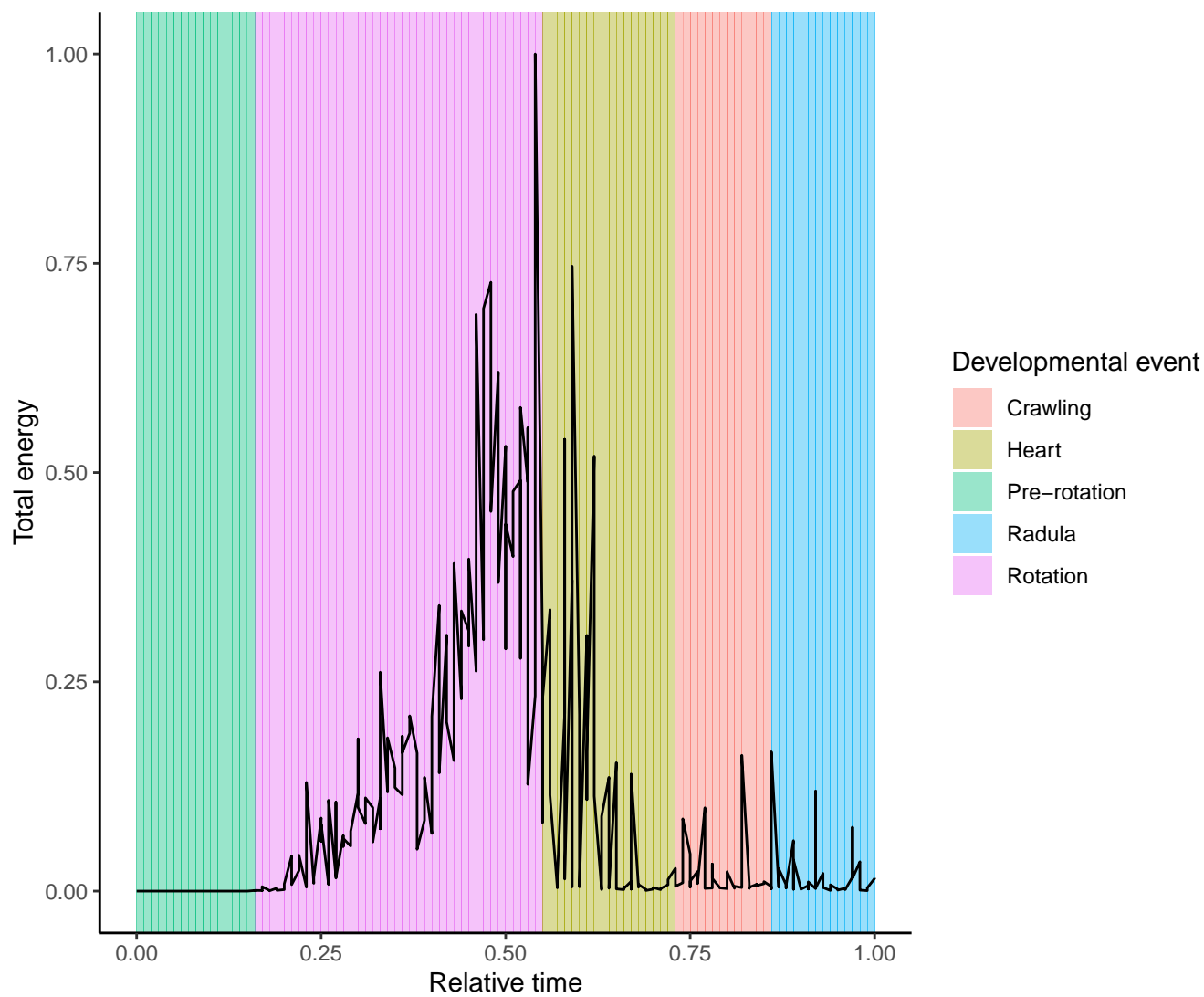

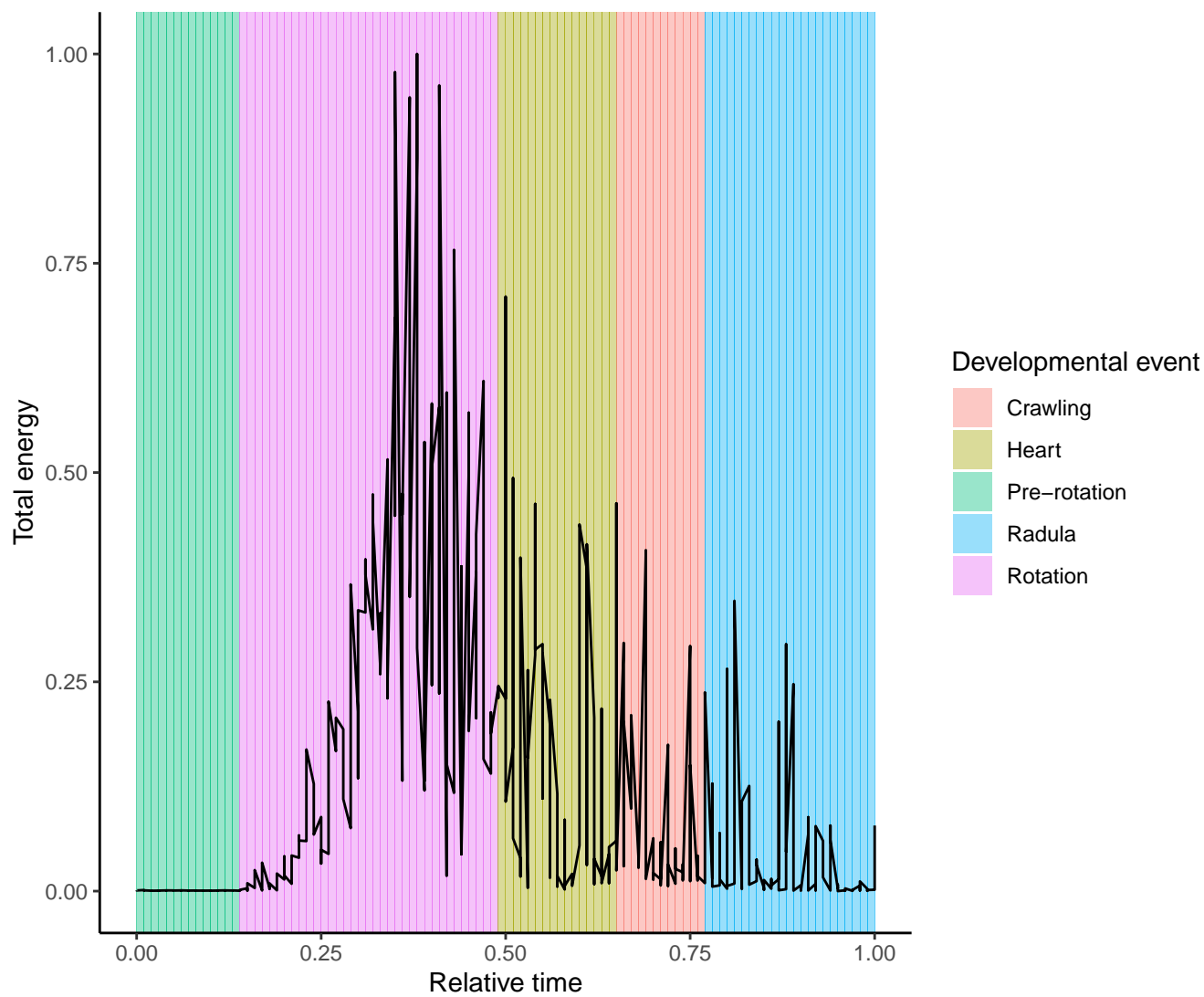

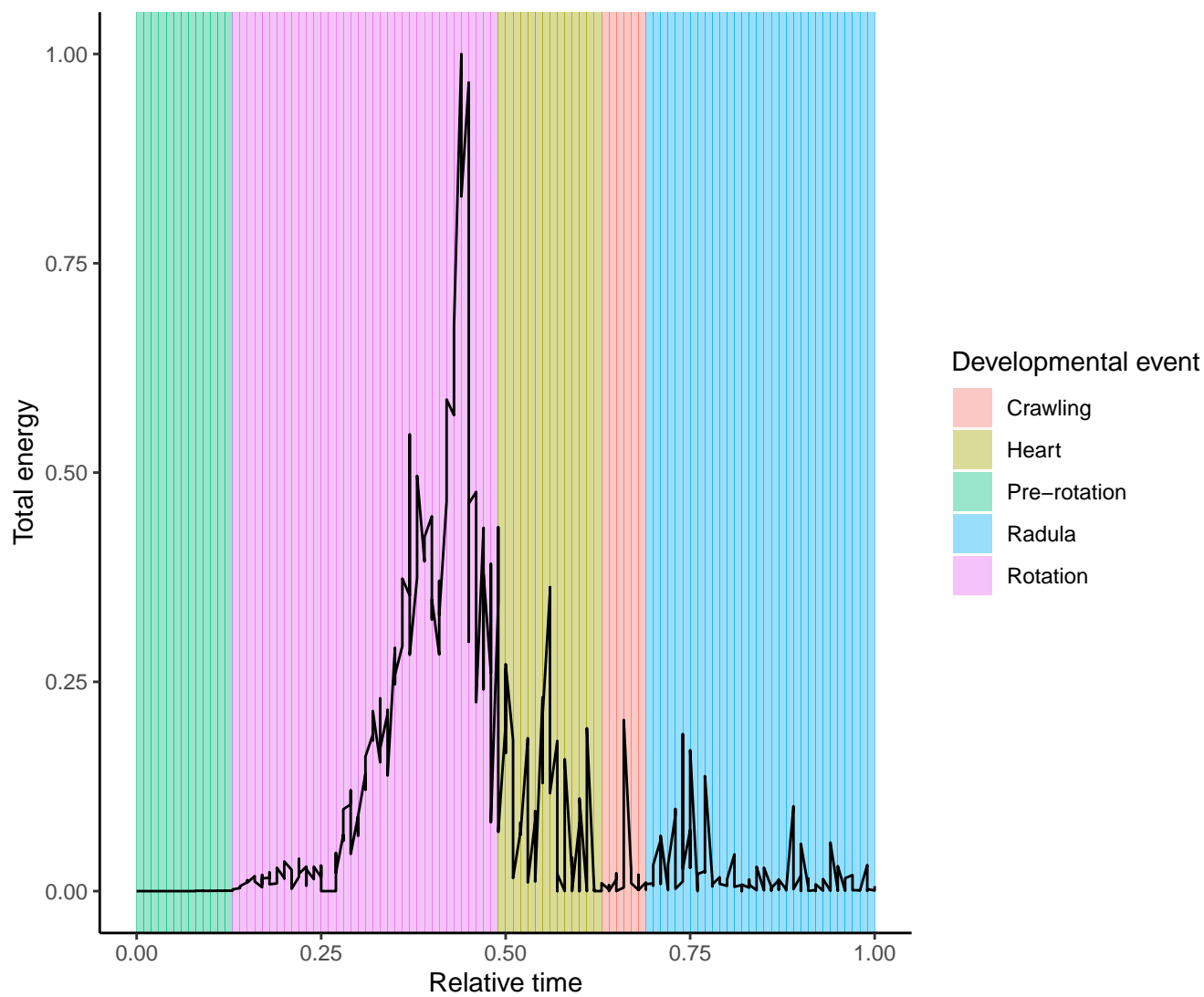

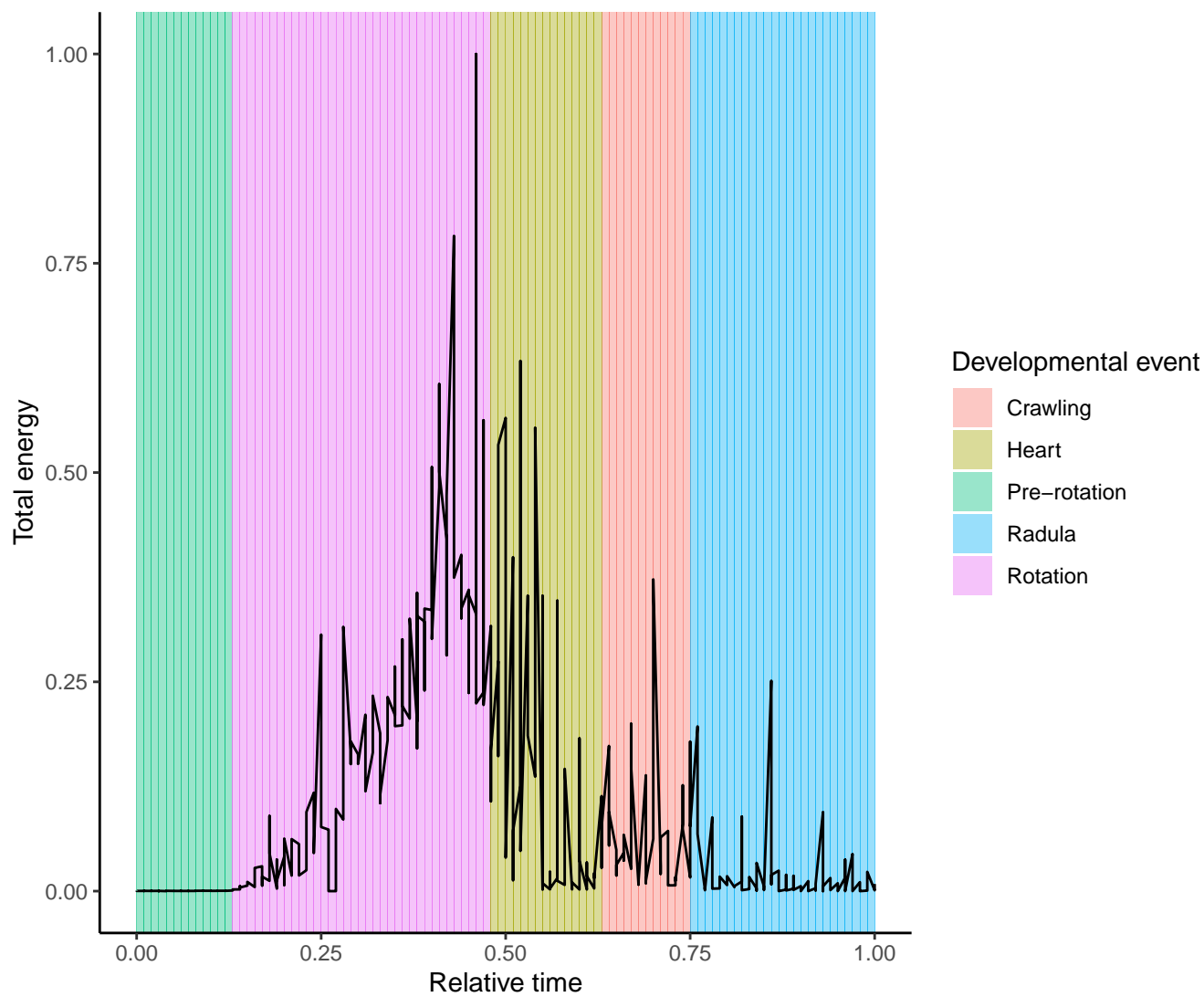

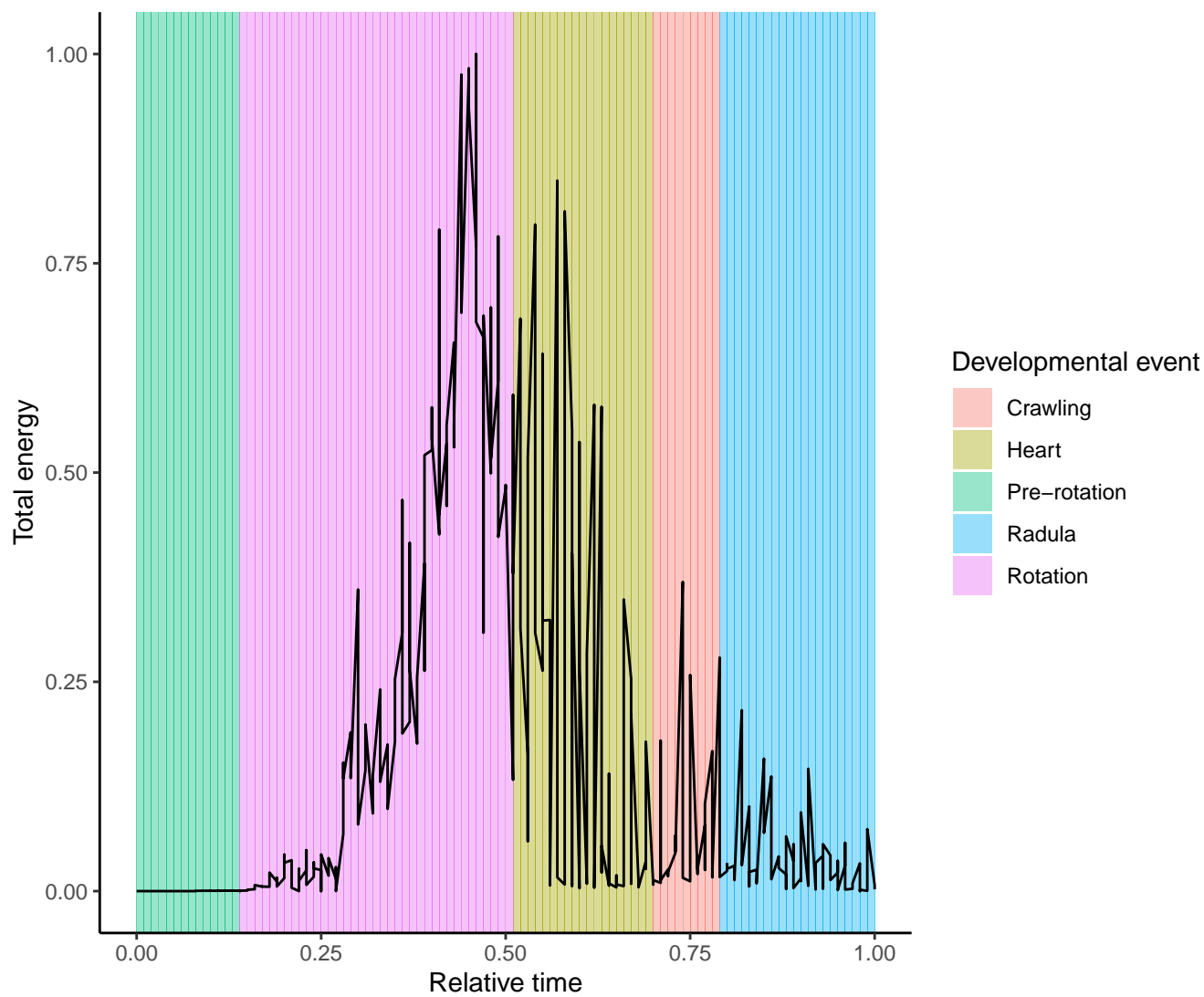

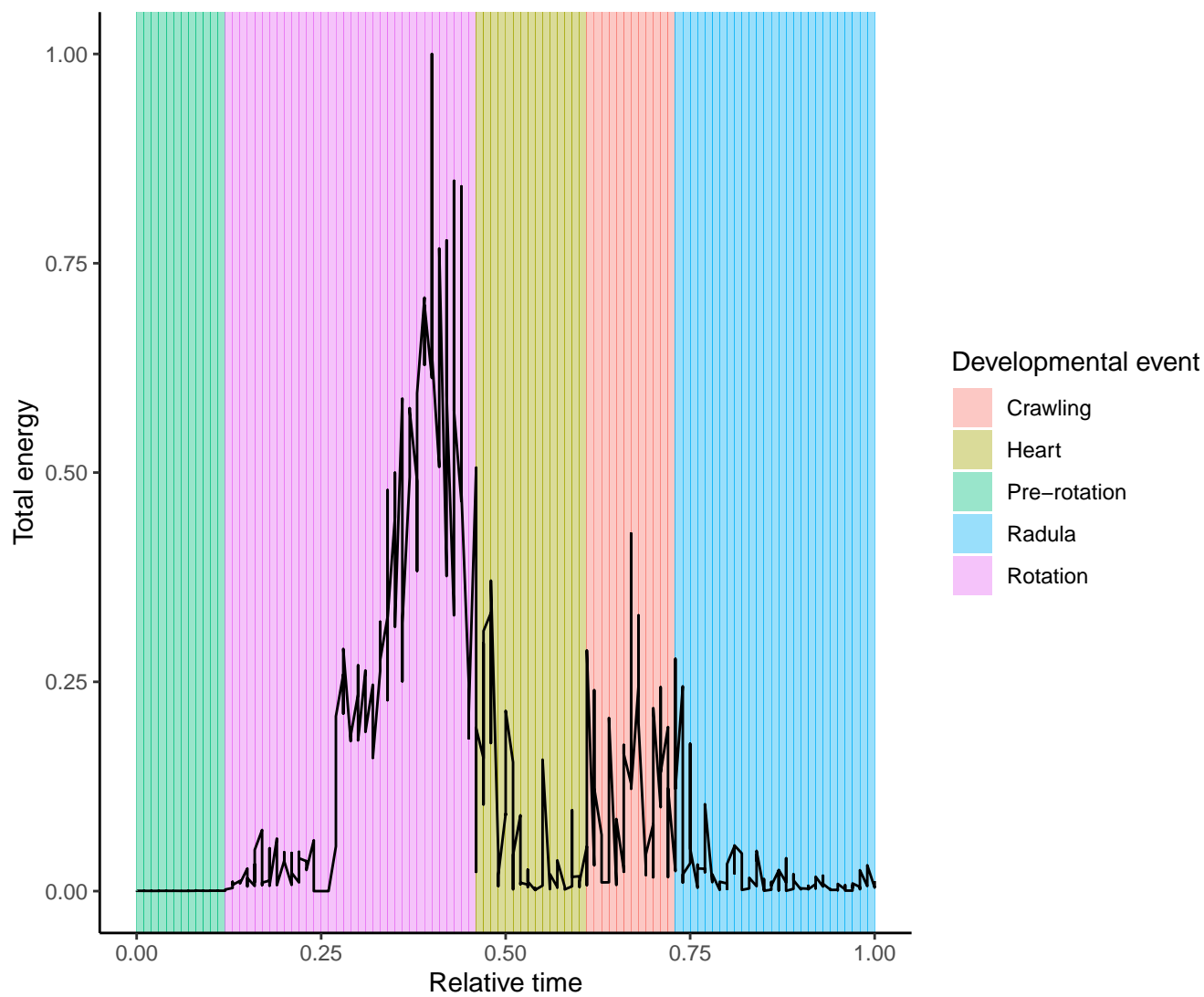

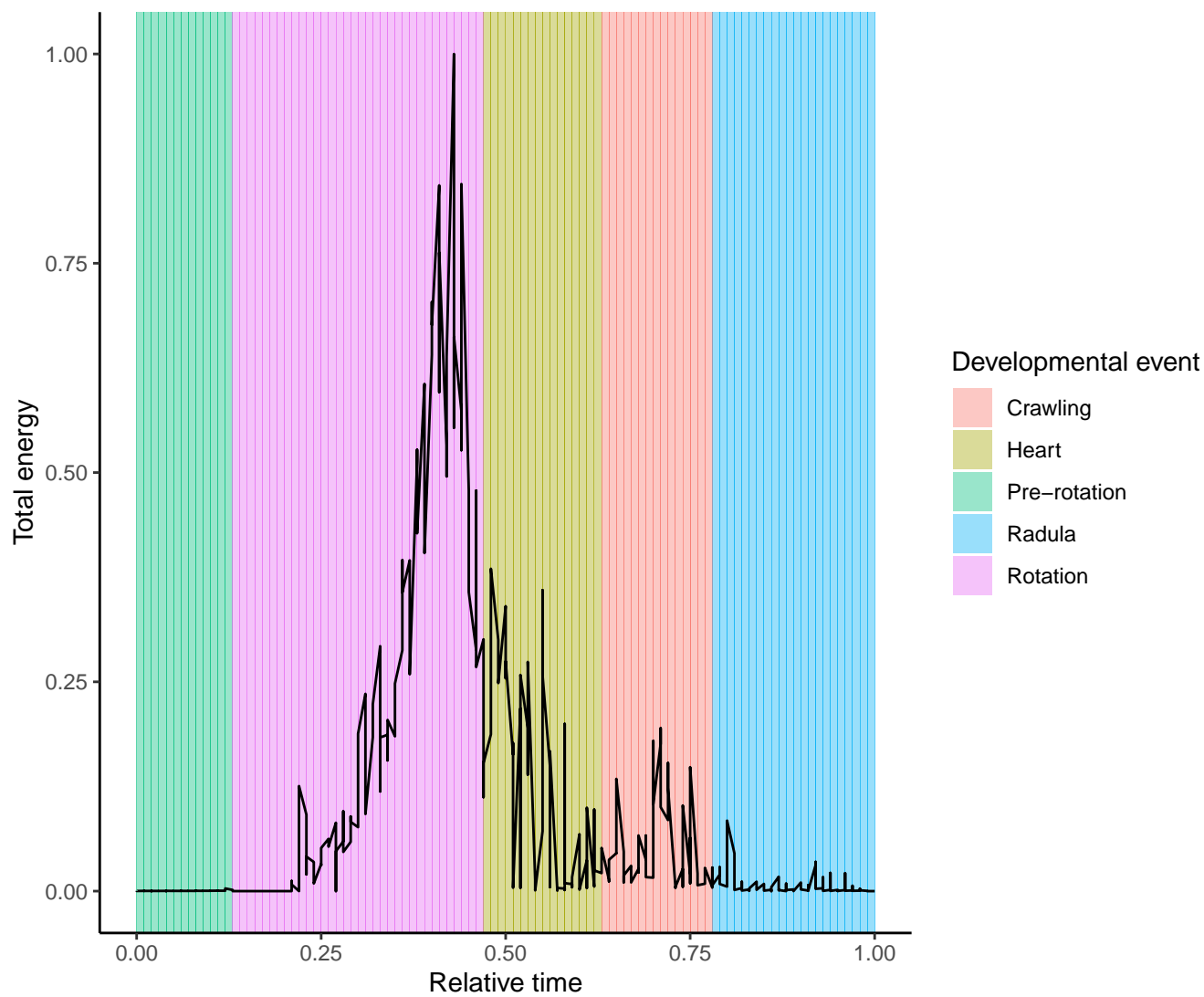

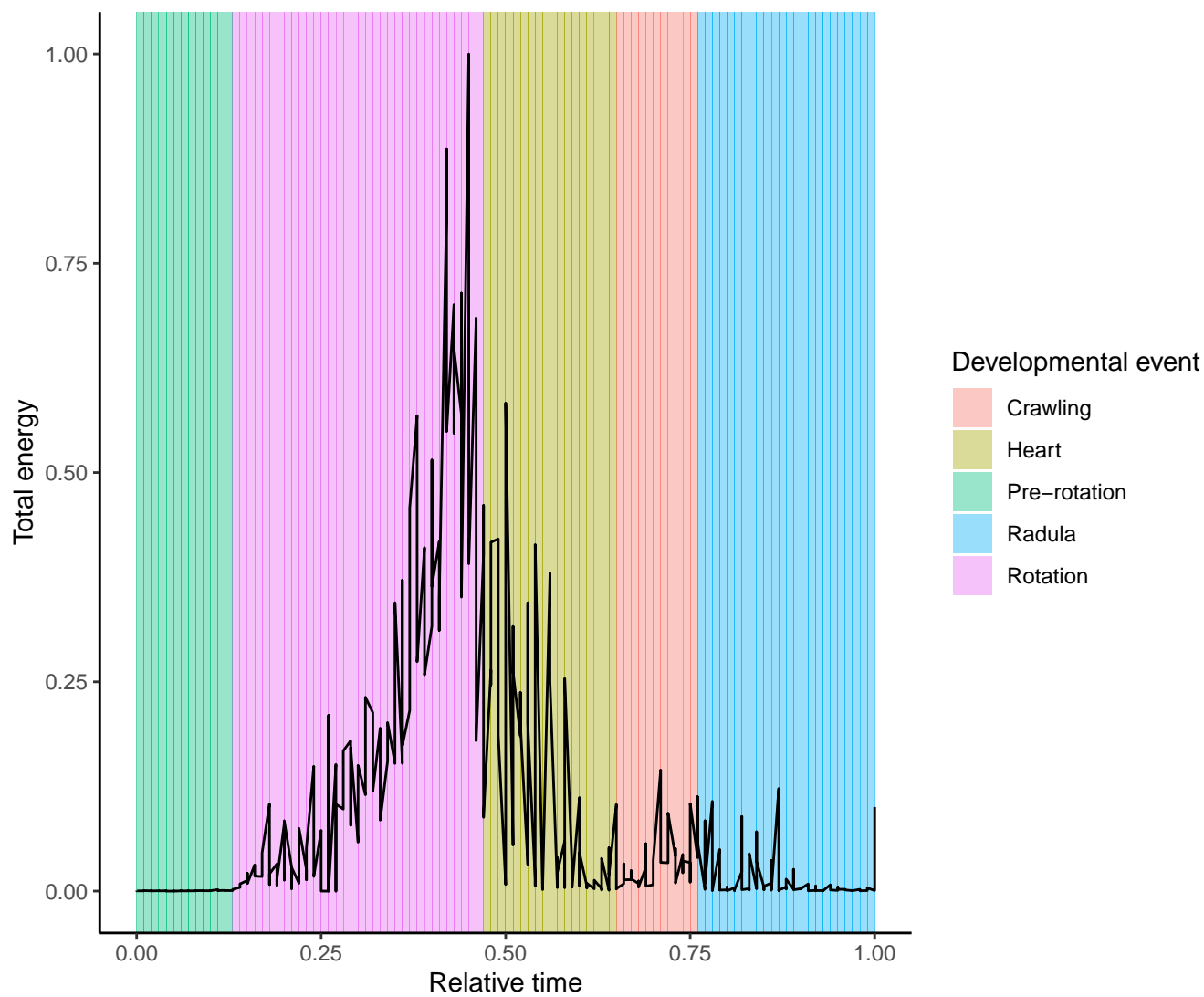

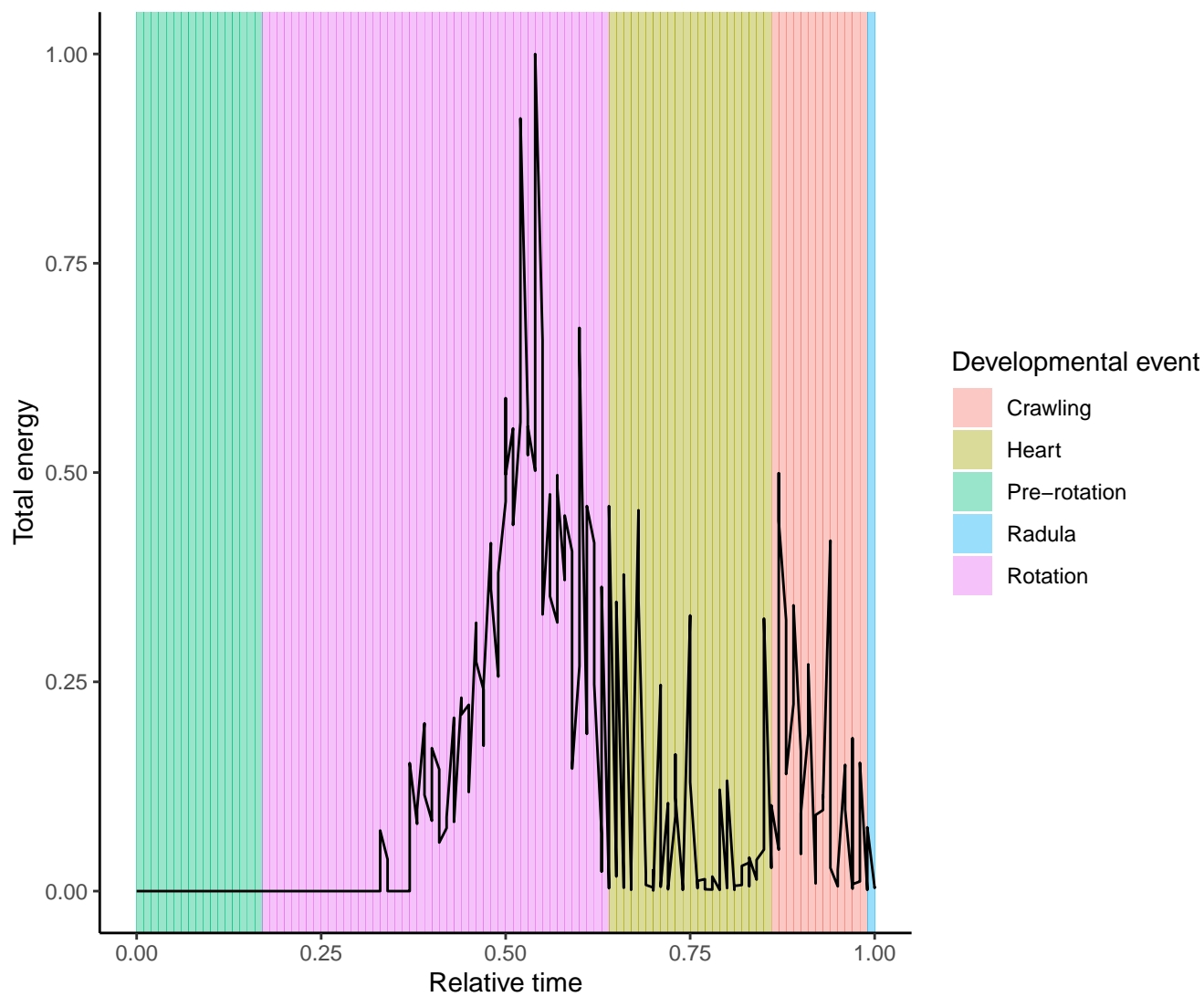

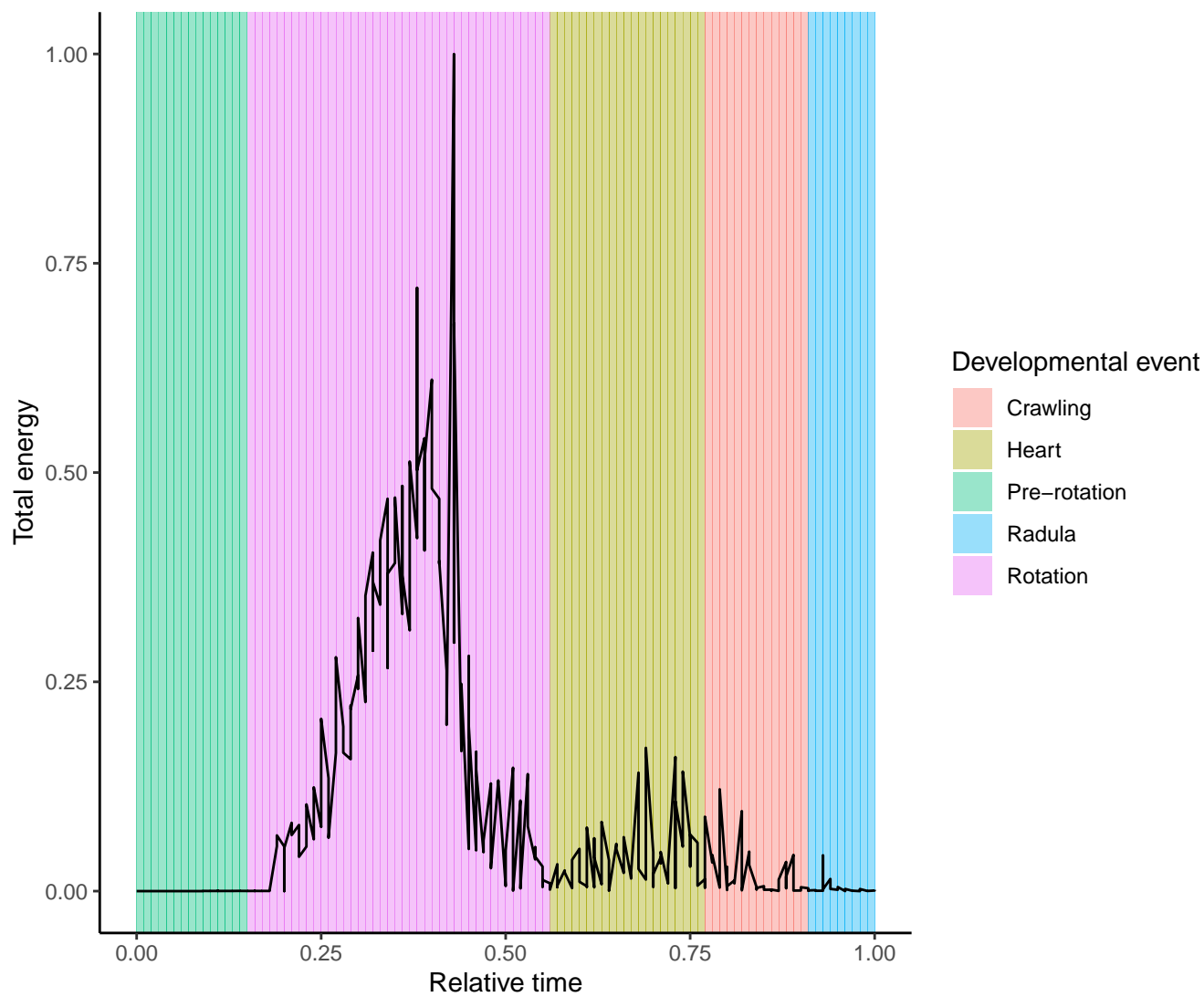

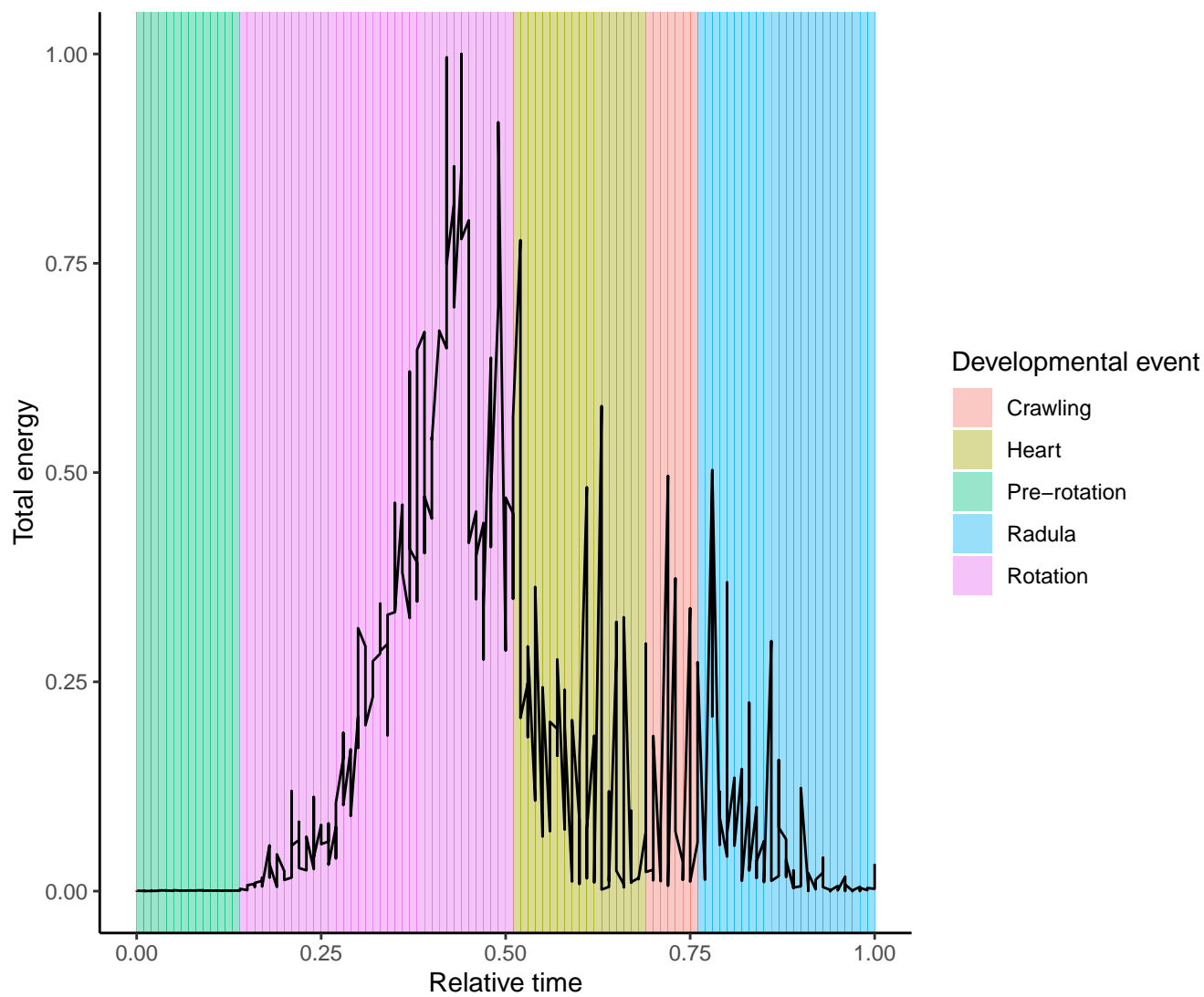

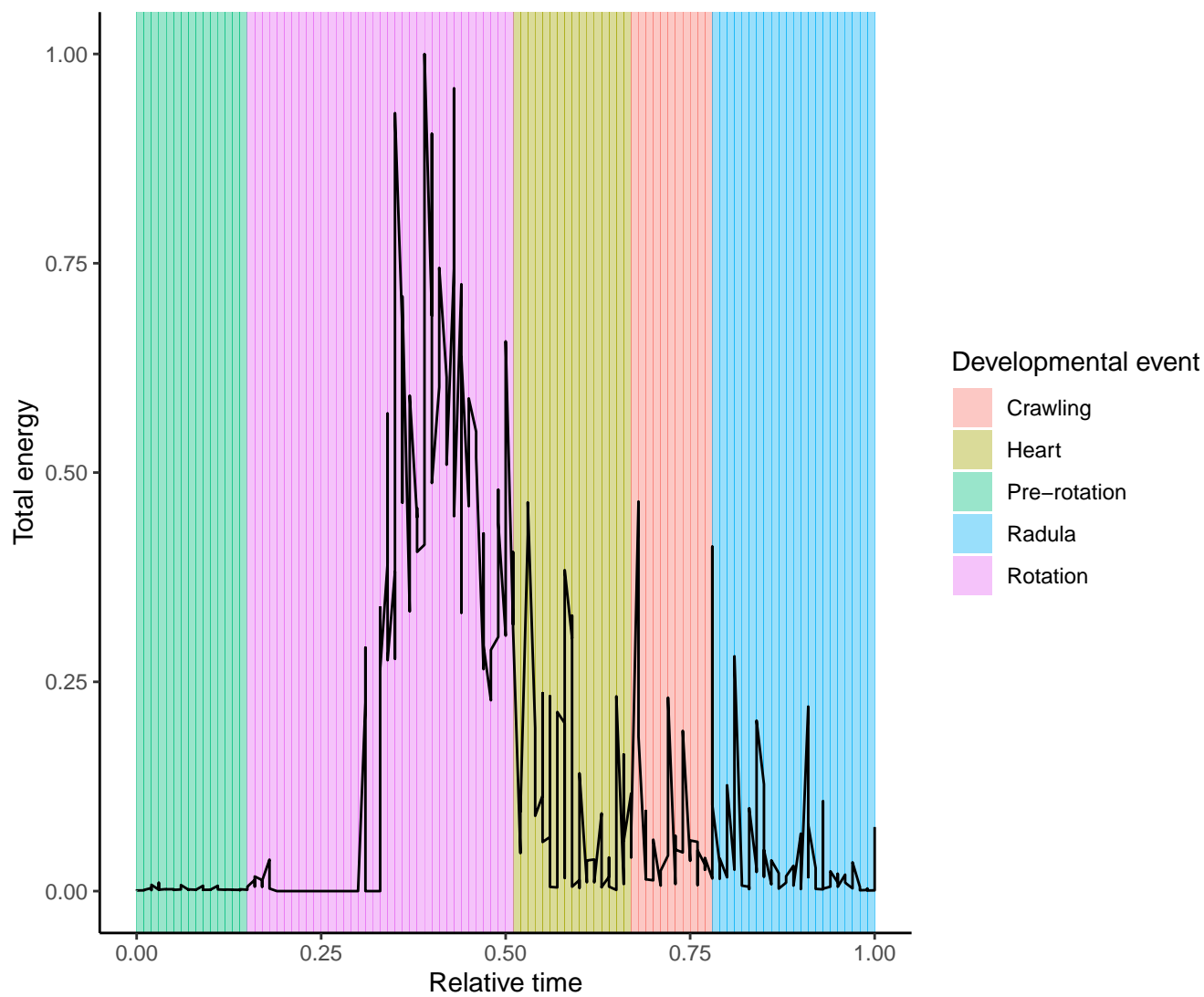

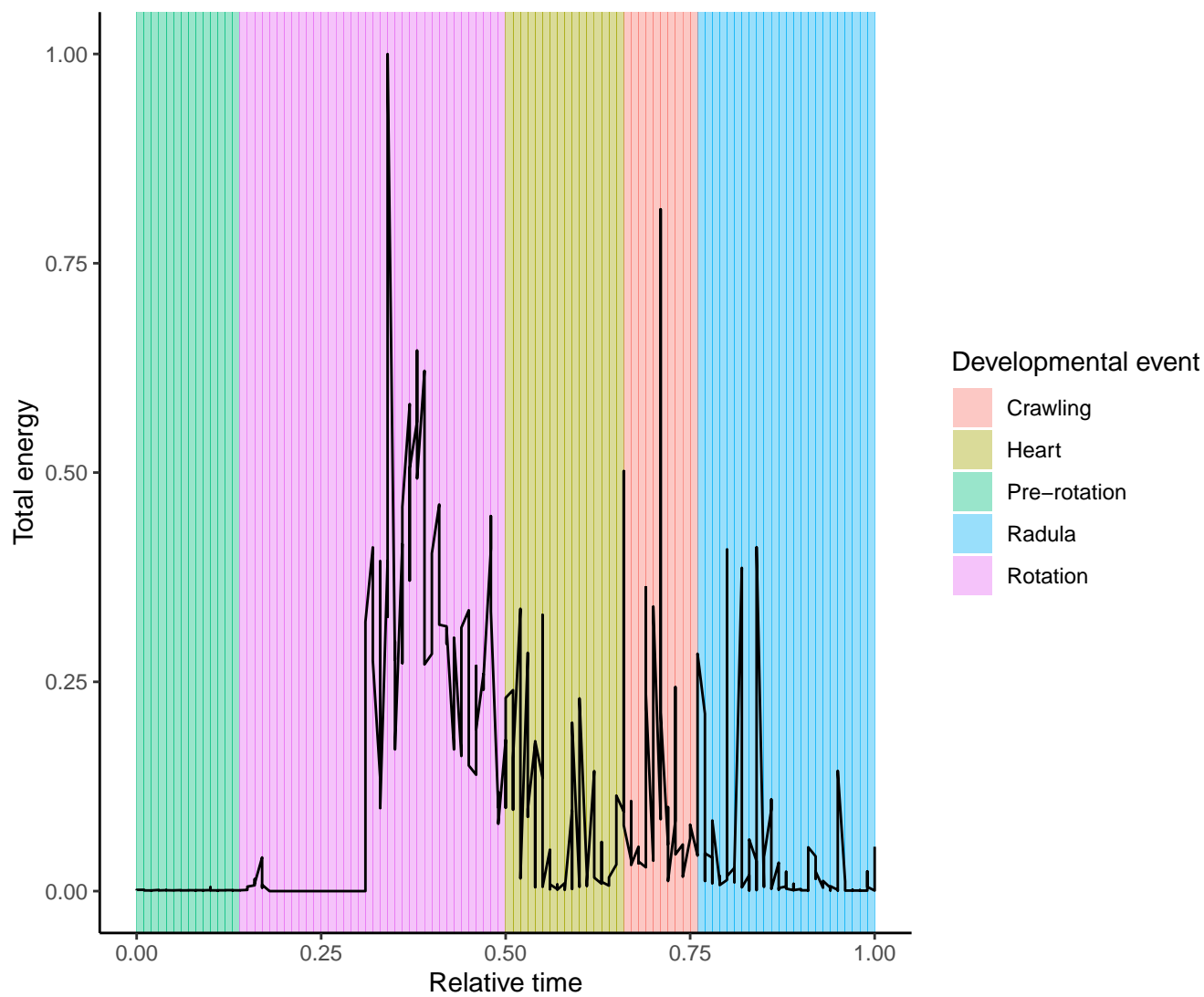

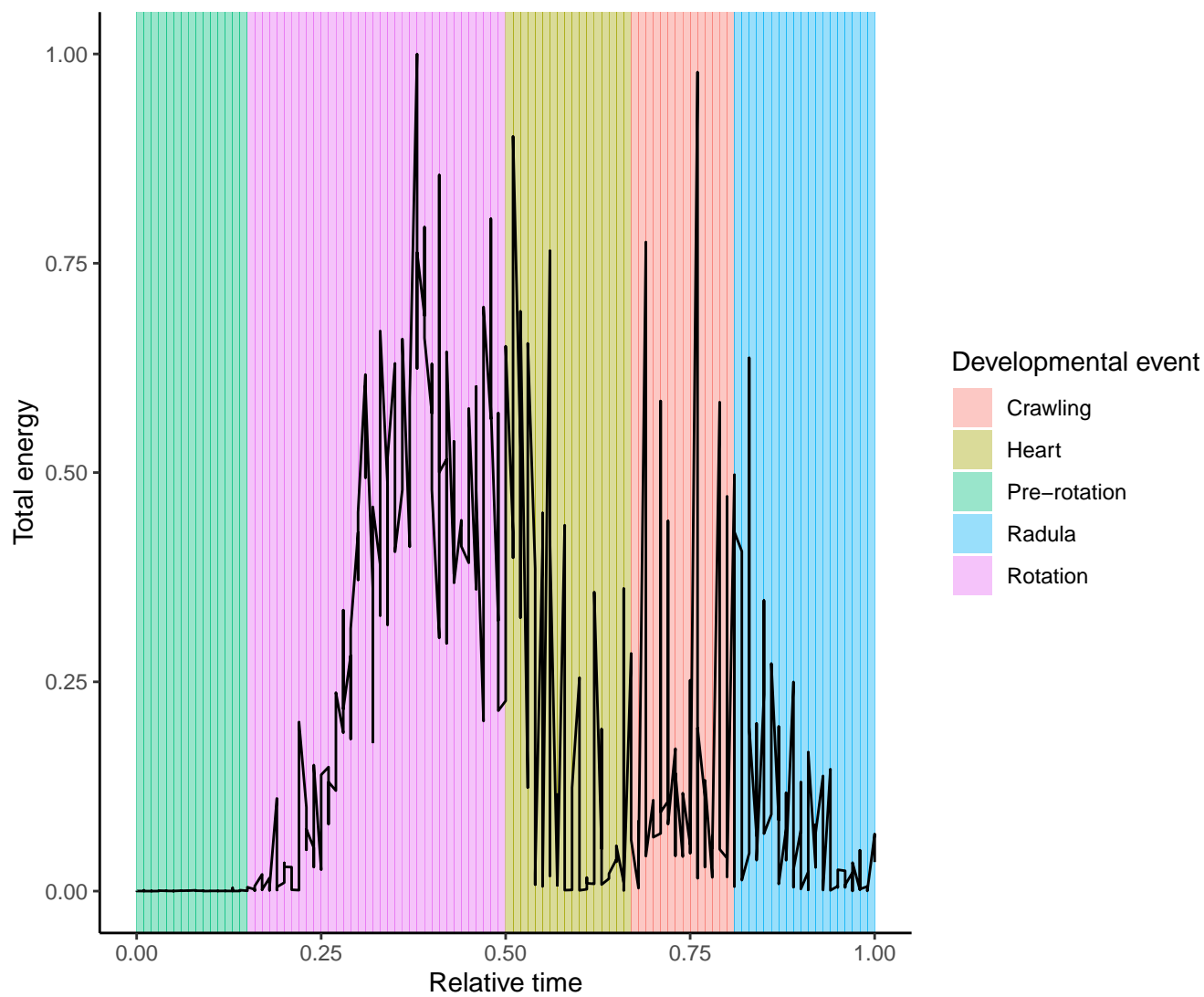

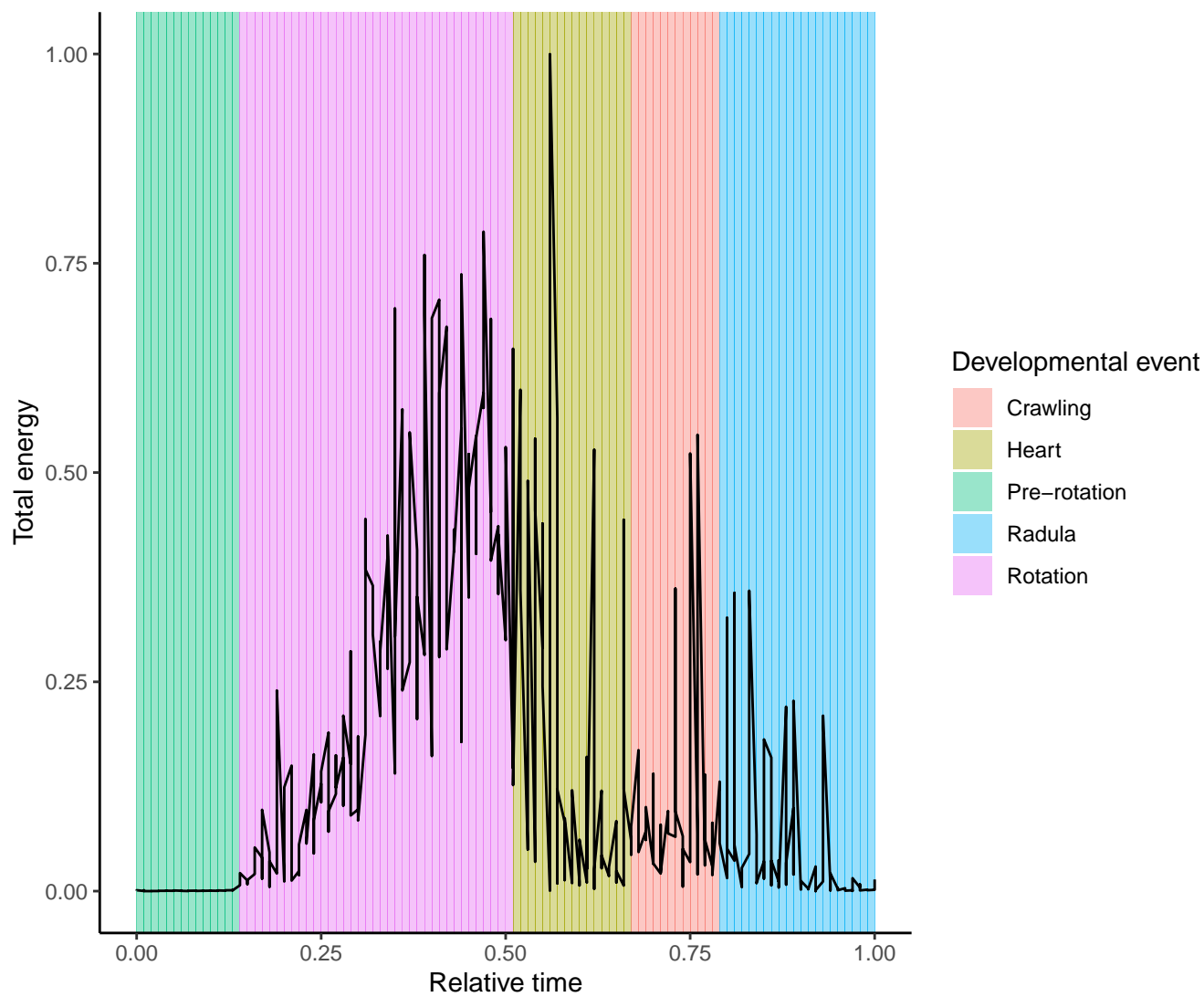

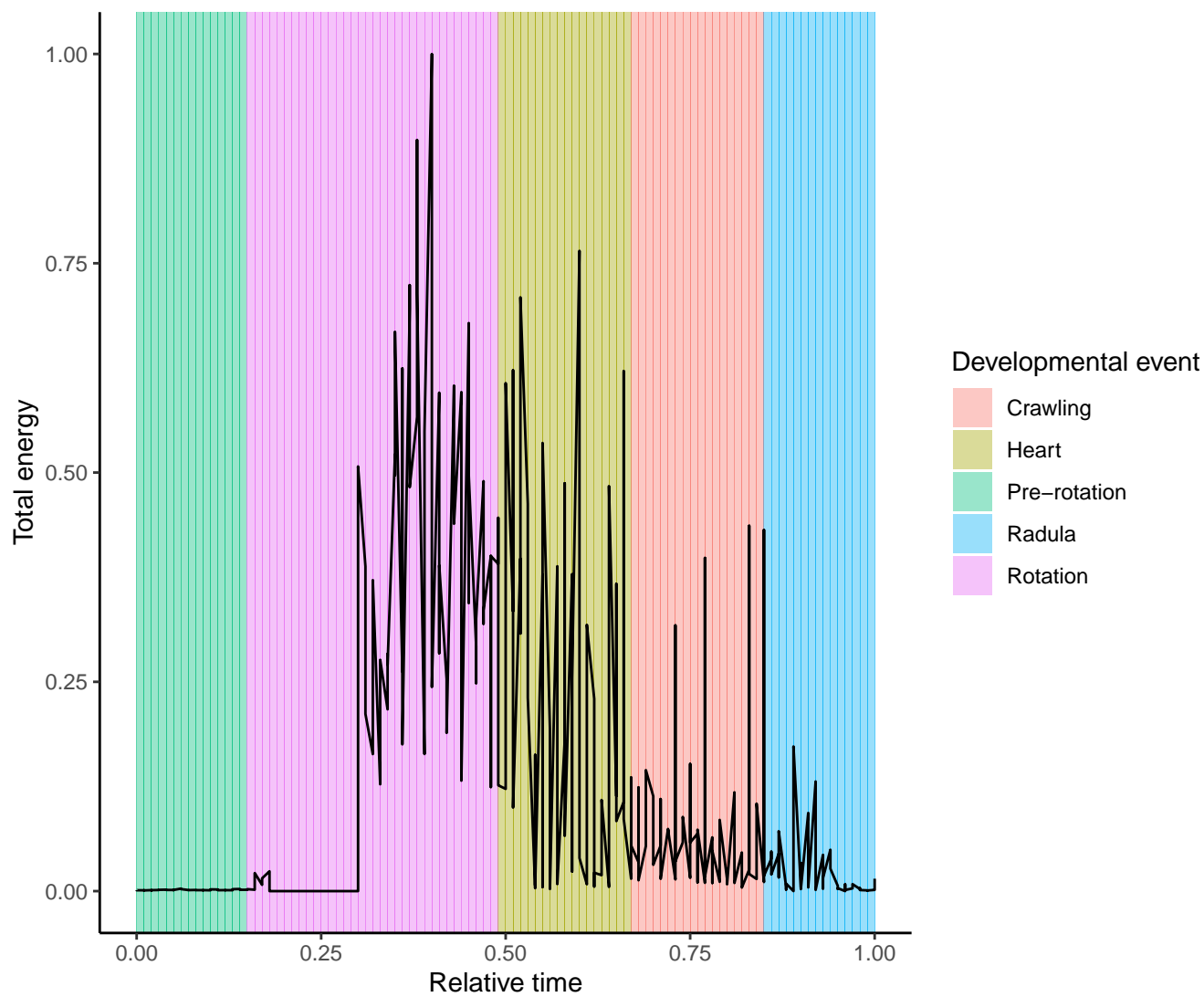

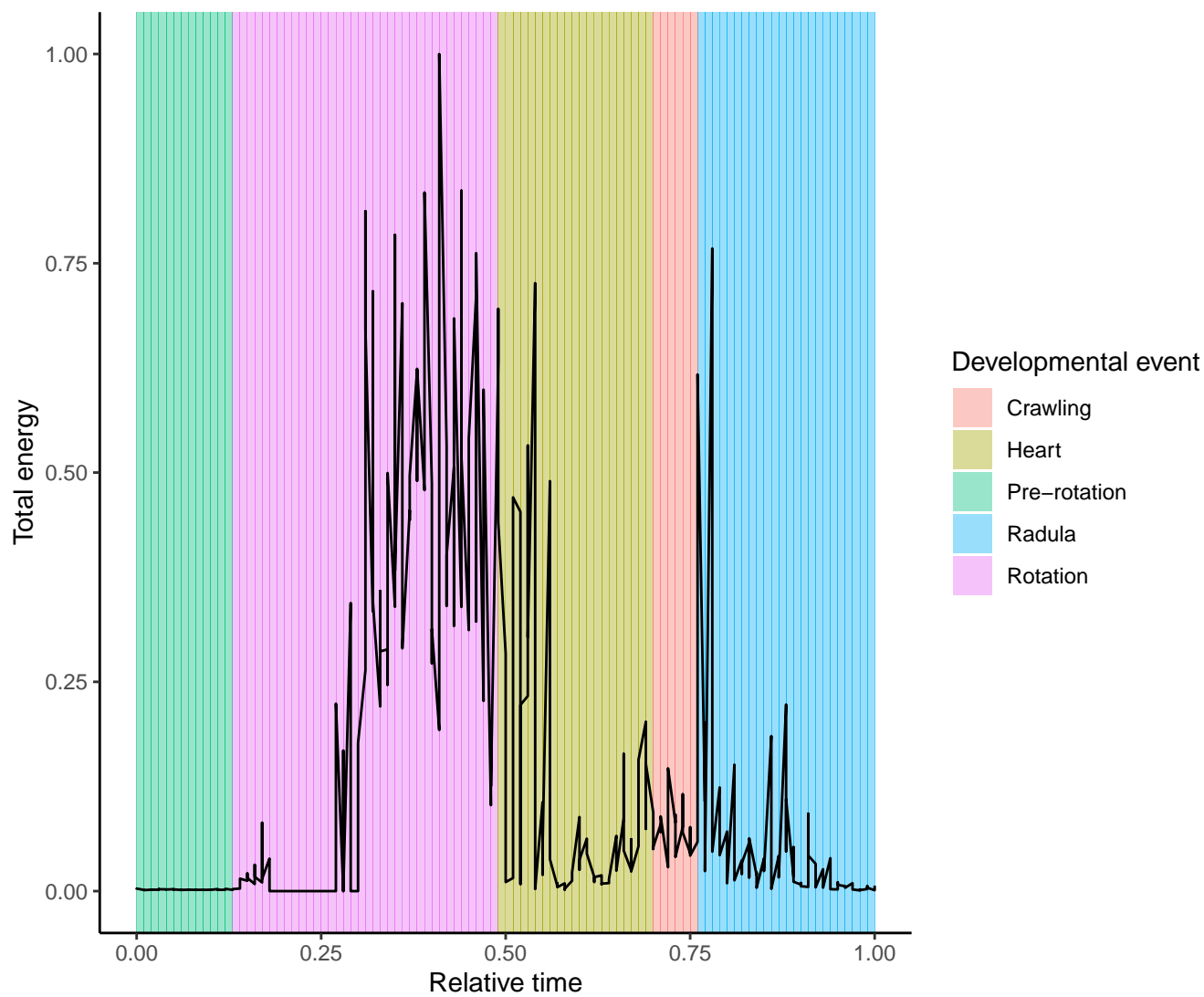

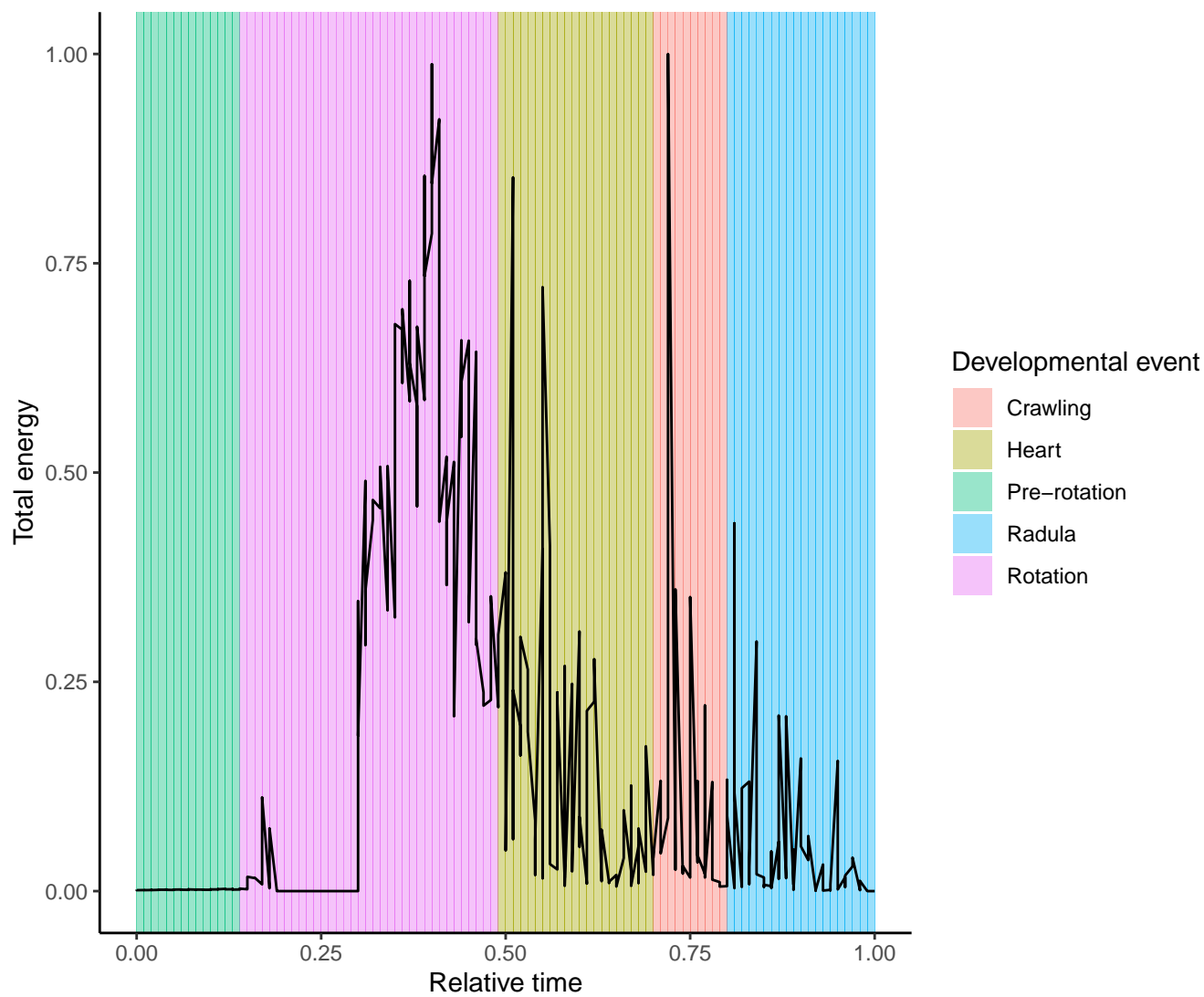

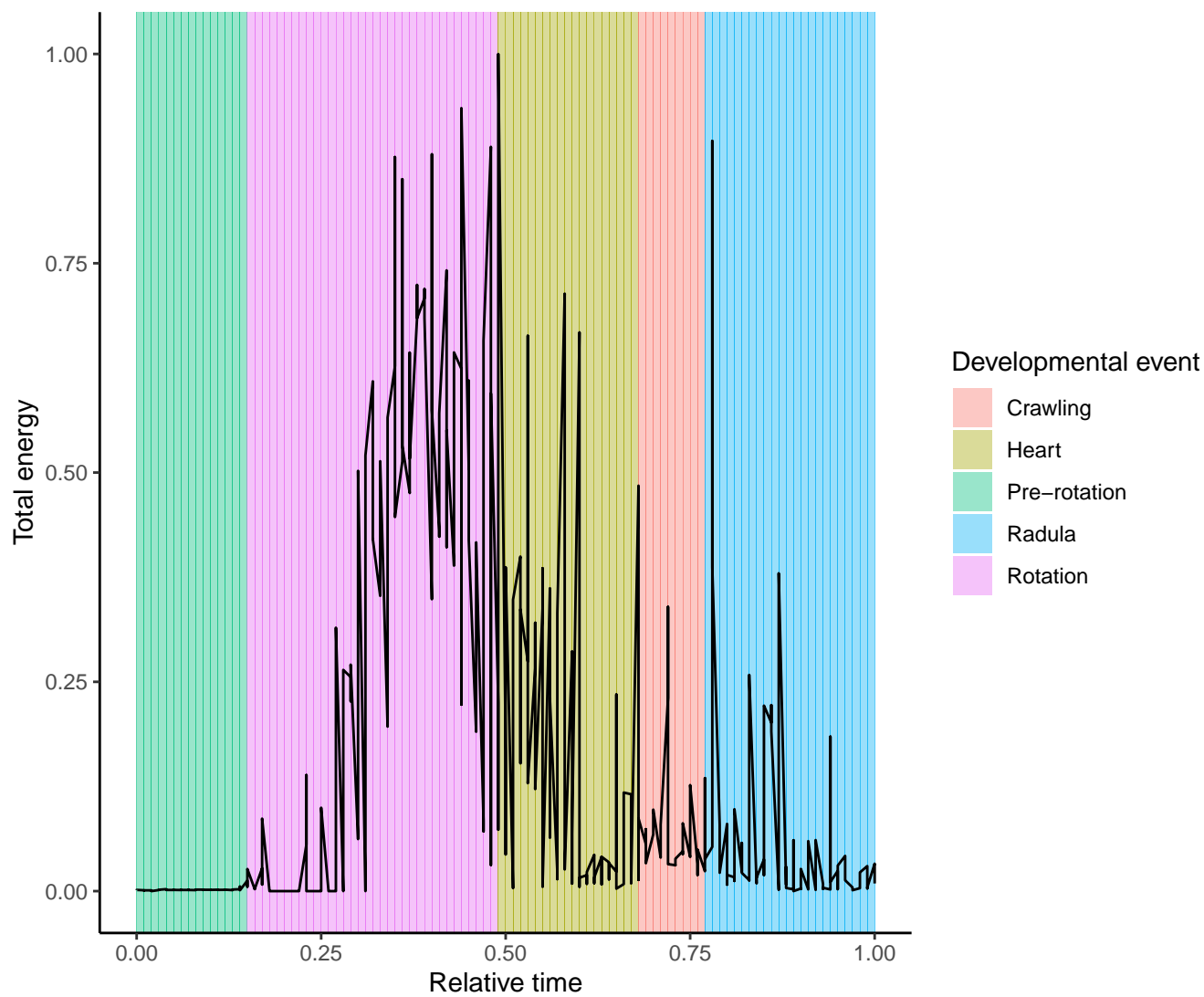

Supplement: Supplementary file 8 [file Image1.PDF]
